# Genetic predisposition to lung cancer: comprehensive literature integration, meta-analysis, and multiple evidence assessment of candidate-gene association studies

**DOI:** 10.1038/s41598-017-07737-0

**Published:** 2017-08-21

**Authors:** Junjun Wang, Qingyun Liu, Shuai Yuan, Weijia Xie, Yuan Liu, Ying Xiang, Na Wu, Long Wu, Xiangyu Ma, Tongjian Cai, Yao Zhang, Zhifu Sun, Yafei Li

**Affiliations:** 1 Department of Epidemiology, College of Preventive Medicine, Third Military Medical University, Chongqing, People’s Republic of China; 2 Center for Clinical Epidemiology and Evidence-Based Medicine, Third Military Medical University, Chongqing, People’s Republic of China; 3 Health Sciences Research, Mayo Clinic College of Medicine, Rochester, Minnesota USA

## Abstract

More than 1000 candidate-gene association studies on genetic susceptibility to lung cancer have been published over the last two decades but with few consensuses for the likely culprits. We conducted a comprehensive review, meta-analysis and evidence strength evaluation of published candidate-gene association studies in lung cancer up to November 1, 2015. The epidemiological credibility of cumulative evidence was assessed using the Venice criteria. A total of 1018 publications with 2910 genetic variants in 754 different genes or chromosomal loci were eligible for inclusion. Main meta-analyses were performed on 246 variants in 138 different genes. Twenty-two variants from 21 genes (*APEX1* rs1130409 and rs1760944, *ATM* rs664677, *AXIN2* rs2240308, *CHRNA3* rs6495309, *CHRNA5* rs16969968, *CLPTM1L* rs402710, *CXCR2* rs1126579, *CYP1A1* rs4646903, *CYP2E1* rs6413432, *ERCC1* rs11615, *ERCC2* rs13181, *FGFR4* rs351855, *HYKK* rs931794, *MIR146A* rs2910164, *MIR196A2* rs11614913, *OGG1* rs1052133, *PON1* rs662, *REV3L* rs462779, *SOD2* rs4880, *TERT* rs2736098, and *TP5*3 rs1042522) showed significant associations with lung cancer susceptibility with strong cumulative epidemiological evidence. No significant associations with lung cancer risk were found for other 150 variants in 98 genes; however, seven variants demonstrated strong cumulative evidence. Our findings provided the most updated summary of genetic risk effects on lung cancer and would help inform future research direction.

## Introduction

Lung cancer is the most common cancer and the leading cause of cancer-related mortality around the world^1^. While smoking is the leading cause of lung cancer, genetics plays an important role as less than 20% of smokers develop this deadly disease in their lifetime^2^ and non-smokers with a family history of cancer have an increased risk of lung cancer^3^.

Genetic variants influencing lung-cancer risk fall into three categories: rare high-risk variants (prevalence of 1% or less), moderate-risk variants (prevalence of not more than 5%), and common low-risk variants (prevalence of more than 5%). Family-based linkage studies is most appropriate for high risk variants with high penetrance but more costly to conduct as lung cancer is a common disease and multiple occurrences of lung cancer in a family are less common. To date, the most concrete linkage and fine mapping studies reveal a lung-cancer susceptibility locus at 6q23–25 and *RGS17* as a possible culprit gene^4–6^.

Based on the “common disease and common variant” hypothesis, genome-wide association studies (GWAS) provide a powerful tool for investigating the genetic association of a complex disease^7^. Over the past ten years, common genetic variations at 5p15.33 (*TERT/CLPTM1L*), 6p21.33 (*BAT3/MSH5*) and 15q25.1 (*CHRNA5/CHRNA3/CHRNB4*) are identified to modify the lung cancer susceptibility in GWAS^8–13^ and GWAS-based meta-analyses^14, 15^ (eg, *TERT* rs2736100, *CHRNA3* rs8042374, *APOM* rs3117582, *MSH5* rs3131379, and *GTF2H4* rs114596632). However, these only explain less than 10% of the risk contribution to lung cancer^16^.

Candidate-gene approaches were the mainstay of genetic association studies before the GWAS era. They are relatively cost-effective and easy to perform. Over 1,000 such studies on the lung cancer susceptibility have been published for the past 25 years. However, there are a number of conflicting reports and it is very challenging to find reliable associations from these highly diverse studies. As a method for systematically integrating data from multiple studies to develop a single conclusion with greater statistical power, meta-analysis is a good way to deal with the diverse and fragmented studies. Although some meta-analyses have been performed on lung cancer, most are limited to investigating a single genetic variant, several variants in a gene, or several variants across a pathway. The recent systematic meta-analyses push the limit to all available genetic association studies in a specific disease and help to achieve a comprehensive view to the genetic contributions to the disease. Alzheimer’s disease^17^, breast cancer^18^, and colorectal cancer^19^ are a few good examples using systematic meta-analyses with consensus outcomes.

Establishing robust evidence of genetic predisposition to lung cancer risk has a potential clinical utility for not only population risk stratification but also primary prevention. The main objective of our study was to identify, consolidate, and interpret genetic associations of common variants with lung cancer using a comprehensive research synopsis and systematic meta-analysis. We attempted to systematically evaluate all published candidate-gene association studies in lung cancer following credible guidelines, which were used to guide and standardize these field synopses^20–22^. Additionally, for variants with significant associations by meta-analysis, we applied Venice criteria^21^ proposed by the Human Genome Epidemiology Network (HuGENet) to assess the epidemiological credibility of cumulative epidemiological evidence of these associations, so as to obtain more reliable results. Moreover, to get a better insight of the differences in genetic variations among populations with different characters, associations stratified by ethnicity, histological types, and smoking status were also examined.

## Results

**Figure 1:**
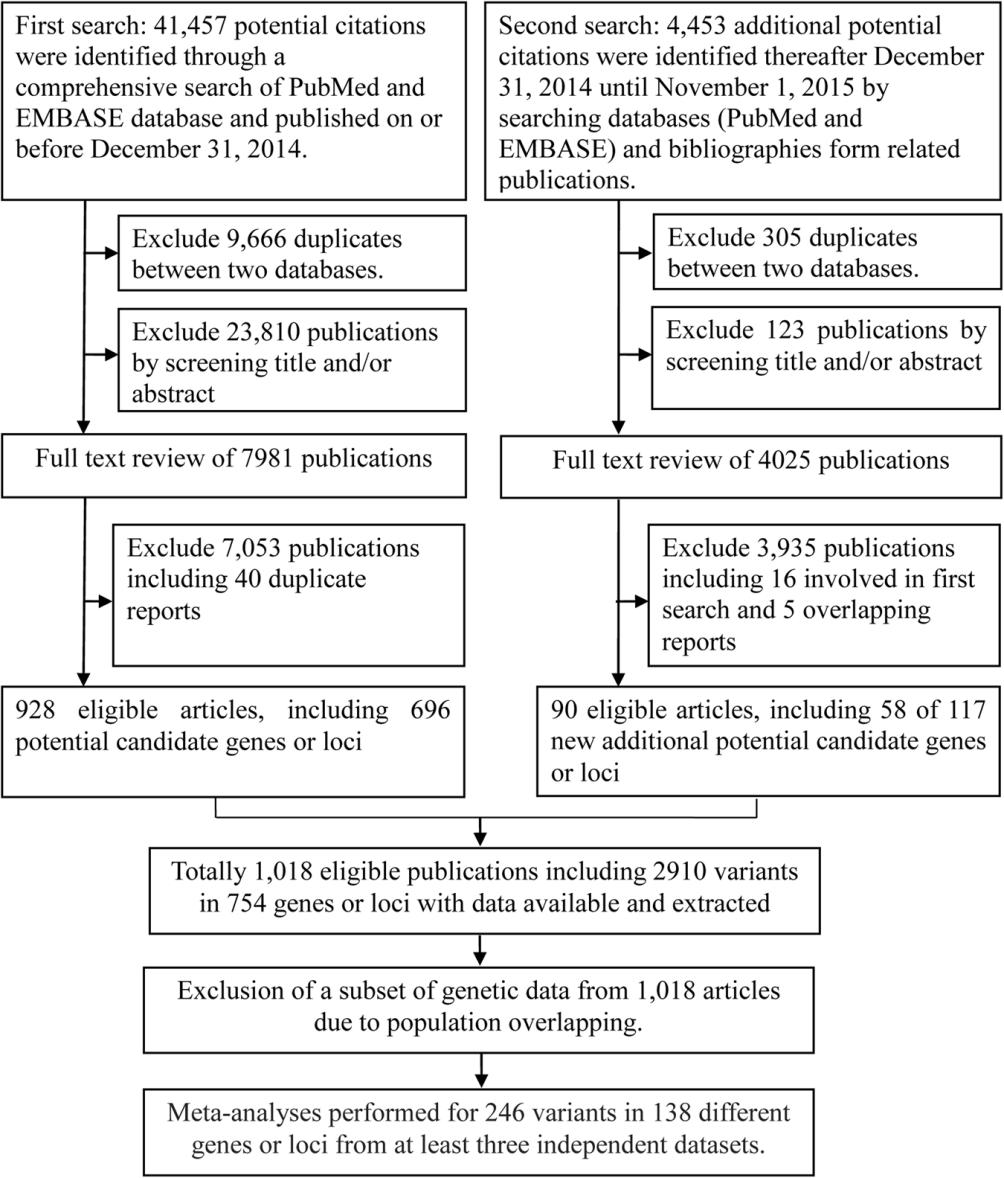
Flowchart of literature search and selection for meta-analyses for candidate-gene association studies of lung cancer.

Among the final 1,018 eligible publications for our meta-analysis (Fig. 1), vast majority (n = 926, 91%) were published after 1999, and 684 (67%) of these papers were published over the past decade (2006~2015) **(**Supplementary Fig. S1 **)**. A total of 2,910 genetic variants from 754 unique candidate genes or loci were eligible for further analyses. The included studies had a mean of 414 cases (range 13–4257) and 565 controls (range 12–55823). Among the 2,910 variants, 254 were reported in at least three independent datasets, and eight had been reported as the top association variants with lung cancer (P < 5 × 10^−8^) in published GWAS^8, 9, 23, 24^. Therefore, our meta-analyses were focused on the remaining 246 genetic variants in 138 genes or loci **(**Supplementary Table S1 **)**. More detailed information of the variants was presented in the Supplementary Results.

### Main meta-analyses

For the 246 variants, we first conducted 246 main meta-analyses, one for each variant. On average, these analyses had 6,315 subjects (range 397–71120) and were combined from eight studies (range 3–133) (Supplementary Table S1). The allelic model was performed for all but nine because of insufficient available data from the original studies (Supplementary Table S1). Of the 246 main meta-analyses, 56 variants within 45 different genes showed nominally significant genetic associations with lung cancer (*p*-value < 0.05) (Table 1, Supplementary Table S2). The strength of association between each genetic variant and lung cancer as measured by ORs had the mean of 1.36 (range 1.08–2.55) for putative “risk” variants and 0.78 (range 0.55–0.90) for putative “protective” variants. Of the 56 main meta-analyses with significant results, 24 had little or no heterogeneity, 16 had evidence of potential bias (publication bias, small study effects, or excess significance bias), and 16 were lack of robustness based on the sensitivity analyses. More details of the results were presented in the Supplementary Results.

**Table 1:** Genetic variants with significant associations with lung cancer risk in main meta-analyses (Continued on next page)

Genes	Variants*	Frequency (%)^†^	Ethnicity	Number evaluated		Genetic associations with lung cancer			Heterogeneity		Begg *P*	Venice criteria grades^∫^	Credibility of evidence^§^
				Studies	Cases/Controls	Contrast^¶﻿^	OR(95%CI)	p value	I^2^ (%)	P_Q_^ǁ^			
*APEX1*	rs1760944(A/C)	47.94	All	8	3588/3783	A vs C	1.16(1.08–1.25)	2.85 × 10^–5^	9	0.360	0.386	AAA	Strong
*AXIN2*	rs2240308(T/C)	37.40	All	3	758/742	T vs C	0.73(0.63–0.85)	6.39 × 10^−5^	0	0.398	1.000	AAA	Strong
*CHRNA3*	rs6495309(T/C)	38.44	All	4	3381/4244	T vs C	0.83(0.77–0.89)	6.55 × 10^−8^	0	0.427	1.000	AAA	Strong
*CXCR2*	rs1126579(T/C)	55.45	All	3	942/964	T vs C	0.84(0.74–0.96)	0.009	0	0.967	1.000	AAA	Strong
*CYP2E1*	rs6413432(A/T)	22.17	All	14	2944/3347	A vs T	0.78(0.71–0.85)	6.76 × 10^−8^	0	0.821	0.827	AAA	Strong
*HYKK*	rs931794(G/A)	32.89	All	5	2435/3180	G vs A	1.23(1.14–1.34)	1.85 × 10^−7^	0	0.864	1.000	AAA	Strong
*PON1*	rs662(A/G)	46.70	All	3	995/834	A vs G	0.77(0.67–0.88)	2.02 × 10^−4^	0	0.701	1.000	AAA	Strong
*REV3L*	rs462779(T/C)	39.36	Asian^‡^	4	1937/2335	T vs C	1.11(1.02–1.22)	0.021	0	0.911	0.734	AAC	Strong
*ATM*	rs189037(A/G)	42.68	Asian^‡^	5	3036/3415	A vs G	1.09(1.00–1.18)	0.050	29	0.227	0.806	ABC	Moderate
*CD3EAP*	rs967591(A/G)	32.09	All	3	676/726	A vs G	1.23(1.01–1.49)	0.036	22	0.278	1.000	BAA	Moderate
*CYP2A6*	rs1801272(A/T)	3.99	Caucasian^‡^	3	2411/2644	carriers vs non-carriers	0.66(0.52–0.84)	0.001	0	0.674	1.000	BAB	Moderate
*HIF1A*	rs11549467(A/G)	9.45	All	3	509/566	A vs G	2.27(1.74–2.96)	1.62 × 10^−9^	0	0.481	0.296	BAA	Moderate
*PDCD5*	rs1862214(G/C)	32.06	All	3	737/683	G vs C	1.32(1.12–1.56)	0.001	0	0.395	0.296	BAB	Moderate
*PROM1*	rs2240688(C/A)	27.37	Asian^‡^	3	2332/2457	C vs A	0.83(0.76–0.91)	6.92 × 10^−5^	0	0.991	0.296	AAB	Moderate
*TP53*	rs12951053(G/T)	9.93	All	3	475/569	G vs T	1.57(1.11–2.23)	0.011	37	0.203	0.296	BBB	Moderate
*TP63*	rs10937405(T/C)	42.62	All	4	4927/8794	T vs C	0.87(0.81–0.94)	2.20 × 10^−4^	34	0.207	0.308	ABA	Moderate
*WWOX*	CNV-67048	2.86	Asian^‡^	4	2942/3074	0 copy vs 2 copies	2.06(1.58–2.70)	1.20 × 10^−7^	0	0.911	1.000	BAB	Moderate
*XRCC1*	rs3213255(G/A)	38.15	All	3	1089/1506	G vs A	1.21(1.08–1.35)	0.001	0	0.457	0.296	AAB	Moderate
*AGER*	rs1800624(A/T)	34.41	Asian^‡^	3	1656/1693	A vs T	1.18(1.04–1.33)	0.010	16	0.305	1.000	AAC	Weak
*BCL2*	rs2279115(A/C)	43.37	All	5	1847/2367	A vs C	0.65(0.46–0.91)	0.011	91	0.000	0.624	ACC	Weak
*CHRNA3*	rs578776(T/C)	31.98	All	3	1245/2009	T vs C	0.87(0.77–0.98)	0.018	0	0.908	1.000	AAC	Weak
*CHRNA3*	rs938682(C/T)	28.37	All	3	1240/1986	C vs T	0.86(0.76–0.96)	0.009	0	0.582	0.296	AAC	Weak
*CHRNA3*	rs12914385(T/C)	35.09	All	4	5356/2873	T vs C	1.20(1.01–1.44)	0.044	76	0.007	0.734	ACA	Weak
*CHRNA5*	rs16969968(A/G)	32.51	All	11	6222/62452	A vs G	1.23(1.06–1.43)	0.007	80	0.000	0.119	ACC	Weak
*CLPTM1L*	rs402710(T/C)	32.92	All	13	7214/8051	T vs C	0.89(0.83–0.95)	2.63 × 10^−4^	38	0.078	0.669	ABC	Weak
*CYP1A1*	rs4646903(C/T)	21.88	All	57	9844/12410	C vs T	1.16(1.07–1.25)	1.59 × 10^−4^	55	0.000	0.772	ACC	Weak
*CYP1A1*	rs1048943(G/A)	17.83	All	54	9869/12114	G vs A	1.23(1.11–1.36)	7.64 × 10^−5^	67	0.000	0.649	ACC	Weak
*CYP1B1*	rs1056836(G/C)	38.50	All	12	3033/3866	G vs C	1.13(1.05–1.22)	0.002	0	0.551	0.064	AAC	Weak
*CYP2A6*	rs5031016(C/T)	9.89	All	3	1527/1138	C vs T	0.57(0.33–1.00)	0.048	73	0.025	0.296	BCC	Weak
*CYP2E1*	rs2031920(T/C)	17.33	All	23	4983/6628	T vs C	0.86(0.76–0.97)	0.018	50	0.003	0.509	ACA	Weak
*ELANE*	rs351107(G/T) (−903T > G, Rep_a)	5.31	Caucasian^‡^	3	745/762	G vs T	0.55(0.34–0.87)	0.011	29	0.246	1.000	BBC	Weak
*ELANE*	rs7254054(A/G) (−741G > A, Rep_b)	27.20	Caucasian^‡^	3	754/750	A vs G	0.77(0.61–0.97)	0.030	46	0.155	0.296	BBC	Weak
*ERCC1*	rs11615(C/T)	51.18	All	12	5731/7058	C vs T	0.90(0.83–0.99)	0.023	52	0.018	0.086	ACC	Weak
*ERCC2*	rs238406(A/C)	40.05	All	6	1754/2688	A vs C	1.12(1.02–1.23)	0.013	0	0.558	0.260	AAC	Weak
*ERCC2*	rs13181(C/A)	25.26	All	40	13111/16749	C vs A	1.12(1.05–1.19)	4.18 × 10^−4^	49	0.000	0.753	ABC	Weak
*ERCC5*	rs1047768(T/C)	43.99	All	4	1449/2248	T vs C	0.86(0.74–1.00)	0.049	48	0.123	0.734	ABC	Weak
*ERCC6*	rs3793784(G/C)	30.82	All	3	1643/1689	G vs C	0.75(0.60–0.92)	0.007	68	0.044	1.000	ACA	Weak
*FGFR4*	rs351855(A/G)	42.47	All	4	1083/1275	A vs G	0.82(0.69–0.98)	0.025	33	0.214	0.089	ABC	Weak
*GSTM1*	Present/null	48.85	All	133	33253/37867	null vs present	1.18(1.12–1.23)	2.54 × 10^−11^	52	0.000	0.105	ACC	Weak
*GSTP1*	rs1695(G/A)	30.41	All	46	12521/14411	G vs A	1.08(1.02–1.15)	0.011	55	0.000	0.075	ACC	Weak
*GSTT1*	GSTT1	26.14	All	77	23009/25365	null vs present	1.10(1.02–1.19)	0.011	58	0.000	0.346	ACC	Weak
*HRAS1*	VNTR(common alleles/rare alleles)	7.03	Caucasian^‡^	4	746/1174	rare vs common	2.55(1.01–6.45)	0.048	69	0.023	0.734	BCC	Weak
*IL10*	rs1800896(G/A)	37.18	All	10	2861/3817	G vs A	1.29(1.05–1.59)	0.017	75	0.000	0.074	ACC	Weak
*MAPKAPK2*	CNV-30450	9.76	Asian^‡^	3	2332/2480	4 copies vs 2 copies	1.60(1.04–2.45)	0.031	81	0.005	1.000	BCB	Weak
*MDM2*	rs2279744(G/T)	41.05	All	19	11076/14434	G vs T	1.10(1.01–1.19)	0.021	75	0.000	0.700	ACC	Weak
*MIR146A*	rs2910164(C/G)	45.26	All	6	3158/3225	C vs G	1.16(1.06–1.27)	0.001	21	0.274	0.260	AAC	Weak
*MMP2*	rs243865(T/C)	16.77	All	3	1751/1729	T vs C	0.63(0.45–0.89)	0.009	80	0.007	0.296	BCC	Weak
*MTRR*	rs1801394(G/A)	43.28	All	3	1668/2291	G vs A	1.13(1.03–1.24)	0.011	0	0.525	1.000	AAC	Weak
*NOD2*	rs2066847 (3020insC/-)	0.50	All	3	807/4078	carriers vs non-carriers	1.42(1.07–1.90)	0.017	0	0.593	1.000	× AC	Weak
*SFTPB*	wild type/ variation	5.83	All	3	157/240	variation vs wild	1.92(1.11–3.33)	0.020	0	0.960	0.296	CAB	Weak
*SOD2*	rs4880(T/C)	51.48	All	9	3738/4467	T vs C	1.20(1.06–1.36)	0.005	61	0.009	0.348	ACA	Weak
*TERT*	rs2736098(A/G)	33.01	All	7	4660/4825	A vs G	1.20(1.08–1.33)	0.001	67	0.006	0.548	ACB	Weak
*UGT1A6*	rs6759892(G/T)	25.10	All	3	266/261	G vs T	2.27(1.14–4.53)	0.020	84	0.002	1.000	BCA	Weak
*XRCC1*	rs1001581(T/C)	34.52	All	5	851/1166	T vs C	1.17(1.00–1.37)	0.044	28	0.232	0.221	ABC	Weak
*XRCC1*	rs1799782(T/C)	18.19	All	30	11096/13772	T vs C	0.90(0.82–0.98)	0.022	62	0.000	0.372	ACC	Weak
*XRCC1*	rs3213245(C/T)	11.03	All	5	2795/2865	C vs T	1.29(1.04–1.59)	0.020	68	0.014	0.806	ACC	Weak

OR = odds ratio; 95% CI = 95% confidence interval. VNTR = variable number of tandem repeats. CNV = copy number variation. ins = insertion. *Minor alleles/major alleles (per Caucasian); majors alleles were treated as reference alleles in the analyses. ^†^Frequency of minor allele or effect genotype (s) in controls in main meta-analyses. ^¶^Allelic contrast or phenotype trait for common variants; genetic comparison for rare variants or variants only with genotype group data. ^ǁ^P value of the test for between-study heterogeneity. ^∫^Venice criteria grades are for amount of evidence, replication of the association, and protection from bias; one rare variant was not scored for amount of evidence (×). ^§^Credibility of evidence is categorized as “strong”, “moderate”, or “weak” for association with lung cancer risk. ^‡^Only Asian or Caucasian data were available for meta-analysis.

The credibility assessment of the cumulative epidemiological evidence found eight genetic variants (*APEX1* rs1760944, *AXIN2* rs2240308, *CHRNA3* rs6495309, *CXCR2* rs1126579, *CYP2E1* rs6413432, *HYKK* rs931794, *PON1* rs662, and *REV3L* rs462779) were strong and ten were moderate (*ATM* rs189037, *CD3EAP* rs967591, *CYP2A6* rs1801272, *HIF1A* rs11549467, *PDCD5* rs1862214, *PROM1* rs2240688, *TP53* rs12951053, *TP63* rs10937405, *WWOX* CNV-67048, and *XRCC1* rs3213255) (Table 1, Supplementary Table S2).

In the dominant genetic model analyses (Supplementary Table S1), 44 variants showed significant associations with lung cancer risk, of which seven had non-significant association in the main allelic meta-analyses yet, interestingly, two (*ATM* rs66467 and *REV3L* rs465646) showed strong and moderate cumulative epidemiological﻿ evidence, respectively (Table 2, Supplementary Table S2). Under the recessive model, 39 variants showed statistically significant associations, of which ten were non-significant under an allelic model. However, none of these showed strong cumulative epidemiologic evidence, although five variants (*CASC8* rs6983267, *CHRNA5* rs142774214, *CYP2A6* non*4/*4, *IL17A* rs2275913, and *XPA* rs1800975) showed moderate evidence (Table 2).

**Table 2:** Genetic variants with significant associations with lung cancer risk under a dominant or recessive genetic model.

Genes	Variants	Alleles*	MAF (%)	Number evaluated		Genetic associations with lung cancer			Heterogeneity		Begg *P*	Venice criteria grades^†^	Credibility of evidence^‡^
				Studies	Cases/Controls	Genetic models	OR(95%CI)	p value	I^2^ (%)	P_Q_^ǁ^			
*ATM*	rs664677	C/T	58.90	3	1627/1641	Dominant	0.76(0.64–0.92)	0.004	0	0.448	1.000	AAA	Strong
*REV3L*	rs465646	C/T	18.18	3	1296/1511	Dominant	0.78(0.67–0.92)	0.003	0	0.437	1.000	BAB	Moderate
*CASC8*	rs6983267	G/T	44.77	3	1539/1989	Recessive	1.22(1.04–1.44)	0.013	0	0.644	0.296	BAA	Moderate
*CHRNA5*	rs142774214	ins/-	37.67	3	1431/1606	Recessive	0.80(0.65–0.98)	0.032	0	0.597	1.000	BAA	Moderate
*CYP2A6*	non*4/*4	del/-	13.48	7	2623/2380	Recessive	0.51(0.35–0.73)	2.93 × 10^−4^	0	0.539	1.000	BAA	Moderate
*IL17A*	rs2275913	A/G	24.90	3	889/998	Recessive	1.76(1.21–2.55)	0.003	18	0.295	0.296	BAB	Moderate
*XPA*	rs1800975	A/G	36.74	12	4221/5240	Recessive	1.22(1.05–1.42)	0.011	33	0.124	0.681	ABA	Moderate
*Chr8q24*	rs16901979	A/C	19.48	3	1534/1992	Dominant	1.18(1.02–1.37)	0.025	0	0.610	1.000	AAC	Weak
*CYP1B1*	rs10012	G/C	25.98	3	622/666	Dominant	1.69(1.05–2.72)	0.031	74	0.021	1.000	BCC	Weak
*EGF*	rs4444903	G/A	59.28	3	666/690	Dominant	2.07(1.01–4.24)	0.048	79	0.009	0.296	ACC	Weak
*MLH1*	rs1800734	A/G	48.86	5	2178/2320	Dominant	0.80(0.68–0.95)	0.009	24	0.260	0.462	AAC	Weak
*PTGS2*	rs689466	G/A	38.07	4	1676/2180	Dominant	0.78(0.62–0.97)	0.026	56	0.076	0.734	ACA	Weak
*FASLG*	rs763110	T/C	34.01	5	4436/4120	Recessive	0.83(0.70–0.99)	0.038	30	0.221	0.462	ABC	Weak
*IL1B*	rs1143627	C/T	38.81	8	4201/5431	Recessive	0.80(0.68–0.95)	0.010	49	0.059	0.019	ABC	Weak
*LIG1*	rs156641	A/G	31.71	3	1112/2048	Recessive	1.45(1.14–1.83)	0.002	0	0.370	1.000	BAC	Weak
*XRCC1*	rs25487	A/G	29.70	48	16999/20567	Recessive	1.16(1.03–1.30)	0.018	54	0.000	0.729	ACC	Weak
*XRCC3*	rs1799794	G/A	41.09	4	1389/1941	Recessive	0.82(0.67–0.99)	0.038	0	0.469	1.000	BAC	Weak

MAF = minor allele frequency in controls. OR = odds ratio; 95% CI = 95% confidence interval. chr = chromosome. ins = insertion. del = deletion. bp = base pair. *Minor alleles/major alleles (per Caucasian); major alleles were treated as reference alleles in the analyses; Dominant model, summary OR was estimated for subjects who carry one or two minor alleles. Recessive model, summary OR was estimated for subjects have homozygous of the minor alleles. ^ǁ^P value of the test for between-study heterogeneity. ^†^Venice criteria grades are for amount of evidence, replication of the association, and protection from bias; one rare variant was not scored for amount of evidence (×). ^‡^Credibility of evidence is categorized as “strong”, “moderate”, or “weak” for association with lung cancer risk.

### Subgroup meta-analyses

#### Ethnicity

Subgroup meta-analyses were conducted in Caucasian and Asian population separately under each of the three genetic models (allelic, dominant, or recessive model) depending on the available data **(**Supplementary Table S3 **)**. We found that 19 and 26 variants were significantly associated with lung cancer susceptibility in Caucasian and Asian population, respectively. Five variants (*APEX1* rs1130409, *CHRNA5* rs16969968, *CLPTM1L* rs402710, *ERCC2* rs13181, and *SOD2* rs4880) showed strong and five (*CYP1A2* rs762551, *CYP1B1* rs1056836, *CYP2A6* rs1801272, *CYP2E1* rs2031920, and *XRCC1* rs1799782) showed moderate evidence in the Caucasian population (Table 3, Supplementary Table S4). For the significant variants in the Asian population, strong and moderate cumulative evidence were observed in seven (*APEX1* rs1760944, *CLPTM1L* rs402710, *CYP2E1* rs6413432, *MIR146A* rs2910164, *MIR196A2* rs11614913, *REV3L* rs462779, and *TERT* rs2736098) and seven variants (*ATM* rs189037, *CHRNA3* rs6495309, *CYP2A6* non*4/*4, *GSTT1* present/null, *PROM1* rs2240688, *REV3L* rs465646, and *WWOX* CNV-67048), respectively (Table 3, Supplementary Table S4). Comparing the significant variants across ethnic groups, we found that 13 variants (*AGER* rs1800624, *ATM* rs189037, *CYP2A6* non*4/*4, *FASLG* rs763110, *IL10* rs1800872, *MAPKAPK2* CNV-30450, *MIR196A2* rs11614913, *PROM1* rs2240688, *REV3L* rs462779, *REV3L* rs465646, *VEGFA* rs833061, *WWOX* CNV-67048, and *XRCC1* rs25487) were unique to the Asian population, and seven (*APEX1* rs1130409, *CYP1A2* rs762551, *CYP2A6* rs1801272, *ELANE* rs351107, *ELANE* rs7254054, *HRAS1* a VNTR variation, and *MTHFR* rs1801131) to Caucasian population. Four variants (*CLPTM1L* rs402710, *CYP1A1* rs4646903, *CYP1A1* rs1048943, and *GSTM1* present/null) shared between the two groups, including one (*CLPTM1L* rs402710) showed consistent strong evidence of significant associations in both groups (Supplementary Fig. S2).

**Table 3:** Genetic variants with significant associations with lung cancer risk in subgroup meta-analyses with strong or moderate cumulative evidence (Continued on next page).

Gene	Subgroup	Variants*	Number evaluated		Lung-cancer risk meta-analysis			Heterogeneity		Begg *P*	Venice criteria grades^∫^	Credibility of evidence^§^
			Studies	Cases/Controls	Genetic models	OR(95%CI)	p value	I^2^ (%)	P_Q_^ǁ^			
*APEX1*	Caucasian	rs1130409(G/T)	7	1807/3065	Recessive	0.84(0.72–0.97)	0.021	0	0.695	0.764	AAA	Strong
*CHRNA5*	Caucasian	rs16969968(A/G)	6	3305/59780	Allelic	1.35(1.27–1.44)	2.03 × 10^−21^	0	0.958	0.990	AAA	Strong
*CLPTM1L*	Caucasian	rs402710(T/C)	4	1801/1908	Allelic	0.86(0.78–0.94)	0.002	0	0.532	0.734	AAA	Strong
*ERCC2*	Caucasian	rs13181(C/A)	18	5967/8851	Recessive	1.15(1.04–1.29)	0.009	16	0.258	0.495	AAA	Strong
*SOD2*	Caucasian	rs4880(T/C)	4	3185/3966	Allelic	1.17(1.10–1.25)	2.24 × 10^−6^	0	0.973	0.406	AAA	Strong
*CYP1A2*	Caucasian	rs762551(C/A)	3	869/1468	Recessive	1.69(1.20–2.36)	0.002	30	0.232	1.000	BBA	Moderate
*CYP1B1*	Caucasian	rs1056836(G/C)	6	1849/2655	Dominant	1.18(1.04–1.34)	0.010	0	0.856	0.711	AAB	Moderate
*CYP2A6*	Caucasian	rs1801272(A/T)	3	2411/2644	Dominant	0.66(0.52–0.84)	0.001	0	0.674	1.000	BAB	Moderate
*CYP2E1*	Caucasian	rs2031920(T/C)	6	665/1224	Allelic	0.61(0.42–0.90)	0.013	0	0.456	0.837	BAB	Moderate
*XRCC1*	Caucasian	rs1799782(T/C)	12	4740/6868	Allelic	0.84(0.72–0.98)	0.028	28	0.172	0.790	ABA	Moderate
*APEX1*	Asian	rs1760944(A/C)	5	3071/3038	Allelic	1.20(1.12–1.29)	9.14 × 10^−7^	0	0.717	0.462	AAA	Strong
*CLPTM1L*	Asian	rs402710(T/C)	8	5413/6143	Dominant	0.84(0.77–0.92)	1.53 × 10^−4^	17	0.296	0.711	AAA	Strong
*CYP2E1*	Asian	rs6413432(A/T)	6	1964/2085	Allelic	0.78(0.70–0.86)	1.31 × 10^−6^	0	0.824	0.707	AAA	Strong
*MIR146A*	Asian	rs2910164(C/G)	4	2807/2841	Recessive	1.23(1.09–1.39)	0.001	0	0.594	1.000	AAA	Strong
*MIR196A2*	Asian	rs11614913(C/T)	4	2376/2413	Dominant	1.22(1.07–1.38)	0.002	0	0.444	0.308	AAA	Strong
*REV3L*	Asian	rs462779(T/C)	4	1937/2335	Allelic	1.11(1.02–1.22)	0.021	0	0.911	0.734	AAC	Strong
*TERT*	Asian	rs2736098(A/G)	5	3829/3992	Dominant	1.26(1.14–1.39)	1.03 × 10^−5^	0	0.896	1.000	AAA	Strong
*ATM*	Asian	rs189037(A/G)	5	3036/3415	Allelic	1.09(1.00–1.18)	0.050	29	0.227	0.806	ABC	Moderate
*CHRNA3*	Asian	rs6495309(T/C)	3	2635/2767	Allelic	0.83(0.76–0.91)	6.17 × 10^−5^	27	0.254	1.000	ABA	Moderate
*CYP2A6*	Asian	*4/non*4	6	2517/2264	Recessive	0.52(0.36–0.75)	0.001	0	0.454	0.707	BAA	Moderate
*GSTT1*	Asian	null/present	14	7043/5289	Allelic	1.15(1.03–1.28)	0.010	34	0.105	0.827	ABA	Moderate
*PROM1*	Asian	rs2240688(C/A)	3	2332/2457	Allelic	0.83(0.76–0.91)	6.92 × 10^−5^	0	0.991	0.296	AAB	Moderate
*REV3L*	Asian	rs465646(C/T)	3	1296/1511	Allelic	0.83(0.71–0.97)	0.016	14	0.311	1.000	BAB	Moderate
*WWOX*	Asian	CNV-67048	4	2942/3074	0 copy vs 2 copies	2.06(1.58–2.70)	1.20 × 10^−7^	0	0.911	1.000	BAB	Moderate
*CYP1A1*	SCLC	rs4646903(C/T)	12	273/2545	Recessive	1.71(1.08–2.71)	0.021	0	0.904	0.244	BAA	Moderate
*GSTM1*	SCLC	null/present	26	1224/7255	Allelic	1.30(1.09–1.56)	0.004	43	0.010	1.000	ABA	Moderate
*CHRNA5*	NSCLC	rs16969968(A/G)	6	3201/4736	Allelic	1.36(1.24–1.48)	1.48 × 10^−11^	13	0.329	0.707	AAA	Strong
*CLPTM1L*	NSCLC	rs402710(T/C)	6	2940/4040	Allelic	0.85(0.79–0.91)	1.13 × 10^−5^	0	0.666	1.000	AAA	Strong
*CYP2E1*	NSCLC	rs6413432(A/T)	6	1290/1809	Allelic	0.80(0.71–0.91)	4.90 × 10^−4^	0	0.868	1.000	AAA	Strong
*ERCC1*	NSCLC	rs11615(C/T)	3	780/811	Allelic	0.68(0.58–0.81)	1.01 × 10^−5^	13	0.316	0.296	AAA	Strong
*FGFR4*	NSCLC	rs351855(A/G)	3	985/1230	Allelic	0.76(0.68–0.86)	1.97 × 10^−5^	0	0.590	1.000	AAA	Strong
*HYKK*	NSCLC	rs931794(G/A)	4	1548/2464	Allelic	1.25(1.13–1.37)	9.08 × 10^−6^	0	0.880	0.734	AAA	Strong
*MIR146A*	NSCLC	rs2910164(C/G)	4	880/1094	Allelic	1.28(1.11–1.46)	4.63 × 10^−4^	0	0.391	0.734	AAA	Strong
*TERT*	NSCLC	rs2736098(A/G)	4	2002/2490	Allelic	1.30(1.19–1.42)	2.59 × 10^−9^	0	0.818	0.734	AAA	Strong
*IL17A*	NSCLC	rs2275913(A/G)	3	780/998	Recessive	1.72(1.12–2.65)	0.013	31	0.235	0.296	BBB	Moderate
*TP63*	NSCLC	rs10937405(T/C)	3	3587/8484	Allelic	0.87(0.82–0.92)	9.91 × 10^−7^	0	0.595	1.000	AAB	Moderate
*XPC*	NSCLC	PAT-/ + (ins/non-ins)	3	967/1340	Recessive	1.46(1.17–1.81)	0.001	0	0.483	1.000	BAA	Moderate
*XRCC1*	NSCLC	rs3213245(C/T)	3	1744/2178	Dominant	1.50(1.29–1.75)	1.89 × 10^−7^	0	0.683	0.296	BAA	Moderate
*CYP2E1*	AD	rs6413432(A/T)	6	500/1809	Allelic	0.79(0.66–0.95)	0.011	0	0.664	0.707	AAA	Strong
*OGG1*	AD	rs1052133(G/C)	12	3603/6677	Recessive	1.25(1.10–1.43)	0.001	20	0.246	0.945	AAA	Strong
*TERT*	AD	rs2736098(A/G)	4	1214/2490	Allelic	1.40(1.26–1.54)	4.97 × 10^−11^	0	0.891	0.308	AAA	Strong
*TP53*	AD	rs1042522(C/G)	22	3504/8822	Recessive	1.20(1.05–1.38)	0.008	16	0.245	0.143	AAA	Strong
*CHRNA5*	AD	rs16969968(A/G)	4	1507/2834	Allelic	1.37(1.14–1.64)	0.001	33	0.214	0.734	ABA	Moderate
*ERCC2*	AD	rs13181(C/A)	4	664/1230	Dominant	1.35(1.06–1.70)	0.013	0	0.635	0.734	BAA	Moderate
*IL17A*	AD	rs2275913(A/G)	3	469/998	Recessive	1.84(1.11–3.06)	0.018	36	0.211	1.000	BBB	Moderate
*MDM2*	AD	rs2279744(G/T)	6	1714/4083	Recessive	1.28(1.04–1.56)	0.018	46	0.098	0.707	ABA	Moderate
*TP63*	AD	rs10937405(T/C)	3	1158/8484	Allelic	0.82(0.75–0.90)	2.91 × 10^−5^	0	0.898	0.296	AAB	Moderate
*XRCC1*	AD	rs3213245(C/T)	3	860/2178	Dominant	1.55(1.29–1.87)	4.72 × 10^−6^	0	0.758	0.296	BAA	Moderate
*CYP1A1*	SCC	rs4646903(C/T)	17	1021/3959	Allelic	1.45(1.26–1.67)	3.77 × 10^−7^	21	0.215	0.232	AAA	Strong
*CYP2E1*	SCC	rs6413432(A/T)	6	715/1809	Allelic	0.76(0.65–0.88)	3.98 × 10^−4^	0	0.911	0.260	AAA	Strong
*APEX1*	smokers	rs1760944(A/C)	3	655/647	Allelic	1.37(1.11–1.69)	0.003	43	0.174	1.000	ABA	Moderate
*CYP1A1*	smokers	rs4646903(C/T)	7	1034/1087	Allelic	1.30(1.02–1.64)	0.033	46	0.088	0.230	BBA	Moderate
*CYP2A6*	smokers	*4/non*4	3	1339/848	Allelic	0.71(0.59–0.85)	2.30 × 10^−4^	13	0.319	1.000	BAA	Moderate
*CYP2E1*	smokers	rs6413432(A/T)	3	796/791	Allelic	0.75(0.63–0.90)	0.002	2	0.360	0.296	BAA	Moderate
*CYP2E1*	smokers	rs2031920(T/C)	3	1064/1220	Allelic	0.76(0.65–0.90)	0.001	0	0.727	0.296	BAA	Moderate
*GSTP1*	smokers	rs1138272(T/C)	3	924/1026	Dominant	1.63(1.28–2.08)	9.17 × 10^−5^	0	0.459	1.000	BAA	Moderate
*NBN*	smokers	rs1805794(G/C)	3	1226/1220	Recessive	0.83(0.71–0.98)	0.030	0	0.554	0.296	BAA	Moderate
*ERCC1*	non-smokers	rs11615(C/T)	3	731/958	Allelic	0.85(0.72–0.99)	0.042	0	0.449	1.000	AAA	Strong
*CYP2E1*	non-smokers	rs6413432(A/T)	5	315/560	Dominant	0.72(0.54–0.97)	0.028	0	0.959	0.806	BAA	Moderate
*CYP2E1*	non-smokers	rs2031920(T/C)	3	304/695	Allelic	0.70(0.54–0.90)	0.005	0	0.863	1.000	BAA	Moderate
*ERCC2*	non-smokers	rs13181(C/A)	3	478/469	Dominant	1.88(1.36–2.58)	1.11 × 10^−4^	0	0.550	0.296	BAA	Moderate
*GSTM1*	non-smokers	null/present	32	1924/4718	Allelic	1.37(1.16–1.61)	1.60 × 10^−4^	41	0.009	0.212	ABA	Moderate
*TP53*	non-smokers	rs1042522(C/G)	11	1882/2887	Recessive	1.28(1.01–1.61)	0.040	39	0.088	0.586	ABA	Moderate
*XRCC1*	non-smokers	rs3213245(C/T)	3	977/1310	Dominant	1.43(1.17–1.75)	4.56 × 10^−4^	0	0.530	0.296	BAA	Moderate

OR = odds ratio; 95%CI = 95% confidence interval. ins = insertion. del = deletion. CNV = copy number variation. SCLC = small cell lung cancer. NSCLC = non-small cell lung cancer. AD = adenocarcinoma. SCC = squamous cell carcinoma. *Minor alleles/major alleles (per Caucasian); major alleles were treated as reference alleles in the analyses. ^ǁ^P value of the test for between-study heterogeneity. ^∫^Venice criteria grades are for amount of evidence, replication of the association, and protection from bias. ^§^Credibility of evidence is categorized as “strong”, “moderate”, or “weak” for association with lung cancer risk; one association with strong evidence for a variant was not considered the bias of low OR for the presence of highly consistent results across studies enrolled in meta-analysis.

#### Histological types of lung cancer

Considering the etiologic differences of different subtypes of lung cancer, subgroup meta-analyses were performed for genetic variants with data available for non-small cell lung cancer [NSCLC], small cell lung cancer [SCLC], adenocarcinoma [AD], and squamous cell carcinoma [SCC] under each of the three genetic models (allelic, dominant, or recessive model) (Supplementary Table S5). In the NSCLC subgroup, statistical significant associations were found for 25 variants where eight variants (*CHRNA5* rs16969968, *CLPTM1L* rs402710, *CYP2E1* rs6413432, *ERCC1* rs11615, *FGFR4* rs351855, *HYKK* rs931794, *MIR146A* rs2910164, and *TERT r*s2736098) demonstrated strong cumulative epidemiological evidence (Table 3, Supplementary Table S6). In the SCLC group, five variants showed significant associations but all were moderate or weak cumulative evidence. Three significant variants (*CHRNA5* rs16969968, *CYP1A1* rs4646903, and *GSTM1* present/null) shared between the NSCLC and SCLC group (Supplementary Fig. S3). For the AD group, 15 variants showed significant associations where four of them have strong evidence (*CYP2E1* rs6413432, OGG1 rs1052133, *TERT* rs2736098, and *TP53* rs1042522). As for SCC, two out of eight significant variants (*CYP1A1* rs4646903 and *CYP2E1* rs6413432) showed strong cumulative evidence. Four significant variants (*CYP2E1* rs6413432, *GSTM1* present/null, *SOD2* rs4880, and *TERT* rs2736098) were shared between the AD and SCC group, including one (*CYP2E1* rs6413432) showed consistent strong evidence of significant associations in both groups (Supplementary Fig. S4).

#### Smoking status

As for subgroup meta-analyses by smoking status, significant associations were found for twenty-two variants and ten variants in the smokers and the non-smokers, respectively. In the smoker population, the significant associations only showed moderate (*APEX1* rs1760944, *CYP1A1* rs4646903, *CYP2A6* non*4/*4, *CYP2E1* rs6413432, *CYP2E1* rs2031920, *GSTP1* rs1138272, and *NBN* rs1805794) or weak cumulative evidence, mostly due to lack of large-scale evidence and the presence of potential biases (Table 3, Supplementary Table S8). In the non-smokers populations, the significant associations had strong, moderate, or weak evidence for one (*ERCC1* rs11615), six (*CYP2E1* rs6413432, *CYP2E1* rs2031920, *ERCC2* rs13181, *GSTM1* present/null, *TP53* rs1042522, and *XRCC1 r*s3213245), and three variants, respectively. Comparing the significant variants between two groups, seventeen were unique to the smoking population, five to the non-smoking population, and five shared between the two populations (Supplementary Fig. S5).

### Functional annotations

Based on main and subgroup meta-analyses, a total of 22 variants showed significant associations to lung cancer susceptibility with strong cumulative evidence. We further performed genomic annotations for these variants using HaploReg v4.1^25^, which can help to predict the functional variants. Of them, twelve variants are located in exon, two in microRNA (miRNA), and the others in non-coding regions (four intronic, two intergenic, one 5′UTR, and one 3′UTR) (Table 4). Most of these variants are located within enhancer or promoter elements that are active across a wide range of tissue types (including lung cancer or normal lung tissues). Furthermore, majority of these 22 variants have been identified as expression quantitative trait loci (eQTLs) of a number of genes in various tissue types including normal lung tissues. The functional potential of ten non-synonymous SNPs were further predicted using PolyPhen-2^26^. The variant rs351855 may result in a probably damaging effect on FGFR4 function. The other non-synonymous SNPs were predicted to be “benign”.

**Table 4:** Functional annotation of 22 variants associated with lung cancer risk with strong evidence using HaploReg v4.1 and PolyPhen-2.

variant	Gene (or near gene)^ǁ^	HaploReg v4.1^∫^											PolyPhen-2^§^	
		GERP conserved	Promoter histone marks	Enhancer histone marks	DNAse	Proteins bound	Motifs changed	NHGRI/EBI GWAS hits	GRASP QTL hits	Selected eQTL hits	RefSeq genes	dbSNP functional annotation	predicted consequence on protein function	PolyPhenscore^¶^
rs1760944	*APEX1*		24 tissues*	14 tissues*	52 tissues*	11 bound proteins			2 hits	69 hits*	*OSGEP*	5′UTR		
rs6495309	*CHRNA3*		THYM	4 tissues	THYM		7 altered		2 hits	10 hits	1.4 kb 3′ of *CHRNB4*			
rs1126579	*CXCR2*		BLD	BLD			9 altered			69 hits*	*CXCR2*	3′UTR		
rs6413432	*CYP2E1*		4 tissues	IPSC			8 altered			1 hit	*CYP2E1*	intronic		
rs931794	*HYKK*			ESDR, SKIN, BRN			4 altered		1 hit	26 hits	*AGPHD1*	intronic		
rs664677	*ATM*			BLD, FAT, LIV			4 altered			24 hits	*ATM*	intronic		
rs402710	*CLPTM1L*		4 tissues	7 tissues			5 altered	1 hit^†^	1 hit	1 hit	*CLPTM1L*	intronic		
rs4646903	*CYP1A1*		SKIN	LNG						8 hits	241 bp 3′ of *CYP1A1*			
rs2240308	*AXIN2*		22 tissues*	23 tissues*	6 tissues		Smad3		2 hits	3 hits	*AXIN2*	missense	benign	0
rs662	*PON1*	conserved	LNG*	10 tissues*					2 hits	2 hits	*PON1*	missense	benign	0
rs462779	*REV3L*	conserved					BRCA1, Nkx3		1 hit	2 hits	*REV3L*	missense	benign	0
rs1130409	*APEX1*		20 tissues*	23 tissues*	4 tissues		ZNF263			8 hits	*APEX1*	missense	benign	0
rs16969968	*CHRNA5*									32 hits*	*CHRNA5*	missense	benign	0.045
rs13181	*ERCC2*	conserved	ESDR, SKIN, SPLN	4 tissues	4 tissues			1 hit^‡^	3 hits	18 hits*	*ERCC2*	missense	benign	0
rs4880	*SOD2*		24 tissues*	19 tissues*	46 tissues*	CMYC,POL2, SIN3AK20	CHD2		1 hit	29 hits*	*SOD2*	missense	benign	0
rs351855	*FGFR4*	conserved	4 tissues	15 tissues*	LIV		5 altered		2 hits	15 hits	*FGFR4*	missense	probably damaging	0.998
rs1052133	*OGG1*	conserved	BLD, SKIN	10 tissues*			GATA			5 hits*	*OGG1*	missense	benign	0.121
rs1042522	*TP53*		5 tissues	9 tissues*	LNG*		9 altered		1 hit	1 hit	*TP53*	missense	benign	0.083
rs2736098	*TERT*		10 tissues*	16 tissues*	BLD		9 altered	1 hit		1 hit*	*TERT*	synonymous		
rs11615	*ERCC1*	conserved	9 tissues	21 tissues*	4 tissues	ZNF263	EBF,Mtf1		2 hits	5 hits	*ERCC1*	synonymous		
rs2910164	*MIR146A*	conserved	4 tissues	8 tissues							*MIR146A*			
rs11614913	*MIR196A2*	conserved	13 tissues	16 tissues*	8 tissues*		HMG-IY		1 hit	6 hits	*MIR196A2*			

^ǁ^The gene name for the SNP, locating in a respective gene, was based on the annotation of dbSNP database (https://www.ncbi.nlm.nih.gov/snp/). The near gene name for a SNP that didn’t map into a gene region but its location nearby a gene based on the annotation of dbSNP database, and we also used this nearby gene name for the SNP in our study. ^∫^HaploReg v4.1: a Web server for annotation of transcription regulation for genetic variants (http://archive.broadinstitute.org/mammals/haploreg/haploreg.php). ^§^PolyPhen-2: a Web server for annotation of potential effects on protein structure and function for non-synonymous SNPs (http://genetics.bwh.harvard.edu/pph2/). ^¶^The PolyPhen-2 reported a score that the calculated naive Bayes posterior probability of a given mutation being damaging ranging from 0 to 1, which was also classified as benign [0, 0.15], possibly damaging (0.15, 0.85], and probably damaging (0.85, 1], respectively. *Including regulatory evidence in lung cancer cell lines/tissues or normal lung cell lines/tissues. ^†^GWAS for the trait of lung cancer with a P-value at 4.0 × 10^−6^. ^‡^GWAS for the trait of lung cancer with a P-value at 9.0 × 10^−7^.

### Non-significant associations

Non-significant associations for 150 variants within 98 genes were found under any genetic model (allelic, dominant, or recessive model) in both main and subgroup meta-analyses (Supplementary Table S9). Among these 150 variants, credibility of cumulative epidemiological evidence were identified as strong, moderate, or weak for seven (*ERCC1* rs16979802, *ERCC1* rs2298881, *ERCC1 r*s735482, *POLI* rs3730668, *PPARG* rs1801282, *PTGS2* rs20417, and *TNF* rs1799724), four (*ERCC2* rs1799793, *TYMS* 28-bp tandem repeat, *XPC* rs2228000, and *XRCC3* rs861539), and 139 variants, respectively (Supplementary Table S9).

## Discussion

To the best of our knowledge, this systematic meta-analysis is the largest and most comprehensive assessment of currently available literatures on candidate-gene association studies in lung cancer. This study examined associations between genetic variants and lung cancer risk using data from 1,018 candidate-gene association studies including 2,910 genetic variants. The meta-analyses and evidence evaluations allowed us to identify 22 genetic variants in 21 genes with strong evidence of associations with lung cancer risk. For these variants, additional genomic annotation information provided evidence of putative regulatory functions, including regulatory histone modification marks, DNase I hypersensitivity, motif changed, and transcription factor binding in multiple cell types including lung tissue.

Variants in non-coding region associated with lung cancer risk may have their effects through transcription, mRNA stability, protein structure/function, or binding sites of miRNA^27^. For example, the variant rs1760944 (−656T > G) at the 5′-promoter region of *APEX1* ^28^ was shown as a significant variant (T vs. C allele, OR 1.16, 95%CI 1.08–1.25) with strong cumulative evidence. This variant is predicted to influence promoter histone marks in 24 tissues including lung and lung cancer cell lines. Previous *in vitro* promoter assay has detected that the rs1760944 T allele significantly lowered promoter activity than that of the G allele, which indicated the variant allele (T) may be associated with a low transcriptional activity of the *APEX1* in lung cancer cells^28^. The variant rs6495309 in *CHRNA3/B4* intergenic region^12^ showed strong evidence of association with lung cancer susceptibility in our meta-analysis. This finding was consistent with the results from a previous meta-analysis performed in Chinese population^29^, and a recent meta-analysis performed on the basis of GWASs of lung cancer^15^. Additional subgroup analysis of Asians in our study also showed the risk effect for the rs6495309 C allele. This SNP overlaps with promoter histone marks and alters regulatory motif. Functional study also demonstrated that the rs6495309 C allele significantly increased the CHRNA3 expression through altering the ability of CHRNA3 promoter binding to the transcriptional factor Oct-1^12^. A common genetic variation rs1126579 (C > T) located in the 3′UTR of the *CXCR2* (*IL8RB*) was found to be associated with a reduced risk of lung cancer with strong evidence. The HaploReg tool identified that rs1126579 was an eQTL for a number of genes including *CXCR2*. Previous studies also reported that CXCR2 was down regulated in lung cancer tissue and might play a suppressive role in lung cancer via the p53-dependent senescence^30, 31^. Functional data indicated that the rs1126579 variant can disrupt the binding site of miR-516a-3p and further increase the expression of CXCR2^30^, which may also explain why rs1126579 showed a protective effect on the risk of lung cancer.

Variants falling within coding regions, especially non-synonymous SNPs, could have some effects on protein structure, function, or expression level, which may explain its association with the susceptibility of disease^32^. For example, the non-synonymous *CHRNA5* rs16969968 (Asp398Asn) causes an amino acid substitution at codon 398 of the CHRNA5 protein. And the aspartic acid (Asp398) is located at the central part of the second intracellular loop in the structure of CHRNA5 protein, and was reported highly conserved across multiple species^10^. The rs1042522 (Arg72Pro) is a common functional SNP in the exon 4 of *TP53*, which encodes an important tumor suppressor protein. *TP53* gene is often mutated in NSCLC tumors, an early event in development of lung cancer^33^. Further functional data showed that the 72Pro allele carriers of lung cancer patients may have a low frequency of the *TP53* mutations in tumors^34^. The rs351855 (Gly388Arg) influences the transmembrane domain of the FGFR4 protein^35^. This SNP resides in a conserved region and causes a possibly damaging effect on protein function of FGFR4 predicted by PolyPhen. Also, rs4800 (Ala16Val) is a non-synonymous SNP in *SOD2*. This SNP with valine variation can reduce enzyme activity^36^ and further increase oxidative stress. Rs2736098 is a synonymous SNP (Asn305Asn) in exon 2 of the *TERT* gene, which is a well known oncogene and encodes the catalytic subunit of the telomerase^37^. This SNP may have association with telomere length^38^. Although it does not change protein amino acid, this SNP is located within the gene regulatory elements and may alter transcription factor binding.

In addition, we found two SNPs with strong evidence of associations with lung cancer risk are located in miRNA gene coding regions, rs2910164 (C > G) in the seed of miR-146a-3p encoded by *MIR146A* and rs11614913 (C > T) in the mature sequence of miR-196a-3p encoded by *MIR196A2* ^39^. Both SNPs showed significant miRNA expression differences between their alleles^39, 40^ and could affect the stability of secondary hairpin structure^39^. Study also showed that rs2910164 can influence the interaction between miR-146a-3p and its potential target genes, and rs11614913 can increase the affinity of miR-196a-3p for *TP53* ^39^.

Our subgroup analyses also provided additional important details of genetic associations in specific groups. The results of subgroup meta-analyses by ethnicity supported the well-known cognition of “racial” differences in genetic effects for complex diseases including lung cancer^41^ and indicated that some variants (eg, *APEX1* rs1130409, *CHRNA5* rs16969968, *ERCC2* rs13181, *SOD2* rs4880, and *CYP2E1* rs6413432) with strong evidence may be ethnic-specifically associated with lung cancer risk. Previous studies had demonstrated the existence of different genetic background in different histological subtypes of lung cancer^15, 42^. When cases were stratified according to histological types, the associations between several variants (eg, *CYP2E1* rs6413432, *OGG1* rs1052133, *TP53* rs1042522, and *CYP1A1* rs4646903) and specific subtypes of lung cancer were of strong evidence. A growing number of studies demonstrates interactions between genetic variants and smoking^43, 44^. Our subgroup analysis also found that some variants showed significant associations with lung cancer risk in smokers but not in non-smokers, for example *CYP1A1* rs4646903 and *GSTP1* rs1695.

As the purpose of meta-analysis is not only to reveal genetic variants significantly associated with lung cancer risk, but also to identify the variants with non-significant associations. Our study revealed that 150 variants in 98 genes had non-significant associations with lung cancer risk. However, most of these variants had weak cumulative epidemiological evidence due to the presence of insufficient statistical power (119/150) and/or strong between-study heterogeneity (73/150), and only 11(7.3%) variants had strong or moderate cumulative evidence. Our results provided important clues to further assess the main effects of these variants.

Despite a comprehensive and systematic approach was applied to the synopsis of genetic association studies in lung cancer, several limitations should be considered when interpreting our results. First, although available studies were searched widely and eligible studies were selected strictly according to the inclusion and exclusion criteria, it is possible that some studies might have been overlooked. Our studies didn’t include research published in the form of abstracts or in language other than English. However, for most abstracts, we also searched and included relevant studies published with whole text and reported by the same research groups. Publication biases were not identified in most meta-analyses with significant association results. Also, the proportion of studies published in language other than English is small therefore it should not have significant influence on the main results. Second, the percentage of meta-analyses with high heterogeneity (*I* ^*2*^ > 50) was more than 40% for all meta-analyses with a significant result. Although subgroup analyses stratified by ethnicity, histology, and smoking status were performed to address the heterogeneity, other sources of heterogeneity could exist and are difficult to address because of limited available data. Third, although we tried to explore the consistency and difference in genetic associations between some variants and lung cancer risk across different ethnic groups, meta-analyses stratified by ethnicity were performed only for Caucasian and Asian populations. Since very few enrolled original studies were carried out in other descent populations (e.g. African descent), the available data were not sufficient to perform subgroup meta-analyses in other descent populations. Additional association studies are needed to establish in populations of other ethnic descent for these reported variants. Finally, although we conducted systematic evaluations of cumulative epidemiological evidence for variants associated with lung cancer risk, biases cannot be completely excluded in this study.

In summary, our comprehensive research synopsis and meta-analysis identified 22 variants in 21 genes had strong cumulative epidemiological evidence of significant associations with lung cancer risk. While, among variants without significant associations with lung cancer, seven had strong evidence. Our findings provided useful data and important references for the future studies to evaluate the genetic role in the field of lung cancer. The identification of genetic variants with robust association to lung cancer may help us to get more precise estimate of population risk stratification and potential target population for primary prevention.

## Methods

### Selection criteria and search strategies

All methods were in accordance with the PRISMA statement, the HuGE Review Handbook (version1.0) guiding genetic reviews specifically, and Meta-analysis Of Observational Studies in Epidemiology (MOOSE) guidelines^20–22, 45^.

A study for inclusion had to meet the following four criteria: (1) it evaluated the association between a genetic polymorphism and lung cancer risk using a case-control, cohort, or a cross-sectional design in human; (2) lung cancer cases were diagnosed by pathological and/or histological examination; (3) it was published in a peer-reviewed scientific journal or online in English; (4) it provided sufficient information of genotype and/or allelic distributions for both cases and controls. We excluded studies with a family-based design and loci with genome-wide significant (P < 5 × 10^−8^) identified by GWAS since they have been replicated by many studies.

To identify all published association studies potentially eligible for inclusion in our meta-analysis, we performed a comprehensive literature search (Fig. 1). Two electronic databases (PubMed and EMBASE) were queried with the terms “lung cancer (as well as synonyms of lung cancer) AND associate*” on or before December 31, 2014. This search yielded 41,457 publications, and then screened respectively for eligibility using the title, abstract, or full-paper, as necessary. For publications between December 31, 2014 and November 1, 2015, we searched databases (PubMed and EMBASE) monthly using the previous search terms and the additional terms of “lung cancer AND [gene/loci names identified in enrolled publications]”. This second search identified 4,453 additional potential publications. Furthermore, we screened for bibliographies in reviews, published meta-analyses, and cited articles from the retrieved publications. Taken together, a total of 1,018 eligible papers were finally selected and their full-text versions were carefully reviewed for further analyses (Fig. 1).

### Data management and abstraction

When multiple publications used the same or overlapping data sets, we kept the data with the largest population or most recent ones as recommended by Little *et al*.^46^. Forty three publications with redundant information were then excluded. Using standard data extraction forms, we extracted the detailed publication information, study design, characteristics of participants, gene and variant information. Subgroup information (ancestry, smoking status, or histological types) were also separately extracted from each study whenever possible. Ancestry was divided into four general groups (African, Asian, Caucasian, and other/mixed) based on ancestry of at least 80% of the subjects^41^. If no details of ethnicity were reported, the determination was made based on the general population of the country or region where the study was done^41^. When a publication reported data from multi-racial groups, data for each population were extracted and analyzed separately if possible.

To avoid the variant nomenclature confusion from different articles, we used the most current gene names and uniform identifiers (“rs” number) of variants in a public single nucleotide polymorphism (SNP) database (dbSNP, http://www.ncbi.nlm.nih.gov/projects/SNP/index.html), to designate the reported variants. For articles with “rs” number, we used as it was; for these without we used bioinformatics tools such as NCBI Blast (http://www.ncbi.nlm.nih.gov/BLAST/) and UCSC In-Silico PCR (http://genome.ucsc.edu/cgi-bin/hgPcr) to find “rs” number for the reported variant; for the remaining without any “rs” number, we used the common nomenclature (eg, *MPG* Arg59Cys according to amino acid substitution and *GSTM1* present/null according to phenotype change) in the original articles.

### Statistical analysis

All statistical analyses were performed using Stata software (version 12.0, StataCorp 2011, TX, USA), except where indicated otherwise. All tests were two-sided and considered statistically significant when p value was at 0.05 or lower, unless otherwise stated.

All variants from at least three data sources were selected for meta-analysis^18^. Association between a variant and lung cancer risk was assessed by study-specific crude odds ratios (ORs) and 95% confidence intervals (CIs) using a DerSimonian and Laird random-effects model^47^. The initial main meta-analyses assessed the variant effect using an allelic genetic model (minor allele vs. major allele) without stratification. For the variation not in the form of single nucleotide substitution, a conventional comparison from the publications was used to assess the effects (eg, *CYP2A6* [*4 vs. non*4], *MMP3* rs3025058 [5A vs. 6A], and *GSTM1* [null vs. present]). When average minor allele frequency (MAF) were greater than 50%, a rare occasion where major and minor alleles are flipped in different ethnic populations, we designated the minor allele from Caucasian population in all analyses. For the variant with sufficient genotype distribution data, we performed additional analyses based on dominant and recessive genetic models.

Subgroup meta-analyses were also performed by ethnicity (Caucasian and Asian), histological types (SCLC, NSCLC, AD, and SCC), and smoking status (smoking and nonsmoking), if sufficient data were available.

Between-study heterogeneity was assessed by calculating the Cochran Q statistic, with a p value less than 0.10 being the significant threshold^48^. We also used *I* ^*2*^ heterogeneity metric to assess the heterogeneity^49^. Generally, *I* ^*2*^ < 25%, 25%-50% and > 50% showed mild, moderate, and strong heterogeneity, respectively.

The publication bias of studies was evaluated by funnel plot analysis (logOR against standard error) and Begg’s test^50^. Potential small study effect (a trend for smaller study to show larger effect) was checked by the modified Egger’s test, which can lower the type I and type II error rates compared to the original Egger’s test^51^. We also conducted an excess significance test to examine whether there was a relative excess of formally significant findings in studies due to potential sources of bias, such as selective analyses, selective outcome reporting, or fabricated data^52^.

For all variants that showed a significant association with lung cancer risk, we performed a sensitivity analysis to examine whether the significant summary ORs were robust after excluding the first published or first positive report, or excluding studies with controls violating Hardy-Weinberg equilibrium [HWE]. We used a Fisher’s exact/chi-square to assess the HWE among controls in each dataset.

### Assessment of cumulative evidence

For each nominally significant results from the meta-analyses, Venice criteria was used to assess the credibility of cumulative epidemiological evidence^21^. Venice criteria is a semi-quantitative index which assigns three aspects for the amount of evidence, extent of replication, and protection from bias, and finally generates a composite assessment of “strong”, “moderate”, or “weak” epidemiological credibility for an association with lung cancer risk^21^. For the three aspects (the amount of evidence, extent of replication, and protection from bias) of Venice criteria, each aspect was assigned three levels (A, B, or C)^21^. Briefly, amount of evidence, depending on total sample size of the smallest genetic group among cases and controls in each meta-analysis, was graded as A (sample size >1000), B (sample size between 100 and 1000), or C (sample size <100). For very rare variant with frequency less than 0.5%, the amount of evidence was not assessed considering an A grade was unlikely to obtain^18^. The extent of replication, depending on between-study heterogeneity, was graded as A (*I* ^*2*^ < 25%), B (*I* ^*2*^ between 25% and 50%), or C (*I* ^*2*^ > 50%). The protection from bias, considering various potential sources of bias in meta-analysis, was graded as A when there was no demonstrable bias and the bias would unlikely invalidate the association, B when there was insufficient information for identifying evidence (eg, missing information for evaluating HWE among controls in an individual study) although there was no obvious bias, and C when the bias was evident and/or was likely to explain the presence of association. More specifically, C grade was assigned if the meta-analysis had any of the following potential sources of bias: (1) the magnitude of the association was low (eg, OR <1.15 for risk effect, OR >0.87 for protective effect) with the exception of a highly consistent OR across studies enrolled in meta-analysis; (2) the sensitivity analysis indicated that the significant summary OR can be substantially changed; (3) the potential small study effect was present according to the modified Egger’s test (*p*-value < 0.10); (4) an excess of significant findings was possible (excess significance test, *p*-value < 0.10); (5) there was a potential publication bias (Begg’s test, *p*-value < 0.10). With the grades from three aspects, the credibility of cumulative epidemiological evidence was categorized as strong (all three aspect grades were A), moderate (any grade was B, but not C), or weak (any grade was C).

Additionally, for the non-significant associations revealed by all meta-analyses, we also evaluated the credibility of cumulative epidemiological evidence based on three aspects: the degree of heterogeneity across studies, potential bias assessment, and statistical power. The statistical power was calculated by using SNP tools^53^. The credibility of cumulative epidemiological evidence of non-significant association was categorized as strong (if there was no or mild [*I* ^*2*^ < 25%] heterogeneity across studies, no demonstrable bias, and sufficient statistical power [power >90%]), weak (heterogeneity *I* ^*2*^ > 50%, or any potential bias detected, or low statistical power [power <80%]), or moderate (for other cases).

### Data Availability

All data generated or analysed during this study are included in this article and its Supplementary Information file.

## Electronic supplementary material

Supplementary information file
