# Supplementary material for: Genetic predisposition to lung cancer: comprehensive literature integration, meta-analysis, and multiple evidence assessment of candidate-gene association studies
Source: Sci Rep. 2017 Aug 21;7:8371. doi: 10.1038/s41598-017-07737-0 (PMC5567126; doi:10.1038/s41598-017-07737-0)
Supplement: Supplementary file 1 — Supplementary information file [file 41598_2017_7737_MOESM1_ESM.pdf]

Supplementary Information to:

Genetic predisposition to lung cancer: comprehensive literature integration, meta-analysis, and multiple evidence assessment of candidate-gene association studies

Junjun Wang M.D.[1, 2 #], Qingyun Liu M.D.[1 #], Shuai Yuan M.D.[1 #], Weijia Xie Ph.D. [1 #], Yuan Liu M.D. [1], Ying Xiang M.P.H. [1, 2], Na Wu M.D. [1, 2], Long Wu M.D. [1, 2], Xiangyu Ma Ph.D. [1, 2], Tongjian Cai Ph.D. [1, 2], Yao Zhang Ph.D. [1, 2], Zhifu Sun M.D.[3], Yafei Li Ph.D. [1, 2*]

[1] Department of Epidemiology, College of Preventive Medicine, Third Military Medical University, Chongqing, People's Republic of China

[2] Center for Clinical Epidemiology and Evidence-Based Medicine, Third Military Medical University, Chongqing, People's Republic of China

[3] Health Sciences Research, Mayo Clinic College of Medicine, Rochester, Minnesota, USA

[#] These authors contributed equally to this work.

[*]**Corresponding author:** Yafei Li, Department of Epidemiology, College of Preventive Medicine, Third Military Medical University, NO.30 Gaotanyan Street, Chongqing 400038, People's Republic of China. E-mail: liyafei2008@hotmail.com.
Telephone: +86 23 68752293. Fax: +86 23 68752293.

Contents

Supplementary Results

Information of variants involved in meta-analyses
Supplementary results of main meta-analyses
Supplementary results of subgroup meta-analyses

Supplementary Figures

Figure S1. Distribution by chromosome of 246 genetic variants available for meta-analyses.
Figure S2. Venn diagram of the distributions of genetic variants with significant associations with lung cancer risk in the main meta-analyses and subgroup meta-analyses by ethnicity
Figure S3. Venn diagram of the distributions of genetic variants with significant associations with specific histological types of lung cancer.
Figure S4. Venn diagram of the distributions of genetic variants with significant associations with lung adenocarcinoma and squamous cell carcinoma.
Figure S5. Venn diagram of the distributions of genetic variants with significant associations in subgroup meta-analyses of smoker and non-smoker.
Figure S6. Forest plots of main or subgroup meta-analyses for variants showed significant associations to lung cancer susceptibility with strong cumulative evidence.
Figure S7. Begg's funnel plots of main or subgroup meta-analyses for variants showed significant associations to lung cancer susceptibility with strong cumulative evidence.

Supplementary Tables

Table S1. Genetic associations with lung cancer risk for all 246 variants analyzed in main meta-analyses under different genetic models
Table S2. Credibility assessment for variants with significant associations with lung cancer risk in meta-analyses for all available data
Table S3. Subgroup meta-analyses stratified by ethnicity for genetic variants with available data
Table S4. Credibility assessment for variants with significant associations with lung cancer risk in subgroup meta-analyses by ethnicity
Table S5. Subgroup meta-analyses stratified by different histological types of lung cancer for variants with sufficient data under different genetic models
Table S6. Credibility assessment for variants with significant associations with lung cancer risk in subgroup meta-analyses by histological types
Table S7. Subgroup meta-analyses stratified by smoking status for variants with sufficient data under different genetic models
Table S8. Credibility assessment for variants with significant associations with lung cancer risk in subgroup meta-analyses by smoking status
Table S9. Credibility assessment for variants with non-significant associations with lung cancer risk in meta-analyses for all available data

Supplementary Literatures

Lists of 1,018 eligible studies enrolled in this comprehensive research synopsis

Supplementary Results

Information of variants involved in meta-analyses

Our systematic literature search identified 1,018 eligible publications reporting 2,910 genetic variants in 754 unique genes or loci. Main meta-analyses were performed for 246 genetic variants (in 138 genes or loci) with sufficient data (**Supplementary Table S1**). All of these variants are located on autosomes, and chromosome 19 has the highest number of variants (n=47, 19%) (**Supplementary Fig. S1**). According to their population frequency, 235 of the 246 variants are common variants and 11 are rare. These variants were further classified by their potential functional effects: (1) synonymous single nucleotide polymorphisms (sSNPs, n=15); (2) non-synonymous SNPs (nsSNPs, n=90); (3) frameshift polymorphism (n=1); (4) SNPs in 3′ or 5′ untranslated regions (UTRs; n=40); (5) intronic SNPs (n=46); (6) intergenic SNPs (n=38); (7) SNP in a splice site (n=1); (8) one deletion/insertion variant; (9) variants with microsatellite or short tandem repeat (STR) variation (n=4); (10) variants with multi-nucleotide variation (n=2); (11) variants with a specific change in phenotype (n=4); (12) variants with copy number variation (CNV) (n=2); (13) SNPs in microRNA (n=2) (**Supplementary Table S1**).

Supplementary results of main meta-analyses

Strength of association between each genetic variant and lung cancer risk

The strength of association between each genetic variant and lung cancer as measured by ORs had the mean of 1.36 (range 1.08-2.55) for putative "risk" variants and 0.78 (range 0.55-0.90) for putative "protective" variants. Four variants (*HIF1A* rs11549467, *HRAS1* a VNTR [variable number of tandem repeat] variation (loss of

heterozygosity in the 15p15.5 region), *UGT1A6* rs6759892, and *WWOX* CNV-67048) showed strong associations (ORs > 2) with risk of lung cancer. Eight variants (*MAPKAPK2* CNV-30450, *SFTPB* variation [a large gene variation within intron 4], *TP53* rs12951053, *BCL2* rs2279115, *CYP2A6* rs1801272 and rs5031016, *ELANE* rs351107 [-903T>G, Rep_a], and *MMP2* rs243865) showed moderate genetic associations ("risk" ORs range 1.5-2.0, or "protective" ORs range 0.50-0.67) with lung cancer. The remaining 44 significant variants showed low associations (ORs range 0.67-1.50) with lung cancer, of note, four variants of them (*CHRNA3* rs6495309, *CYP2E1* rs6413432, *GSTM1* phenotype, and *HYKK* rs931794) showed highly significant associations ($P < 10^{-7}$) with lung cancer risk (**Table 1 and Supplementary Table S2**).

Assessment of heterogeneity across studies

In the 246 main meta-analyses, 86 (34.96%) had little or no heterogeneity ($I^2 <$ 25%) and 122 (49.59%) had high heterogeneity ($I^2 >$ 50%) (**Supplementary Table S1**). And among the 56 main meta-analyses with statistically significant summary ORs, 24 (42.86%) had little or no heterogeneity and 22 (39.29%) had high heterogeneity (**Table 1; Supplementary Table S2**).

Examinations for potential bias in studies enrolled in meta-analyses

Of the 246 main meta-analyses, 21(8.54%) had evidence of substantial publication bias on the basis of the Begg's test ($P < 0.10$), 11 of which had statistically significant pooled ORs ($P \leq 0.05$) in main meta-analyses (**Supplementary Table S1**). Furthermore, a modified Egger's test was used to evaluate whether small studies provide different results from larger studies for all

main meta-analyses, and 30 (12.20%) had a significant test ($P < 0.10$) including 11 with statistically significant pooled ORs (*BCL2* rs2279115, *CHRNA3* rs938682, *CYP1A1* rs1048943, *ELANE* rs7254054 (-741G>A, Rep_b), *ERCC1* rs11615, *FGFR4* rs351855, *GSTM1* phenotype, *GSTP1* rs1695, *IL10* rs1800896, *MIR146A* rs2910164, and *MMP2* rs243865). To examine whether there were relative excess of formally significant findings in studies due to potential sources of bias, such as selective analyses, selective outcome reporting, and fabricated data, we performed an excess significance test. A total of 40 meta-analyses had evidence of inclusion of many individual studies reporting excess significant results (**Supplementary Table S1**). Among these 40 meta-analyses, seven had statistically significant pooled ORs with small effect sizes (mean pooled ORs 1.18, range 1.08-1.20). When the excess of significance was examined in aggregate across all studies enrolled in all main meta-analyses, there was significant evidence of existing excess significance ($P < 10^{-8}$), indicating potential bias existed in these published studies. A sensitivity analysis was performed to further assess the robustness of these results (**Supplementary Table S2**). Fourteen meta-analyses lost significance after removing the initial study. Of 17 meta-analyses with statistically significant pooled ORs, three became non-significant after removal of studies showing deviations from Hardy-Weinberg equilibrium (HWE).

Supplementary results of subgroup meta-analyses

Subgroup meta-analyses by ethnicity

Subgroup meta-analyses were conducted in Caucasian and Asian population under each of the three genetic models (allelic, dominant, or recessive model),

respectively (**Table 3, Supplementary Table S3**). Among those 246 genetic variants investigated in main meta-analyses, there were 86 variants with sufficient available data for subgroup analyses in Caucasian and 71 variants in Asian population (**Supplementary Table S3**). In addition, 29 variants studied only in one specific ethnic population (17 variants in Caucasians and 12 in Asians) have been evaluated previously in the main meta-analyses (**Supplementary Table S1**). Thus, a total of 103 variants were evaluated in Caucasian population and 83 variants in Asian population.

In Caucasian population, 19 (22.1%) variants significantly associated with lung cancer risk. The strength of association between each variant and lung cancer as measured by ORs had the mean of 1.39 (range 1.07-1.99) for putative "risk" variants and 0.73 (range 0.59-0.92) for putative "protective" variants. Of the 19 significant variants, one (*HRAS1* a VNTR variation) showed strong genetic association (OR > 2), five (*CYP1A2* rs762551, *SFTPB* a combination variation [combined deletion and insertion], *CYP2A6* rs1801272, *CYP2E1* rs2031920, and *ELANE* rs351107 [-903T>G, Rep_a]) showed moderate genetic associations ("risk" ORs range 1.5-2.0, or "protective" ORs range 0.50-0.67), and the others showed low associations (ORs range 0.67-1.50) with lung cancer, including one variant (*CHRNA5* rs16969968) showed highly significant association with lung cancer risk with *P*-value at 2.03×10^{-21} (**Supplementary Table S4**). Of the 19 significant variants, 13 were identified from meta-analyses with little or no heterogeneity (I^2 < 25%), four (*CYP1A2* rs762551, *ELANE* rs351107, *ELANE* rs7254054, and *XRCC1* rs1799782) were identified from meta-analyses with moderate heterogeneity (25% ≤ I^2 ≤ 50%), and two (*CYP1A1* rs1048943 and *HRAS1* a VNTR variation) were identified from meta-analyses with high heterogeneity. According to the examinations of potential bias (the Begg’s test,

modified Egger's test, and the excess significance test), significant associations for four variants (*CYP1A1* rs4646903, *SFTPB* variation, *ELANE* rs7254054, and *CYP1A1* rs1048943) had evidence of potential bias (publication bias, small study effects, or excess significance bias) (**Supplementary Table S4**). When the initial study was excluded, the association for four variants (*ELANE* rs351107 and rs7254054, *ERCC2* rs238406, and *HRAS1* a VNTR variation) was no longer statistical significant. When studies with HWE-violating controls were excluded, the association for two variants (*ERCC2* rs238406 and *MTHFR* rs1801131) was no longer statistical significant.

In Asian population, 26 (31.3%) variants showed significant associations with lung cancer. The strength of association between each genetic variant and lung cancer as measured by ORs had the mean of 1.27 (range 1.08-1.50) for putative "risk" variants and 0.76 (range 0.64-0.91) for putative "protective" variants. Of the 26 significant variants, one (*WWOX* CNV-67048) showed strong genetic association (OR > 2), three (*MAPKAPK2* CNV-30450, *CYP2A6* non*4/*4, and *VEGFA* rs833061) showed moderate genetic associations ("risk" ORs range 1.5-2.0, or "protective" ORs range 0.50-0.67), and the other 22 variants showed low genetic associations (ORs range 0.67-1.50) with lung cancer (**Supplementary Table S4**). Of the 26 significant variants, 14 were identified from meta-analyses with little or no heterogeneity (I^2 < 25%), four (*ATM* rs189037, *CHRNA3* rs6495309, *GSTP1* rs1695, and *GSTT1* present/null) were identified from meta-analyses with moderate heterogeneity (25% ≤ I^2 ≤ 50%), and eight (*CYP1A1* rs4646903, *CYP1A1* rs1048943, *GSTM1* present/null, *IL10* rs1800872, *MAPKAPK2* CNV-30450, *MDM2* rs2279744, *XRCC1* rs25487, and *XRCC1* rs3213245) were identified from meta-analyses with high heterogeneity. Among the 26 significant variants, two (*CYP1A1* rs4646903 and *MDM2* rs2279744)

had associations showing evidence of potential bias of small study effects and three (*CYP1A*1 rs1048943, *GSTM1* present/null, and *XRCC1* rs25487) had associations showing evidence of inclusion of studies with excess of significant findings and statistically significant pooled ORs with small effect sizes (mean pooled ORs 1.23, range 1.05-1.43) (**Supplementary Table S4**). When initial study was excluded, the association for seven variants (*AGER* rs1800624, *CYP1A1* rs4646903, *FASLG* rs763110, *IL10* rs1800872, *VEGFA* rs833061, *XRCC1* rs25487 and rs3213245) was no longer significant. When studies with HWE-violating controls were excluded, the association for one variant (*XRCC1* rs25487) was no longer significant.

Subgroup meta-analyses by histological types of lung cancer

Subgroup meta-analyses stratified by histological types of lung cancer were performed under each of the three genetic models (allelic, dominant, or recessive model) (**Supplementary Table S5**). There was sufficient data to evaluate 22 variants in small cell lung cancer [SCLC], 92 in non-small cell lung cancer [NSCLC], 48 in adenocarcinoma [AD], and 46 in squamous cell carcinoma [SCC] group, respectively.

In the SCLC subgroup, statistical significant associations were found for five variants, where three (*CHRNA5* rs16969968, *CYP1A1* rs4646903, and *NQO1* rs1800566) and two (*GSTM1* present/null and *XPC* rs2228001) showed moderate genetic associations (ORs range 1.5-2.0) and low genetic associations (ORs range 0.67-1.50) with lung cancer risk, respectively (**Supplementary Table S6**). Of these significant variants, two (*CYP1A1* rs4646903 and *XPC* rs2228001) were identified from meta-analyses with little or no heterogeneity (I^2 < 25%), one (*GSTM1* present/null) was identified from meta-analysis with moderate heterogeneity (25% ≤

$I^2 \leq 50\%$), and two (*CHRNA5* rs16969968 and *NQO1* rs1800566) were identified from meta-analyses with high heterogeneity. Based on the examinations of potential bias, one variant (*CHRNA5* rs16969968) had association with evidence of potential bias of small study effects. Based on the sensitivity analyses, the association for two variants (*NQO1* rs1800566 and *XPC* rs2228001) was no longer significant after removing the initial study.

In the NSCLC subgroup, 25 (27.2%) variants showed significant associations with lung cancer. The strength of these associations as measured by ORs had the mean of 1.37 (range 1.11-1.73) for putative "risk" variants and 0.77 (range 0.65-0.91) for putative "protective" variants. Of the 25 significant variants, five (*FAS* rs2234767, *IL17A* rs2275913, *TNF* rs1800629, *XRCC1* rs2256507, and *MMP2* rs243865) showed moderate genetic associations ("risk" ORs range 1.5-2.0, or "protective" ORs range 0.50-0.67) and 25 variants showed low genetic associations (ORs range 0.67-1.50) with lung cancer where two variants (*CHRNA5* rs16969968 and *XRCC1* rs3213245) showed highly significant associations ($P < 10^{-7}$) with lung cancer risk (**Supplementary Table S6**). Of the 25 significant variants, 14 were identified from meta-analyses with little or no heterogeneity ($I^2 < 25\%$), four (*ERCC2* rs13181, *IL17A* rs2275913, *OGG1* rs1052133, and *XRCC1* rs1001581) were identified from meta-analyses with moderate heterogeneity ($25\% \leq I^2 \leq 50\%$), and seven (*GSTM1* present/null, *IL1B* rs1143627, *IL6* rs1800796, *MMP2* rs243865, *MPO* rs2333227, *SOD2* rs4880, and *TNF* rs1800629) were identified from meta-analyses with high heterogeneity. Based on the further examinations of potential bias, evidence of publication bias, potential bias of small study effects, inclusion of studies with excess significant findings, was found for one variant (*IL1B* rs1143627), four variants (*IL1B*

rs1143627, *IL6* rs1800796, *MMP2* rs243865, and *SOD2* rs4880), and one variant (*OGG1* rs1052133) with association of small effect size (OR 1.16, 95%CI 1.02-1.32), respectively (**Supplementary Table S6**). When initial study was excluded, association for five variants (*FAS* rs2234767, *IL1B* rs1143627, *MMP2* rs243865, *TNF* rs1800629, and *XRCC1* rs1001581) was no longer significant. When sensitivity analyses removing studies with HWE-violating controls, two variants (*ERCC2* rs13181 and *IL1B* rs1143627) became non-significant.

In the AD subgroup, 15 (31.3%) variants significantly associated with lung cancer risk. The strength of associations between each genetic variant and lung cancer as measured by ORs had the mean of 1.37 (range 1.11-1.70) for putative "risk" variants and 0.81 (range 0.71-0.93) for putative "protective" variants. Of the 15 significant variants, three (*BIRC5* rs9904341, *IL17A* rs2275913, and *XRCC1* rs3213245) showed moderate genetic associations (ORs range 1.5-2.0) and 12 variants showed low associations (ORs range 0.67-1.50) with lung cancer where one (*TERT* rs2736098) showed highly significant association ($P = 4.97\times10^{-11}$) (**Supplementary Table S6**). Of the 15 significant variants, ten were identified from meta-analyses with little or no heterogeneity ($I^2 < 25\%$), four (*CHRNA5* rs16969968, *GSTM1* present/null, *IL17A* rs2275913, and *MDM2* rs2279744) were identified from meta-analyses with moderate heterogeneity ($25\% \leq I^2 \leq 50\%$), and one (*BIRC5* rs9904341) was identified from meta-analysis with high heterogeneity. Based on the examinations of potential bias, only one variant (*GSTM1* present/null) had association with evidence of potential bias of small study effect and inclusion of excess significant findings. When the initial study was excluded, the association for three variants (*MIR146A* rs2910164, *SOD2* rs4880, and *XPC* PAT-/+) was no longer

significant. When studies included HWE-violating controls were excluded, the association for two variants (*ERCC2* rs238406 and *MTHFR* rs1801131) was no longer significant.

In the SCC subgroup, statistical significant associations were found for eight variants, where three (*SOD2* rs4880, *TNF* rs1800629, and *MMP2* rs243865) and five (*CYP1A1* rs4646903 and rs1048943, *GSTM1* present/null, *TERT* rs2736098, and *CYP2E1* rs6413432) showed moderate genetic associations ("risk" ORs range 1.5-2.0, or "protective" ORs range 0.50-0.67) and low genetic associations (ORs range 0.67-1.50) with lung cancer risk, respectively (**Supplementary Table S6**). Of note, one variant (*CYP1A1* rs4646903) with association showed low strength (OR 1.45, 95%CI 1.26-1.67) but had highly statistical significant (P = 3.77×10^{-7}). Of the eight significant variants, three (*CYP1A1* rs4646903, *CYP2E1* rs6413432, and *TERT* rs2736098) were identified from meta-analyses with little or no heterogeneity (I^2 < 25%) and five (*CYP1A1* rs1048943, *GSTM1* present/null, *MMP2* rs243865, *SOD2* rs4880, and *TNF* rs1800629) were identified from meta-analyses with high heterogeneity. Two variants (*CYP1A1* rs1048943 and *MMP2* rs243865) had associations with evidence of potential bias of small study effects and one variant (*GSTM1* present/null) had association of small effect size (OR 1.23, 95%CI 1.08-1.40) with evidence of potential bias of inclusion of excess significant findings. Based on the sensitivity analyses, three variants (*MMP2* rs243865, *SOD2* rs4880, and *TNF* rs1800629) lost significant associations with lung cancer risk after relative meta-analysis removing the initial study.

Subgroup meta-analyses by smoking status

Subgroup meta-analyses by smoking status were conducted separately in smokers and non-smokers under each of the three genetic models (allelic, dominant, or recessive model), if sufficient data were available. Sixty variants were evaluated in smokers and thirty-eight variants in non-smokers (**Supplementary Table S7**).

In smokers, 22 (36.7%) variants significantly associated with lung cancer. The strength of association between each genetic variant and lung cancer as measured by ORs had the mean of 1.32 (range 1.07-1.64) for putative "risk" variants and 0.74 (range 0.58-0.95) for putative "protective" variants (**Supplementary Table S8**). Of the 22 significant variants, two (*GSTP1* rs1138272 and *CYP2A6* rs5031016) showed moderate genetic associations ("risk" ORs range 1.5-2.0, or "protective" ORs range 0.50-0.67) and 20 showed low associations (ORs range 0.67-1.50) with lung cancer, including one variant (*CHRNA5* rs16969968) showed highly significant association ($P = 2.77\times10^{-10}$). Of the 22 significant variants, 12 were identified from meta-analyses with little or no heterogeneity ($I^2 < 25\%$), five (*APEX1* rs1760944, *CYP1A1* rs4646903, *SOD2* rs4880, *XRCC1* rs1799782 and rs25487) were identified from meta-analyses with moderate heterogeneity ($25\% \leq I^2 \leq 50\%$), and five (*CYP1A1* rs1048943, *CYP2A6* rs5031016, *GSTM1* present/null, *GSTP1* rs1695, and *TP53* rs1042522) were identified from meta-analyses with high heterogeneity. Of the 22 significant variants, five (*CHRNA5* rs16969968, *CYP1A1* rs1048943, *GSTM1* present/null, *NAT2* rs1799930, and *XRCC1* rs25489) were identified from meta-analyses showing evidence of potential bias of small study effects and two (*CYP1A1* rs1048943 and *TP53* rs1042522) were identified from meta-analyses showing evidence of inclusion of studies with excess significant findings and having small summary ORs (OR 1.42, 95%CI 1.04-1.93, association for *CYP1A1* rs1048943;

OR 1.23, 95%CI 1.01-1.50, association for *TP53* rs1042522). When sensitivity analyses removing the initial study, 11 (*CYP1A1* rs1048943, *CYP1B1* rs1800440 and rs1056836, *CYP2A6* rs5031016, *EPHX1* rs2234922, *MTHFR* rs1801131, *SOD2* rs4880, *TP53* rs1042522, *XRCC1* rs25489, rs1799782, and rs25487) of 22 significant variants became non-significant. When sensitivity analyses removing studies with HWE-violating controls, four variants (*CYP1A1* rs1048943, *XRCC1* rs25489, rs1799782, and rs25487) became non-significant.

In non-smokers, statistical significant associations were found for ten variants, where two (*CYP1A1* rs1048943 and *ERCC2* rs13181) and eight (*ATM* rs189037, *GSTM1* present/null, *OGG1* rs1052133, *TP53* rs1042522, *XRCC1* rs3213245, *CYP2E1* rs6413432, rs2031920, and *ERCC1* rs11615) showed moderate genetic associations (ORs range 1.5-2.0) and low genetic associations (ORs range 0.67-1.50) with lung cancer risk, respectively (**Supplementary Table S8**). Of the ten significant variants, six (*CYP2E1* rs6413432 and rs2031920, *ERCC1* rs11615, *ERCC2* rs13181, *OGG1* rs1052133, and *XRCC1* rs3213245) and four (*ATM* rs189037, *CYP1A1* rs1048943, *GSTM1* present/null, and *TP53* rs10425220) were identified from meta-analyses with little or no heterogeneity ($I^2 < 25\%$) and moderate heterogeneity ($25\% \leq I^2 \leq 50\%$), respectively. Of the ten significant variants, the variant (*OGG1* rs1052133) and variant (*ATM* rs189037) were identified from meta-analyses showing evidence of potential publication bias and bias of small study effects, respectively (**Supplementary Table S8**). Based on the sensitivity analyses, two variants (*ATM* rs189037 and *CYP1A1* rs1048943) became non-significant associations with lung cancer risk after relative meta-analysis removing the initial study.

Supplementary Figures

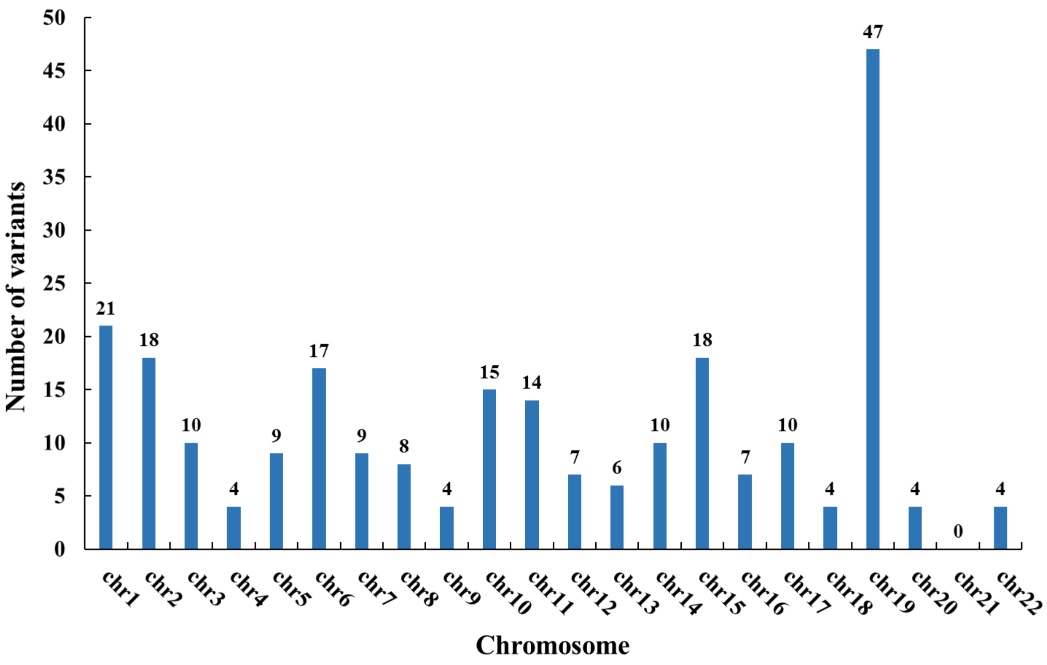

Supplementary Figure S1. Distribution by chromosome of 246 genetic variants available for meta-analyses.

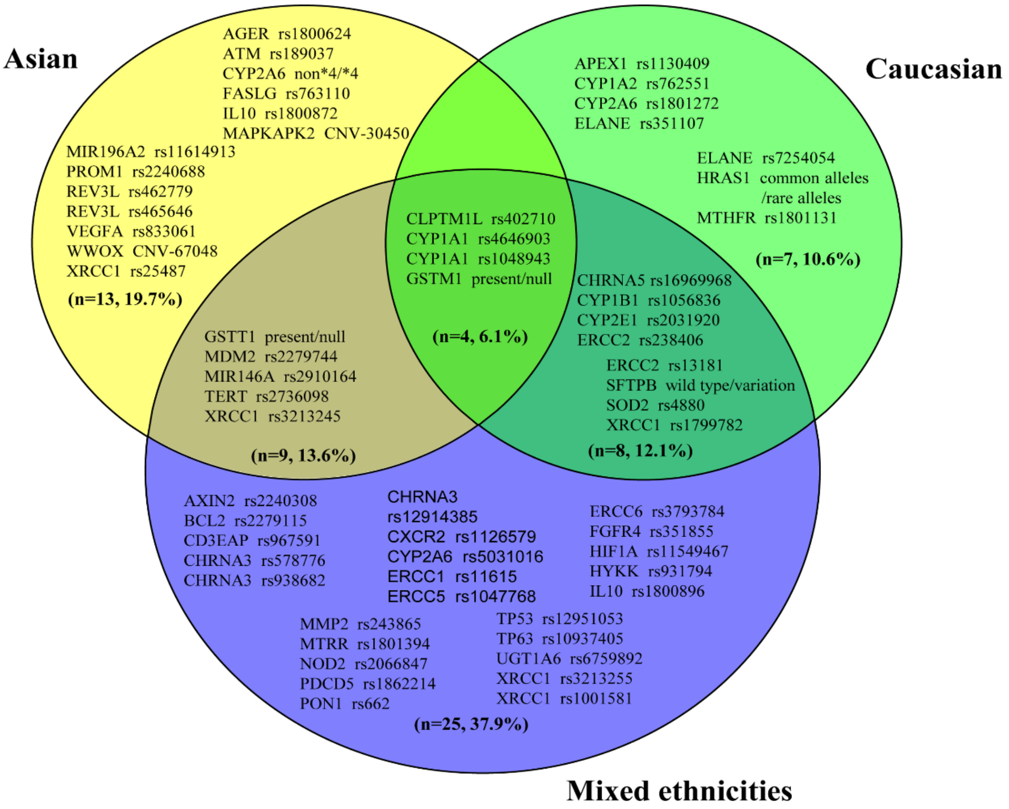

Supplementary Figure S2. Venn diagram of the distributions of genetic variants with significant associations with lung cancer risk in the main meta-analyses and subgroup meta-analyses by ethnicity (Asian and Caucasian).

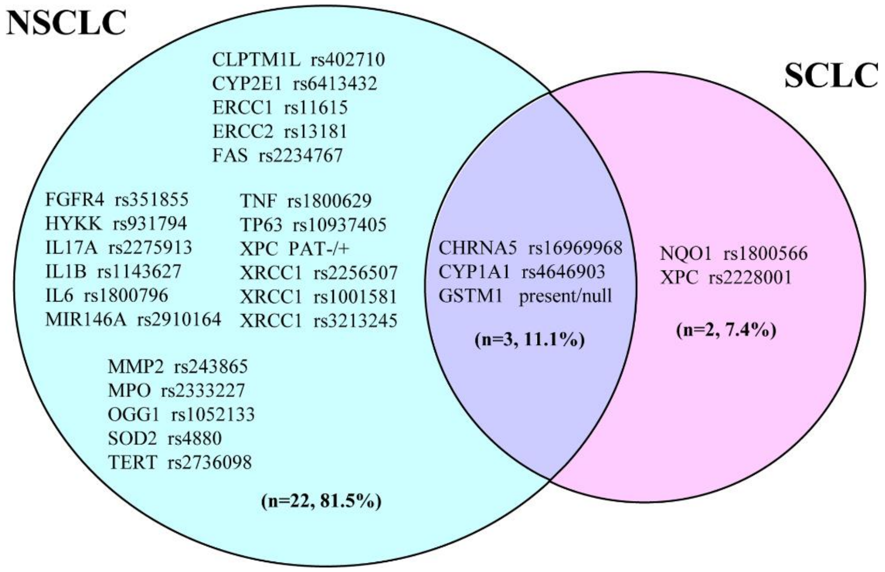

Supplementary Figure S3. Venn diagram of the distributions of genetic variants with significant associations with specific histological types of lung cancer. NSCLC: non-small cell lung cancer; SCLC: small cell lung cancer.

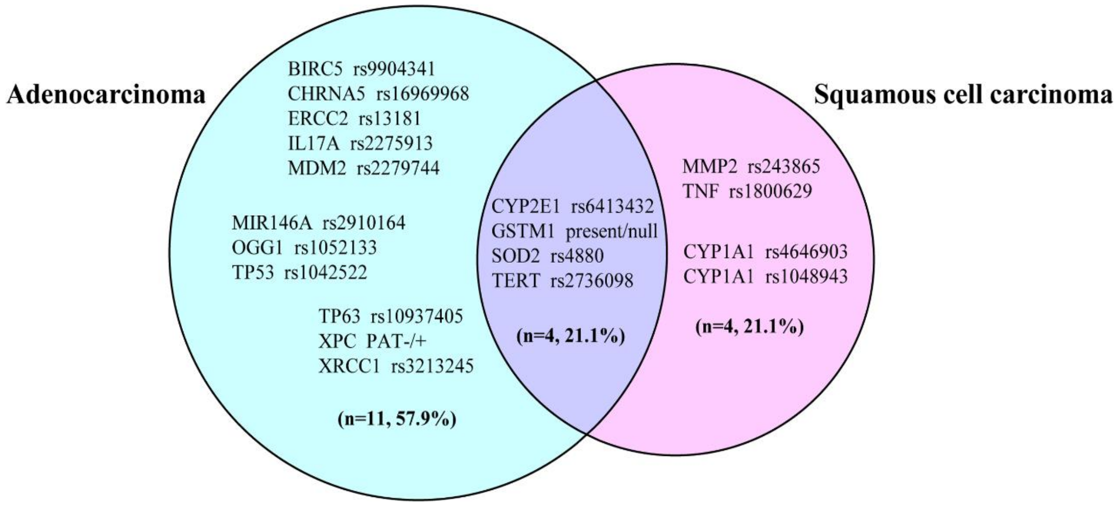

Supplementary Figure S4. Venn diagram of the distributions of genetic variants with significant associations with lung adenocarcinoma and squamous cell carcinoma.

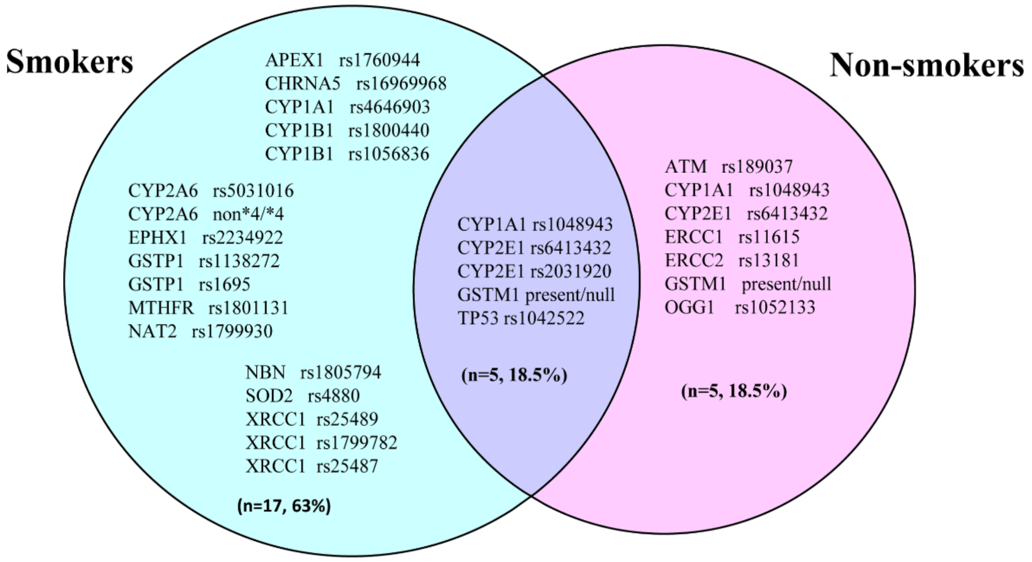

Supplementary Figure S5. Venn diagram of the distributions of genetic variants with significant associations in subgroup meta-analyses of smoker and non-smoker.

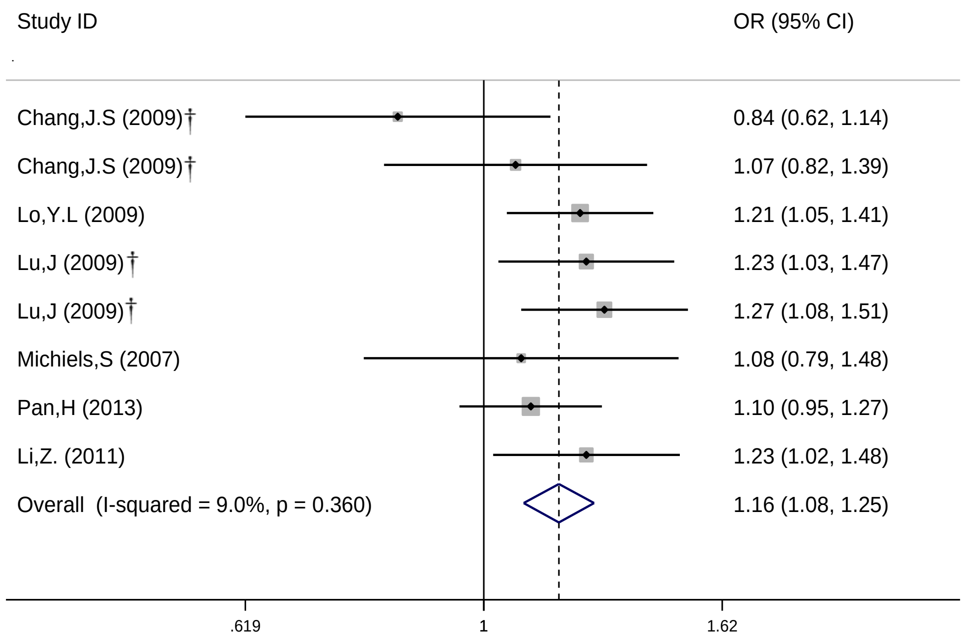

Supplementary Figure S6.1. Main meta-analysis of *APEX1* rs1760944 (allelic contrast: A vs. C).

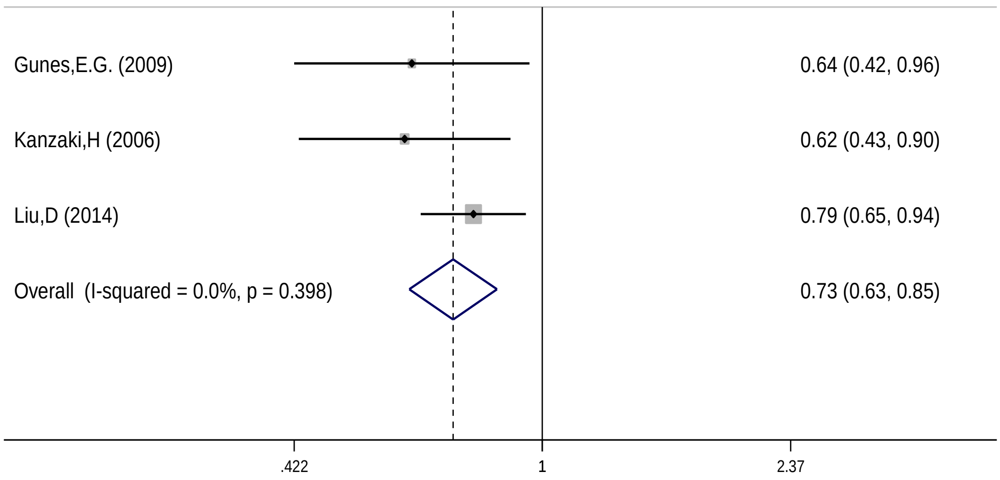

Supplementary Figure S6.2. Main meta-analysis of *AXIN2* rs2240308 (allelic contrast: T vs. C).

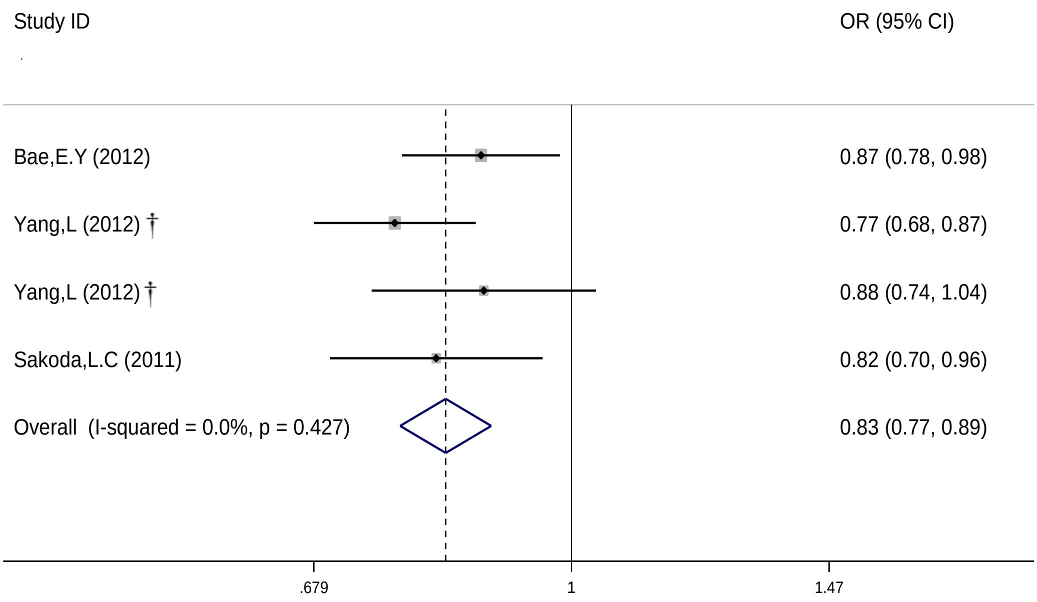

Supplementary Figure S6.3. Main meta-analysis of *CHRNA3* rs6495309 (allelic contrast: T vs. C).

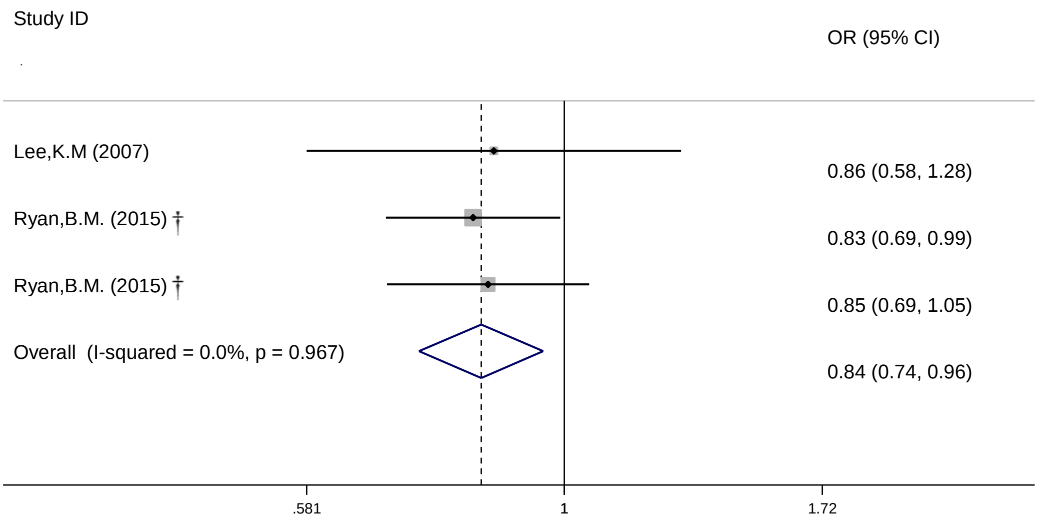

Supplementary Figure S6.4. Main meta-analysis of *CXCR2* rs1126579 (allelic contrast: T vs. C).

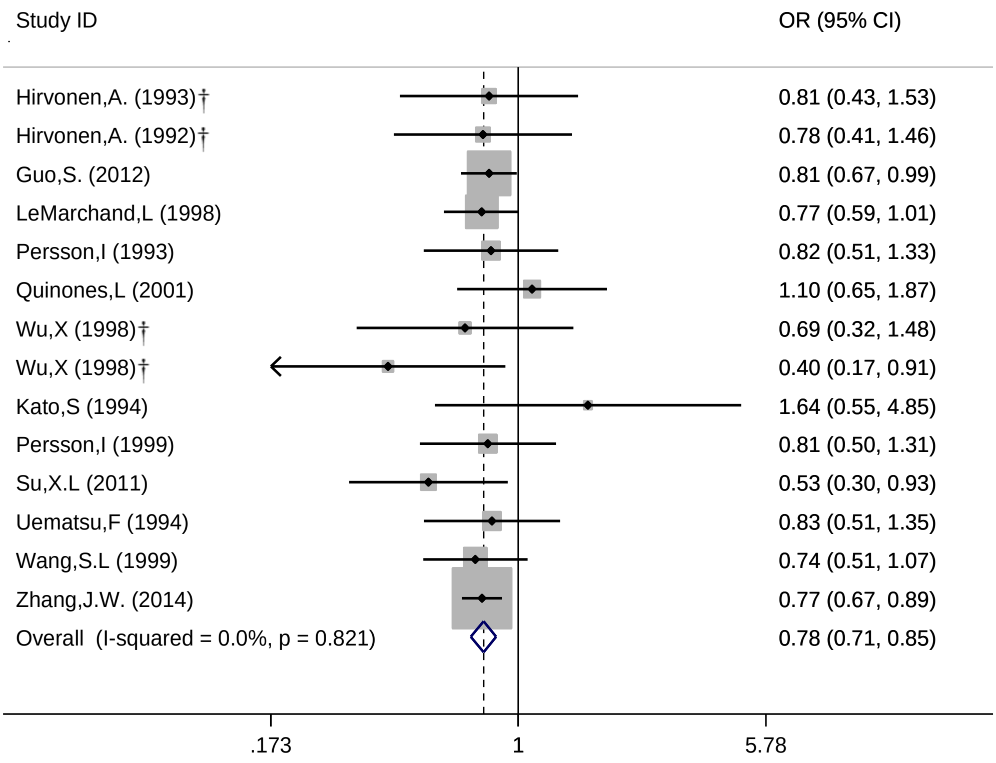

Supplementary Figure S6.5. Main meta-analysis of *CYP2E1* rs6413432 (allelic contrast: A vs. T).

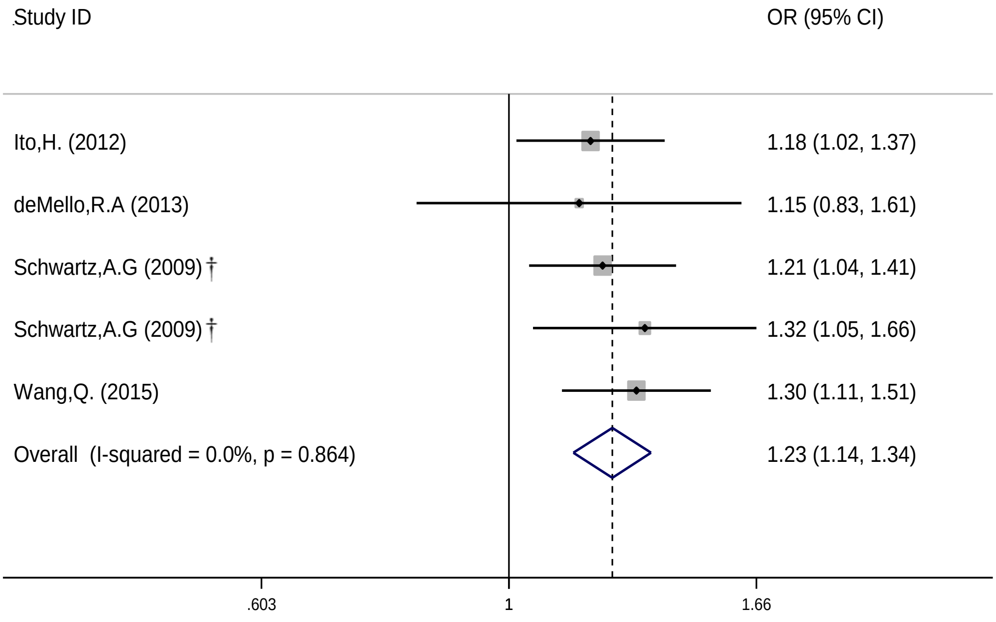

Supplementary Figure S6.6. Main meta-analysis of *HYKK* rs931794 (allelic contrast:

G vs. A).

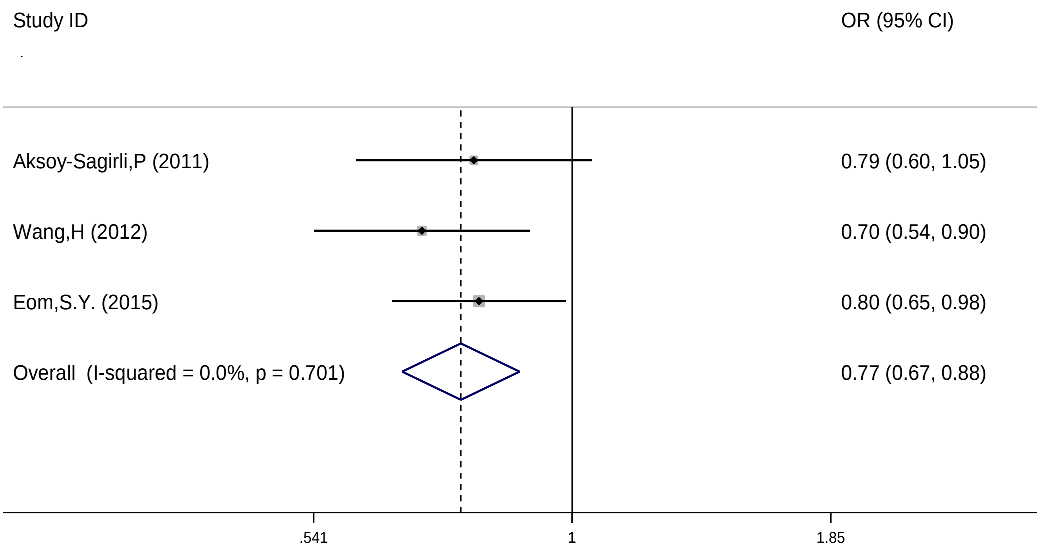

Supplementary Figure S6.7. Main meta-analysis of *PON1* rs662 (allelic contrast: A vs. G).

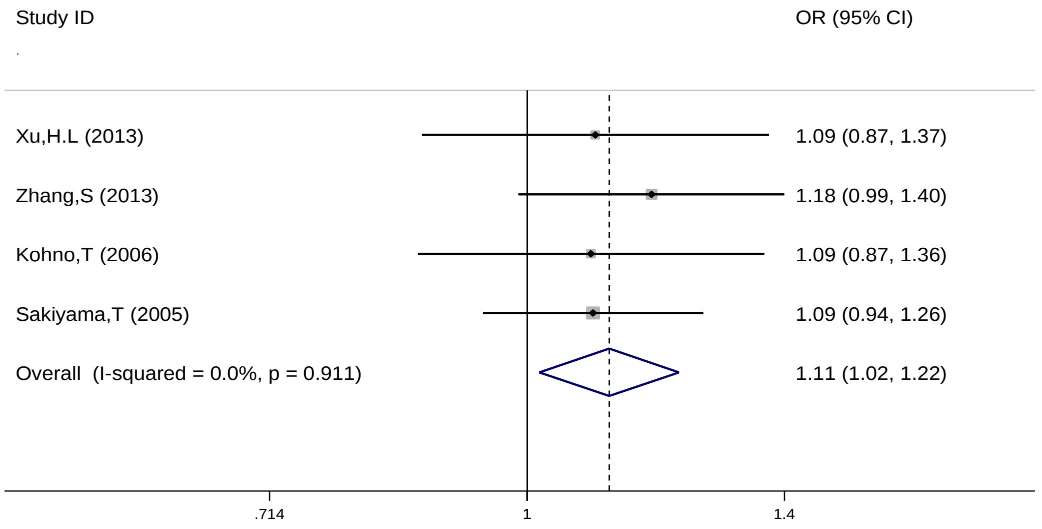

Supplementary Figure S6.8. Main meta-analysis of *REV3L* rs462779 (allelic contrast: T vs. C). (Available data were limited to Asian populations.)

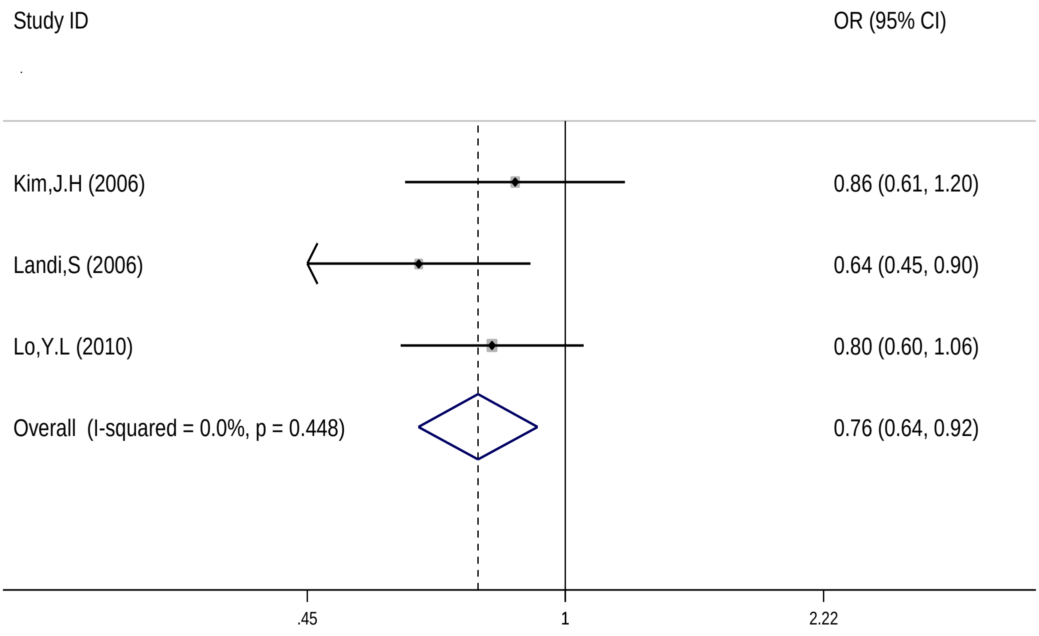

Supplementary Figure S6.9. Meta-analysis of *ATM* rs664677 (Dominant: CC+CT vs. TT).

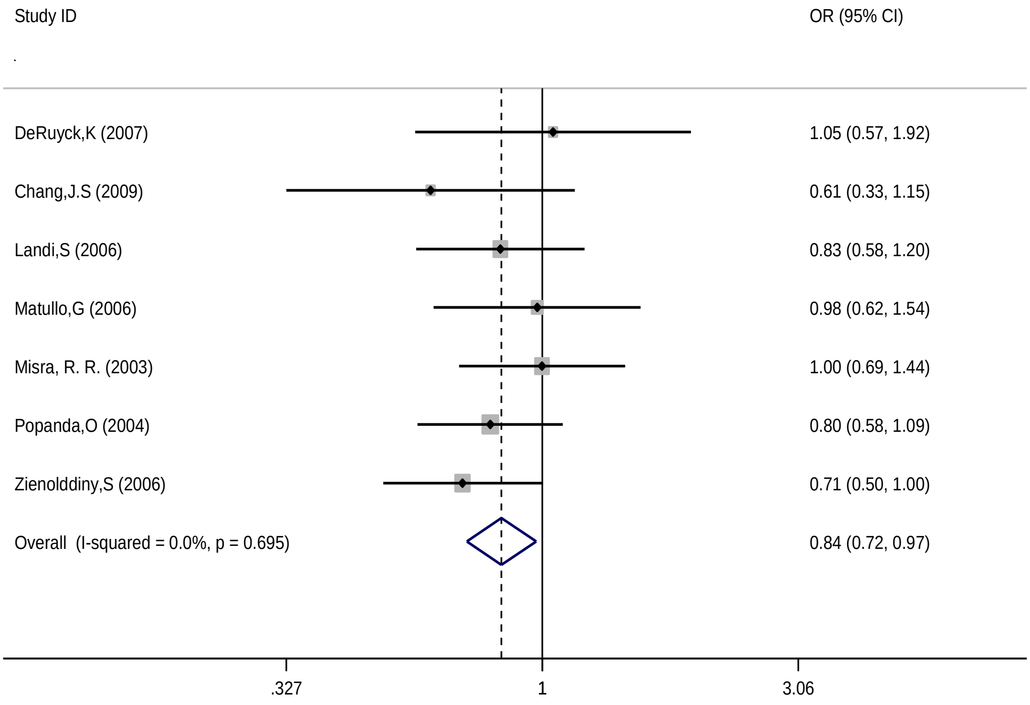

Supplementary Figure S6.10. Subgroup meta-analysis of *APEX1* rs1130409 in Caucasian (Recessive: TT vs. GG+GT).

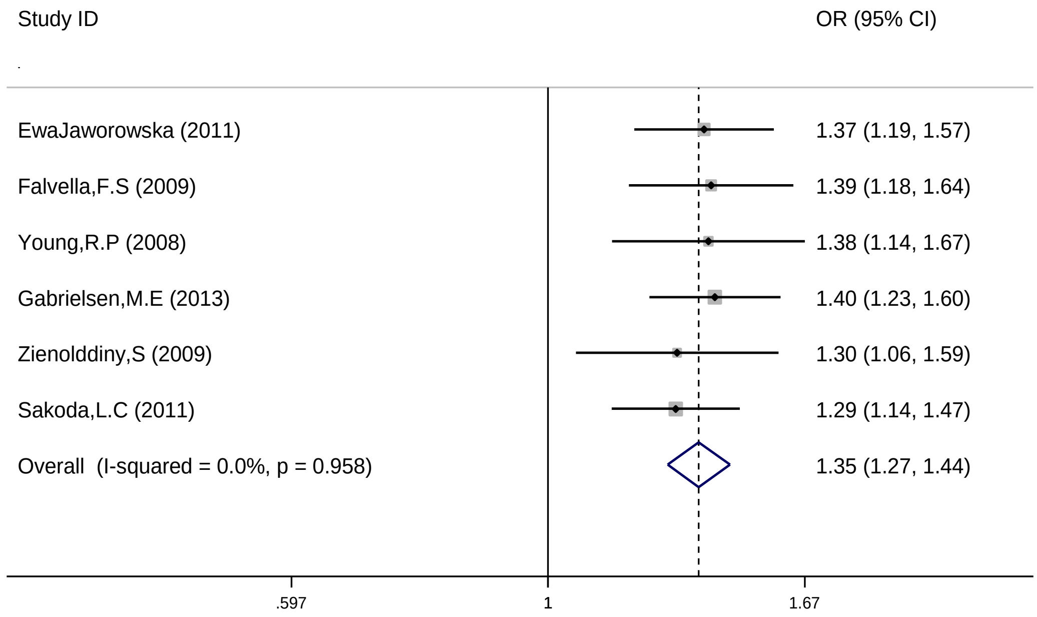

Supplementary Figure S6.11. Subgroup meta-analysis of *CHRNA5* rs16969968 in Caucasian (allelic contrast: A vs. G).

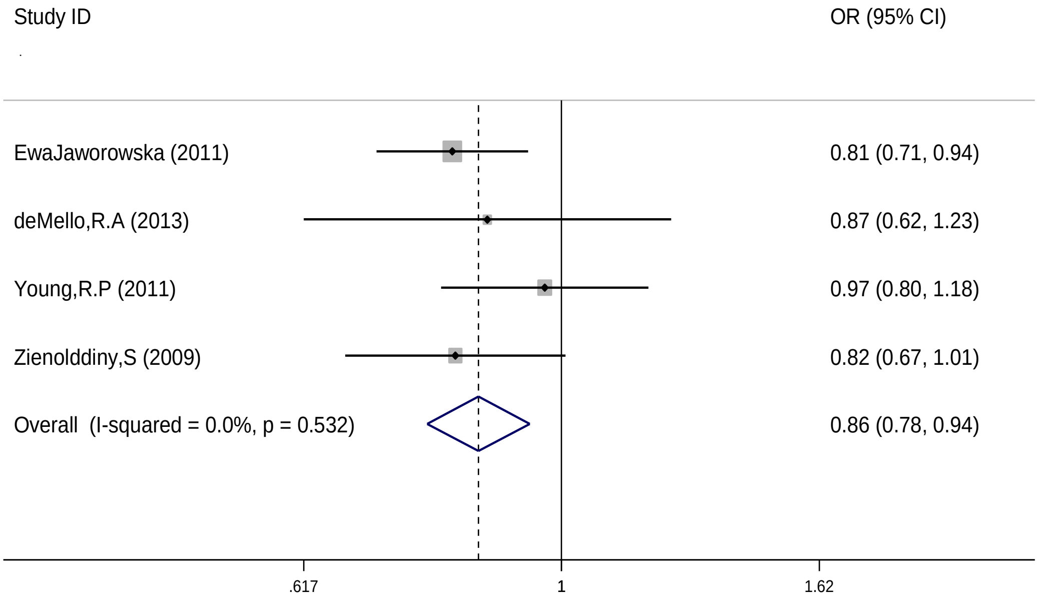

Supplementary Figure S6.12. Subgroup meta-analysis of *CLPTM1L* rs402710 in Caucasian (allelic contrast: T vs. C).

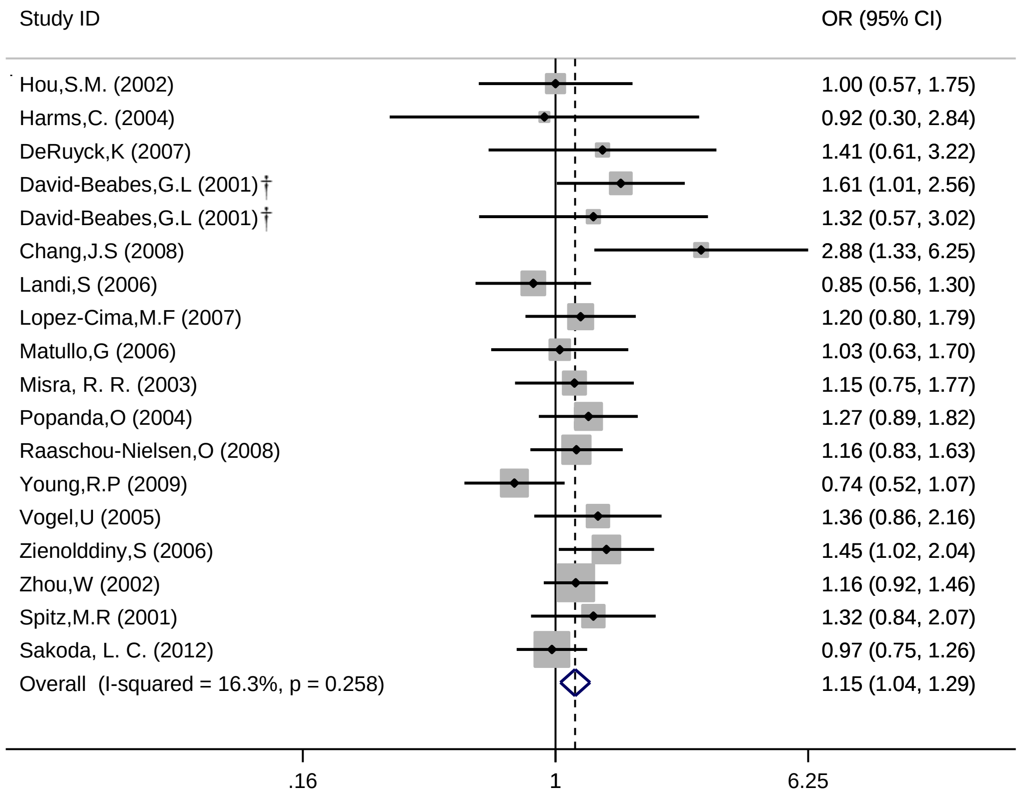

Supplementary Figure S6.13. Subgroup meta-analysis of *ERCC2* rs13181 in Caucasian (Recessive: AA vs. CC+CA).

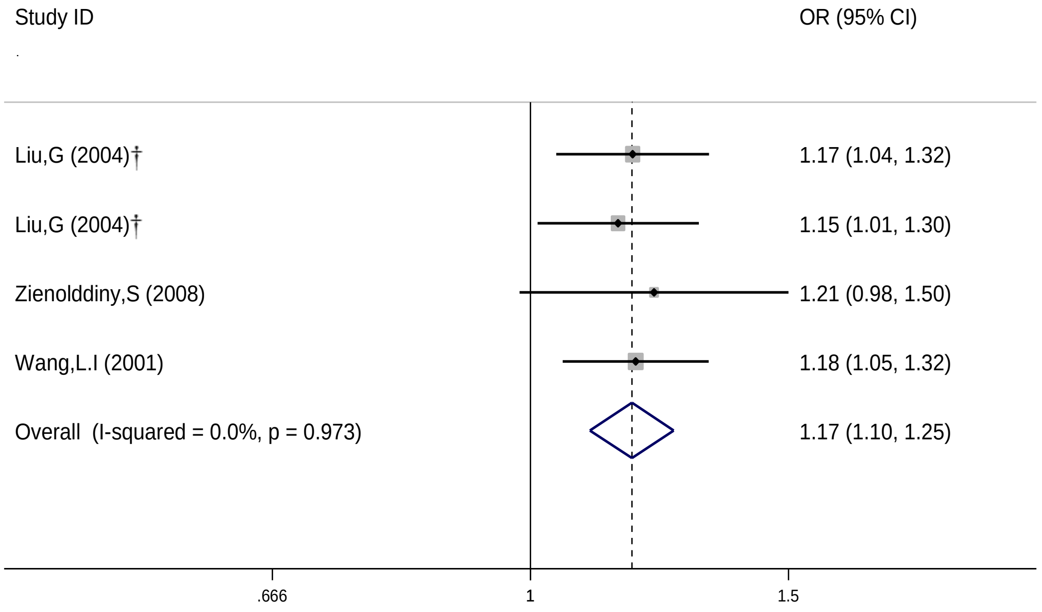

Supplementary Figure S6.14. Subgroup meta-analysis of *SOD2* rs4880 in Caucasian (allelic contrast: T vs. C).

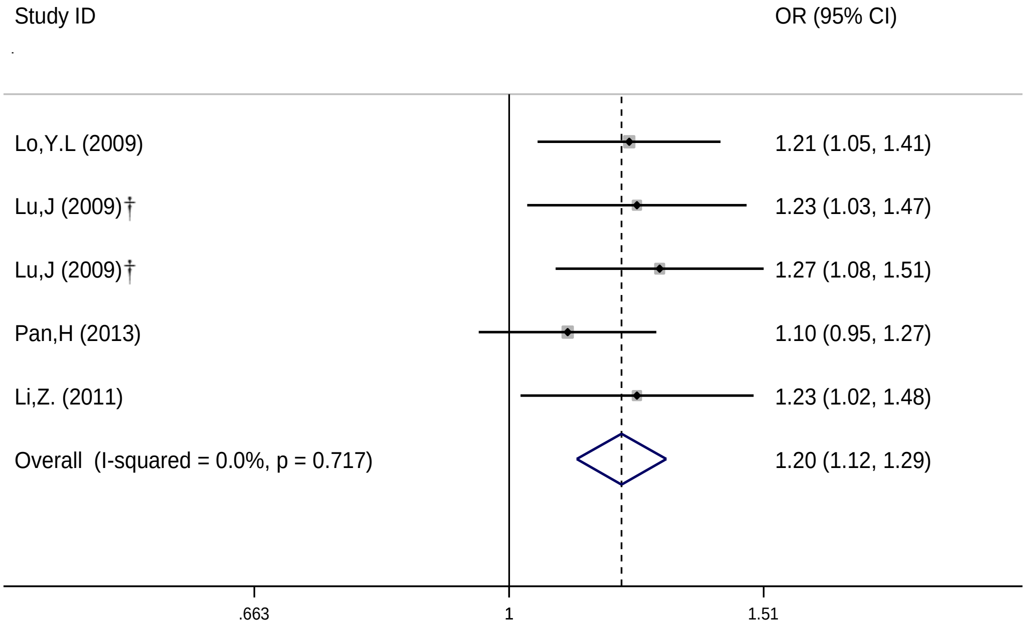

Supplementary Figure S6.15. Subgroup meta-analysis of *APEX1* rs1760944 in Asian (allelic contrast: A vs. C).

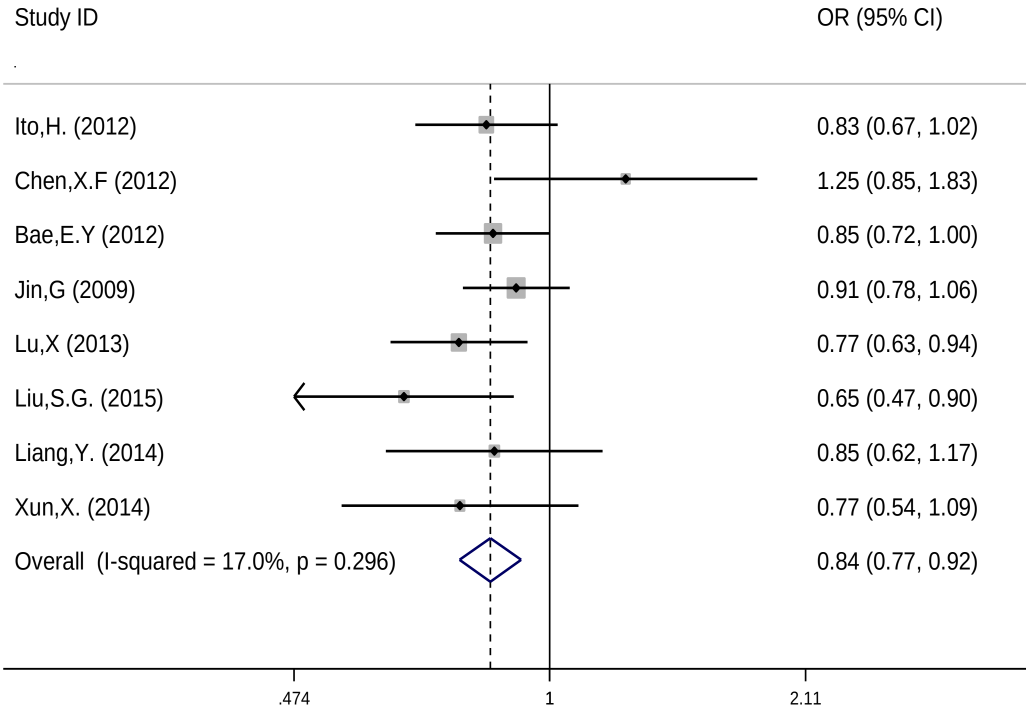

Supplementary Figure S6.16. Subgroup meta-analysis of *CLPTM1L* rs402710 in Asian (Dominant: TT+TC vs. CC).

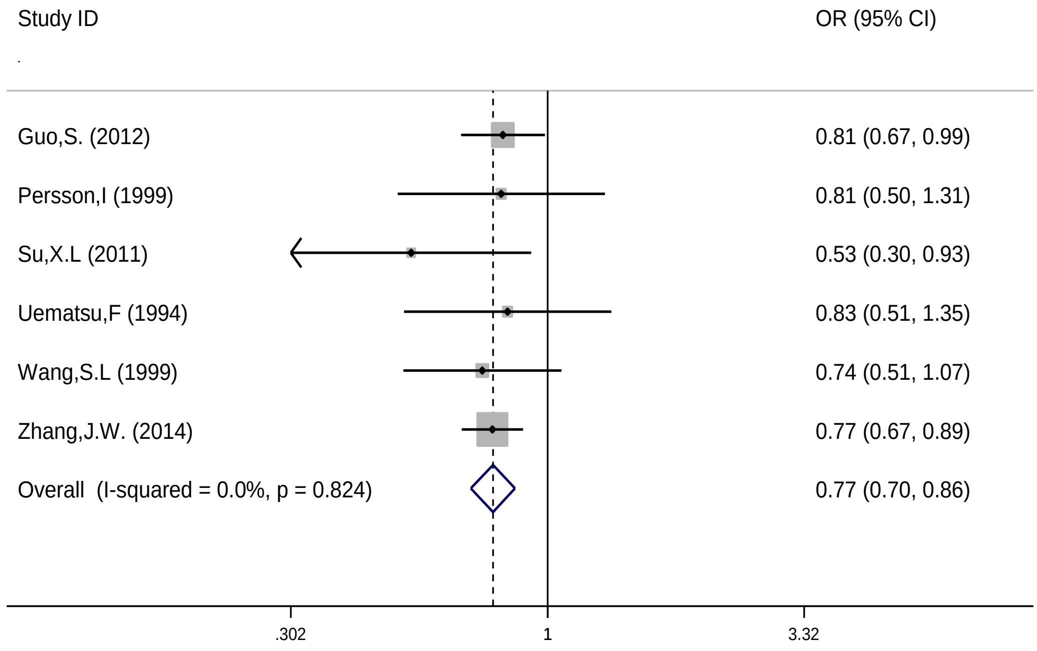

Supplementary Figure S6.17. Subgroup meta-analysis of *CYP2E1* rs6413432 in Asian (allelic contrast: A vs. T).

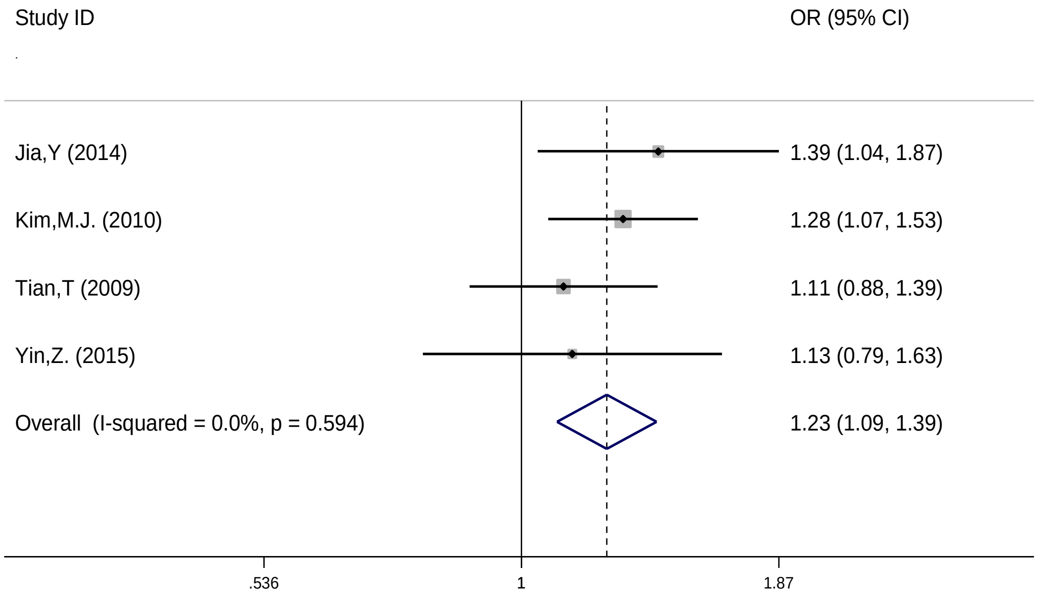

Supplementary Figure S6.18. Subgroup meta-analysis of *MIR146A* rs2910164 in Asian (Recessive: GG vs. CC+CG).

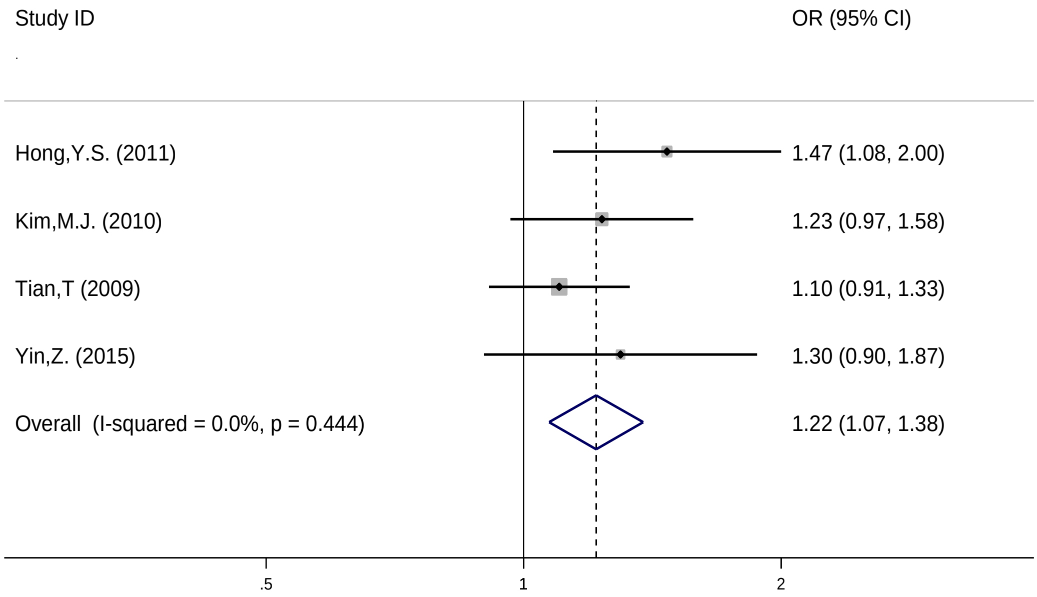

Supplementary Figure S6.19. Subgroup meta-analysis of *MIR196A2* rs11614913 in Asian (Dominant: CC+CT vs. TT).

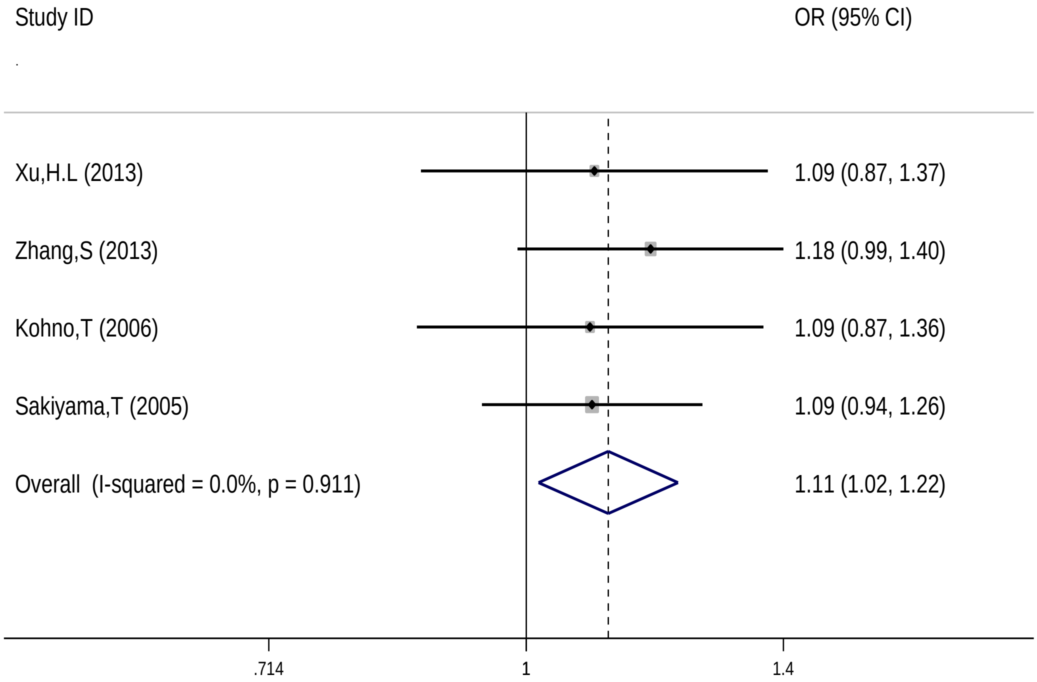

Supplementary Figure S6.20. Subgroup meta-analysis of *REV3L* rs462779 in Asian (allelic contrast: T vs. C).

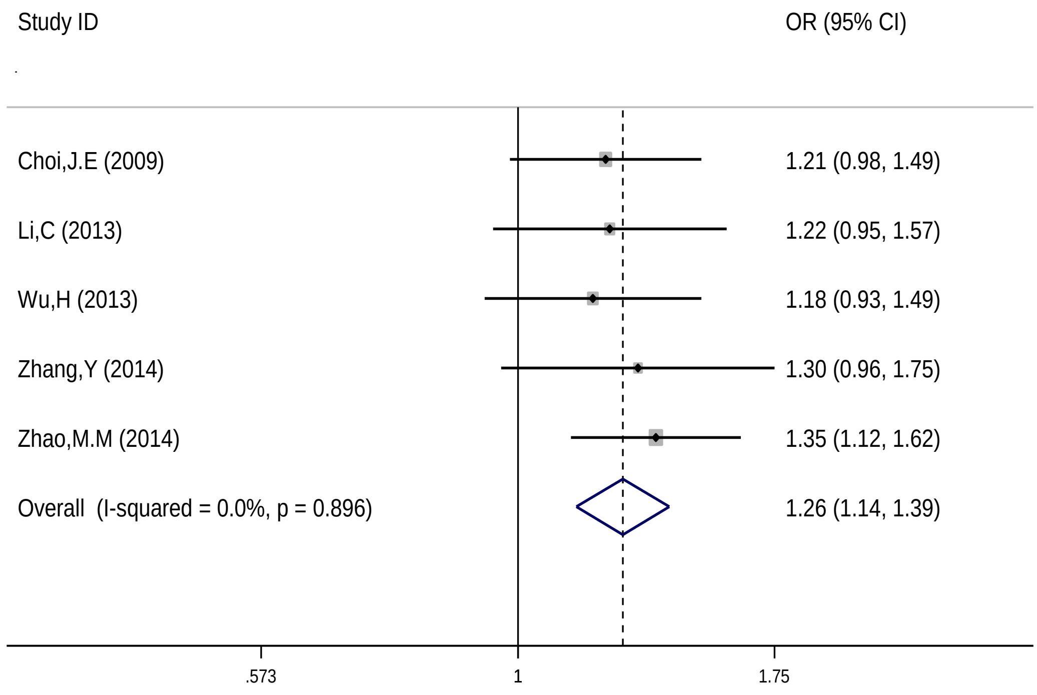

Supplementary Figure S6.21. Subgroup meta-analysis of *TERT* rs2736098 in Asian (Dominant: AA+AG vs. GG).

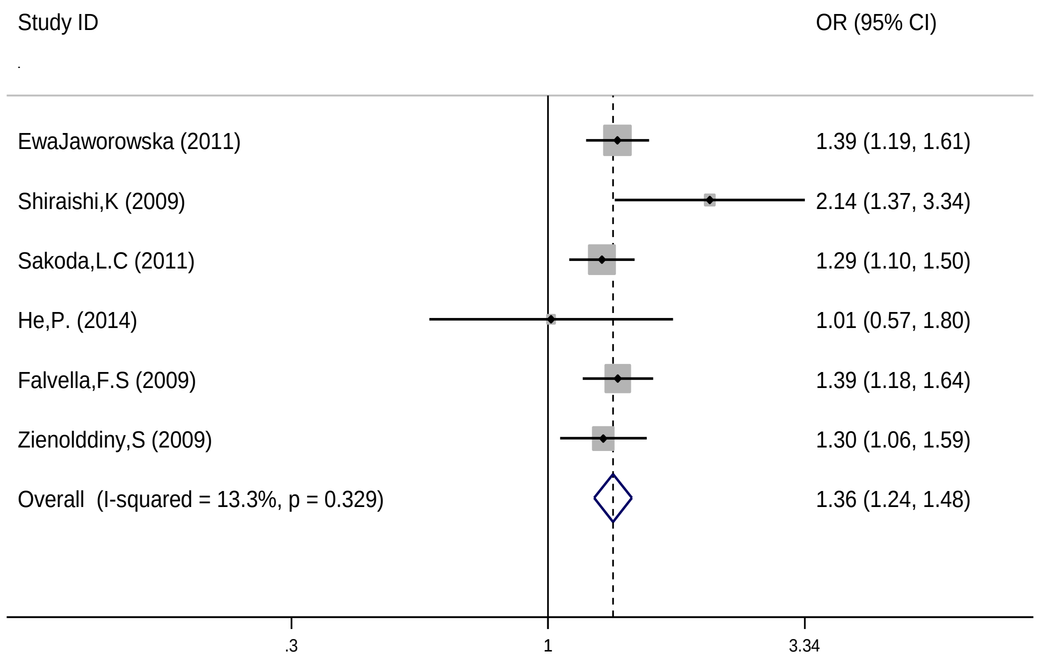

Supplementary Figure S6.22. Subgroup meta-analysis of *CHRNA5* rs16969968 in non-small cell lung cancer population (allelic contrast: A vs. G).

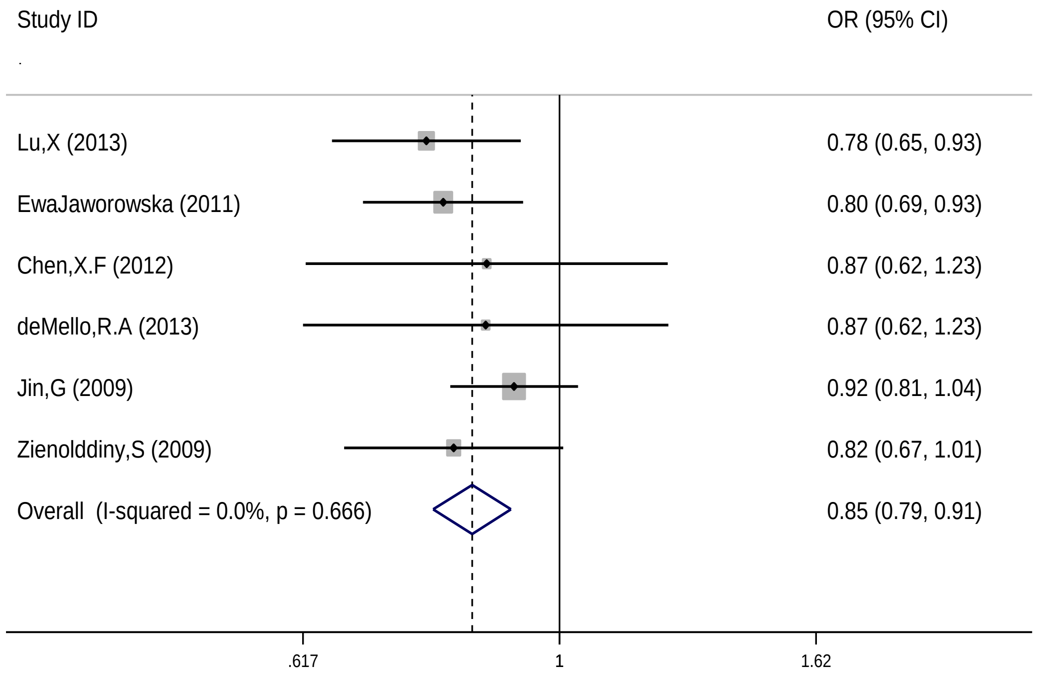

Supplementary Figure S6.23. Subgroup meta-analysis of *CLPTM1L* rs402710 in non-small cell lung cancer population (allelic contrast: T vs. C).

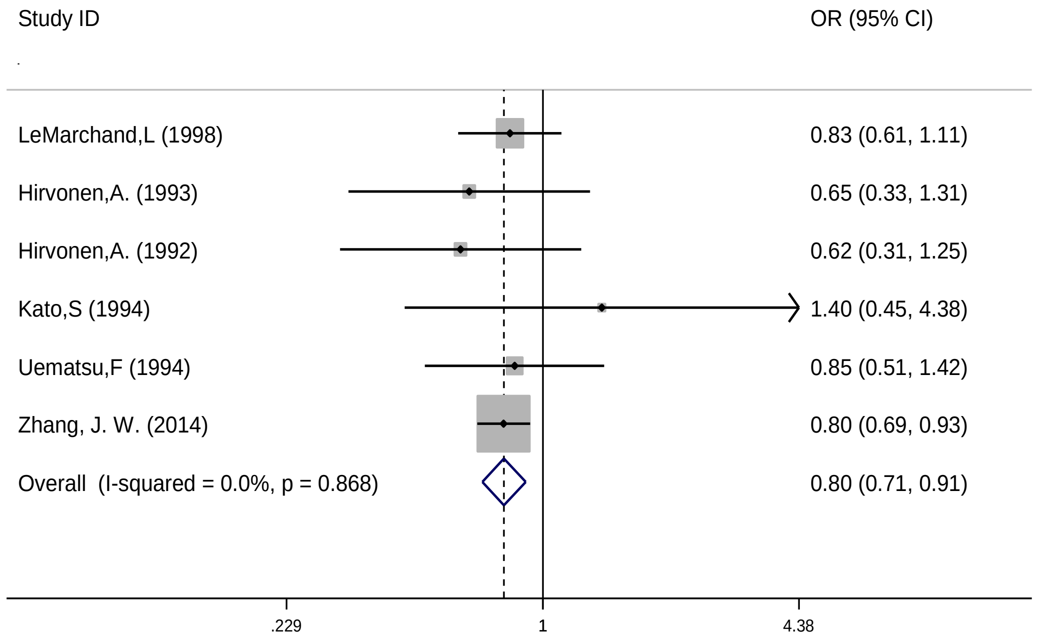

Supplementary Figure S6.24. Subgroup meta-analysis of *CYP2E1* rs6413432 in non-small cell lung cancer population (allelic contrast: A vs. T).

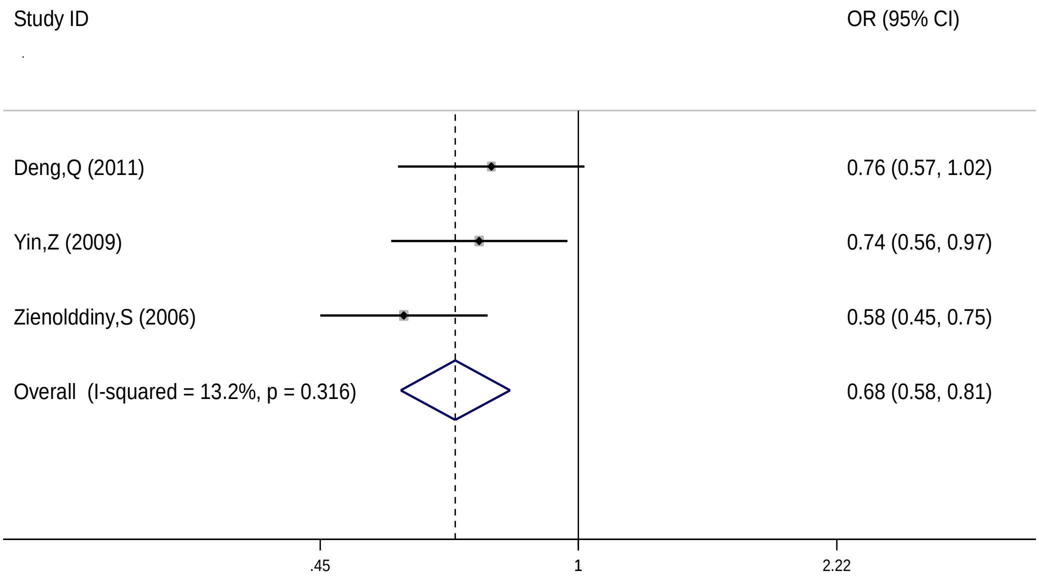

Supplementary Figure S6.25. Subgroup meta-analysis of *ERCC1* rs11615 in non-small cell lung cancer population (allelic contrast: C vs. T).

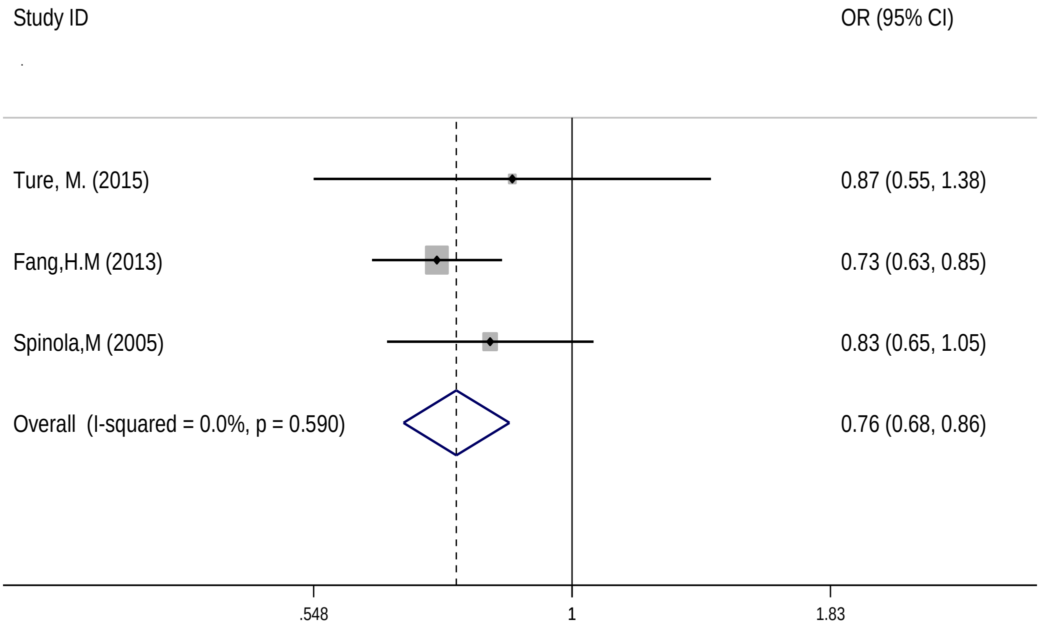

Supplementary Figure S6.26. Subgroup meta-analysis of *FGFR4* rs351855 in non-small cell lung cancer population (allelic contrast: A vs. G).

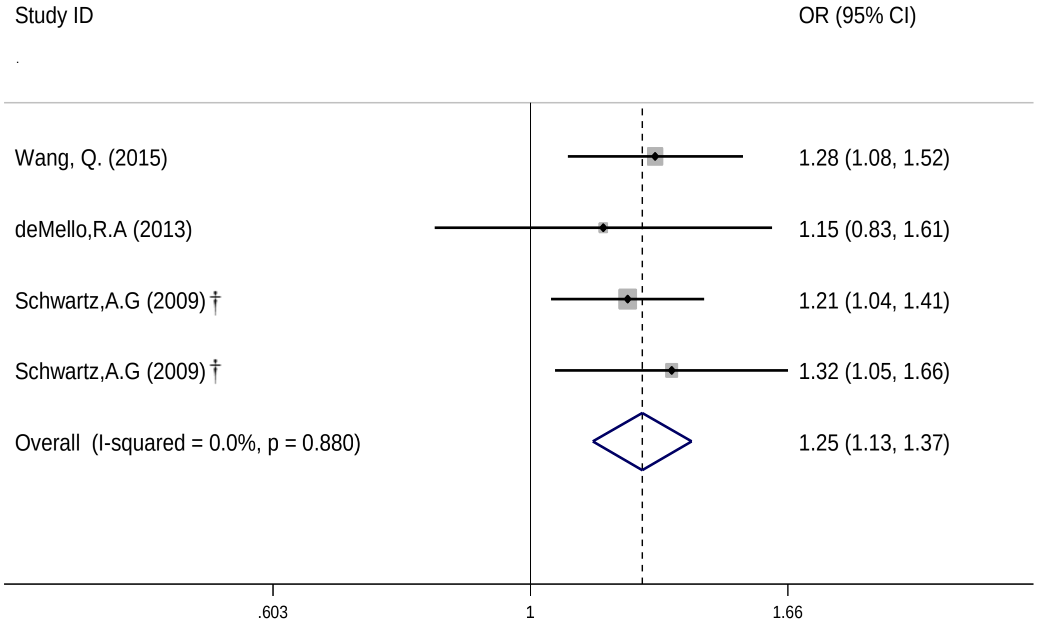

Supplementary Figure S6.27. Subgroup meta-analysis of *HYKK* rs931794 in non-small cell lung cancer population (allelic contrast: G vs. A).

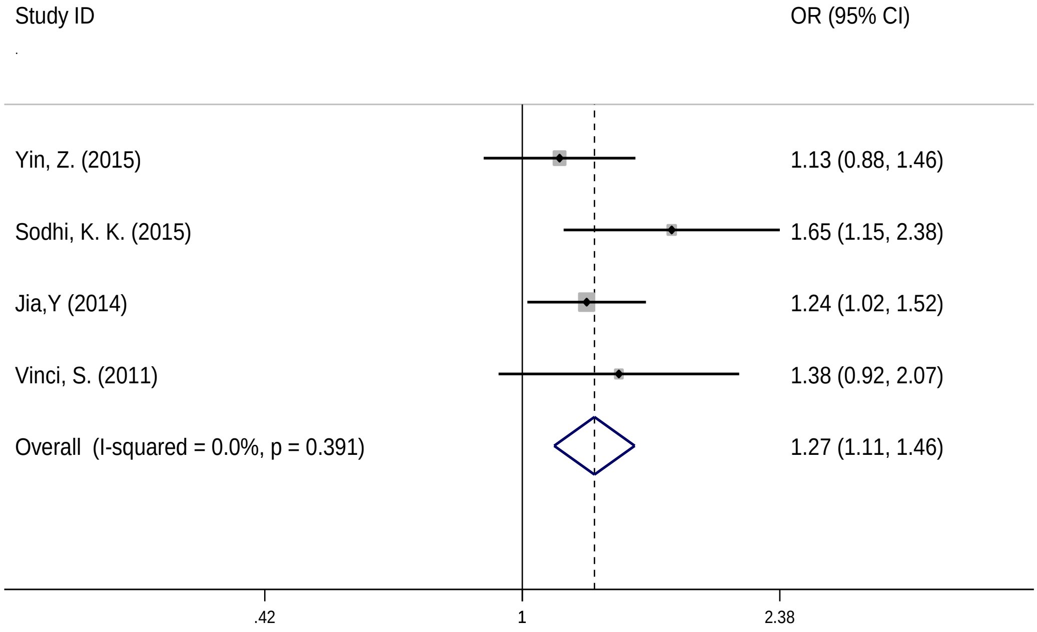

Supplementary Figure S6.28. Subgroup meta-analysis of *MIR146A* rs2910164 in non-small cell lung cancer population (allelic contrast: C vs. G).

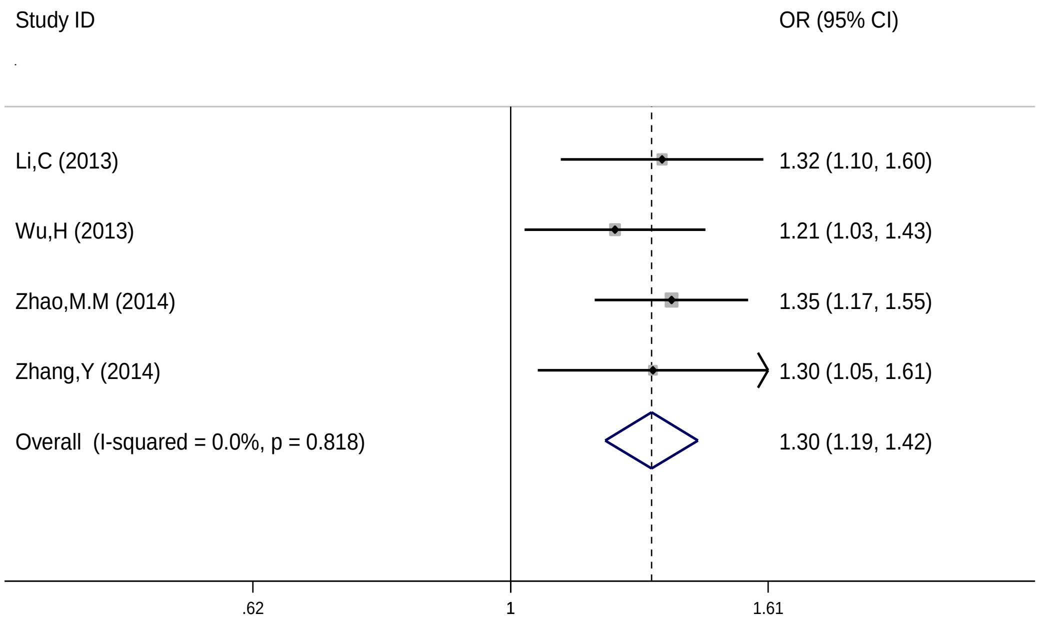

Supplementary Figure S6.29. Subgroup meta-analysis of *TERT* rs2736098 in non-small cell lung cancer population (allelic contrast: A vs. G).

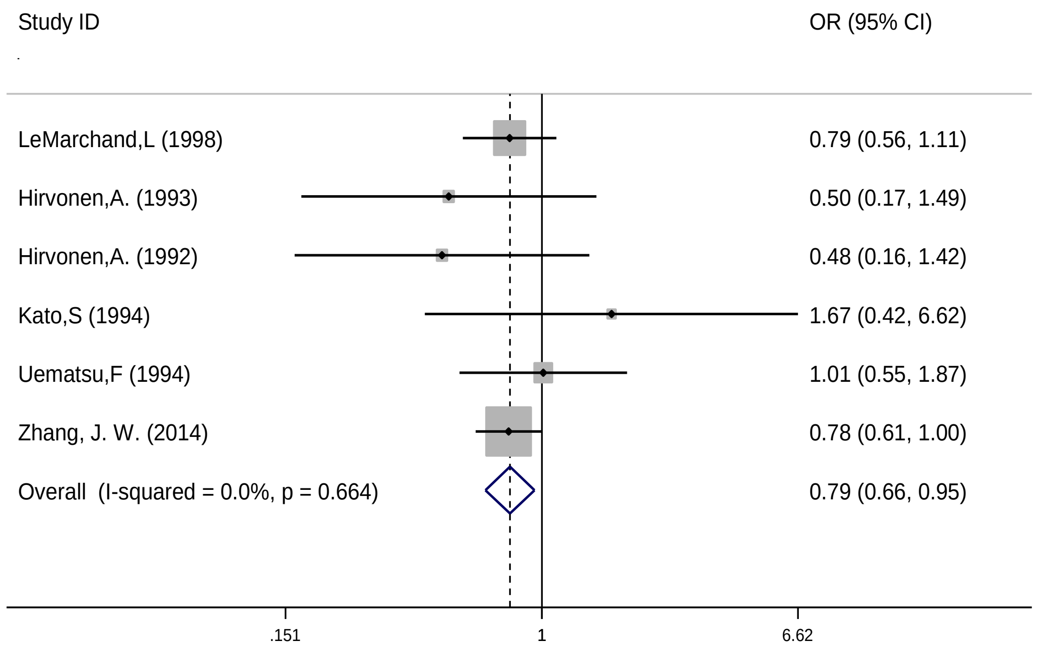

Supplementary Figure S6.30. Subgroup meta-analysis of *CYP2E1* rs6413432 in adenocarcinoma population (allelic contrast: A vs. T).

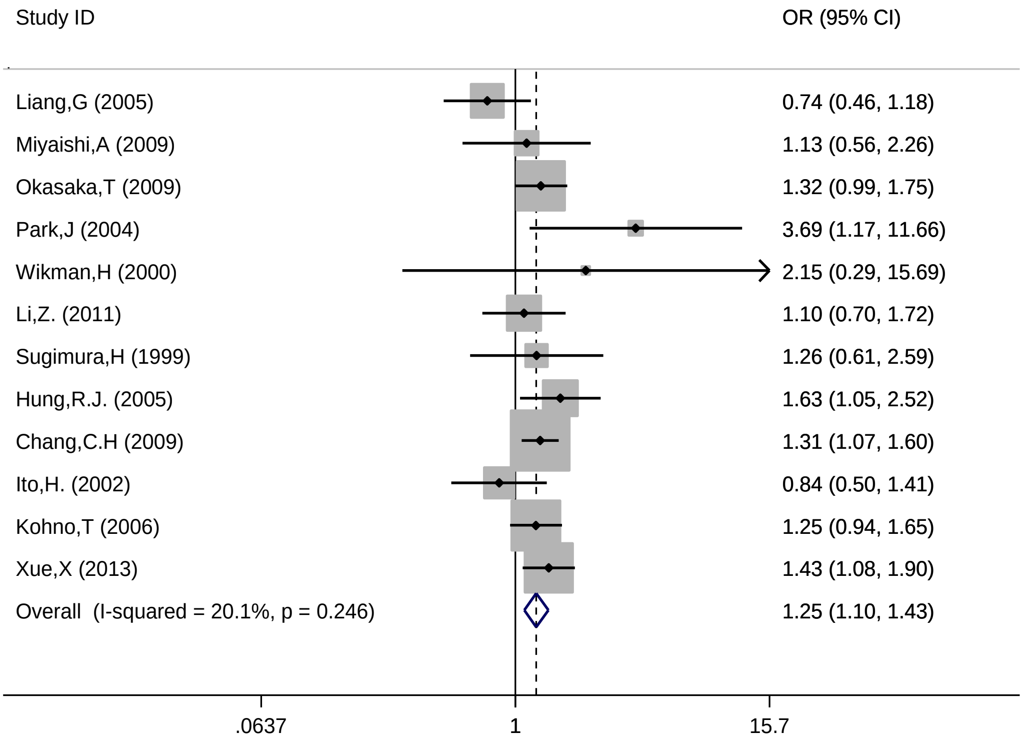

Supplementary Figure S6.31. Subgroup meta-analysis of *OGG1* rs1052133 in adenocarcinoma population (Recessive: CC vs. GG+GC).

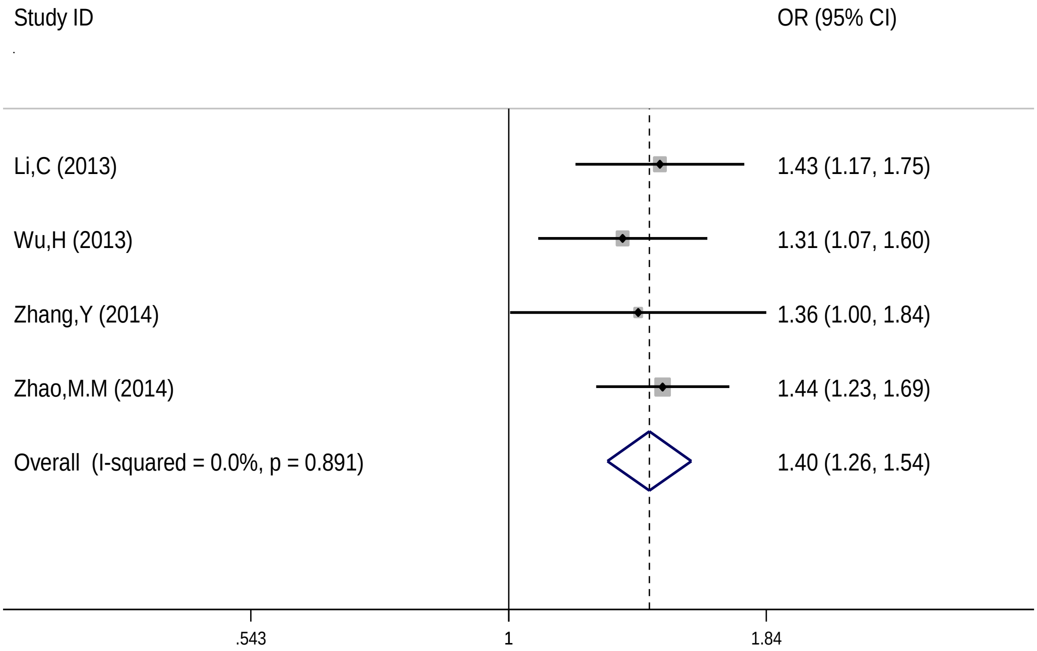

Supplementary Figure S6.32. Subgroup meta-analysis of *TERT* rs2736098 in adenocarcinoma population (allelic contrast: A vs. G).

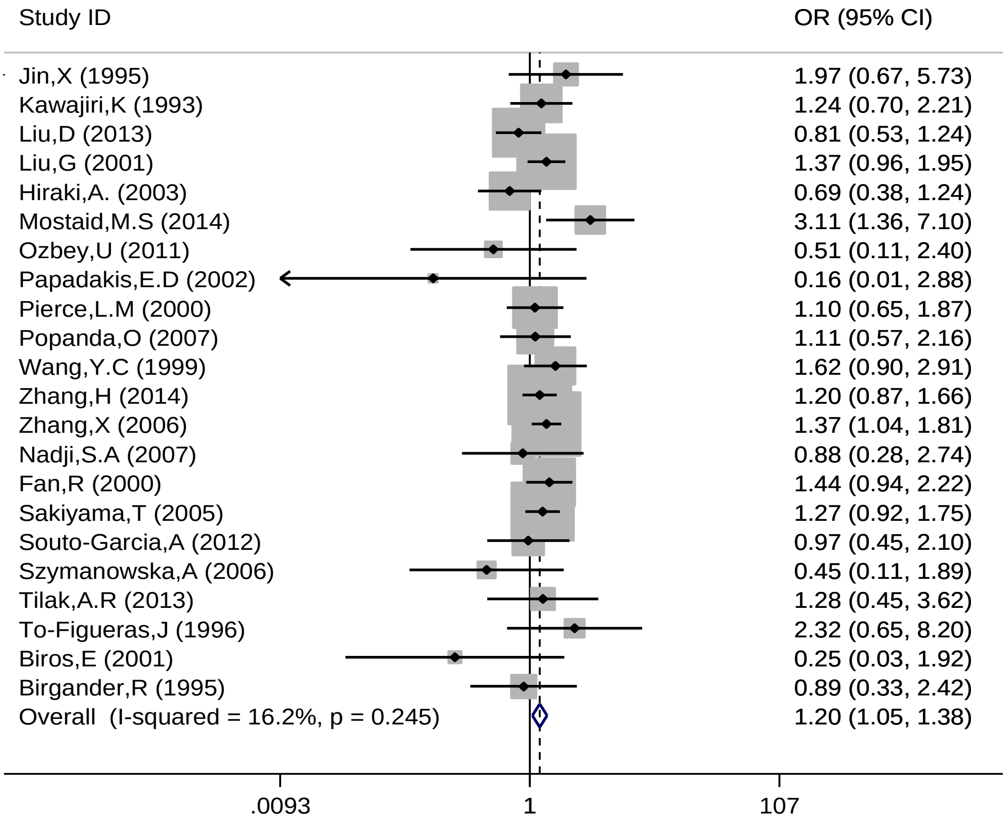

Supplementary Figure S6.33. Subgroup meta-analysis of *TP53* rs1042522 in adenocarcinoma population (Recessive: GG vs. CC+CG).

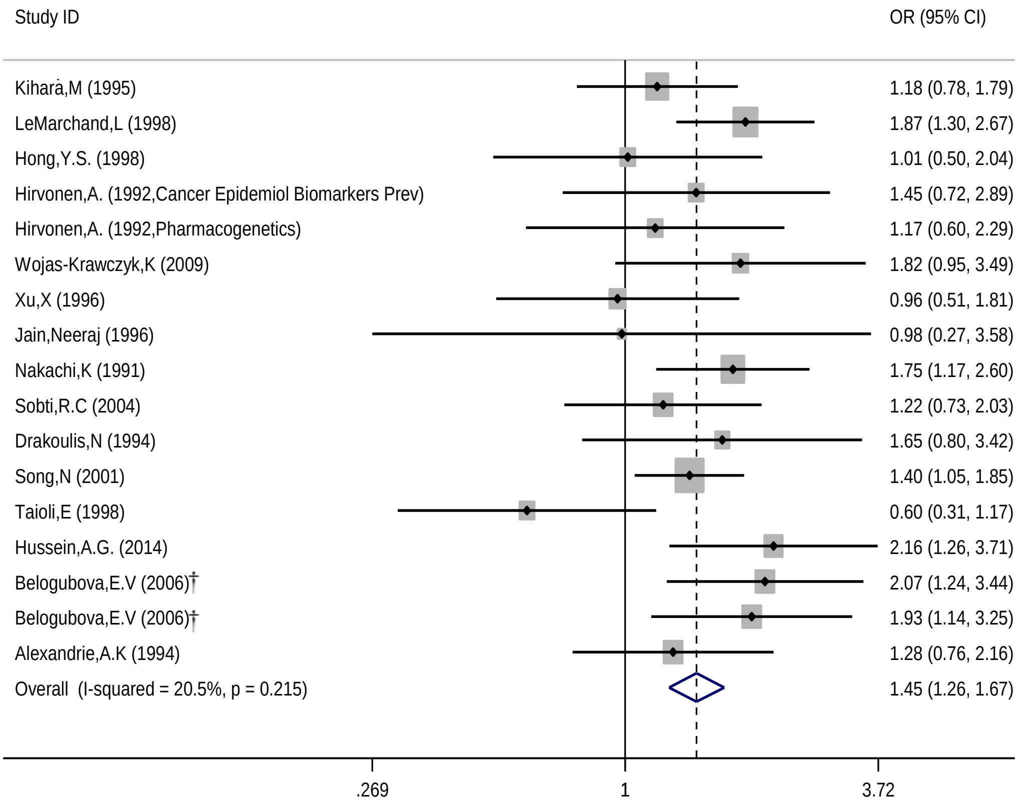

Supplementary Figure S6.34. Subgroup meta-analysis of *CYP1A1* rs4646903 in squamous cell carcinoma population (allelic contrast: C vs. T).

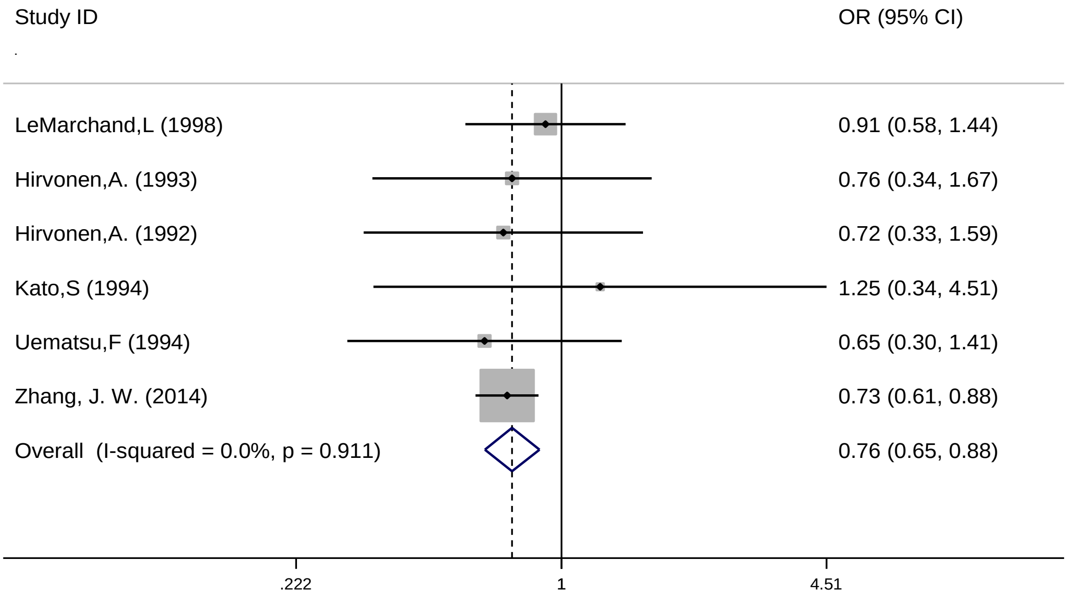

Supplementary Figure S6.35. Subgroup meta-analysis of *CYP2E1* rs6413432 in squamous cell carcinoma population (allelic contrast: A vs. T).

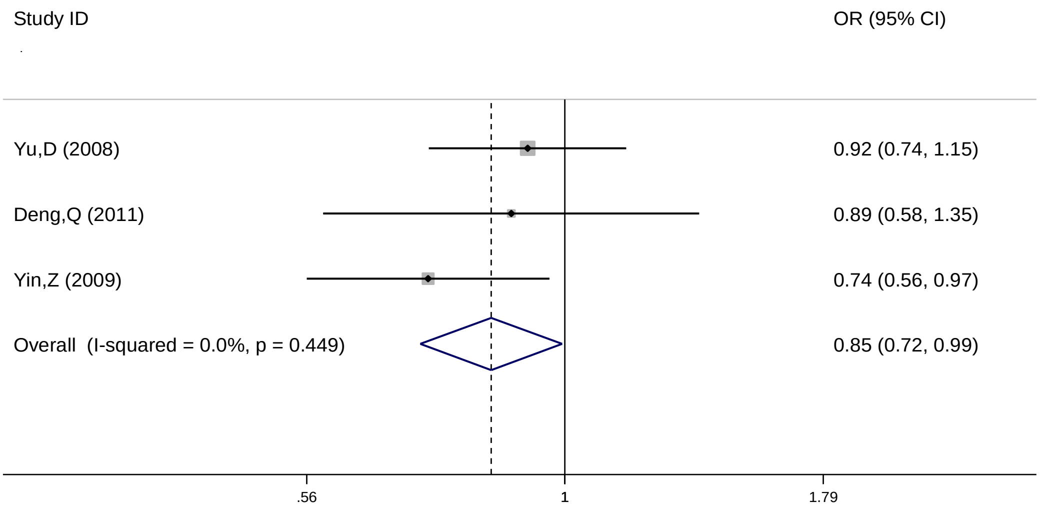

Supplementary Figure S6.36. Subgroup meta-analysis of *ERCC1* rs11615 in non-smokers (allelic contrast: C vs. T).

Supplementary Figure S6. Forest plots of main or subgroup meta-analyses for variants showed significant associations to lung cancer susceptibility with strong cumulative evidence. Study-specific allelic odds ratios (ORs, gray squares) and 95% confidence intervals (CIs, lines) were calculated for each included independent dataset. The summary OR and CI was calculated using DerSimonian and Laird random-effects models (white diamond), and genetic model used for meta-analysis was annotated in each single panel. For a complete list of meta-analyses performed for these variants, see **Supplementary Table S2**, **Table S4**, **Table S6**, and **Table S8**. †A report included two independent study populations or different racial groups, and data for each independent population was extracted and analyzed separately.

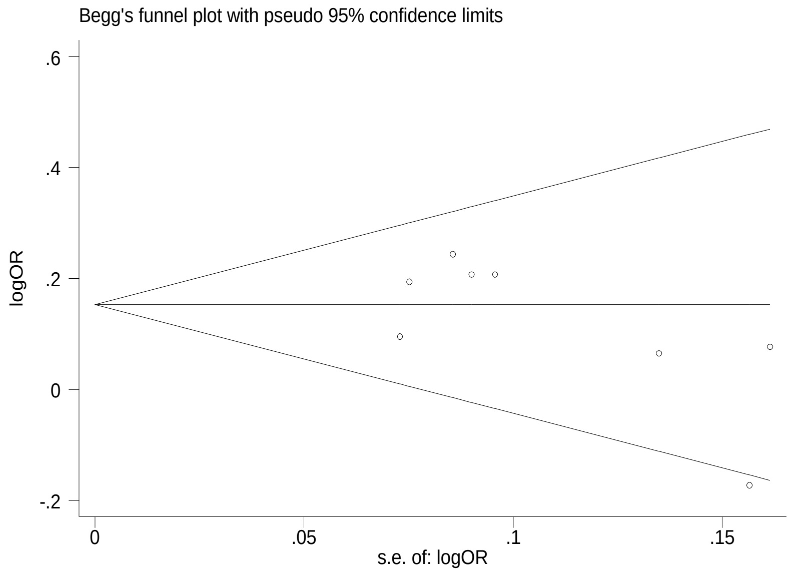

Supplementary Figure S7.1. Main meta-analysis of *APEX1* rs1760944 (allelic contrast: A vs. C).

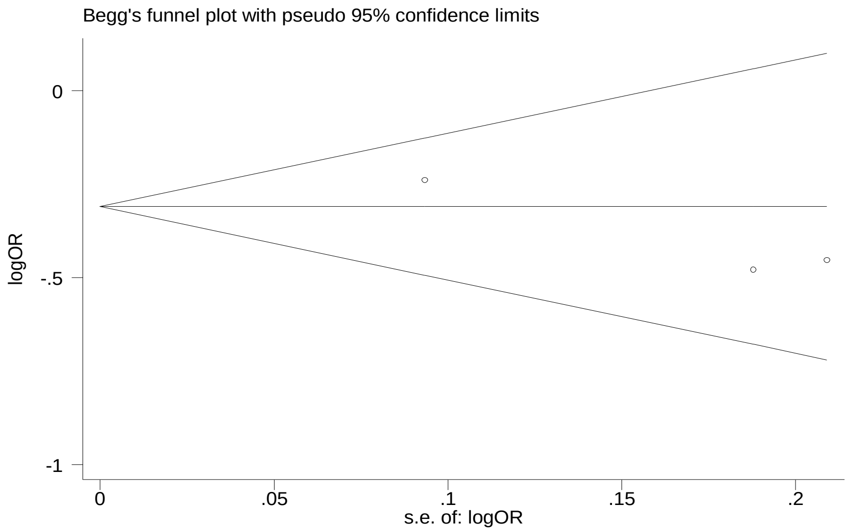

Supplementary Figure S7.2. Main meta-analysis of *AXIN2* rs2240308 (allelic contrast: T vs. C).

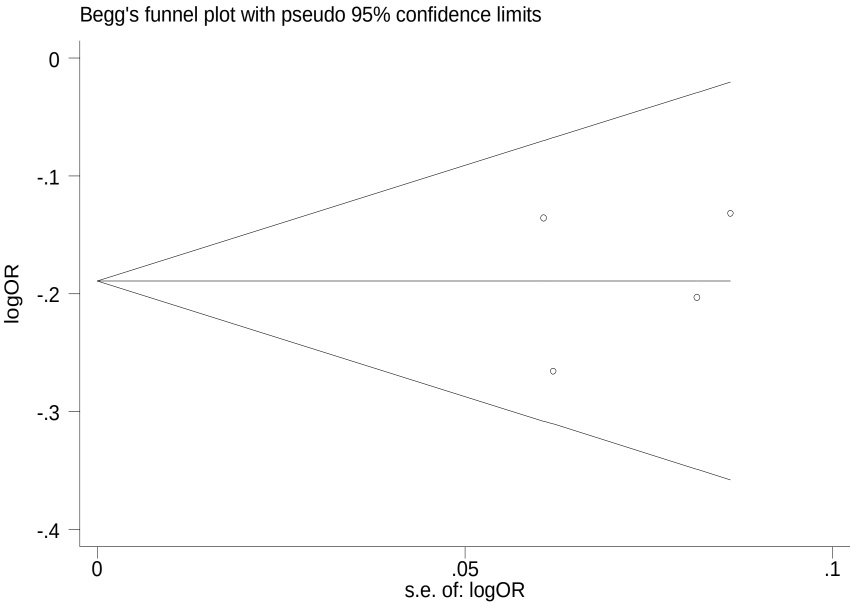

Supplementary Figure S7.3. Main meta-analysis of *CHRNA3* rs6495309 (allelic contrast: T vs. C).

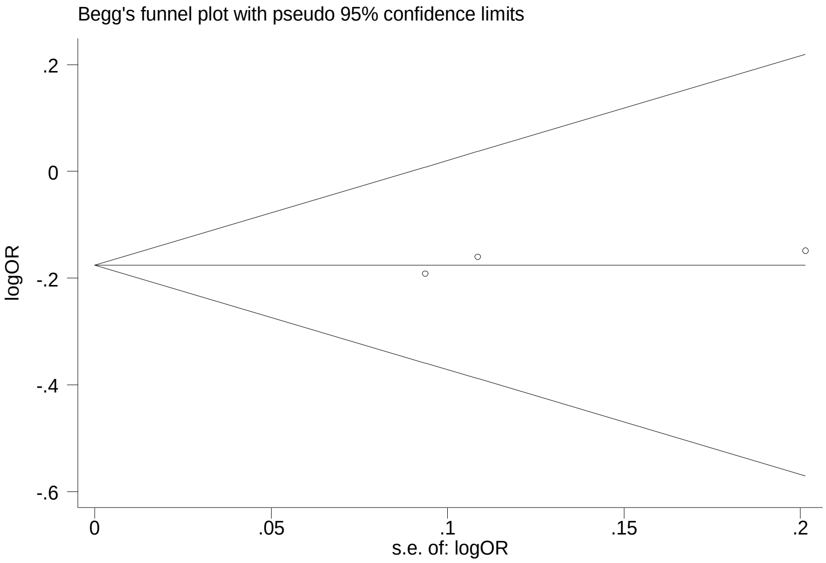

Supplementary Figure S7.4. Main meta-analysis of *CXCR2* rs1126579 (allelic contrast: T vs. C).

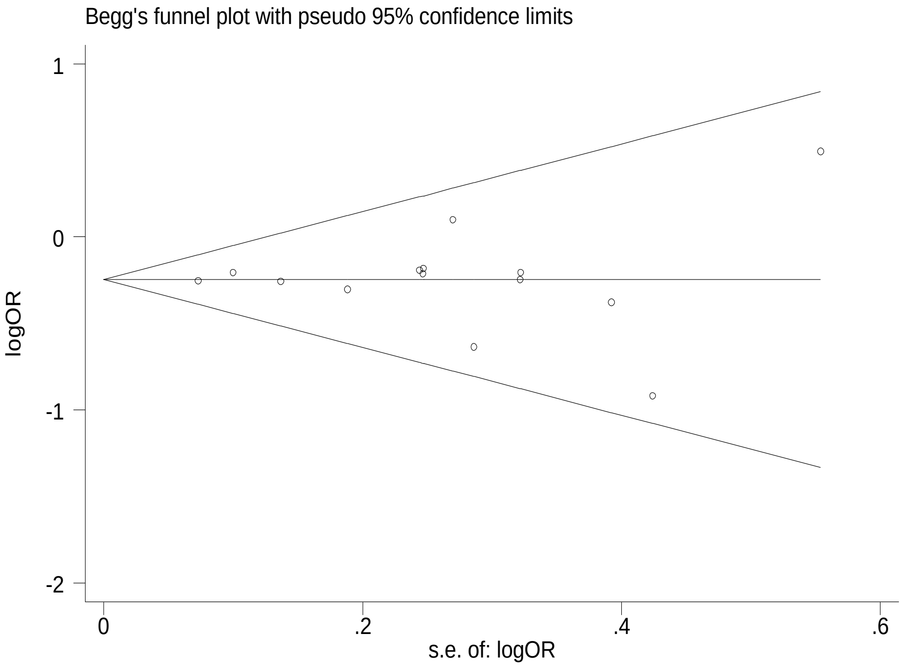

Supplementary Figure S7.5. Main meta-analysis of *CYP2E1* rs6413432 (allelic contrast: A vs. T).

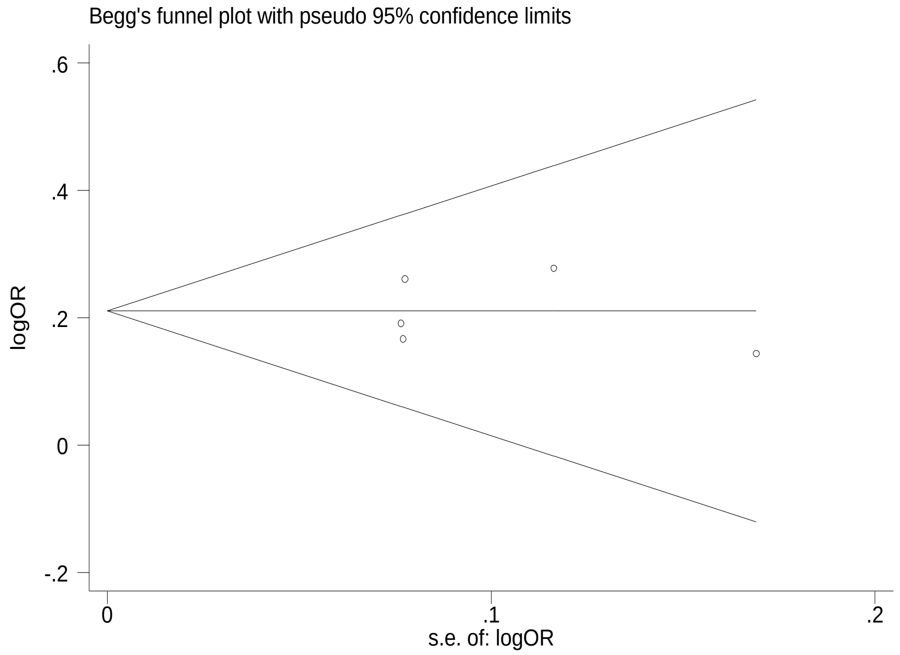

Supplementary Figure S7.6. Main meta-analysis of *HYKK* rs931794 (allelic contrast: G vs. A).

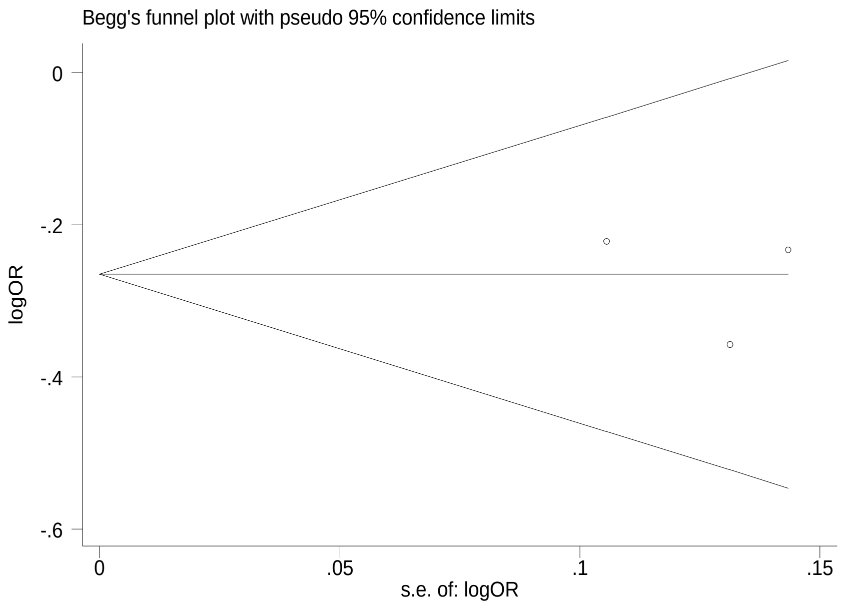

Supplementary Figure S7.7. Main meta-analysis of *PON1* rs662 (allelic contrast: A vs. G).

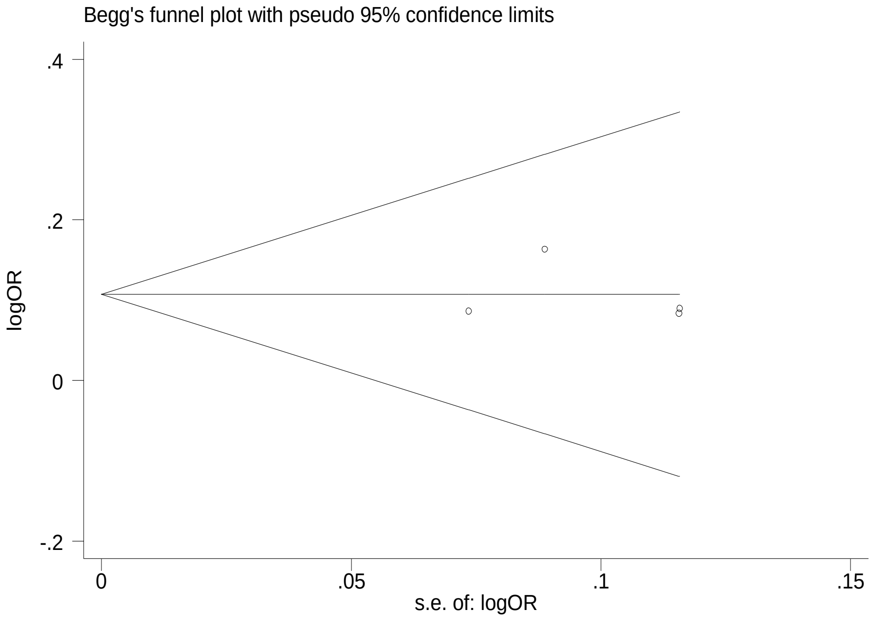

Supplementary Figure S7.8. Main meta-analysis of *REV3L* rs462779 (allelic contrast: T vs. C). (Available data limited to Asian populations.)

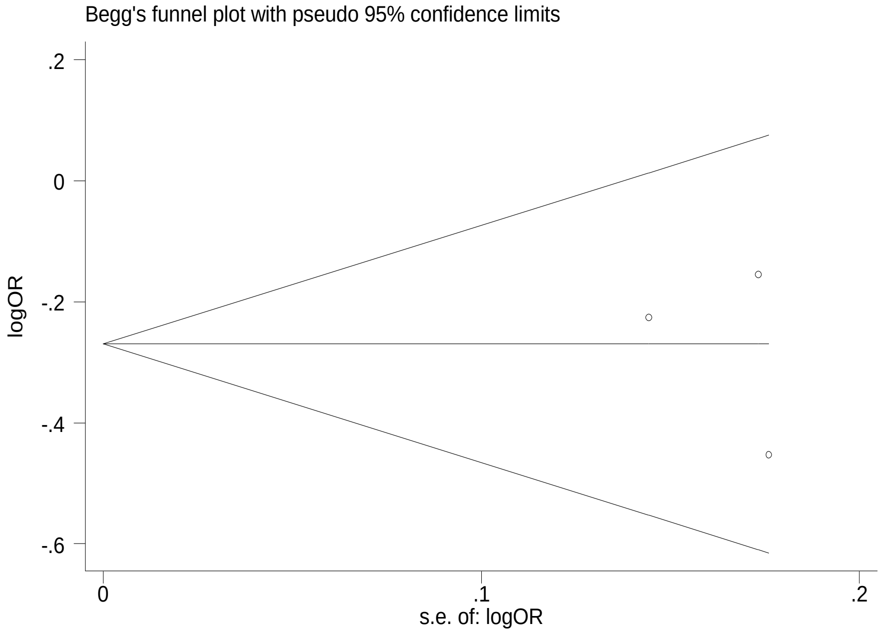

Supplementary Figure S7.9. Meta-analysis of *ATM* rs664677 (Dominant: CC+CT vs. TT).

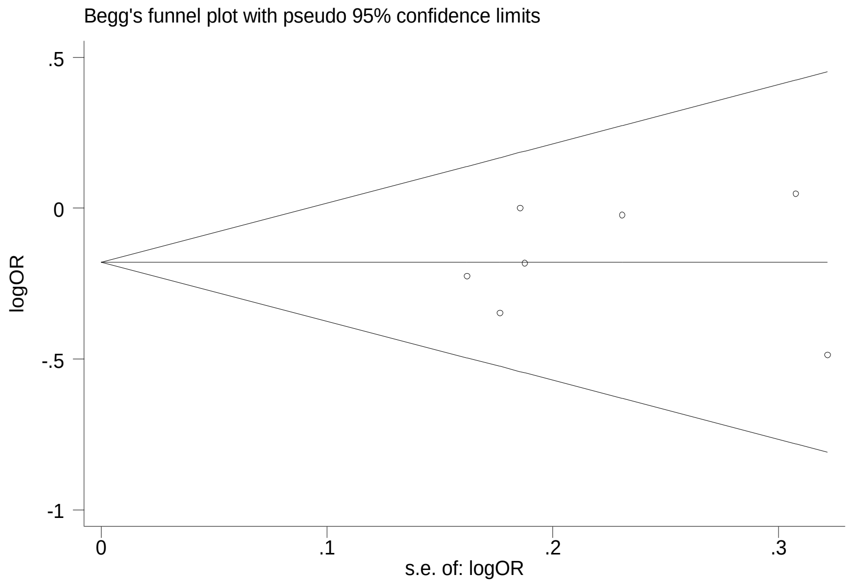

Supplementary Figure S7.10. Subgroup meta-analysis of *APEX1* rs1130409 in Caucasian (Recessive: GG+GT vs. TT).

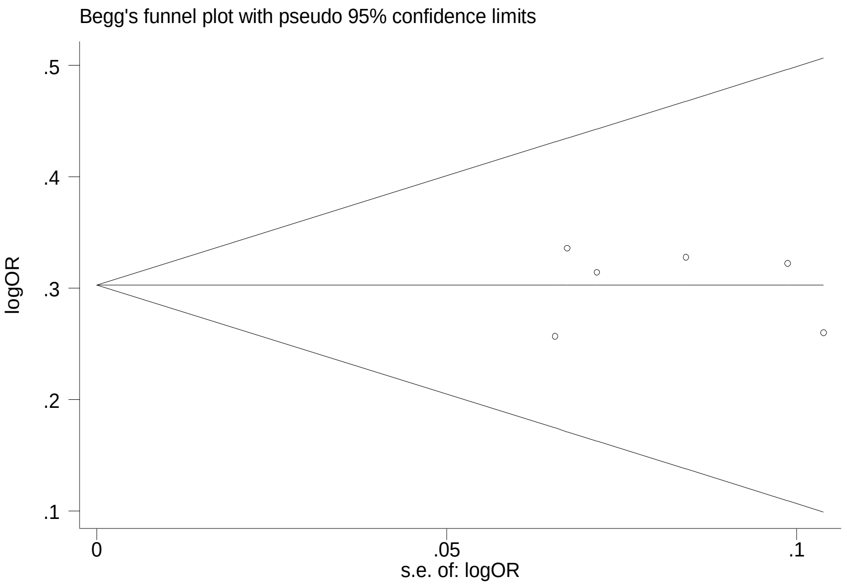

Supplementary Figure S7.11. Subgroup meta-analysis of *CHRNA5* rs16969968 in Caucasian (allelic contrast: A vs. G).

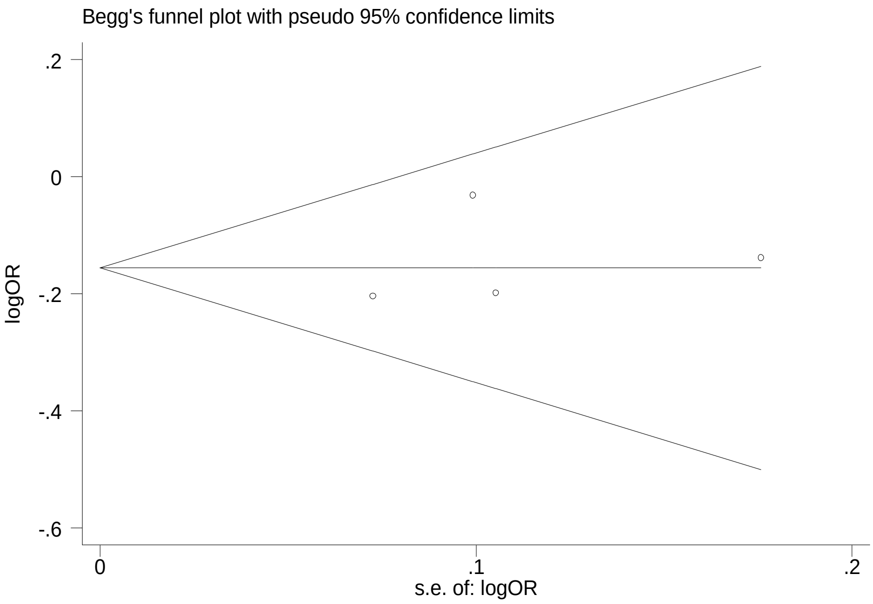

Supplementary Figure S7.12. Subgroup meta-analysis of *CLPTM1L* rs402710 in Caucasian (allelic contrast: T vs. C).

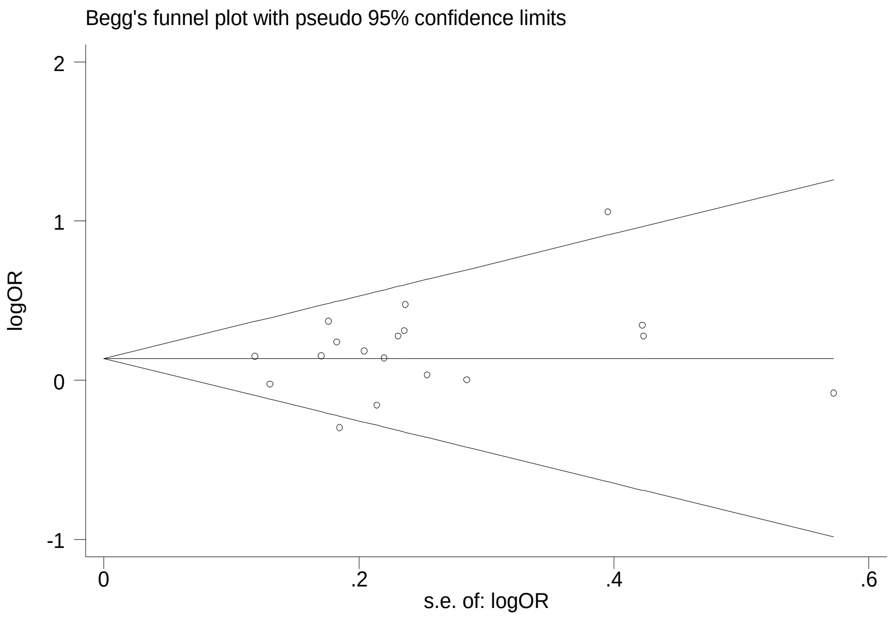

Supplementary Figure S7.13. Subgroup meta-analysis of *ERCC2* rs13181 in Caucasian (Recessive: AA vs. CC+CA).

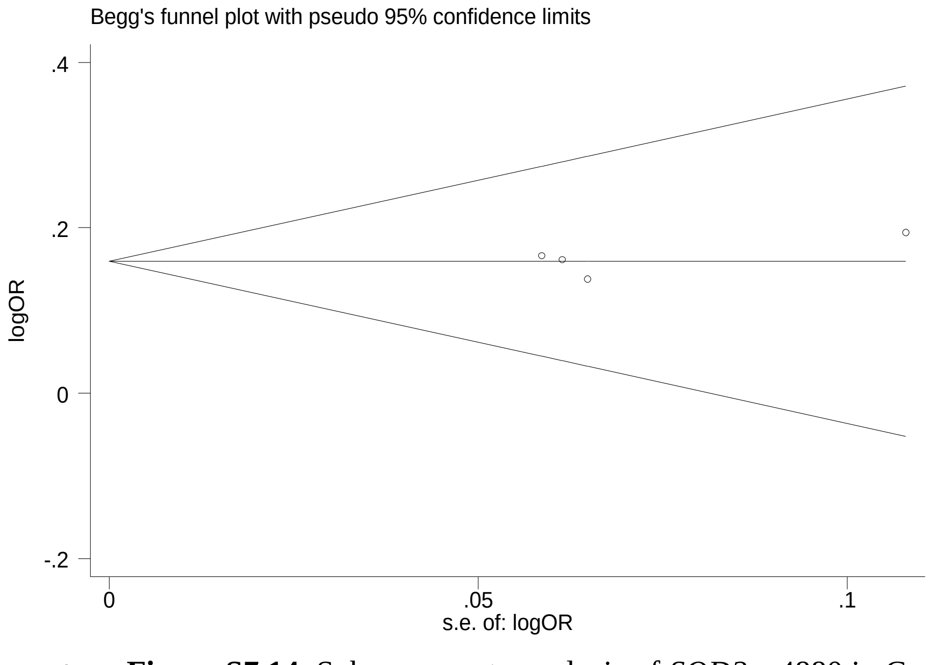

Supplementary Figure S7.14. Subgroup meta-analysis of *SOD2* rs4880 in Caucasian (allelic contrast: T vs. C).

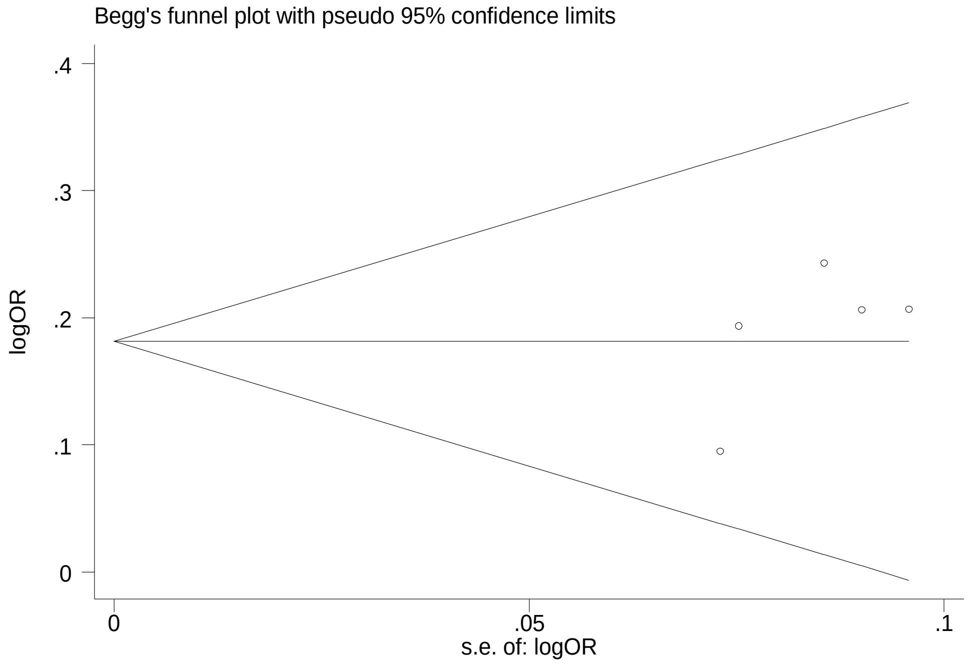

Supplementary Figure S7.15. Subgroup meta-analysis of *APEX1* rs1760944 in Asian (allelic contrast: A vs. C).

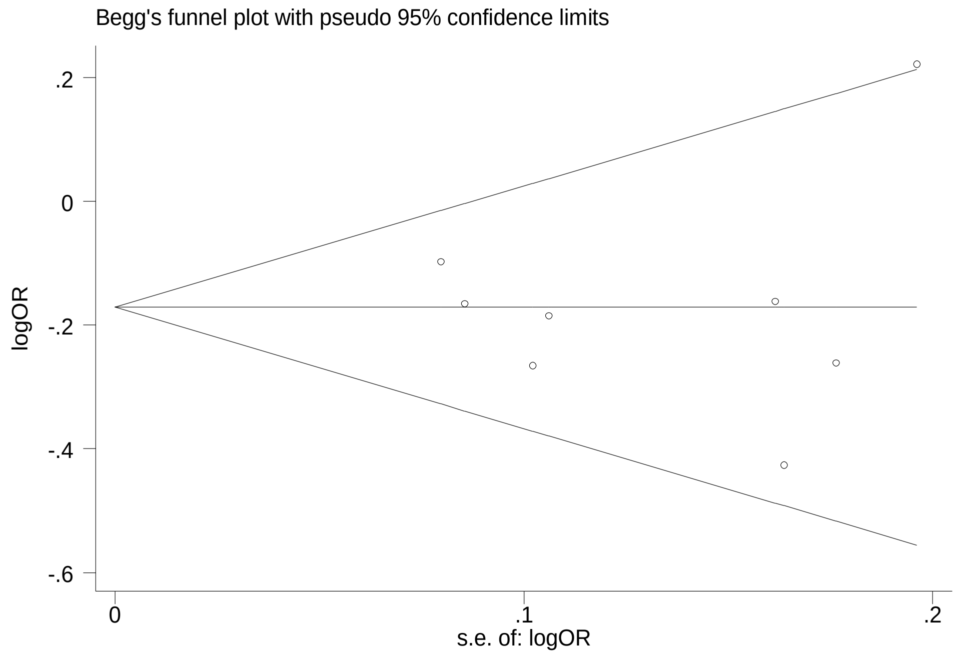

Supplementary Figure S7.16. Subgroup meta-analysis of *CLPTM1L* rs402710 in Asian (Dominant: TT+TC vs. CC).

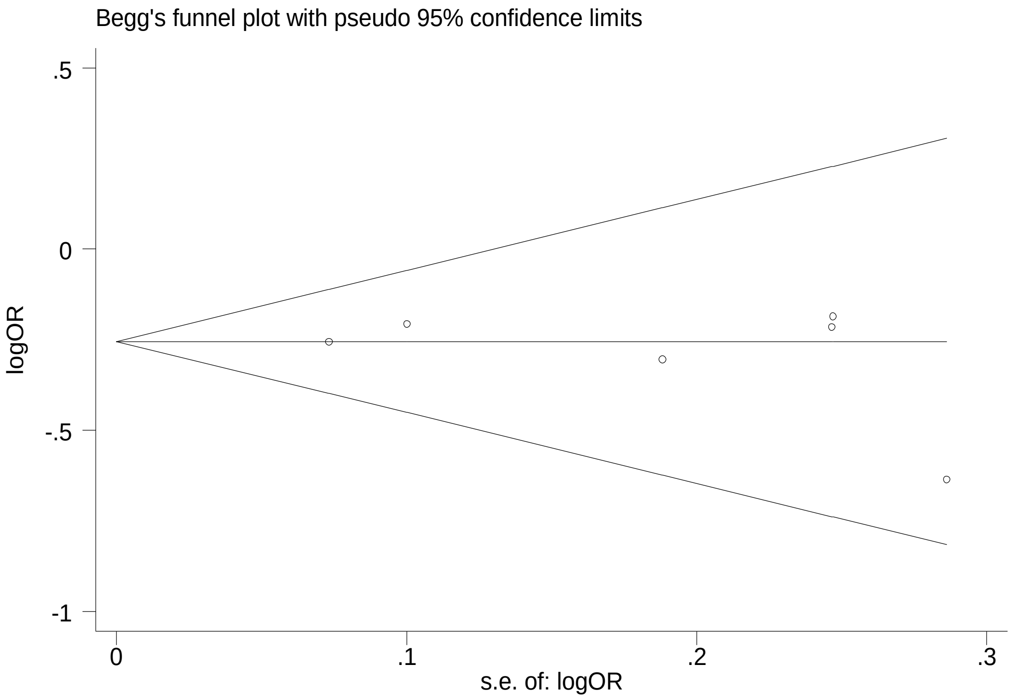

Supplementary Figure S7.17. Subgroup meta-analysis of *CYP2E1* rs6413432 in Asian (allelic contrast: A vs. T).

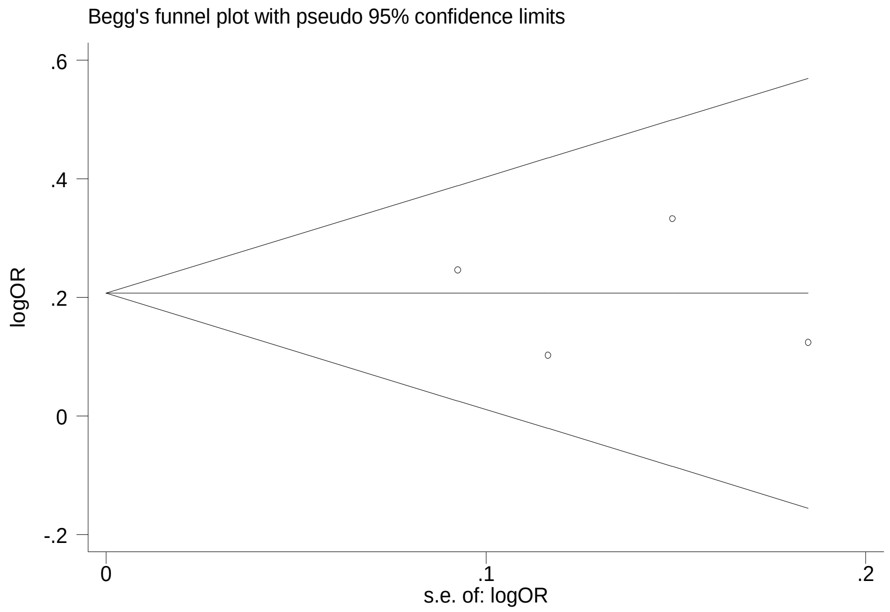

Supplementary Figure S7.18. Subgroup meta-analysis of *MIR146A* rs2910164 in Asian (Recessive: GG vs. CC+CG).

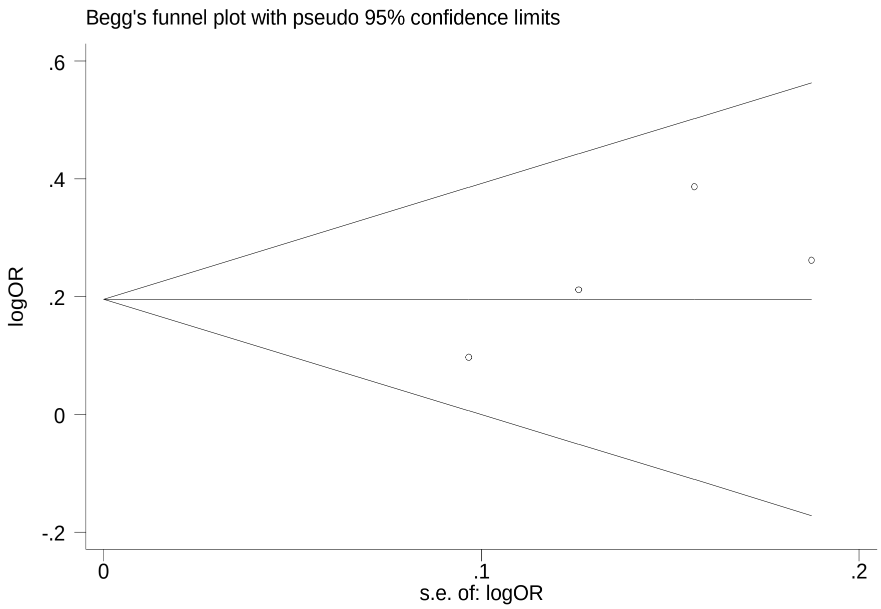

Supplementary Figure S7.19. Subgroup meta-analysis of *MIR196A2* rs11614913 in Asian (Dominant: CC+CT vs. TT).

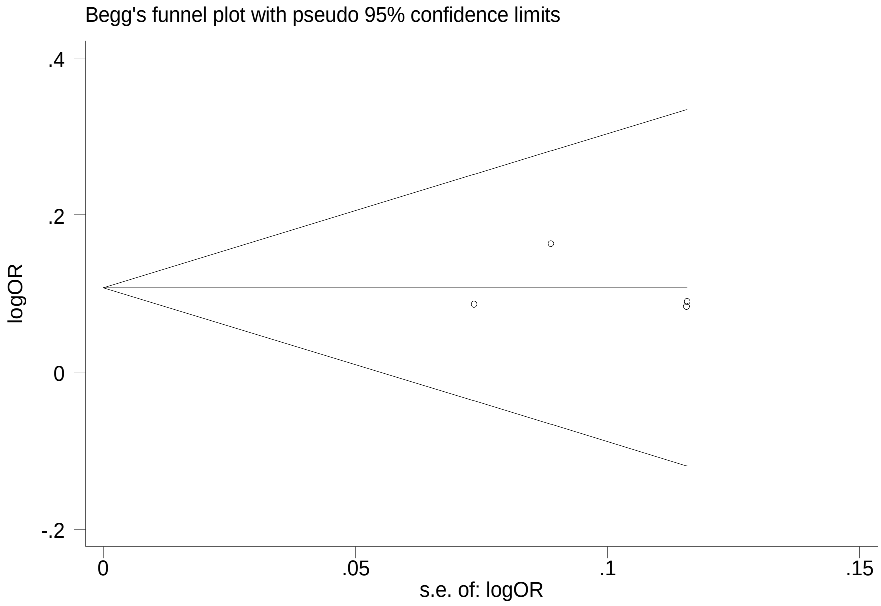

Supplementary Figure S7.20. Subgroup meta-analysis of *REV3L* rs462779 in Asian (allelic contrast: T vs. C).

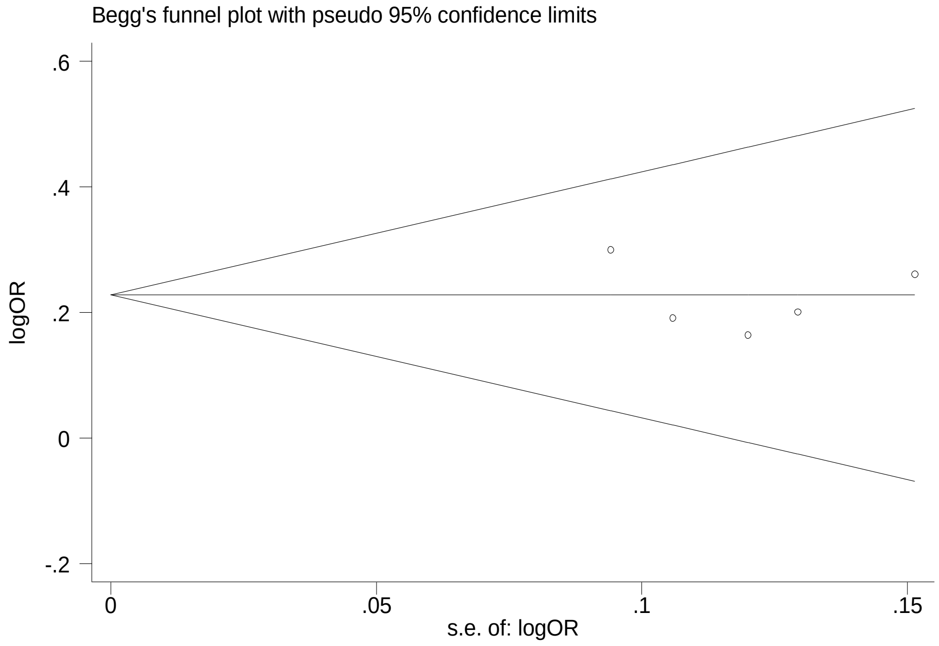

Supplementary Figure S7.21. Subgroup meta-analysis of *TERT* rs2736098 in Asian (Dominant: AA+AG vs. GG).

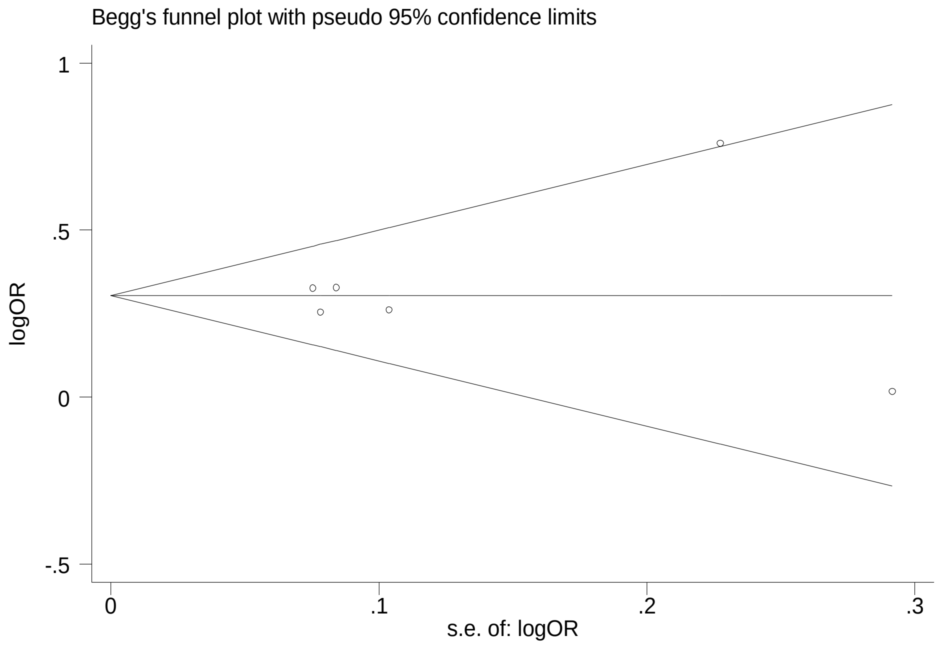

Supplementary Figure S7.22. Subgroup meta-analysis of *CHRNA5* rs16969968 in non-small cell lung cancer population (allelic contrast: A vs. G).

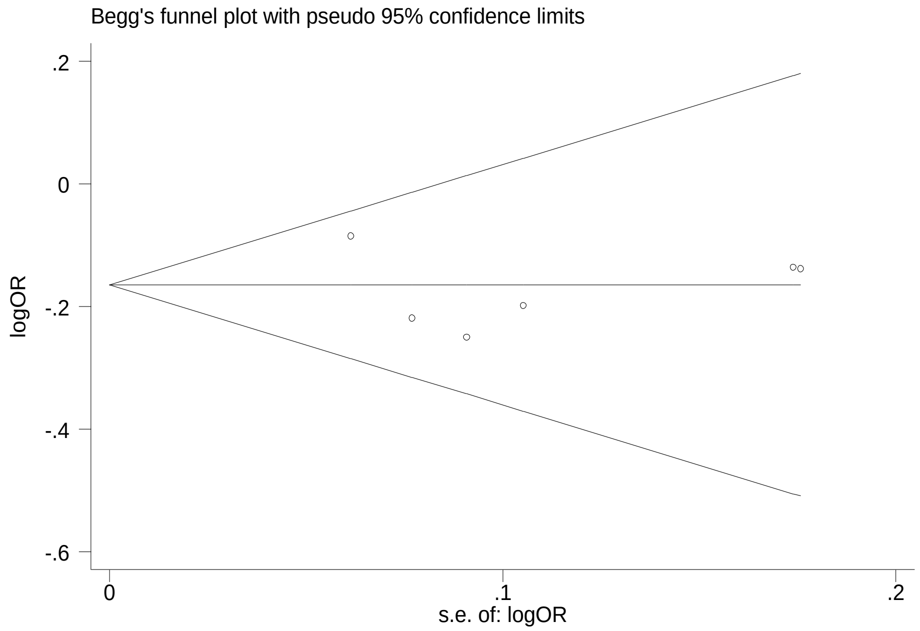

Supplementary Figure S7.23. Subgroup meta-analysis of *CLPTM1L* rs402710 in non-small cell lung cancer population (allelic contrast: T vs. C).

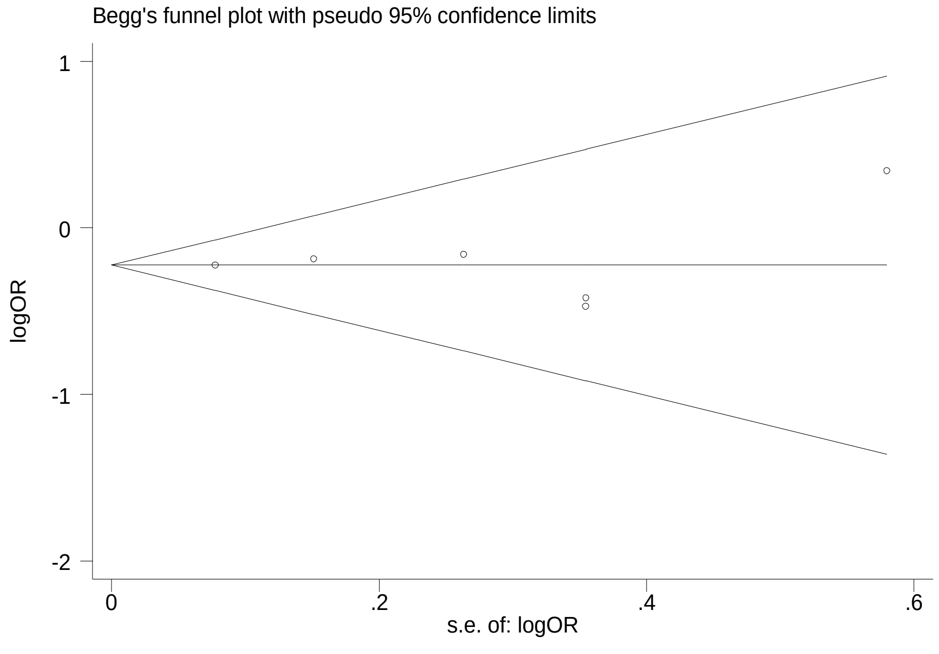

Supplementary Figure S7.24. Subgroup meta-analysis of *CYP2E1* rs6413432 in non-small cell lung cancer population (allelic contrast: A vs. T).

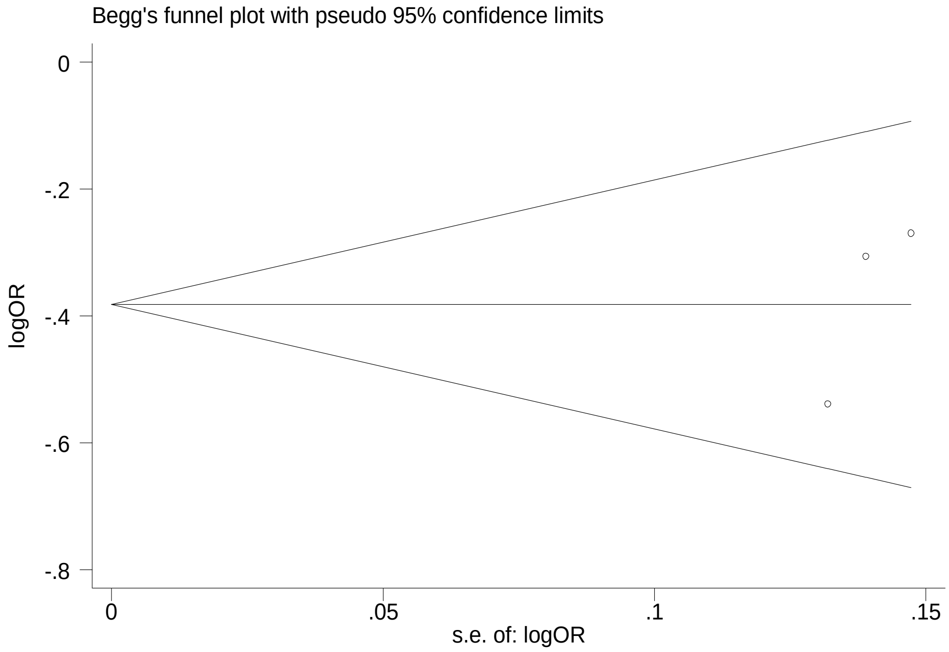

Supplementary Figure S7.25. Subgroup meta-analysis of *ERCC1* rs11615 in non-small cell lung cancer population (allelic contrast: C vs. T).

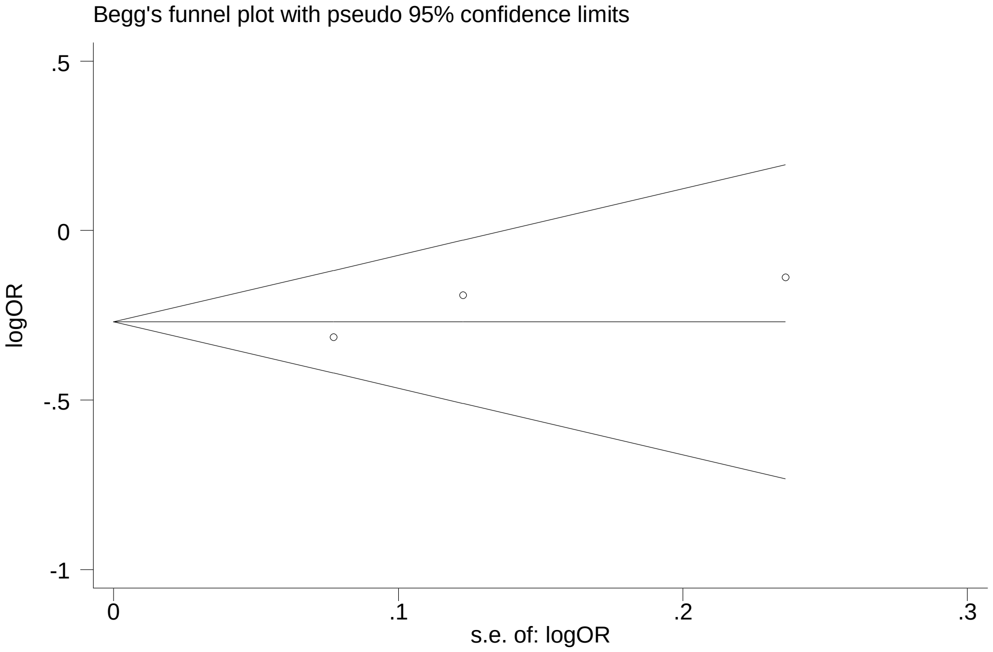

Supplementary Figure S7.26. Subgroup meta-analysis of *FGFR4* rs351855 in non-small cell lung cancer population (allelic contrast: A vs. G).

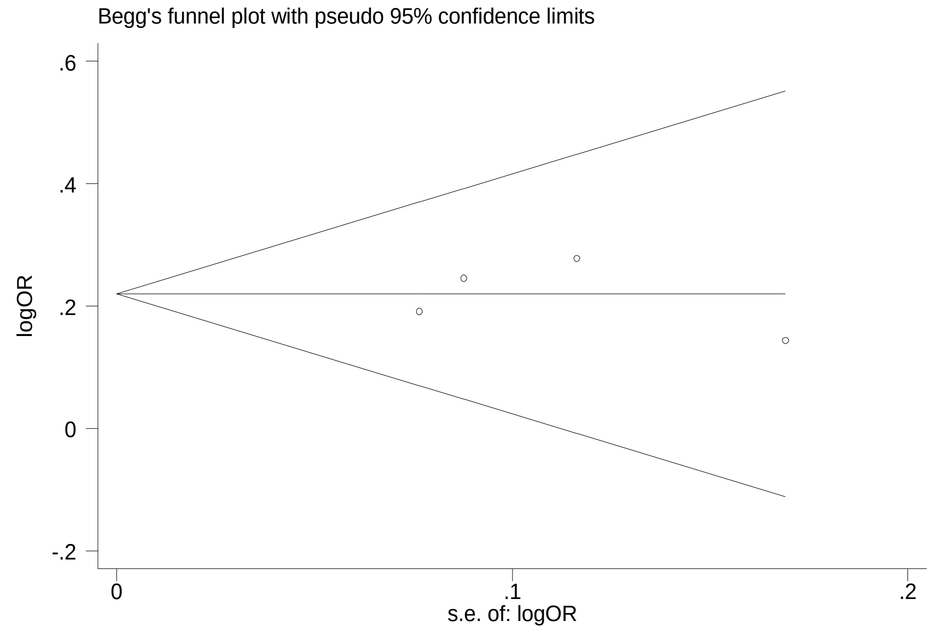

Supplementary Figure S7.27. Subgroup meta-analysis of *HYKK* rs931794 in non-small cell lung cancer population (allelic contrast: G vs. A).

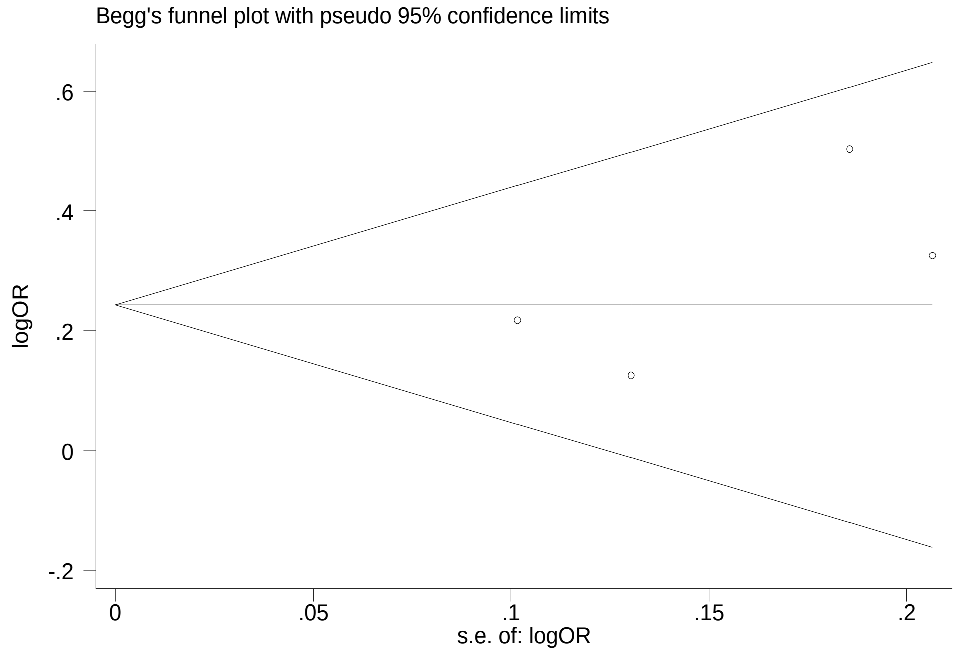

Supplementary Figure S7.28. Subgroup meta-analysis of *MIR146A* rs2910164 in non-small cell lung cancer population (allelic contrast: C vs. G).

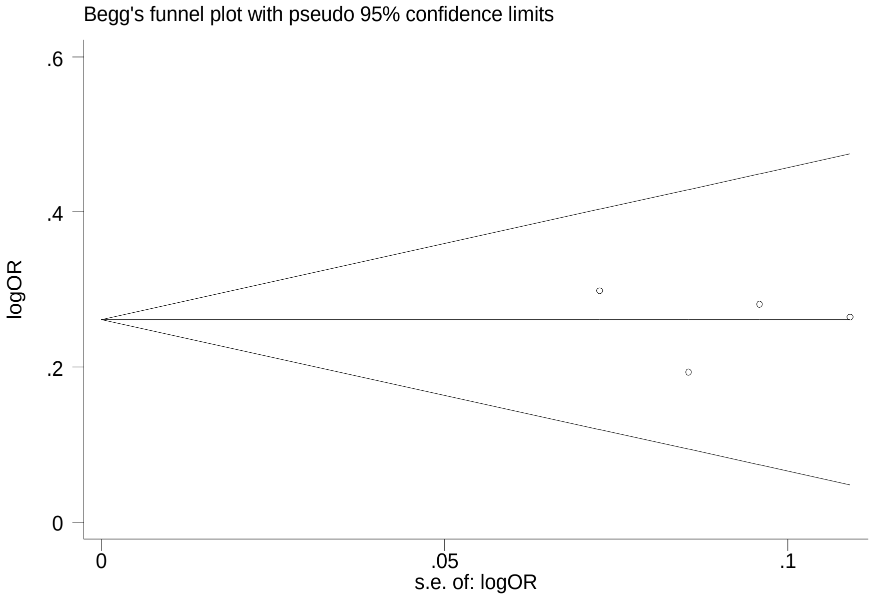

Supplementary Figure S7.29. Subgroup meta-analysis of *TERT* rs2736098 in non-small cell lung cancer population (allelic contrast: A vs. G).

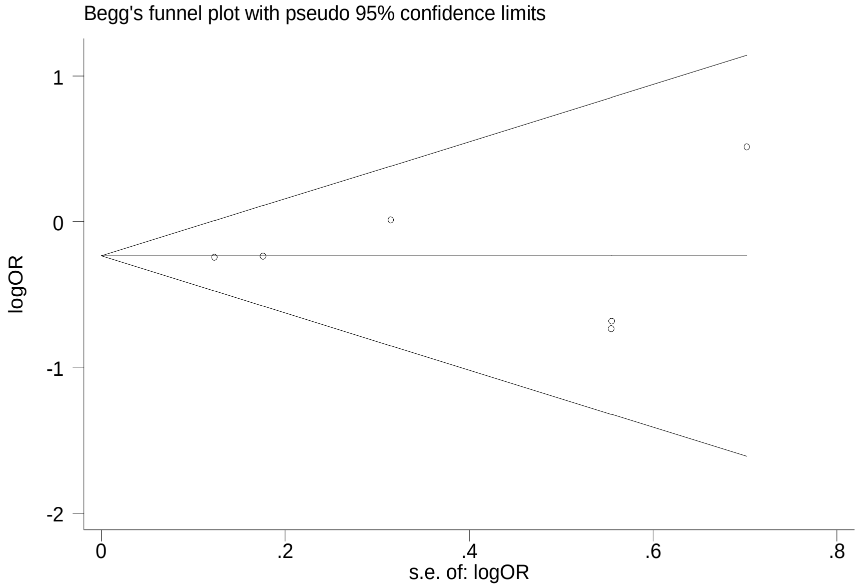

Supplementary Figure S7.30. Subgroup meta-analysis of *CYP2E1* rs6413432 in adenocarcinoma population (allelic contrast: A vs. T).

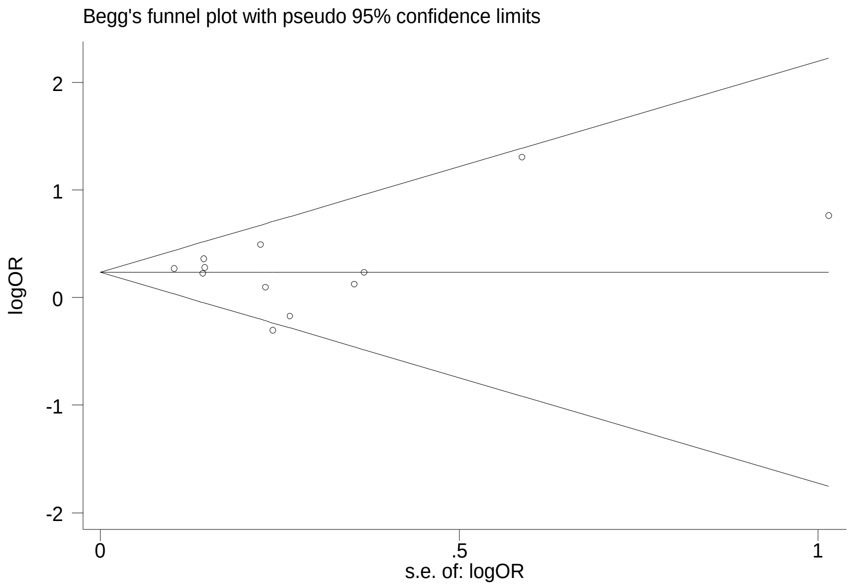

Supplementary Figure S7.31. Subgroup meta-analysis of *OGG1* rs1052133 in adenocarcinoma population (Recessive: CC vs. GG+GC).

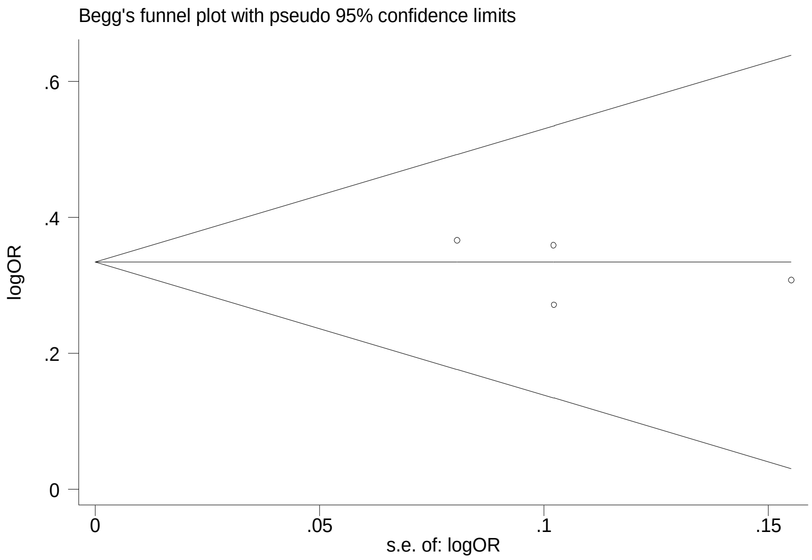

Supplementary Figure S7.32. Subgroup meta-analysis of *TERT* rs2736098 in adenocarcinoma population (allelic contrast: A vs. G).

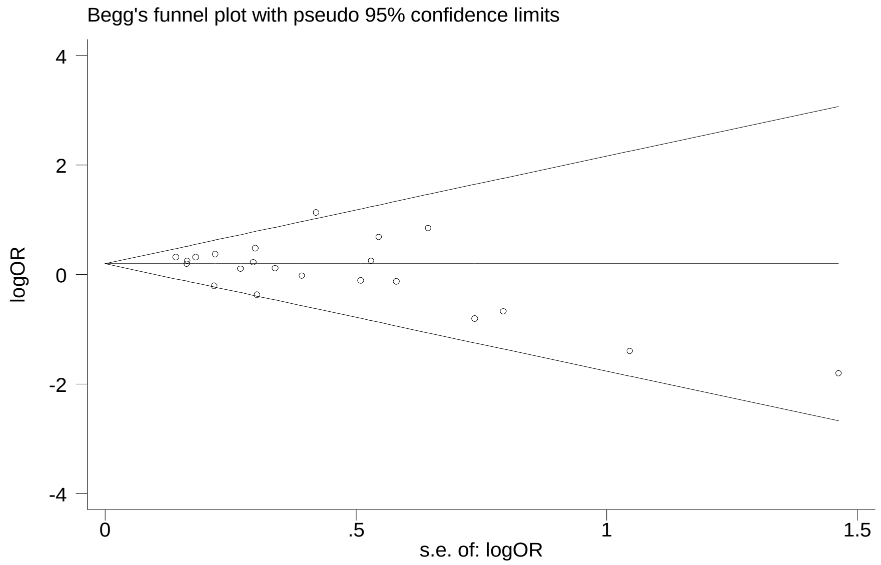

Supplementary Figure S7.33. Subgroup meta-analysis of *TP53* rs1042522 in adenocarcinoma population (Recessive: GG vs. CC+CG).

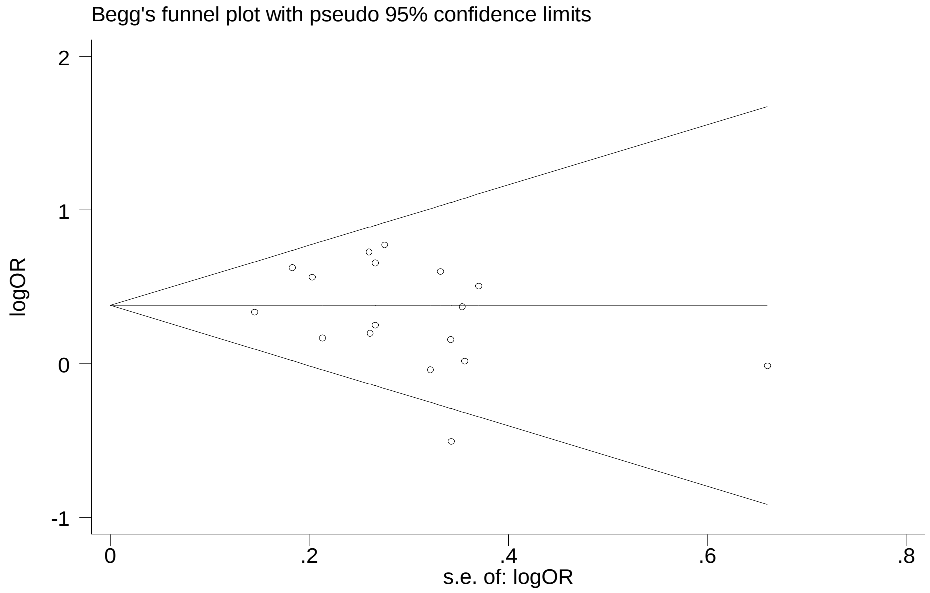

Supplementary Figure S7.34. Subgroup meta-analysis of *CYP1A1* rs4646903 in squamous cell carcinoma population (allelic contrast: C vs. T).

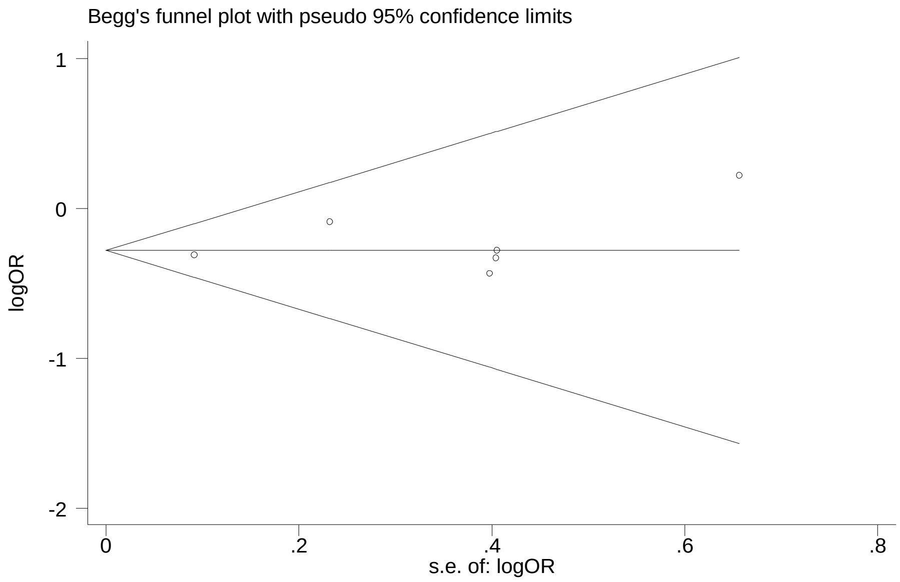

Supplementary Figure S7.35. Subgroup meta-analysis of *CYP2E1* rs6413432 in squamous cell carcinoma population (allelic contrast: A vs. T).

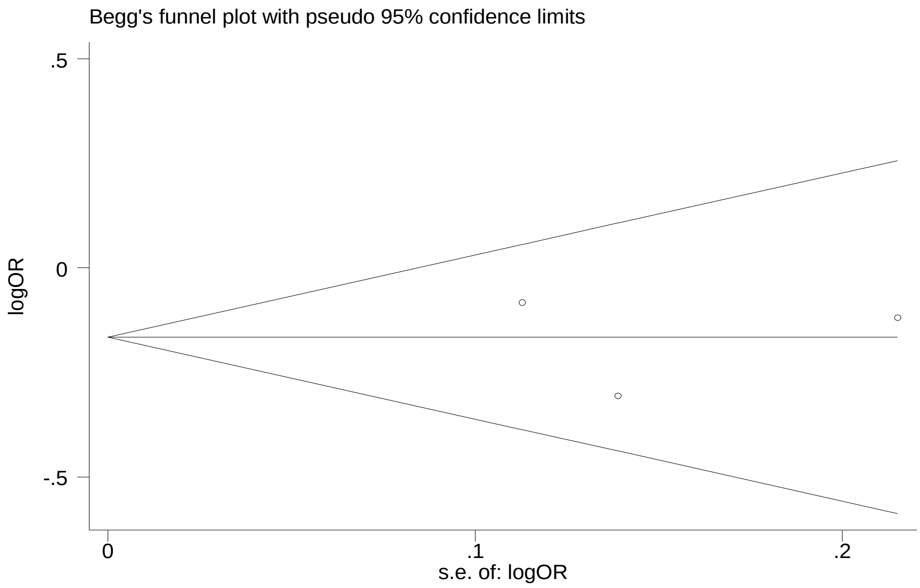

Supplementary Figure S7.36. Subgroup meta-analysis of *ERCC1* rs11615 in non-smokers (allelic contrast: C vs. T).

Supplementary Figure S7. Begg's funnel plots of main or subgroup meta-analyses for variants showed significant associations to lung cancer susceptibility with strong cumulative evidence. Begg's funnel plots were used for publication bias test. Each point represented a separate study. LogOR, natural

logarithm of OR. Horizontal line, mean effect size. The degree of asymmetry of the funnel plots were evaluated by the Begg’s test, and all P-value of Begg’s test were displayed on the respective table in the manuscript.

Supplementary Table S1. Genetic associations with lung cancer risk for all 246 variants analyzed in main meta-analyses under different genetic models (Continued on next three pages)

Gene	Variant	Chr	Category	Ethnicity	Comparison[a]	Frequency(%)[b]	Number evaluated		Lung cancer risk meta-analysis in allelic model								Dominant model			Recessive model		
							Studies	Cases/Controls	OR(95% CI)	*P* value	Q	*P* for Q	I^2(%)	Begg *P*	Modified *P*[c]	Excess *P*[d]	OR(95% CI)	*P* value	I^2(%)	OR(95% CI)	*P* valute	I^2(%)
ABCB1	rs1045642(p.Ile1145Ile,c.3435T>C)	7	synonymous	All	T vs C	39.40	4	1025/1080	1.30(0.79-2.16)	0.307	28.98	0.00	90	0.308	0.535	1.000	1.21(0.67-2.19)	0.535	83	1.34(0.72-2.50)	0.353	79
ACE	287-bp repeat(I/D)	17	STR	All	Ins vs del	49.31	8	1730/8359	1.01(0.85-1.21)	0.885	19.95	0.01	65	0.536	0.627	0.019	1.00(0.76-1.32)	0.985	57	1.00(0.76-1.33)	0.998	66
AGER	rs1800624(-374T>A,Mun1)	6	intergenic	Asian	A vs T	34.41	3	1656/1693	1.18(1.04-1.33)	0.010	2.38	0.31	16	1.000	0.480	1.000	1.34(0.97-1.85)	0.077	72	1.18(0.95-1.46)	0.127	0
AGER	rs1800625(-429T>C,Alu1)	6	intergenic	Asian	C vs T	31.19	3	1656/1693	1.16(0.78-1.74)	0.468	20.99	0.00	91	1.000	0.741	0.232	1.12(0.74-1.68)	0.598	85	1.52(0.52-4.42)	0.443	90
AGER	rs2070600(p.Gly82Ser,c.244G>A)	6	non-synonymous	All	A vs G	31.57	4	2104/2178	1.10(0.93-1.31)	0.270	7.15	0.07	58	0.089	0.003	0.110	1.17(0.81-1.70)	0.408	83	1.15(0.77-1.72)	0.502	57
AHR	rs2066853(Arg554Lys,Ex10+501G>A)	7	non-synonymous	All	A vs G	30.44	5	1749/1925	0.96(0.84-1.10)	0.567	5.82	0.21	31	1.000	0.922	1.000	0.95(0.74-1.22)	0.703	63	0.96(0.78-1.18)	0.699	0
ALDH2	rs671(p.Glu487Lys/Glu504Lys,c.1510G>A)	12	non-synonymous	All	A vs G	24.03	3	1555/2079	1.36(0.74-2.50)	0.322	29.62	0.00	93	1.000	0.724	1.000	1.16(0.72-1.88)	0.539	89	1.10(0.53-2.29)	0.793	63
APEX1	rs1760944(c.-468T>G,-141T>G)	14	5'UTR	All	A vs C	47.94	8	3588/3783	1.16(1.08-1.25)	2.85×10^{-5}	7.70	0.36	9	0.386	0.119	0.287	1.21(1.02-1.44)	0.030	55	1.24(1.11-1.38)	1.54×10^{-4}	0
APEX1	rs1048945(p.Gln51His,c.153G>C)	14	non-synonymous	Caucasian	C vs G	4.72	3	745/816	0.93(0.67-1.31)	0.684	0.30	0.86	0	1.000	0.500	1.000						
APEX1	rs1130409(p.Asp148Glu,c.444T>G)	14	non-synonymous	All	G vs T	42.30	21	6479/8565	1.00(0.93-1.09)	0.922	34.22	0.02	45	1.000	0.985	0.023	1.07(0.96-1.19)	0.224	56	0.97(0.84-1.13)	0.731	56
APEX1	rs2307486(p.Ile64Val,c.190A>G)	14	non-synonymous	All	G vs A	6.97	4	2281/2505	0.82(0.63-1.06)	0.121	6.35	0.10	53	0.089	0.119	0.567						
ATM	rs609429(IVS48+238C>G)	11	intron	All	(GG+GC) vs CC	54.72	3	1490/1501	0.82(0.66-1.03)	0.086	3.02	0.22	34	1.000	0.495	0.515	0.82(0.66-1.03)	0.086	34			
ATM	rs664677(IVS21T-77C)	11	intron	All	C vs T	58.90	3	1627/1641	0.91(0.82-1.00)	0.056	1.82	0.40	0	1.000	0.427	1.000	0.76(0.64-0.92)	0.004	0	0.97(0.85-1.11)	0.680	0
ATM	rs189037(c.-111G>A,−4519G>A)	11	5'UTR	Asian	A vs G	42.68	5	3036/3415	1.09(1.00-1.18)	0.050	5.65	0.23	29	0.806	0.195	1.000	1.10(0.98-1.23)	0.105	9	1.16(1.01-1.33)	0.043	20
ATM	rs664143(IVS34G+60A)	11	intron	All	(GG+GA) vs AA	45.53	3	1465/1465	0.93(0.79-1.10)	0.392	1.61	0.45	0	0.296	0.083	1.000	0.93(0.79-1.10)	0.392	0			
AURKA	rs2273535(p.Phe31Ile,c.91T>A)	20	non-synonymous	Caucasian	A vs T	48.28	3	2301/2069	0.96(0.86-1.06)	0.421	0.63	0.73	0	0.296	0.338	1.000	0.97(0.83-1.14)	0.723	0	0.88(0.57-1.34)	0.547	61
AURKA	rs1047972(p.Ile57Val,c.169A>G)	20	non-synonymous	Caucasian	A vs G	15.75	3	2463/2283	0.96(0.86-1.07)	0.425	1.65	0.44	0	0.296	0.167	1.000	0.97(0.86-1.11)	0.681	0	0.77(0.53-1.11)	0.156	0
AXIN2	rs2240308(p.Pro50Ser,c.148C>T)	17	non-synonymous	All	T vs C	37.40	3	758/742	0.73(0.63-0.85)	6.39×10^{-5}	1.84	0.40	0	1.000	0.120	0.251	0.69(0.56-0.85)	3.98×10^{-4}	0	0.55(0.34-0.89)	0.015	32
BCL2	rs2279115(c.-938C>A)	18	5'UTR	All	A vs C	43.37	5	1847/2367	0.65(0.46-0.91)	0.011	45.33	0.00	91	0.624	0.035	0.481	0.59(0.39-0.90)	0.014	88	0.59(0.41-0.84)	0.004	80
BIRC5	rs9904341(c.-31G>C）	17	5'UTR	All	C vs G	43.56	5	1008/963	1.23(0.80-1.88)	0.352	27.16	0.00	85	0.806	0.925	0.091	1.07(0.65-1.77)	0.783	78	1.75(0.99-3.12)	0.056	70
BRCA2	rs144848(p.Asn372His,c.1114A>C)	13	non-synonymous	All	C vs A	23.94	4	1586/2513	1.00(0.82-1.21)	0.959	6.90	0.08	57	0.734	0.734	1.000						
CASC8	rs1447295(C>A)	8	intron	All	A vs C	14.41	3	1541/2057	1.00(0.88-1.14)	0.991	1.52	0.47	0	1.000	0.441	1.000	0.96(0.80-1.15)	0.658	33	1.43(0.90-2.26)	0.129	0
CASC8	rs6983267(T>G)	8	intron	All	G vs T	44.77	3	1539/1989	1.14(0.95-1.38)	0.166	7.70	0.02	74	0.296	0.223	0.263	1.16(0.81-1.66)	0.409	83	1.22(1.04-1.44)	0.013	0
CASP5	rs523104(p.Leu318Val,c.748C>G)	11	non-synonymous	All	C vs G	36.90	3	758/943	1.12(0.98-1.29)	0.105	1.18	0.55	0	1.000	0.371	1.000	1.09(0.90-1.33)	0.378	0	1.32(0.94-1.86)	0.113	33
CASP8	rs3834129(-652 6N del,-/CTTACT)	2	intergenic	All	Del vs non-del	27.82	4	2737/2696	0.95(0.77-1.17)	0.631	16.45	0.00	82	0.308	0.167	0.275	0.93(0.73-1.17)	0.526	76	0.98(0.72-1.32)	0.890	52
CASP8	rs1045485(p.Asp302His,c.853G>C)	2	non-synonymous	All	C vs G	10.96	4	1054/1259	1.20(0.97-1.49)	0.101	4.02	0.26	25	1.000	0.186	0.460	1.25(1.00-1.56)	0.056	20	0.90(0.42-1.91)	0.781	0
CCND1	rs678653(G>C)	11	3'UTR	All	C vs G	29.98	3	1323/1901	1.03(0.85-1.24)	0.773	5.03	0.08	60	1.000	0.347	1.000	0.99(0.76-1.27)	0.905	62	1.09(0.87-1.35)	0.464	0
CCND1	rs9344(rs603965,p.Pro241Pro,c.723G>A)	11	synonymous	All	A vs G	48.82	11	6058/7315	1.02(0.92-1.12)	0.696	30.90	0.00	68	0.533	0.992	0.007	0.98(0.85-1.12)	0.745	55	1.07(0.90-1.26)	0.460	69
CD3EAP	rs967591(c.-21G>A)	19	5'UTR	All	A vs G	32.09	3	676/726	1.23(1.01-1.49)	0.036	2.56	0.28	22	1.000	0.988	1.000	1.38(0.92-2.07)	0.119	62	1.30(0.95-1.78)	0.105	0
CDKN1A	rs1801270(p.Ser31Arg, c.93C>A, 6829C>A)	6	non-synonymous	All	A vs C	13.18	9	4039/4804	1.04(0.89-1.21)	0.663	16.82	0.03	52	0.029	0.009	0.280	1.08(0.88-1.32)	0.483	61	0.92(0.73-1.16)	0.489	0
CDKN2A	rs3731249(p.Ala148Thr,c.442G>A)	9	non-synonymous	Caucasian	(AA+AG) vs GG	1.75	3	2875/2707	1.38(0.81-2.35)	0.230	4.56	0.10	56	0.296	0.177	1.000	1.38(0.81-2.35)	0.230	56			
Chr8q24	rs16901979(C>A)	8	intergenic	All	A vs C	19.48	3	1534/1992	1.15(0.99-1.35)	0.065	3.29	0.19	39	1.000	0.259	1.000	1.18(1.02-1.37)	0.025	0	1.22(0.80-1.86)	0.352	51
CHRNA3	rs12914385(2594C>T)	15	intron	All	T vs C	35.09	4	5356/2873	1.20(1.01-1.44)	0.044	12.27	0.01	76	0.734	0.353	1.000	1.26(1.00-1.57)	0.047	70	1.34(1.04-1.71)	0.022	49
CHRNA3	rs3743073(A>C)	15	intron	All	G vs T	43.53	3	1391/1500	1.27(0.89-1.83)	0.191	23.05	0.00	91	0.296	0.088	1.000	1.37(0.95-1.98)	0.094	80	1.32(0.72-2.44)	0.368	91
CHRNA3	rs578776(C>T)	15	intron	All	T vs C	31.98	3	1245/2009	0.87(0.77-0.98)	0.018	0.19	0.91	0	1.000	0.969	0.576	0.84(0.72-0.98)	0.026	0	0.84(0.66-1.07)	0.162	0
CHRNA3	rs938682(T>C,T78896547C)	15	intron	All	C vs T	28.37	3	1240/1986	0.86(0.76-0.96)	0.009	1.08	0.58	0	0.296	0.054	1.000						
CHRNA3	rs6495309(4064C>T)	15	5'UTR	All	T vs C	38.44	4	3381/4244	0.83(0.77-0.89)	6.55×10^{-8}	2.78	0.43	0	1.000	0.727	1.000	0.78(0.71-0.86)	9.82×10^{-7}	0	0.81(0.68-0.95)	0.010	37
CHRNA5	rs142774214(rs3841324,-62ins/del,22bp indel)	15	5'UTR	All	Del vs ins	37.67	3	1431/1606	0.92(0.83-1.03)	0.131	2.02	0.36	1	1.000	0.828	1.000	0.96(0.83-1.11)	0.571	0	0.80(0.65-0.98)	0.032	0
CHRNA5	rs16969968(p.Asp398Asn,c.1192G>A)	15	non-synonymous	All	A vs G	32.51	11	6222/62452	1.23(1.06-1.43)	0.007	48.96	0.00	80	0.119	0.131	0.071	1.26(1.01-1.59)	0.045	85	1.49(1.32-1.68)	5.18×10^{-11}	0
CLPTM1L	rs402710(C>T)	5	intron	All	T vs C	32.92	13	7214/8051	0.89(0.83-0.95)	2.63×10^{-4}	19.48	0.08	38	0.669	0.437	0.335	0.84(0.78-0.90)	2.42×10^{-7}	0	0.81(0.71-0.93)	0.002	24
COMT	rs4680(p.Val158Met,c.472G>A)	22	non-synonymous	All	A vs G	29.90	5	1497/1958	1.09(0.84-1.43)	0.517	21.84	0.00	82	0.806	0.920	0.077	1.13(0.87-1.45)	0.366	63	1.12(0.66-1.90)	0.674	77
CRP	rs2808630(T>C)	1	3'UTR	All	C vs T	27.26	3	826/895	0.82(0.68-1.01)	0.056	2.79	0.25	28	1.000	0.859	1.000	0.81(0.61-1.07)	0.135	44	0.71(0.48-1.04)	0.080	0
CTLA4	rs3087243(g.6230G>A,CT60)	2	3'UTR	All	A vs G	26.64	3	1132/1220	0.99(0.86-1.14)	0.887	1.05	0.59	0	0.296	0.158	1.000	1.05(0.88-1.26)	0.566	0	0.81(0.59-1.11)	0.184	0
CTLA4	rs231775(p.Thr17Ala,c.49A>G)	2	non-synonymous	All	G vs A	61.94	6	2828/2953	0.88(0.64-1.21)	0.445	63.12	0.00	92	0.707	0.894	0.024	0.83(0.50-1.38)	0.477	91	0.93(0.66-1.31)	0.682	82
CTLA4	rs5742909(g.319C>T,-318C>T)	2	intergenic	All	T vs C	15.64	4	1205/1349	0.68(0.30-1.56)	0.364	48.66	0.00	94	0.308	0.581	0.634	0.67(0.30-1.50)	0.331	92	0.78(0.13-4.81)	0.791	82
CXCL12	rs1801157(801G>A)	10	3'UTR	All	A vs G	28.71	3	489/815	1.29(0.76-2.18)	0.351	17.39	0.00	89	1.000	0.954	0.277	1.40(0.75-2.60)	0.296	85	1.38(0.56-3.39)	0.483	81
CXCR2	rs1126579(C>T)	2	3'UTR	All	T vs C	55.45	3	942/964	0.84(0.74-0.96)	0.009	0.07	0.97	0	1.000	0.496	1.000	0.72(0.58-0.90)	0.004	0	0.87(0.71-1.06)	0.171	0
CYP17A1	rs743572(T-34C,A1/A2)	10	intergenic	All	A2 vs A1(G vs A)	56.19	4	1245/1689	1.03(0.89-1.19)	0.701	4.80	0.19	38	0.308	0.114	1.000	1.01(0.75-1.35)	0.961	45	1.04(0.84-1.30)	0.703	34
CYP1A1	rs1799814(m4,Thr461Asn,C>A)	15	non-synonymous	All	A vs C	5.97	5	1712/1967	1.22(0.57-2.62)	0.617	51.87	0.00	92	0.806	0.859	0.122	1.40(0.77-2.54)	0.269	90	1.77(0.19-16.66)	0.617	81
CYP1A1	rs1800031(m3,T5639C)	15	3'UTR	All	(CC+CT) vs TT	4.08	3	500/919	1.32(0.83-2.11)	0.248	0.63	0.73	0	0.296	0.146	1.000	1.32(0.83-2.11)	0.248	0			
CYP1A1	rs2606345(IVS1+606T>G)	15	intron	Caucasian	G vs T	34.44	3	2770/3609	1.00(0.91-1.11)	0.937	3.05	0.22	35	1.000	0.998	1.000	1.02(0.89-1.18)	0.786	38	0.99(0.85-1.15)	0.882	0
CYP1A1	rs4646903(m1,MspI,T3801C)	15	3'UTR	All	C vs T	21.88	57	9844/12410	1.16(1.07-1.25)	1.59×10^{-4}	121.62	0.00	55	0.772	0.256	0.014	1.20(1.10-1.30)	1.92×10^{-5}	46	1.30(1.11-1.52)	0.001	40
CYP1A1	rs1048943(*2C,m2,p.Ile462Val,c.1384A>G)	15	non-synonymous	All	G vs A	17.83	54	9869/12114	1.23(1.11-1.36)	7.64×10^{-5}	159.34	0.00	67	0.649	0.081	0.007	1.27(1.13-1.43)	5.60×10^{-5}	65	1.44(1.18-1.77)	4.16×10^{-4}	46
CYP1A2	rs2069514(CYP1A2*1C,-3860G>A)	15	intron	All	A vs G	4.23	5	925/1383	1.55(0.64-3.74)	0.327	32.99	0.00	88	0.221	0.927	1.000	1.28(0.72-2.26)	0.400	78			
CYP1A2	rs2069526(-739T>G)	15	intron	All	G vs T	8.00	3	396/494	1.70(0.77-3.76)	0.189	11.15	0.00	82	1.000	0.858	1.000	1.63(0.92-2.87)	0.095	54			
CYP1A2	rs35694136(-2467delT)	15	5'UTR	All	Carriers vs non-carriers	18.62	4	811/1254	1.16(0.67-2.02)	0.594	11.94	0.01	75	0.734	0.839	0.002	1.16(0.67-2.02)	0.594	75			
CYP1A2	rs762551(CYP1A2*1F,c.A-164C)	15	intron	All	C vs A	31.14	6	1273/1877	1.07(0.82-1.40)	0.616	27.04	0.00	82	0.452	0.491	0.000	1.01(0.72-1.43)	0.947	78	1.27(0.86-1.85)	0.226	67
CYP1B1	rs10012(p.Arg48Gly,c.142C>G)	2	non-synonymous	All	G vs C	25.98	3	622/666	1.46(0.88-2.40)	0.142	15.51	0.00	87	0.296	0.099	1.000	1.69(1.05-2.72)	0.031	74	1.54(0.52-4.53)	0.436	87
CYP1B1	rs1056827(p.Ala119Ser,c.355G>T,m2)	2	non-synonymous	All	T vs G	26.90	5	1501/2245	1.16(0.90-1.50)	0.253	18.67	0.00	79	1.000	0.789	0.289	1.24(0.91-1.69)	0.170	76	1.18(0.69-2.01)	0.549	73
CYP1B1	rs1800440(p.Asn453Ser,Ex3+315A>G)	2	non-synonymous	All	G vs A	18.97	5	3009/3887	0.99(0.85-1.14)	0.836	6.88	0.14	42	0.462	0.318	1.000	0.95(0.80-1.14)	0.579	47	1.23(0.97-1.56)	0.093	0
CYP1B1	rs1056836(CYP1B1*3,p.Leu432Val,c.1294C>G)	2	non-synonymous	All	G vs C	38.50	12	3033/3866	1.13(1.05-1.22)	0.002	9.77	0.55	0	0.064	0.228	0.387	1.17(1.05-1.30)	0.005	0	1.15(1.00-1.31)	0.043	0
CYP2A13	rs8192789(p.Arg257Cys,3375C>T)	19	non-synonymous	All	(TT+TC) vs CC	9.36	3	1517/2231	0.81(0.57-1.15)	0.238	3.54	0.17	43	1.000	0.482	0.384	0.81(0.57-1.15)	0.238	43			
CYP2A6	non*4/*4(*1/*4,a deletion of the CYP2A6 gene)	19	a deletion of gene	All	Del vs non-del	13.48	8	2867/2630	0.81(0.64-1.02)	0.074	23.21	0.00	70	0.711	0.928	0.015	0.88(0.68-1.14)	0.324	71	0.51(0.35-0.73)	2.93×10^{-4}	0
CYP2A6	rs1801272(p.Leu160His,Ex3-15T>A)	19	non-synonymous	Caucasian	(AA+AT) vs TT	3.99	3	2411/2644	0.66(0.52-0.84)	0.001	0.79	0.67	0	1.000	0.929	1.000	0.66(0.52-0.84)	0.001	0			
CYP2A6	rs5031016(p.Ile471Thr,c1412T>C,wt/*7)	19	non-synonymous	All	C vs T	9.89	3	1527/1138	0.57(0.33-1.00)	0.048	7.38	0.03	73	0.296	0.587	1.000	0.58(0.34-0.98)	0.043	66			
CYP2C9	rs1799853(p.Arg144Cys,c.430C>T)	10	non-synonymous	All	(TT+TC) vs CC	12.23	4	756/1293	1.14(0.85-1.55)	0.381	4.57	0.21	34	1.000	0.660	0.240	1.14(0.85-1.55)	0.381	34			
CYP2D6	phenotype(EM/HEM/PM)	22	phenotype	All	PM vs EM	5.38	9	1627/1970	0.80(0.53-1.22)	0.296	9.90	0.27	19	0.348	0.139	1.000	1.07(0.88-1.30)	0.487	27	0.81(0.55-1.20)	0.300	26
CYP2D6	rs1065852(p.Pro34Ser,188C>T)	22	non-synonymous	All	C vs T	43.08	3	581/636	0.86(0.57-1.30)	0.463	9.94	0.01	80	0.296	0.059	0.041	0.86(0.58-1.27)	0.439	55	0.55(0.17-1.85)	0.337	89
CYP2E1	rs2031920(RsaI,c1>c2,-1053C>T)	10	intergenic	All	T vs C (c2 vs c1)	17.33	23	4983/6628	0.86(0.76-0.97)	0.018	44.31	0.00	50	0.509	0.250	0.309	0.87(0.76-0.99)	0.030	44	0.91(0.60-1.37)	0.638	55
CYP2E1	rs3813867(g.3739G>C,c.-1293G>C)	10	intergenic	All	C vs G (c2 vs c1)	13.17	4	893/1029	0.94(0.56-1.56)	0.803	8.50	0.04	65	0.734	0.480	0.008	0.85(0.61-1.19)	0.348	28	1.06(0.34-3.34)	0.919	59
CYP2E1	rs6413432(c.967+1143T>A,7632T>A)	10	intron	All	A vs T(variation vs wild)	22.17	14	2944/3347	0.78(0.71-0.85)	6.76×10^{-8}	8.34	0.82	0	0.827	0.999	0.541	0.78(0.69-0.88)	3.12×10^{-5}	8	0.65(0.47-0.90)	0.010	16
CYP3A4	rs2740574(-392A>G,*1A/*1B)	7	intergenic	All	G vs A	21.71	4	1283/956	1.20(0.73-1.97)	0.465	9.14	0.03	67	1.000	0.153	0.362	1.13(0.89-1.42)	0.319	0	0.99(0.38-2.59)	0.981	36
CYP3A5	rs776746(A6986G,CYP3A5*3,22892A>G)	7	intron	All	A vs G	25.62	4	1166/929	0.65(0.36-1.16)	0.144	25.41	0.00	88	0.308	0.220	1.000	0.71(0.47-1.07)	0.099	77	0.85(0.28-2.56)	0.773	81
DRD2	rs1079597(G>A,TaqI-)	11	intron	All	A vs G	20.43	3	393/509	1.09(0.86-1.38)	0.499	1.53	0.47	0	0.296	0.436	1.000	1.01(0.78-1.30)	0.957	0	1.35(0.47-3.91)	0.582	56
DRD2	rs1800497(p.Glu714Lys,A2/A1,C>T)	11	non-synonymous	All	A2 vs A1(C vs T)	32.13	3	451/607	1.05(0.85-1.29)	0.667	0.48	0.79	0	1.000	0.611	1.000	1.06(0.79-1.42)	0.691	0	0.97(0.71-1.32)	0.833	0
EGF	rs4444903(+61A>G)	4	5'UTR	All	G vs A	59.28	3	666/690	1.54(0.93-2.57)	0.096	16.52	0.00	88	0.296	0.218	1.000	2.07(1.01-4.24)	0.048	79	1.47(0.84-2.59)	0.180	78
ELANE	rs351107(-903T>G,Rep_a)	19	5'UTR	Caucasian	G vs T	5.31	3	745/762	0.55(0.34-0.87)	0.011	2.80	0.25	29	1.000	0.794	0.565	0.54(0.31-0.94)	0.030	45			

Gene	Variant	Chr	Category	Ethnicity	Comparison[a]	Frequency(%)[b]	Number evaluated		Lung cancer risk meta-analysis in allelic model								Dominant model			Recessive model		
							Studies	Cases/Controls	OR(95% CI)	*P* value	Q	*P* for Q	I^2(%)	Begg *P*	Modified *P*[c]	Excess *P*[d]	OR(95% CI)	*P* value	I^2(%)	OR(95% CI)	*P* valute	I^2(%)
ELANE	rs7254054(-741G>A,REP_b)	19	5'UTR	Caucasian	A vs G	27.20	3	754/750	0.77(0.61-0.97)	0.030	3.72	0.16	46	0.296	0.022	0.567	0.77(0.63-0.95)	0.014	0	0.50(0.18-1.38)	0.183	81
EPHX1	rs1051740(p.Tyr113His,c.337T>C)	1	non-synonymous	All	C vs T	31.81	21	3461/7217	1.05(0.89-1.24)	0.564	106.98	0.00	81	0.057	0.153	0.000	1.05(0.86-1.28)	0.633	77	1.01(0.79-1.28)	0.951	61
EPHX1	rs2234922(p.His139Arg,c.416A>G)	1	non-synonymous	All	G vs A	18.85	23	5582/9556	1.12(0.99-1.28)	0.076	69.15	0.00	68	0.051	0.032	0.289	1.15(1.00-1.33)	0.059	65	1.09(0.88-1.35)	0.428	18
ERCC1	rs11615(rs3177700,p.Asn118Asn,c.354T>C)	19	synonymous	All	C vs T	51.18	12	5731/7058	0.90(0.83-0.99)	0.023	22.97	0.02	52	0.086	0.072	0.434	0.86(0.73-1.00)	0.048	51	0.89(0.80-1.00)	0.051	31
ERCC1	rs16979802(15310G>C)	19	intron	Caucasian	C vs G	21.81	3	1311/2114	0.87(0.74-1.03)	0.102	0.87	0.65	0	1.000	0.444	1.000	0.91(0.73-1.13)	0.399	0	0.75(0.45-1.26)	0.281	15
ERCC1	rs2298881(262G>T,C>A)	19	intron	All	T vs G	29.82	4	3049/3791	0.99(0.91-1.07)	0.723	3.50	0.32	14	0.308	0.727	1.000	0.96(0.86-1.07)	0.439	0	1.02(0.88-1.18)	0.799	0
ERCC1	rs3212948(C>G)	19	intron	All	G vs C	56.25	5	2144/2544	1.04(0.89-1.22)	0.591	10.24	0.04	61	0.462	0.568	0.243	0.95(0.81-1.11)	0.523	3	1.13(0.94-1.36)	0.181	34
ERCC1	rs3212955(A>G)	19	intron	All	G vs A	26.05	4	2271/2845	1.08(0.98-1.18)	0.109	2.42	0.49	0	0.734	0.744	1.000	1.06(0.94-1.20)	0.350	10	1.21(0.96-1.53)	0.107	9
ERCC1	rs3212961(IVS5+33C＞A,17677C>A)	19	intron	All	A vs C	35.50	6	2207/2311	0.94(0.86-1.03)	0.167	4.90	0.43	0	1.000	0.704	1.000	0.93(0.81-1.07)	0.321	0	0.97(0.74-1.28)	0.848	37
ERCC1	rs3212986(8092C>A,14443C>A)	19	3'UTR	All	A vs C	26.25	5	4079/4475	1.00(0.94-1.08)	0.931	1.63	0.80	0	0.806	0.929	1.000	1.01(0.93-1.10)	0.846	0	0.99(0.84-1.17)	0.894	0
ERCC1	rs735482(p.Lys261Thr,A>C)	19	non-synonymous	All	C vs A	19.32	5	1682/2797	1.02(0.91-1.15)	0.709	4.34	0.36	8	0.462	0.490	1.000	1.02(0.85-1.22)	0.830	25	1.14(0.86-1.51)	0.359	0
ERCC2	rs1052555(Asp711Asp,G>A)	19	synonymous	All	A vs G	18.91	3	511/751	1.09(0.85-1.40)	0.493	2.95	0.23	32	0.296	0.145	1.000						
ERCC2	rs171140(A>C)	19	intron	All	C vs A	34.69	3	511/751	1.04(0.87-1.26)	0.645	0.61	0.74	0	1.000	0.716	1.000						
ERCC2	rs13181(p.Lys751Gln,c.2251A>C)	19	non-synonymous	All	C vs A	25.26	40	13111/16749	1.12(1.05-1.19)	4.18×10^{-4}	76.17	0.00	49	0.753	0.272	0.048	1.16(1.08-1.26)	1.26×10^{-4}	40	1.15(1.01-1.30)	0.034	41
ERCC2	rs1799787(IVS19-70C＞T,G>A)	19	intron	All	T vs C	21.34	6	1792/2807	1.03(0.83-1.27)	0.824	10.74	0.06	54	1.000	0.996	0.171	1.02(0.81-1.28)	0.872	49	1.07(0.61-1.88)	0.807	25
ERCC2	rs1799793(p.Asp312Asn,c.981G>A)	19	non-synonymous	All	A vs G	23.17	26	10209/12967	1.02(0.95-1.09)	0.637	43.81	0.01	43	0.628	0.578	0.182	1.02(0.96-1.09)	0.498	1	1.10(0.97-1.25)	0.129	14
ERCC2	rs238406(p.Arg156Arg,C>A)	19	synonymous	All	A vs C	40.05	6	1754/2688	1.12(1.02-1.23)	0.013	3.94	0.56	0	0.260	0.332	0.599	1.08(0.93-1.25)	0.303	11	1.30(1.10-1.52)	0.001	0
ERCC4	rs1799801(p.Ser835Ser,Ex11-247T＞C)	16	synonymous	All	C vs T	27.99	3	1009/1758	1.02(0.88-1.17)	0.835	2.16	0.34	8	1.000	0.786	1.000						
ERCC4	rs1800067(Arg415Gln)	16	non-synonymous	Caucasian	A vs G	6.78	5	1521/2420	1.10(0.92-1.32)	0.286	1.19	0.88	0	0.806	0.952	1.000	1.12(0.92-1.37)	0.261	0	1.63(0.51-5.22)	0.413	20
ERCC5	rs1047768(p.His46His,335C>T)	13	synonymous	All	T vs C	43.99	4	1449/2248	0.86(0.74-1.00)	0.049	5.78	0.12	48	0.734	0.414	1.000	0.92(0.80-1.06)	0.256	0	0.76(0.61-0.94)	0.013	32
ERCC5	rs17655(p.Asp1104His,c.3310G>C)	13	non-synonymous	All	C vs G	39.06	10	3528/4481	0.96(0.84-1.10)	0.525	29.83	0.00	70	1.000	0.952	0.087	0.96(0.77-1.21)	0.754	72	0.90(0.72-1.12)	0.352	53
ERCC6	rs3793784(-6530C>G)	10	5'UTR	All	G vs C	30.82	3	1643/1689	0.75(0.60-0.92)	0.007	6.23	0.04	68	1.000	0.550	0.167						
ERCC6	rs2228528(p.Gly399Asp,G1275A)	10	non-synonymous	All	T vs C	41.74	4	1856/2059	1.05(0.95-1.15)	0.365	2.91	0.41	0	0.734	0.556	1.000						
EXO1	rs1047840(p.Glu589Lys,c.1765G>A)	1	non-synonymous	All	A vs G	21.78	3	1114/1166	1.23(0.96-1.57)	0.099	6.15	0.05	68	1.000	0.981	0.300	1.23(0.85-1.79)	0.274	78	1.33(0.94-1.88)	0.105	0
FANCG	rs2237857(p.Thr297Ile,C1382T)	9	non-synonymous	All	T vs C	8.77	3	1553/1774	0.98(0.68-1.41)	0.903	4.52	0.10	56	1.000	0.330	0.100						
FAS	rs2234767(-1377G/A)	10	5'UTR	All	A vs G	27.07	5	4125/3696	1.09(0.97-1.22)	0.153	7.90	0.10	49	1.000	0.777	0.171	1.05(0.93-1.18)	0.480	24	1.30(0.99-1.73)	0.063	58
FASLG	rs763110(-844C/T)	1	5'UTR	All	T vs C	34.01	4	4004/3639	0.83(0.65-1.07)	0.156	34.08	0.00	91	0.734	0.536	0.630	0.80(0.56-1.14)	0.213	92	0.83(0.70-0.99)	0.038	30
FGFR4	rs351855(p.Gly388Arg,G>A)	5	non-synonymous	All	A vs G	42.47	4	1083/1275	0.82(0.69-0.98)	0.025	4.48	0.21	33	0.089	0.015	1.000	0.73(0.61-0.87)	3.56×10^{-4}	0	0.87(0.58-1.32)	0.521	51
GPC5	rs2352028(C>T)	13	intron	Asian	T vs C	21.65	3	1648/2002	0.97(0.87-1.09)	0.611	1.43	0.49	0	1.000	0.765	1.000						
GPX1	rs1050450(p.Pro200Leu,c.599C>T)	3	non-synonymous	Caucasian	T vs C	34.06	4	976/1339	1.04(0.75-1.44)	0.811	16.02	0.00	81	0.734	0.934	0.157	1.11(0.72-1.71)	0.632	80	0.93(0.54-1.59)	0.785	71
GSTM1	Present/Null(Large gene deletion)	1	phenotype	All	Null vs present	48.85	133	33253/37867	1.18(1.12-1.23)	2.54×10^{-11}	269.09	0.00	52	0.105	0.037	0.011						
GSTM3	rs1799735(c.468+21delAGG,3bp deletion,*A/*B)	1	intron	All	Del vs non-del	17.64	8	1749/2069	0.97(0.86-1.09)	0.608	3.43	0.84	0	0.902	0.668	1.000	0.95(0.84-1.09)	0.483	0	0.90(0.50-1.63)	0.728	54
GSTP1	rs1138272(p.Ala114Val,c.341C>T)	11	non-synonymous	Caucasian	T vs C	7.63	6	1479/1573	1.24(0.93-1.65)	0.149	10.60	0.06	53	1.000	0.529	0.177	1.18(0.90-1.55)	0.236	63	1.56(0.51-4.77)	0.438	39
GSTP1	rs1695(p.Ile105Val,c.313A>G)	11	non-synonymous	All	G vs A	30.41	46	12521/14411	1.08(1.02-1.15)	0.011	100.57	0.00	55	0.075	0.009	0.008	1.08(1.00-1.16)	0.059	55	1.11(1.01-1.22)	0.035	13
GSTT1	Present/Null(Large gene deletion)	22	phenotype	All	Null vs present	26.14	77	23009/25365	1.10(1.02-1.19)	0.011	179.38	0.00	58	0.346	0.345	0.000						
HIF1A	rs11549465(p.Pro582Ser,c.1744C>T)	14	non-synonymous	All	T vs C	13.60	3	509/566	1.19(0.50-2.86)	0.690	18.09	0.00	89	1.000	0.488	0.293	1.19(0.51-2.76)	0.688	86			
HIF1A	rs11549467(p.Ala588Thr,c.1762G>A)	14	non-synonymous	All	A vs G	9.45	3	509/566	2.27(1.74-2.96)	1.62×10^{-9}	1.47	0.48	0	0.296	0.261	0.610	2.15(1.56-2.95)	2.51×10^{-6}	0			
HRAS1	common alleles /rare alleles(HRAS1 VNTR alleles, loss of heterozygosity in the 15p15.5 region)	11	combined variant	Caucasian	rare vs common	7.03	4	746/1174	2.55(1.01-6.45)	0.048	9.56	0.02	69	0.734	0.561	0.346						
HYKK	rs931794(A>G)	15	intron	All	G vs A	32.89	5	2435/3180	1.23(1.14-1.34)	1.85×10^{-7}	1.28	0.86	0	1.000	0.967	0.411	1.29(1.15-1.43)	6.08×10^{-6}	0	1.35(1.16-1.58)	1.58×10^{-4}	0
IFNG	rs2069705 (-1615T>C)	12	intergenic	All	C vs T	38.30	3	2119/2654	1.02(0.93-1.11)	0.727	2.01	0.37	0	1.000	0.662	1.000						
IGFBP3	rs2854744 (C-202A)	7	intergenic	Asian	C vs A	27.71	3	789/960	0.79(0.52-1.20)	0.262	13.99	0.00	86	0.296	0.298	1.000	0.79(0.50-1.26)	0.328	82	0.57(0.26-1.28)	0.175	73
IL10	rs1800871 (c.-854T>C, -819C>T)	1	5'UTR	All	T vs C	30.14	11	3269/4627	0.93(0.76-1.14)	0.488	37.41	0.00	73	0.640	0.656	0.019	0.90(0.64-1.26)	0.525	75	0.92(0.69-1.23)	0.569	60
IL10	rs1800872 (g.4433A>C, -592C>A)	1	intergenic	All	A vs C	35.53	8	3710/4965	0.90(0.81-1.01)	0.075	15.64	0.03	55	0.174	0.165	0.369	0.86(0.73-1.04)	0.122	52	0.84(0.63-1.11)	0.211	61
IL10	rs1800896 (g.3943A>G, -1082A>G)	1	intergenic	All	G vs A	37.18	10	2861/3817	1.29(1.05-1.59)	0.017	35.95	0.00	75	0.074	0.042	1.000	1.49(0.96-2.31)	0.072	80	1.25(1.00-1.56)	0.048	0
IL13	rs1800925 (-1069C>T, -1112C>T)	5	intergenic	All	T vs C	21.93	3	1788/1920	1.02(0.91-1.13)	0.785	0.91	0.64	0	0.296	0.311	1.000						
IL13	rs20541 (Arg130Gln,+2044G>A)	5	non-synonymous	All	A vs G	21.66	3	1877/2020	1.01(0.90-1.12)	0.921	0.93	0.63	0	1.000	0.999	1.000						
IL17A	rs2275913 (-73G>A, -152G>A)	6	5'UTR	All	A vs G	24.90	3	889/998	1.40(1.00-1.96)	0.051	9.72	0.01	79	1.000	0.932	1.000	1.40(0.90-2.17)	0.140	80	1.76(1.21-2.55)	0.003	18
IL1B	rs1143634 (p.Phe105Phe, Ex5+14C>T)	2	synonymous	All	T vs C	21.37	5	4626/4116	1.05(0.95-1.17)	0.317	5.76	0.22	31	0.462	0.304	0.345	1.14(0.92-1.40)	0.229	41	1.14(0.87-1.48)	0.346	0
IL1B	rs1143627 (g.4970C>T, -31T>C)	2	intergenic	All	C vs T	38.81	9	5735/7138	0.93(0.84-1.03)	0.176	27.03	0.00	70	0.029	0.040	0.104	0.95(0.78-1.14)	0.554	74	0.80(0.68-0.95)	0.010	49
IL1B	rs16944 (C-511T)	2	intergenic	All	T vs C	41.62	11	4866/5910	0.94(0.84-1.06)	0.332	36.62	0.00	73	1.000	0.533	0.215	0.94(0.79-1.12)	0.517	68	0.86(0.70-1.06)	0.155	65
IL4	rs2243250 (-509C>T)	5	intergenic	All	T vs C	42.88	5	3496/4037	1.09(0.92-1.31)	0.320	14.46	0.01	72	0.462	0.815	0.542	1.07(0.68-1.68)	0.784	69	1.12(0.82-1.54)	0.470	71
IL6	rs1800795 (G-174C)	7	intergenic	All	C vs G	42.08	10	4619/5490	1.08(0.94-1.24)	0.287	28.73	0.00	69	0.858	0.567	1.000	1.09(0.84-1.42)	0.517	70	1.14(0.88-1.47)	0.326	47
IL6	rs1800796 (-572C>G, 634C>G)	7	intergenic	All	G vs C	22.43	9	2972/3395	0.96(0.75-1.22)	0.717	54.15	0.00	85	0.754	0.818	0.000	0.99(0.75-1.31)	0.927	84	0.94(0.64-1.39)	0.766	54
IL8	rs4073 (T-251A)	4	intergenic	All	T vs A	49.84	7	4787/5310	1.02(0.90-1.15)	0.764	18.80	0.01	68	1.000	0.783	0.233	1.01(0.83-1.23)	0.894	45	1.10(0.88-1.39)	0.398	71
LIG1	rs156641 (G>A)	19	intron	All	A vs G	31.71	4	1255/2220	1.12(0.98-1.28)	0.092	3.57	0.31	16	0.734	0.139	0.513	1.13(0.95-1.33)	0.163	7	1.45(1.14-1.83)	0.002	0
LIG1	rs20579 (-7C>T)	19	5'UTR	All	A vs G	14.40	5	1818/3139	1.12(0.84-1.51)	0.440	23.17	0.00	83	0.806	0.366	0.017	1.11(0.82-1.50)	0.510	78	1.41(0.66-3.01)	0.380	68
LIG1	rs20581 (p.Asp802Asp, G>A)	19	synonymous	All	A vs G	41.09	4	828/1386	1.02(0.90-1.17)	0.726	0.96	0.81	0	0.734	0.506	1.000	0.91(0.74-1.13)	0.400	0	1.09(0.76-1.57)	0.651	33
LIG1	rs3730931 (IVS9-21A>G)	19	intron	All	G vs A	13.56	4	1399/2364	1.05(0.80-1.38)	0.715	9.55	0.02	69	0.308	0.139	0.174	1.05(0.79-1.40)	0.723	63	0.95(0.55-1.64)	0.859	20
LIG1	rs439132 (A>G)	19	intron	All	G vs A	8.36	3	739/1220	1.21(0.74-1.96)	0.444	6.66	0.04	70	1.000	0.339	0.053	1.21(0.70-2.09)	0.507	72	1.82(0.72-4.55)	0.204	20
LIG1	rs20580 (p.Ala170Ala, c.417C>A)	19	synonymous	All	A vs C	52.69	6	2357/3592	0.92(0.77-1.10)	0.370	24.88	0.00	80	0.133	0.151	0.559	0.95(0.75-1.19)	0.643	58	0.96(0.71-1.30)	0.780	82
LIG4	rs1805388 (p.Thr9Ile,Ex2+54C>T)	13	non-synonymous	All	T vs C	17.05	3	515/1246	1.07(0.77-1.50)	0.676	4.84	1.50	59	1.000	0.785	0.140	1.07(0.76-1.52)	0.688	48	1.41(0.72-2.76)	0.313	7
LTA	rs909253 (A+252G)	6	intron	All	G vs A	35.92	7	941/1683	0.93(0.83-1.05)	0.263	4.55	0.60	0	0.035	0.208	1.000	1.00(0.82-1.23)	0.972	18	0.79(0.61-1.01)	0.057	0
MAPKAPK2	CNV-30450(2 copies/3 copies/4 copies)	1	CNV	Asian	4 copies vs 2 copies	9.76	3	2332/2480	1.60(1.04-2.45)	0.031	10.57	0.01	81	1.000	0.947	0.298	1.28(1.05-1.57)	0.016	59	1.58(1.04-2.40)	0.034	81
MBD4	rs140693 (p.Glu346Lys, G1212A)	3	non-synonymous	Asian	A vs G	35.62	4	2295/2469	0.99(0.86-1.13)	0.881	7.04	0.07	57	1.000	0.655	1.000						
MCP1	rs1024611 (-2518A>G, PvuII)	17	5'UTR	All	G vs A	31.47	3	1985/1981	0.76(0.53-1.09)	0.134	13.04	0.00	85	1.000	0.260	1.000						
MDM2	rs117039649 (G>C, SNP285)	12	intron	All	C vs G	3.20	3	1846/4400	1.10(0.89-1.37)	0.374	0.51	0.77	0	1.000	0.743	1.000	1.09(0.87-1.36)	0.445	0			
MDM2	rs2279744 (SNP309,c.14+309T>G)	12	intergenic	All	G vs T	41.05	19	11076/14434	1.10(1.01-1.19)	0.021	71.76	0.00	75	0.700	0.355	0.242	1.12(1.00-1.27)	0.058	75	1.15(1.04-1.27)	0.005	46
MDM2	rs769412 (p.Glu184Glu,2164A>G,SNP354)	12	synonymous	All	G vs A	27.44	4	1691/1915	1.02(0.91-1.14)	0.785	1.58	0.66	0	0.734	0.836	1.000	0.98(0.84-1.14)	0.768	0	1.11(0.89-1.39)	0.338	0
MGMT	rs12268840 (C>T)	10	intron	Caucasian	T vs C	32.82	3	247/355	0.88(0.64-1.19)	0.396	2.64	0.27	24	1.000	0.996	1.000	0.84(0.60-1.16)	0.282	0	0.87(0.39-1.94)	0.730	35
MGMT	rs12917 (p.Leu115Phe, c.343C>T)	10	non-synonymous	All	T vs C	15.64	7	2552/3836	1.05(0.95-1.17)	0.335	5.87	0.44	0	0.548	0.766	1.000	1.05(0.93-1.18)	0.445	0	1.15(0.85-1.56)	0.377	0
MGMT	rs1803965 (Leu53Leu,171C>T, TaqI)	10	synonymous	All	T vs C	12.27	3	1060/1149	0.96(0.80-1.15)	0.671	0.75	0.69	0	0.296	0.416	1.000	0.98(0.81-1.20)	0.870	0	0.69(0.32-1.45)	0.321	0
MGMT	rs2308321 (p.Ile174Val,Ile143Val,c.520A>G)	10	non-synonymous	All	G vs A	12.32	6	2023/2191	1.05(0.86-1.28)	0.649	7.97	0.16	37	0.452	0.643	1.000	1.06(0.82-1.38)	0.665	48	0.86(0.51-1.43)	0.550	0
MGMT	rs2308327 (p.Lys178Arg, c.533A>G)	10	non-synonymous	Caucasian	G vs A	20.45	5	759/880	0.73(0.50-1.07)	0.109	12.48	0.01	68	0.086	0.079	1.000	0.73(0.47-1.12)	0.149	67	0.92(0.59-1.41)	0.687	0
MIR146A	rs2910164 (C>G)	5	non-coding transcript	All	C vs G	45.26	6	3158/3225	1.16(1.06-1.27)	0.001	6.35	0.27	21	0.260	0.076	0.415	1.20(1.04-1.38)	0.012	24	1.23(1.09-1.39)	0.001	0
MIR196A2	rs11614913 (T>C)	12	non-coding transcript	All	C vs T	48.95	6	2727/2797	1.02(0.88-1.18)	0.839	16.57	0.01	70	0.060	0.093	0.014	1.13(0.91-1.41)	0.260	56	0.94(0.73-1.20)	0.618	71
MLH1	rs1799977 (p.Ile219Val, c.655A>G)	3	non-synonymous	All	G vs A	9.64	5	2147/2355	0.92(0.72-1.18)	0.531	7.91	0.10	49	0.806	0.401	1.000						
MLH1	rs1800734 (-93G>A)	3	5'UTR	All	A vs G	48.86	6	2328/2491	0.98(0.86-1.12)	0.780	11.53	0.04	57	0.707	0.479	0.015	0.80(0.68-0.95)	0.009	24	1.27(0.94-1.70)	0.114	75
MLH3	rs175080 (p.Pro844Leu, C2645T)	14	non-synonymous	All	T vs C	20.13	3	1363/1540	1.03(0.79-1.33)	0.837	5.85	0.05	66	0.296	0.611	0.103						
MMP1	rs1799750 (-1607 1G/2G,insG)	11	intergenic	All	2G vs 1G	54.13	10	4787/4446	1.08(0.96-1.22)	0.197	27.39	0.00	67	0.474	0.852	0.331	1.11(0.99-1.25)	0.087	13	1.10(0.91-1.34)	0.341	70
MMP2	rs243865 (-1306C>T)	16	intergenic	All	T vs C	16.77	3	1751/1729	0.63(0.45-0.89)	0.009	9.86	0.01	80	0.296	0.001	0.458	0.60(0.40-0.90)	0.014	82	0.60(0.36-0.99)	0.047	0
MMP3	rs3025058 (-1612 5A/6A, -1171 5A/6A)	11	intergenic	All	5A vs 6A	44.49	5	3043/2422	1.08(0.93-1.27)	0.322	8.27	0.08	52	0.027	0.100	0.489	1.05(0.92-1.18)	0.489	0	1.09(0.88-1.35)	0.448	31

Gene	Variant	Chr	Category	Ethnicity	Comparison[a]	Frequency(%)[b]	Number evaluated		Lung cancer risk meta-analysis in allelic model								Dominant model			Recessive model		
							Studies	Cases/Controls	OR(95% CI)	*P* value	Q	*P* for Q	I^2(%)	Begg *P*	Modified *P*[c]	Excess *P*[d]	OR(95% CI)	*P* value	I^2(%)	OR(95% CI)	*P* valute	I^2(%)
MMP9	rs3918242 (g.3430C>T,-1562 C>T)	20	intergenic	All	T vs C	13.28	3	1205/1099	0.89(0.69-1.14)	0.357	3.40	0.18	41	1.000	0.987	1.000	0.92(0.74-1.14)	0.438	15	0.53(0.13-2.08)	0.362	48
MPO	rs2333227 (G-463A)	17	intron	All	A vs G	20.93	27	9595/10754	0.92(0.85-1.00)	0.058	56.29	0.00	54	0.269	0.098	0.048	0.92(0.85-1.00)	0.055	38	0.92(0.74-1.15)	0.471	45
MSH3	rs26279 (p.Ala1045Thr,c.3133G>A)	5	non-synonymous	All	G vs A	27.74	4	1649/1849	0.90(0.75-1.08)	0.251	6.83	0.08	56	0.734	0.688	0.447						
MSH6	rs1042821 (p.Gly39Glu,c.116G>A)	2	non-synonymous	All	A vs G	29.44	3	1454/1620	0.95(0.83-1.08)	0.415	2.30	0.32	13	0.296	0.271	1.000						
MTHFR	rs1801131 (p.Ala429Glu, c.1286A>C, A1298C)	1	non-synonymous	All	C vs A	24.91	11	4125/5131	1.07(1.00-1.14)	0.069	7.93	0.64	0	0.876	0.443	1.000	1.03(0.91-1.16)	0.636	38	1.22(0.99-1.51)	0.067	27
MTHFR	rs1801133 (p.Ala222Val, Ex4+79C＞T)	1	non-synonymous	All	T vs C	37.83	16	8692/8412	1.10(0.98-1.24)	0.125	69.01	0.00	78	0.192	0.101	0.069	1.07(0.92-1.26)	0.365	73	1.15(0.96-1.37)	0.124	62
MTR	rs1805087 (Asp919Gly, A2756G)	1	non-synonymous	All	G vs A	13.88	4	2488/3335	1.06(0.93-1.22)	0.387	4.61	0.20	35	1.000	0.920	0.261	1.07(0.88-1.29)	0.507	55	1.14(0.79-1.63)	0.490	0
MTRR	rs1801394 (Ile49Met, A66G)	5	non-synonymous	All	G vs A	43.28	3	1668/2291	1.13(1.03-1.24)	0.011	1.29	0.53	0	1.000	0.916	1.000	1.19(1.00-1.42)	0.054	23	1.15(0.98-1.34)	0.086	0
MUTYH	rs3219489 (p.Gln324His, c.972G>C)	1	non-synonymous	All	C vs G	31.13	4	1250/1137	1.10(0.86-1.39)	0.455	8.17	0.04	63	1.000	0.778	0.321	1.05(0.82-1.34)	0.690	41	1.25(0.76-2.06)	0.372	54
MYCL	L/S(EcoRI)	1	the restriction site absence	All	S vs L	49.65	9	636/1004	1.05(0.91-1.22)	0.530	3.99	0.86	0	1.000	0.792	1.000	1.10(0.86-1.40)	0.443	0	1.03(0.81-1.31)	0.813	0
NAT2	Rapid/slow(Acetylation Phenotype)	8	phenotype	All	Slow vs rapid	48.33	17	3747/4852	1.06(0.90-1.25)	0.502	38.19	0.00	58	0.064	0.622	0.000						
NAT2	rs1799929 (p.Leu161Leu, 481C>T)	8	synonymous	All	T vs C	36.88	3	576/686	1.11(0.87-1.41)	0.407	4.14	0.13	52	1.000	0.717	0.257	1.36(0.76-2.44)	0.308	80	1.06(0.72-1.57)	0.754	24
NAT2	rs1799930 (p.Arg197Gln, 590G>A,NAT2*6B)	8	non-synonymous	All	A vs G	32.47	4	712/893	1.01(0.81-1.25)	0.964	5.94	0.12	50	0.734	0.903	0.107	0.97(0.74-1.28)	0.831	65	1.20(0.82-1.76)	0.361	21
NBN	rs1805794 (p.Gln185Glu, Ex5-32C>G, 605C>G)	8	non-synonymous	All	G vs C	49.91	8	3578/4688	0.95(0.80-1.13)	0.554	41.58	0.00	83	0.174	0.292	0.001	0.93(0.66-1.30)	0.662	82	0.90(0.72-1.12)	0.334	58
NFKB1	rs28362491 (-94ins/delATTG)	4	intergenic	All	Ins vs del	45.72	3	935/935	1.02(0.72-1.43)	0.919	11.51	0.00	83	0.296	0.355	0.003	1.04(0.59-1.83)	0.892	82	1.01(0.69-1.49)	0.961	64
NOD2	rs2066847 (3020insC, WT/insC, L1007fs)	16	frameshift	All	Carriers vs non-carriers	0.50	3	807/4078	1.42(1.07-1.90)	0.017	1.04	0.59	0	1.000	0.988	0.567	1.42(1.07-1.90)	0.017	0			
NQO1	rs1800566 (p.Pro187Ser,c.648C>T, 609C>T,*2)	16	non-synonymous	All	T vs C	29.63	23	5088/6381	1.02(0.92-1.14)	0.670	54.88	0.00	60	0.398	0.598	0.031	1.03(0.92-1.16)	0.586	56	1.06(0.86-1.31)	0.586	45
OGG1	rs1052133 (p.Ser326Cys,c.977C>G,8055C>G)	3	non-synonymous	All	G vs C	38.90	33	11944/14309	1.04(0.97-1.11)	0.243	76.29	0.00	58	0.914	0.970	0.000	1.06(0.96-1.17)	0.289	54	1.11(1.00-1.24)	0.052	41
PADPRP	A/B (a 193-bp deletion)	13	STR	All	Del vs non-del	34.59	3	288/292	1.13(0.70-1.82)	0.617	6.42	0.04	69	0.296	0.331	0.168	1.24(0.71-2.18)	0.451	59	1.09(0.51-2.31)	0.829	46
PARP1	rs1136410(p.Val762Ala,Ex17+8T＞C)	1	non-synonymous	All	C vs T	41.02	6	3192/2874	1.01(0.89-1.15)	0.842	12.64	0.03	60	1.000	0.313	0.170	1.05(0.88-1.27)	0.572	36	1.11(0.88-1.40)	0.372	43
PCNA	rs25406(2232C>T)	20	intron	All	T vs C	38.63	4	970/1302	0.91(0.77-1.06)	0.231	4.99	0.17	40	0.734	0.345	1.000	0.91(0.76-1.10)	0.322	0	0.91(0.52-1.60)	0.741	77
PDCD5	rs1862214 (G>A)	19	intergenic	All	G vs C	32.06	3	737/683	1.32(1.12-1.56)	0.001	1.86	0.40	0	0.296	0.156	1.000	1.42(1.13-1.77)	0.002	0	1.54(0.83-2.87)	0.173	58
POLD1	rs1726801 (p.Arg119His,c.356G>A)	19	non-synonymous	Asian	A vs G	19.30	3	1263/1420	1.11(0.96-1.28)	0.168	1.61	0.45	0	0.296	0.073	1.000						
POLI	rs3730668 (-78G>T)	18	intergenic	All	T vs G	58.99	3	672/823	0.94(0.80-1.09)	0.397	0.63	0.73	0	1.000	0.688	1.000						
POLI	rs8305 (Thr706Ala, 2180A>G)	18	non-synonymous	All	G vs A	25.24	4	1655/2593	1.09(0.98-1.20)	0.111	2.17	0.54	0	0.734	0.250	0.379						
POLL	rs3730477 (p.Arg438Trp, 1683C>T)	10	non-synonymous	All	T vs C	3.66	3	1356/1542	1.29(0.69-2.40)	0.425	4.07	0.13	51	1.000	0.503	0.347						
PON1	rs662 (p.Gln192Arg,c.575A>G)	7	non-synonymous	All	A vs G	46.70	3	995/834	0.77(0.67-0.88)	2.02×10^{-4}	0.71	0.70	0	1.000	0.704	1.000	0.78(0.63-0.96)	0.018	0	0.61(0.46-0.83)	0.001	30
PPARG	rs1801282 (p.Pro12Ala,c.36C>G)	3	non-synonymous	All	G vs C	15.24	4	2476/3377	1.00(0.88-1.12)	0.956	0.82	0.84	0	0.734	0.887	1.000	0.93(0.93-1.18)	0.542	0	1.17(0.80-1.73)	0.415	0
PPP1R13L	rs6966 (T>A)	19	3'UTR	All	T vs A	46.66	5	1120/1361	1.09(0.92-1.29)	0.302	6.96	0.14	43	0.806	0.755	0.349	1.16(0.95-1.43)	0.145	0	1.11(0.82-1.51)	0.502	54
PPP1R13L	rs1970764 (IVS1-4364A>G)	19	intron	All	G vs A	36.71	3	678/726	1.12(0.90-1.39)	0.313	3.24	0.20	38	1.000	0.918	1.000	1.12(0.89-1.41)	0.349	0	1.07(0.54-2.10)	0.851	69
PROM1	rs2240688 (A>C)	4	3'UTR	Asian	C vs A	27.37	3	2332/2457	0.83(0.76-0.91)	6.92×10^{-5}	0.02	0.99	0	0.296	0.379	0.281	0.80(0.72-0.90)	1.65×10^{-4}	0	0.78(0.63-0.97)	0.026	0
PTGS2	rs20417 (-765G>C)	1	intergenic	All	C vs G	16.15	4	2355/2731	0.96(0.86-1.08)	0.505	1.84	0.61	0	1.000	0.299	1.000	0.80(0.57-1.13)	0.200	42			
PTGS2	rs689466 (g.38139393T>C,-1195A>G)	1	intergenic	All	G vs A	38.07	4	1676/2180	0.82(0.65-1.02)	0.070	13.44	0.00	78	0.734	0.218	0.626	0.78(0.62-0.97)	0.026	56	0.87(0.62-1.20)	0.391	66
PTGS2	rs5275 (c.*427T>C, 8473T>C)	1	3'UTR	All	C vs T	29.41	11	6813/7988	0.95(0.83-1.09)	0.493	58.16	0.00	83	0.755	0.955	0.038	0.93(0.79-1.10)	0.389	74	0.90(0.62-1.30)	0.578	79
RAD23B	rs1805329 (p.Ala249Val,Ex7+65C＞T)	9	non-synonymous	All	T vs C	23.29	4	653/878	1.10(0.80-1.51)	0.572	9.39	0.03	68	0.089	0.059	0.231	1.10(0.67-1.81)	0.703	75	1.04(0.52-2.06)	0.922	49
REV1	rs3087386 (p.Phe257Ser,892T>C)	2	non-synonymous	All	C vs T	40.81	3	1437/1818	1.03(0.92-1.14)	0.658	0.51	0.78	0	1.000	0.366	1.000				0.98(0.74-1.31)	0.909	52
REV3L	rs462779 (p.Thr1146Ile,4259C>T)	6	non-synonymous	Asian	T vs C	39.36	4	1937/2335	1.11(1.02-1.22)	0.021	0.54	0.91	0	0.734	0.921	1.000						
REV3L	rs465646 (460T>C)	6	3'UTR	All	C vs T	18.18	4	1438/1683	0.88(0.73-1.07)	0.197	5.42	0.14	45	0.308	0.058	0.429	0.78(0.67-0.92)	0.003	0	0.93(0.62-1.41)	0.740	0
SFTPB	wild type/variation (deletions or insertions)	2	intron	All	Variation vs wild	5.83	3	157/240	1.92(1.11-3.33)	0.020	0.08	0.96	0	0.296	0.495	1.000	2.09(1.16-3.76)	0.014	0	0.32(0.15-0.68)	0.003	0
SOD2	rs4880 (rs1799725, Ala16Val)	6	non-synonymous	All	T vs C	51.48	9	3738/4467	1.20(1.06-1.36)	0.005	20.34	0.01	61	0.348	0.723	0.733	1.33(1.08-1.64)	0.007	52	1.17(0.98-1.38)	0.082	55
SULT1A1	rs9282861(p.Arg213His,c.638G>A,*1/*2)	16	non-synonymous	All	A vs G	21.68	6	1965/2286	1.20(0.97-1.48)	0.094	18.08	0.00	72	1.000	0.779	1.000	1.24(0.94-1.63)	0.127	74	1.14(0.87-1.50)	0.343	0
TDG	rs4135113(p.Gly199Ser,c.595G>A)	12	non-synonymous	All	A vs G	6.13	3	289/318	0.85(0.51-1.42)	0.531	1.45	0.49	0	1.000	0.497	1.000						
TERT	rs2736098(p.Ala305Ala,c.915G>A)	5	synonymous	All	A vs G	33.01	7	4660/4825	1.20(1.08-1.33)	0.001	17.98	0.01	67	0.548	0.734	0.706	1.23(1.13-1.35)	4.84×10^{-6}	0	1.65(1.43-1.89)	1.87×10^{-12}	0
TGFB1	rs1800469 (C-509T)	19	intergenic	Asian	T vs C	49.31	4	1091/1238	0.95(0.85-1.07)	0.428	3.10	0.38	3	0.089	0.100	1.000	0.96(0.72-1.28)	0.777	56	0.96(0.79-1.17)	0.691	0
TGFB1	rs1800470 (p.Leu10Pro,c.29C>T)	19	non-synonymous	All	C vs T	44.49	7	1398/1553	1.16(0.97-1.39)	0.104	15.61	0.02	62	0.764	0.718	1.000	1.33(0.97-1.82)	0.079	69	1.10(0.92-1.32)	0.285	0
TNF	rs1799724 (-857C>T, -1036C>T)	6	intergenic	All	C vs T	28.19	3	2080/2164	0.98(0.86-1.11)	0.727	1.80	0.41	0	1.000	0.797	1.000						
TNF	rs1800629 (G-308A, *1/*2)	6	intergenic	All	A vs G	15.20	15	3817/4813	1.09(0.85-1.41)	0.488	84.83	0.00	84	0.843	0.839	0.065	1.11(0.79-1.57)	0.538	83	1.28(0.73-2.24)	0.395	41
TNF	rs361525 (A-417G, -238G>A)	6	intergenic	All	A vs G	7.32	6	1047/1099	1.07(0.55-2.08)	0.847	33.99	0.00	85	0.133	0.356	0.001	0.99(0.52-1.88)	0.967	82			
TNFRSF10A	rs2230229 (p.Arg441Lys, c.1322G>A)	8	non-synonymous	All	G vs A	14.57	5	1479/1932	1.06(0.80-1.39)	0.702	11.74	0.02	66	0.806	0.885	1.000	1.10(0.83-1.47)	0.499	61	0.89(0.56-1.41)	0.616	1
TP53	rs1042522 (p.Pro72Arg, c.215C>G)	17	non-synonymous	All	C vs G	33.34	59	23673/25061	1.04(0.98-1.12)	0.221	287.17	0.00	80	0.013	0.011	0.000	1.06(0.98-1.15)	0.176	72	1.08(0.96-1.22)	0.216	73
TP53	rs12951053 (IVS7+92T>G)	17	intron	All	G vs T	9.93	3	475/569	1.57(1.11-2.23)	0.011	3.19	0.20	37	0.296	0.169	0.579	1.80(1.01-3.19)	0.045	67	1.82(0.76-4.32)	0.178	0
TP53	rs1625895 (IVS6+62G>A, A2>A1)	17	intron	All	A vs G	14.33	8	2666/2847	1.10(0.90-1.34)	0.351	17.49	0.01	60	0.386	0.160	0.117	1.09(0.84-1.41)	0.534	68	0.95(0.53-1.70)	0.860	44
TP53	rs1642785 (IVS2+38G>C, 1827G>C)	17	5'UTR	All	C vs G	36.23	5	534/621	1.05(0.88-1.25)	0.614	3.00	0.56	0	0.086	0.011	1.000	1.13(0.88-1.45)	0.322	0	0.94(0.66-1.33)	0.711	0
TP53	rs17878362(IVS3 16bp Del/Ins,a 16-bp duplication)	17	intergenic	Caucasian	Ins vs del	12.97	4	4196/4327	1.13(0.96-1.34)	0.152	8.22	0.04	64	0.734	0.904	1.000	1.14(0.93-1.38)	0.210	67	1.25(0.75-2.08)	0.395	55
TP53BP1	rs560191(p.Asp353Glu, c.1059C>G）	15	non-synonymous	All	G vs C	46.36	3	1393/1373	0.94(0.69-1.30)	0.723	15.68	0.00	87	1.000	0.814	0.219	0.97(0.67-1.40)	0.853	79	0.88(0.51-1.53)	0.655	85
TP63	rs10937405(C>T)	3	intron	All	T vs C	42.62	4	4927/8794	0.87(0.81-0.94)	2.20×10^{-4}	4.56	0.21	34	0.308	0.108	0.636	0.85(0.76-0.94)	0.002	34	0.82(0.75-0.91)	6.83×10^{-5}	0
TP73	G4C14/A4T14	1	Combined variant	All	AT vs GC(mutation vs wild)	22.98	7	3054/3311	1.04(0.88-1.24)	0.647	23.42	0.00	74	0.024	0.053	0.000	1.10(0.93-1.30)	0.289	59	1.00(0.67-1.49)	0.980	68
TYMS	28-bp tandem repeat	18	STR	All	2R vs 3R(2R vs Non-2R)	30.41	4	2052/2861	0.99(0.90-1.08)	0.742	1.79	0.62	0	1.000	0.446	1.000	0.97(0.85-1.09)	0.582	0	1.02(0.85-1.22)	0.857	0
UGT1A6	rs1105879 (p.Arg184Ser,c.552A>C)	2	non-synonymous	All	G vs T	20.40	3	253/250	3.03(0.79-11.62)	0.106	36.84	0.00	95	0.296	0.248	0.151	6.14(0.70-54.11)	0.102	96	2.09(0.56-7.79)	0.272	52
UGT1A6	rs6759892 (p.Ser7Ala, c.19T>G)	2	non-synonymous	All	G vs T	25.10	3	266/261	2.27(1.14-4.53)	0.020	12.15	0.00	84	1.000	0.437	0.338	2.96(1.12-7.83)	0.029	86	3.17(0.69-14.68)	0.139	41
VEGFA	rs2010963 (634G>C)	6	5'UTR	All	C vs G	37.63	5	2989/2522	1.12(0.99-1.27)	0.083	7.23	0.12	45	0.806	0.667	1.000	1.21(0.98-1.49)	0.071	50	1.12(0.88-1.42)	0.377	46
VEGFA	rs3025039 (C936T)	6	3'UTR	All	T vs C	14.90	6	2887/2506	1.01(0.87-1.17)	0.917	7.10	0.21	30	1.000	0.777	1.000	1.02(0.90-1.15)	0.790	0	1.09(0.70-1.69)	0.702	22
VEGFA	rs699947 (C-2578A)	6	intergenic	All	A vs C	26.74	4	879/804	0.97(0.81-1.15)	0.712	3.60	0.31	17	0.308	0.413	1.000	0.89(0.70-1.13)	0.335	26	1.54(0.69-3.45)	0.297	73
VEGFA	rs833061 (-460T>C)	6	intergenic	All	C vs T	40.74	5	3011/2532	0.93(0.82-1.05)	0.245	7.02	0.14	43	0.221	0.222	0.482	0.94(0.78-1.14)	0.527	50	0.87(0.71-1.05)	0.147	15
vWF	rs9295740 (G>A)	6	intergenic	All	A vs G	19.86	3	2221/2334	1.02(0.92-1.13)	0.685	0.59	0.74	0	0.296	0.144	1.000	1.03(0.91-1.16)	0.670	0	1.02(0.76-1.38)	0.896	0
WWOX	CNV-67048(2copies/1copy/0copy)	16	CNV	Asian	0 copy vs 2 copies	2.86	4	2942/3074	2.06(1.58-2.70)	1.20×10^{-7}	0.54	0.91	0	1.000	0.648	1.000	1.38(1.24-1.54)	1.00×10^{-8}	0	1.92(1.47-2.50)	1.59×10^{-6}	0
XPA	rs1800975 (c.-4A>G, 23G>A)	9	5'UTR	All	A vs G	36.74	12	4221/5240	1.09(0.99-1.19)	0.082	22.18	0.02	50	0.411	0.292	0.125	1.04(0.93-1.17)	0.467	37	1.22(1.05-1.42)	0.011	33
XPC	PAT-/+ (24660_24664delGTAAC inspoly(AT))	3	STR	All	Ins vs non-ins	39.34	4	1417/1449	1.06(0.90-1.25)	0.475	6.34	0.10	53	1.000	0.677	0.252	1.02(0.82-1.26)	0.877	43	1.22(0.95-1.57)	0.113	31
XPC	rs2228000 (p.Ala499Val, Ex9-377C>T)	3	non-synonymous	All	T vs C	28.61	6	2981/3537	1.05(0.97-1.14)	0.205	5.17	0.40	3	1.000	0.890	0.347	1.07(0.94-1.21)	0.302	29	1.09(0.91-1.31)	0.342	0
XPC	rs2228001 (p.Gln939Lys, Ex16+211A＞C)	3	non-synonymous	All	C vs A	37.46	16	5920/7337	1.04(0.98-1.10)	0.232	19.01	0.21	21	0.137	0.136	0.538	1.04(0.93-1.16)	0.471	37	1.12(0.99-1.27)	0.074	10
XRCC1	rs1001581 (c.145-216G>A)	19	intron	All	T vs C	34.52	5	851/1166	1.17(1.00-1.37)	0.044	5.59	0.23	28	0.221	0.132	1.000	1.25(0.93-1.69)	0.139	54	1.20(0.91-1.59)	0.200	0
XRCC1	rs1799782 (p.Arg194Trp,c.580C>T,Ex6-22C＞T)	19	non-synonymous	All	T vs C	18.19	30	11096/13772	0.90(0.82-0.98)	0.022	76.74	0.00	62	0.372	0.536	0.121	0.85(0.76-0.96)	0.010	62	1.06(0.87-1.31)	0.563	35
XRCC1	rs2030404 (A>G)	19	3'UTR	All	G vs A	35.55	3	514/751	0.98(0.83-1.17)	0.836	2.05	0.36	2	1.000	0.567	1.000						
XRCC1	rs2256507 (G>A)	19	intron	All	A vs G	25.61	3	565/779	1.13(0.95-1.35)	0.162	1.79	0.41	0	0.296	0.065	1.000	1.12(0.90-1.40)	0.308	0	1.41(0.87-2.28)	0.167	15
XRCC1	rs25486 (A>G)	19	intron	All	G vs A	40.44	3	507/795	0.99(0.74-1.34)	0.955	5.17	0.08	61	1.000	0.885	1.000	1.10(0.85-1.42)	0.482	0	0.97(0.50-1.89)	0.923	69
XRCC1	rs25487 (p.Gln399Arg,c.1196A>G)	19	non-synonymous	All	A vs G	29.70	51	18308/22033	1.03(0.98-1.08)	0.272	109.98	0.00	55	0.465	0.706	0.001	1.01(0.95-1.08)	0.685	45	1.16(1.03-1.30)	0.018	54
XRCC1	rs25489 (p.Arg280His,c.839G>A,Ex9+16G>A)	19	non-synonymous	All	A vs G	7.65	18	7822/8474	0.96(0.81-1.14)	0.650	60.06	0.00	72	0.112	0.592	0.002	0.95(0.76-1.19)	0.640	77	1.09(0.69-1.72)	0.703	0
XRCC1	rs2682562 (A>G)	19	3'UTR	All	G vs A	14.05	3	513/751	1.00(0.79-1.25)	0.974	0.70	0.71	0	1.000	0.989	1.000						
XRCC1	rs2682585 (p.His6Arg,c.17A>G)	19	non-synonymous	All	A vs G	15.75	3	514/749	1.09(0.88-1.35)	0.416	1.91	0.39	0	1.000	0.971	1.000						
XRCC1	rs2854510 (A>G)	19	intron	All	G vs A	17.58	3	513/751	0.97(0.79-1.20)	0.777	0.48	0.79	0	0.296	0.259	1.000						
XRCC1	rs3213255 (A>G)	19	intron	All	G vs A	38.15	3	1089/1506	1.21(1.08-1.35)	0.001	1.57	0.46	0	0.296	0.118	1.000	1.18(1.00-1.39)	0.052	0	1.30(0.89-1.90)	0.173	50

Gene	Variant	Chr	Category	Ethnicity	Comparison[a]	Frequency(%)[b]	Number evaluated		Lung cancer risk meta-analysis in allelic model								Dominant model			Recessive model		
							Studies	Cases/Controls	OR(95% CI)	*P* value	Q	*P* for Q	I^2(%)	Begg *P*	Modified *P*[c]	Excess *P*[d]	OR(95% CI)	*P* value	I^2(%)	OR(95% CI)	*P* valute	I^2(%)
XRCC1	rs3213403 (A>G)	19	3'UTR	All	G vs A	9.52	3	511/751	0.98(0.72-1.33)	0.880	2.19	0.34	9	1.000	0.706	1.000						
XRCC1	rs3547 (p.Gln632Gln,c.1896G>A)	19	synonymous	All	A vs G	23.31	5	881/1201	1.00(0.86-1.17)	0.991	1.17	0.88	0	0.806	0.559	1.000	1.03(0.83-1.27)	0.800	0	1.10(0.71-1.70)	0.671	0
XRCC1	rs3213245 (-77T>C)	19	5'UTR	All	C vs T	11.03	5	2795/2865	1.29(1.04-1.59)	0.020	12.53	0.01	68	0.806	0.539	0.661	1.31(1.04-1.65)	0.021	65	1.58(1.05-2.38)	0.030	0
XRCC2	rs3218536 (p.Arg188His,c.563G>A)	7	non-synonymous	Caucasian	A vs G	8.08	3	645/1373	1.23(0.54-2.77)	0.625	16.79	0.00	88	1.000	0.049	0.317	1.26(0.51-3.11)	0.618	89	1.78(0.58-5.46)	0.315	0
XRCC3	rs1799794 (4541A>G)	14	5'UTR	All	G vs A	41.09	4	1389/1941	0.91(0.76-1.08)	0.266	7.01	0.07	57	1.000	0.614	0.438	0.92(0.72-1.17)	0.495	58	0.82(0.67-0.99)	0.038	0
XRCC3	rs1799796(17893A>G,IVS5-14)	14	intron	All	G vs A	31.99	5	1478/3040	1.02(0.92-1.12)	0.756	3.45	0.49	0	0.806	0.675	1.000	1.00(0.87-1.16)	0.956	16	1.06(0.85-1.32)	0.604	0
XRCC3	rs861539(p.Thr241Met,c.722C>T)	14	non-synonymous	All	T vs C	20.93	18	6407/8356	1.04(0.95-1.13)	0.414	27.73	0.05	39	0.705	0.314	0.142	1.03(0.92-1.16)	0.600	42	1.06(0.90-1.24)	0.489	0

Chr=chromosome; OR=odds ratio, 95%CI = 95% confidence interval; ins=insertion; del=deletion; STR=short tandem repeat ; VNTR=variable number of tandem repeats; UTR=untranslated region; CNV=copy number variation.

[a] Allelic contrast or phenotype trait for common variants; genetic comparison for rare variants or variants only with genotype group data

[b] Frequency of minor allele or effect genotype(s) in controls in main meta-analyses

[c] *P* value for potential small study effect based on the modified Egger's test

[d] *P* value for possible existing an excess of significant findings (the number of observed significant studies more than the number of expected significant studies) based on the excess significance test

.

Supplementary Table S2. Credibility assessment for variants with significant associations with lung cancer risk in meta-analyses for all available data (Continued on next page)

Genes	Variants	Alleles[a]	Ethinicity	Number evaluated		Lung-cancer risk meta-analysis					Venice criteria grades[d]	Credibility of evidence[e]	Amount of evidence[f]		Replication[g]	Protection from bias[h]			Sensitivity analysis[i]: Initial study influence		Sensitivity analysis[i]: Deviation from HWE	Low OR[j]	Modified P[k]	Excess P[l]	Begg P[m]
				Studies	Cases/Controls	Genetic models	OR(95%CI)[b]	P value	I^2 (%)	P_Q[c]			Numbers	Grade		Grade	Reasons for bias	Reason for bias exemption	OR(95%CI)	P value					
**Associations identified by analysis of all available data in main meta-analysis**																									
AGER	rs1800624	A/T	Asian	3	1656/1693	Allelic	1.18(1.04-1.33)	0.010	16	0.305	AAC	Weak	2292	A	A	C	First study		1.20(0.90-1.60)	0.215	None	No	0.480	1.000	1.000
APEX1	rs1760944	A/C	All	8	3588/3783	Allelic	1.16(1.08-1.25)	2.85×10^{-5}	9	0.360	AAA	Strong	7338	A	A	A			1.17(1.08-1.26)	0.000	None	No	0.119	0.287	0.386
ATM	rs189037	A/G	Asian	5	3036/3415	Allelic	1.09(1.00-1.18)	0.050	29	0.227	ABC	Moderate	5653	A	B	C	Low OR	Highly consistent OR	1.12(1.02-1.22)	0.016	None	Yes	0.195	1.000	0.806
AXIN2	rs2240308	T/C	All	3	758/742	Allelic	0.73(0.63-0.85)	6.39×10^{-5}	0	0.398	AAA	Strong	1015	A	A	A			0.76(0.64-0.90)	0.001	None	No	0.120	0.251	1.000
BCL2	rs2279115	A/C	All	5	1847/2367	Allelic	0.65(0.46-0.91)	0.011	91	0.000	ACC	Weak	3448	A	C	C	Small study		0.58(0.51-0.66)	0.000	None	No	0.035	0.481	0.624
CD3EAP	rs967591	A/G	All	3	676/726	Allelic	1.23(1.01-1.49)	0.036	22	0.278	BAA	Moderate	956	B	A	A			1.33(1.10-1.61)	0.003	None	No	0.988	1.000	1.000
CHRNA3	rs578776	T/C	All	3	1245/2009	Allelic	0.87(0.77-0.98)	0.018	0	0.908	AAC	Weak	2089	A	A	C	First study		0.86(0.70-1.05)	0.139	None	No	0.969	0.576	1.000
CHRNA3	rs938682	C/T	All	3	1240/1986	Allelic	0.86(0.76-0.96)	0.009	0	0.582	AAC	Weak	1836	A	A	C	First study,small study		0.91(0.77-1.09)	0.307	None	No	0.054	1.000	0.296
CHRNA3	rs12914385	T/C	All	4	5356/2873	Allelic	1.20(1.01-1.44)	0.044	76	0.007	ACA	Weak	6567	A	C	A			1.32(1.19-1.46)	0.000	None	No	0.353	1.000	0.734
CHRNA3	rs6495309	T/C	All	4	3381/4244	Allelic	0.83(0.77-0.89)	6.55×10^{-8}	0	0.427	AAA	Strong	5804	A	A	A			0.83(0.76-0.91)	0.000	None	No	0.727	1.000	1.000
CHRNA5	rs16969968	A/G	All	11	6222/62452	Allelic	1.23(1.06-1.43)	0.007	80	0.000	ACC	Weak	43607	A	C	C	Excess of significant studies		1.20(1.02-1.42)	0.032	NO	No	0.131	0.071	0.119
CLPTM1L	rs402710	T/C	All	13	7214/8051	Allelic	0.89(0.83-0.95)	2.63×10^{-4}	38	0.078	ABC	Weak	9708	A	B	C	Low OR		0.88(0.82-0.95)	0.001	NO	Yes	0.437	0.335	0.669
CXCR2	rs1126579	T/C	All	3	942/964	Allelic	0.84(0.74-0.96)	0.009	0	0.967	AAA	Strong	2037	A	A	A			0.84(0.73-0.96)	0.012	None	No	0.496	1.000	1.000
CYP1A1	rs4646903	C/T	All	56	9789/12307	Allelic	1.16(1.07-1.25)	1.59×10^{-4}	55	0.000	ACC	Weak	9955	A	C	C	Excess of significant studies		1.16(1.07-1.25)	0.000	NO	No	0.256	0.014	0.772
CYP1A1	rs1048943	G/A	All	54	9869/12114	Allelic	1.23(1.11-1.36)	7.64×10^{-5}	67	0.000	ACC	Weak	8169	A	C	C	Small study,excess of significant studies		1.22(1.10-1.35)	0.000	NO	No	0.081	0.007	0.649
CYP1B1	rs1056836	G/C	All	12	3033/3866	Allelic	1.13(1.05-1.22)	0.002	0	0.551	AAC	Weak	5372	A	A	C	Low OR,publication bias		1.13(1.04-1.22)	0.003	None	Yes	0.228	0.387	0.064
CYP2A6	rs5031016	C/T	All	3	1527/1138	Allelic	0.57(0.33-1.00)	0.048	73	0.025	BCC	Weak	465	B	C	C	First study		0.46(0.17-1.24)	0.125	NO	No	0.587	1.000	0.296
CYP2A6	rs1801272	A/T	Caucasian	3	2411/2644	carriers vs non-carriers	0.66(0.52-0.84)	0.001	0	0.674	BAB	Moderate	307	B	A	B	Missing information		0.65(0.51-0.83)	0.001	NA	No	0.929	1.000	1.000
CYP2E1	rs6413432	A/T(C/D)	All	14	2944/3347	Allelic	0.78(0.71-0.85)	6.76×10^{-8}	0	0.821	AAA	Strong	2561	A	A	A			0.78(0.71-0.86)	0.000	NO	No	0.999	0.541	0.827
CYP2E1	rs2031920	T/C(c2/c1)	All	23	4983/6628	Allelic	0.86(0.76-0.97)	0.018	50	0.003	ACA	Weak	4010	A	C	A			0.86(0.76-0.97)	0.015	NO	No	0.250	0.309	0.509
ELANE	rs351107	G/T	Caucasian	3	745/762	Allelic	0.55(0.34-0.87)	0.011	29	0.246	BBC	Weak	126	B	B	C	First study		0.57(0.24-1.38)	0.213	None	No	0.794	0.565	1.000
ELANE	rs7254054	A/G	Caucasian	3	754/750	Allelic	0.77(0.61-0.97)	0.030	46	0.155	BBC	Weak	735	B	B	C	First study,small study		0.84(0.53-1.32)	0.444	NA	No	0.022	0.567	0.296
ERCC1	rs11615	C/T	All	12	5731/7058	Allelic	0.90(0.83-0.99)	0.023	52	0.018	ACC	Weak	13244	A	C	C	Low OR,small study,publication bias	Highly consistent OR	0.90(0.82-0.99)	0.026	NO	Yes	0.072	0.434	0.086
ERCC2	rs238406	A/C	All	6	1754/2688	Allelic	1.12(1.02-1.23)	0.013	0	0.558	AAC	Weak	3611	A	A	C	Low OR		1.10(1.01-1.21)	0.037	NO	Yes	0.332	0.599	0.260
ERCC2	rs13181	C/A	All	40	13111/16749	Allelic	1.12(1.05-1.19)	4.18×10^{-4}	49	0.000	ABC	Weak	14855	A	B	C	Low OR,excess of significant studies		1.12(1.05-1.19)	0.001	NO	Yes	0.272	0.048	0.753
ERCC5	rs1047768	T/C	All	4	1449/2248	Allelic	0.86(0.74-1.00)	0.049	48	0.123	ABC	Weak	3204	A	B	C	First study,HWE		0.86(0.72-1.04)	0.114	Yes	No	0.414	1.000	0.734
ERCC6	rs3793784	G/C	All	3	1643/1689	Allelic	0.75(0.60-0.92)	0.007	68	0.044	ACA	Weak	1834	A	C	A			0.72(0.55-0.95)	0.022	None	No	0.550	0.167	1.000
FGFR4	rs351855	A/G	All	4	1083/1275	Allelic	0.82(0.69-0.98)	0.025	33	0.214	ABC	Weak	1892	A	B	C	First study,small study,publication bias		0.90(0.74-1.09)	0.266	NO	No	0.015	1.000	0.089
GSTM1	Present/Null	NA	All	131	32985/37533	null vs present	1.18(1.12-1.24)	2.54×10^{-11}	52	0.000	ACC	Weak	35699	A	C	C	Small study,excess of significant studies		1.18(1.12-1.24)	0.000	None	No	0.037	0.011	0.105
GSTP1	rs1695	G/A	All	46	12521/14411	Allelic	1.08(1.02-1.15)	0.011	55	0.000	ACC	Weak	16519	A	C	C	Low OR,small study,excess of significant studies,publication bias		1.07(1.01-1.14)	0.024	NO	Yes	0.009	0.008	0.075
GSTT1	Present/Null	NA	All	76	22796/25134	null va present	1.10(1.02-1.19)	0.015	58	0.000	ACC	Weak	13531	A	C	C	Low OR,excess of significant studies		1.10(1.02-1.18)	0.018	None	Yes	0.345	0.000	0.346
HIF1A	rs11549467	A/G	All	3	509/566	Allelic	2.27(1.74-2.96)	1.62×10^{-9}	0	0.481	BAA	Moderate	297	B	A	A			2.31(1.77-3.02)	0.000	None	No	0.261	0.610	0.296
HRAS1	common/rare (HRAS1 VNTR)	NA	Caucasian	4	746/1174	rare vs common	2.55(1.01-6.45)	0.048	69	0.023	BCC	Weak	305	B	C	C	First study		2.02(0.66-6.21)	0.221	None	No	0.561	0.346	0.734
HYKK	rs931794	G/A	All	5	2435/3180	Allelic	1.23(1.14-1.34)	1.85×10^{-7}	0	0.864	AAA	Strong	3927	A	A	A			1.23(1.11-1.36)	0.000	None	No	0.967	0.411	1.000
IL10	rs1800896	G/A	All	10	2861/3817	Allelic	1.29(1.05-1.59)	0.017	75	0.000	ACC	Weak	5175	A	C	C	First study,small study,publication bias		1.13(0.97-1.30)	0.108	NO	No	0.042	1.000	0.074
MAPKAPK2	CNV-30450	NA	Asian	3	2332/2480	4 copies vs 2 copies	1.60(1.04-2.45)	0.031	81	0.005	BCB	Weak	586	B	C	B	Missing information		NA		None	No	0.947	0.298	1.000
MDM2	rs2279744	G/T	All	19	11076/14434	Allelic	1.10(1.01-1.19)	0.021	75	0.000	ACC	Weak	21593	A	C	C	Low OR		1.10(1.01-1.19)	0.031	None	Yes	0.355	0.242	0.700
MIR146A	rs2910164	C/G	All	6	3158/3225	Allelic	1.16(1.06-1.27)	0.001	21	0.274	AAC	Weak	5983	A	A	C	Small study		1.20(1.10-1.31)	0.000	None	No	0.076	0.415	0.260
MMP2	rs243865	T/C	All	3	1751/1729	Allelic	0.63(0.45-0.89)	0.009	80	0.007	BCC	Weak	954	B	C	C	First study,small study		0.74(0.37-1.48)	0.395	None	No	0.001	0.458	0.296
MTRR	rs1801394	G/A	All	3	1668/2291	Allelic	1.13(1.03-1.24)	0.011	0	0.525	AAC	Weak	3616	A	A	C	HWE,low OR		1.12(1.02-1.24)	0.024	Yes	Yes	0.916	1.000	1.000
NOD2	rs2066847	3020insC/-	All	3	807/4078	carriers vs non-carriers	1.42(1.07-1.90)	0.017	0	0.593	×AC	Weak	340	×	A	C	First study		1.23(0.83-1.84)	0.310	NA	No	0.988	0.567	1.000
PDCD5	rs1862214	G/C	All	3	737/683	Allelic	1.32(1.12-1.56)	0.001	0	0.395	BAB	Moderate	971	B	A	B	Missing information		NA		None	No	0.156	1.000	0.296
PON1	rs662	A/G	All	3	995/834	Allelic	0.77(0.67-0.88)	2.02×10^{-4}	0	0.701	AAA	Strong	1569	A	A	A			0.76(0.65-0.89)	0.001	None	No	0.704	1.000	1.000
PROM1	rs2240688	C/A	Asian	3	2332/2457	Allelic	0.83(0.76-0.91)	6.92×10^{-5}	0	0.991	AAB	Moderate	2459	A	A	B	Missing information		NA		None	No	0.379	0.281	0.296
REV3L	rs462779	T/C	Asian	4	1937/2335	Allelic	1.11(1.02-1.22)	0.021	0	0.911	AAC	Strong	3433	A	A	C	Low OR	Highly consistent OR	1.13(1.00-1.27)	0.043	None	Yes	0.921	1.000	0.734
SFTPB	wild type/variation	NA	All	3	157/240	variation vs wild	1.92(1.11-3.33)	0.020	0	0.960	CAB	Weak	59	C	A	B	Missing information		NA		None	No	0.495	1.000	0.296
SOD2	rs4880	T/C	All	9	3738/4467	Allelic	1.20(1.06-1.36)	0.005	61	0.009	ACA	Weak	8801	A	C	A			1.21(1.03-1.43)	0.023	NO	No	0.723	0.733	0.348
TERT	rs2736098	A/G	All	7	4660/4825	Allelic	1.20(1.08-1.33)	0.001	67	0.006	ACB	Weak	6619	A	C	B	Missing information		1.19(1.05-1.35)	0.006	NA	No	0.734	0.706	0.548
TP53	rs12951053	G/T	All	3	475/569	Allelic	1.57(1.11-2.23)	0.011	37	0.203	BBB	Moderate	258	B	B	B	Missing information		NA		None	No	0.169	0.579	0.296
TP63	rs10937405	T/C	All	4	4927/8794	Allelic	0.87(0.81-0.94)	2.20×10^{-4}	34	0.207	ABA	Moderate	11355	A	B	A			0.75(0.62-0.89)	0.001	None	No	0.108	0.636	0.308
UGT1A6	rs6759892	G/T	All	3	266/261	Allelic	2.27(1.14-4.53)	0.020	84	0.002	BCA	Weak	346	B	C	A			3.21(2.21-4.67)	0.000	None	No	0.437	0.338	1.000
WWOX	CNV-67048	NA	Asian	4	2942/3074	0 copy vs 2 copies	2.06(1.58-2.70)	1.20×10^{-7}	0	0.911	BAB	Moderate	247	B	A	B	Missing information		NA		None	No	0.648	1.000	1.000
XRCC1	rs3213255	G/A	All	3	1089/1506	Allelic	1.21(1.08-1.35)	0.001	0	0.457	AAB	Moderate	2110	A	A	B	Missing information		NA		None	No	0.118	1.000	0.296
XRCC1	rs1001581	T/C	All	5	851/1166	Allelic	1.17(1.00-1.37)	0.044	28	0.232	ABC	Weak	1467	A	B	C	First study		1.18(0.97-1.44)	0.094	None	No	0.132	1.000	0.221
XRCC1	rs1799782	T/C	All	30	11096/13772	Allelic	0.90(0.82-0.98)	0.022	62	0.000	ACC	Weak	9151	A	C	C	HWE,low OR		0.90(0.82-0.99)	0.031	Yes	Yes	0.536	0.121	0.372
XRCC1	rs3213245	C/T	All	5	2795/2865	Allelic	1.29(1.04-1.59)	0.020	68	0.014	ACC	Weak	1413	A	C	C	First study		1.23(0.94-1.61)	0.138	None	No	0.539	0.661	0.806
**Associations identified from additional analyses under dominant or recessive genetic model**																									
ATM	rs664677	C/T	All	3	1627/1641	Dominant	0.76(0.64-0.92)	0.004	0	0.448	AAA	Strong	2692	A	A	A			0.73(0.59-0.91)	0.004	None	No	0.763	1.000	1.000
Chr8q24	rs16901979	A/C	All	3	1534/1992	Dominant	1.18(1.02-1.37)	0.025	0	0.610	AAC	Weak	1306	A	A	C	First study,HWE		1.13(0.95-1.34)	0.167	Yes	No	0.324	0.543	1.000
CYP1B1	rs10012	G/C	All	3	622/666	Dominant	1.69(1.05-2.72)	0.031	74	0.021	BCC	Weak	657	B	C	C	First study		1.47(0.58-3.75)	0.415	No	No	0.460	1.000	1.000
EGF	rs4444903	G/A	All	3	666/690	Dominant	2.07(1.01-4.24)	0.048	79	0.009	ACC	Weak	1141	A	C	C	First study,small study		1.55(0.79-3.05)	0.201	None	No	0.041	1.000	0.296
MLH1	rs1800734	A/G	All	5	2178/2320	Dominant	0.80(0.68-0.95)	0.009	24	0.260	AAC	Weak	3344	A	A	C	HWE		0.78(0.64-0.95)	0.013	Yes	No	0.583	0.605	0.462
PTGS2	rs689466	G/A	All	4	1676/2180	Dominant	0.78(0.62-0.97)	0.026	56	0.076	ACA	Weak	2198	A	C	A			0.72(0.54-0.98)	0.034	No	No	0.336	0.644	0.734
REV3L	rs465646	C/T	All	3	1296/1511	Dominant	0.78(0.67-0.92)	0.003	0	0.437	BAB	Moderate	888	B	A	B	Missing information		NA		None	No	0.423	1.000	1.000
CASC8	rs6983267	G/T	All	3	1539/1989	Recessive	1.22(1.04-1.44)	0.013	0	0.644	BAA	Moderate	802	B	A	A			1.30(1.06-1.59)	0.012	None	No	0.117	0.566	0.296
CHRNA5	rs142774214	ins/-	All	3	1431/1606	Recessive	0.80(0.65-0.98)	0.032	0	0.597	BAA	Moderate	432	B	A	A			0.78(0.62-0.98)	0.031	None	No	0.861	1.000	1.000
CYP2A6	non*4/*4	del/-(*4/non*4)	All	7	2623/2380	Recessive	0.51(0.35-0.73)	2.93×10^{-4}	0	0.539	BAA	Moderate	133	B	A	A			0.52(0.35-0.76)	0.001	No	No	0.826	0.654	1.000
FASLG	rs763110	T/C	All	5	4436/4120	Recessive	0.83(0.70-0.99)	0.038	30	0.221	ABC	Weak	1034	A	B	C	First study		0.86(0.70-1.05)	0.137	None	No	0.205	1.000	0.462

IL17A	rs2275913	A/G	All	3	889/998	Recessive	1.76(1.21-2.55)	0.003	18	0.295	BAB	Moderate	179	B	A	B	Missing information	2.00(1.41-2.83)	<0.001	NA	No	0.492	0.587	0.296
IL1B	rs1143627	C/T	All	8	4201/5431	Recessive	0.80(0.68-0.95)	0.010	49	0.059	ABC	Weak	1588	A	B	C	Small study,publication bias	0.83(0.70-0.98)	0.027	No	No	0.002	0.404	0.019
LIG1	rs156641	A/G	All	3	1112/2048	Recessive	1.45(1.14-1.83)	0.002	0	0.370	BAC	Weak	335	B	A	C	HWE	NA		Yes	No	0.217	1.000	1.000
XPA	rs1800975	A/G	All	12	4221/5240	Recessive	1.22(1.05-1.42)	0.011	33	0.124	ABA	Moderate	1463	A	B	A		1.23(1.04-1.45)	0.015	No	No	0.524	0.397	0.681
XRCC1	rs25487	A/G	All	48	16999/20567	Recessive	1.16(1.03-1.30)	0.018	54	0.000	ACC	Weak	3691	A	C	C	HWE,excess of significant studies	1.15(1.02-1.30)	0.022	Yes	No	0.428	0.006	0.729
XRCC3	rs1799794	G/A	All	4	1389/1941	Recessive	0.82(0.67-0.99)	0.038	0	0.469	BAC	Weak	571	B	A	C	First study	0.83(0.68-1.01)	0.058	No	No	0.712	0.490	1.000

Chr=chromosome; OR=odds ratio, 95%CI = 95% confidence interval; VNTR=variable number of tandem repeats; ins=insertion, del=deletion; NA=Not Applicable.

[a] Minor alleles/major alleles (per Caucasian)

[b] Summary ORs were calculated based on the DerSimonian-Lairs random-effects model

[c] *P*-value of heterogeneity test between studies

[d] Venice criteria grades contained assessments of three aspects: the amount of evidence, extent of replication, and protection from bias, and each assessment was assigned three levels (A, B, or C).

[e] Credibility of evidence was evaluated based on Venice criteria with three aspects (the amount of evidence, extent of replication, and protection from bias), and was categorized as strong (all three aspect grades were A), moderate (any grade was B, but not C) or weak (any grade was C).

[f] Amount of evidence, depending on total sample size of the smallest genetic group among cases and controls in each meta-analysis, was graded as A (sample size>1000), B (sample size between 100 and 1000), or C (sample size <100). One rare variant was not scored for amount of evidence (×), for this variant with frequency less than 0.5% and an A grade was unlikely to obtain.

[g] Extent of replication, depending on between-study heterogeneity, was graded as A (I^2<25%), B (I^2 between 25% and 50%), or C (I^2>50%).

[h] Protection from bias, considering various potential sources of bias in meta-analysis, was graded as A when there was no demonstrable bias and bias was unlikely invalidate the association, was graded as B when there was insufficient information for identifying evidence (i.e. missing information for evaluating Hardy-Weinberg equilibrium (HWE) among controls in an individual study) although there was no obvious bias, and C when the bias was evident and/or was likely to explain the presence of association. For the C grade, it was assigned if a meta-analysis had any of the following potential sources of bias: (1) magnitude of the association was low; (2) sensitivity analysis altered the statistical significance; (3) potential small study effect; (4) possible existence of an excess of significant findings (the number of observed significant studies more than the number of expected significant studies); (5) potential publication bias.

First study=Significance lost excluding the first published or first positive report; Low OR=Low magnitude of the association (i.e. 0.87<OR<1.15); Small study=Possible existence of potential small study effect (modified Egger's test, *p*<0.10); Excess of significant studies=Possible existence of an excess of significant findings (excess significance test, *p*<0.10); Publication bias= Possible existence of potential publication bias (Begg's test, *p*<0.10); Missing information= Insufficient information for bias evaluation; HWE=Significance lost excluding studies with controls violating HWE; Highly consistent OR=A meta-analysis with a low OR was not considered if there is a highly consistent OR across studies enrolled in this meta-analysis.

[i] A sensitivity analysis was performed to examine whether the significant summary ORs were robust after excluding the first published or first positive report (defined as the earliest study reported a significant association), or excluding studies with controls violating HWE. None=A meta-analysis didn't include studies with controls violating HWE or evaluated association for a variant with a phenotype change; NO=Without the influence from studies with controls violating HWE; NA=Insufficient information for evaluation of influence from first published/positive study or studies with controls violating HWE; Yes=Evidence of influence from studies with controls violating HWE.

[j] Low OR (i.e. 0.87<OR<1.15; No=Association without a low OR; Yes=Association with a low OR.)

[k] *P* value for potential small study effect based on the modified Egger's test. (*P*<0.10 is usually considered evidence for significant evidence of small study effect.)

[l] *P* value for possible existing an excess of significant findings based on the excess significance test. (*P*<0.10 is usually considered evidence for significant evidence of an excess of significant findings.)

[m] *P* value for potential publication bias of studies based on the Begg's test. (*P*<0.10 is usually considered evidence for significant evidence of publication bias.)

Supplementary Table S3. Subgroup meta-analyses stratified by ethnicity for genetic variants with available data (Continued on next three pages)

Gene	Variant	Alleles[a]	Meta-analysis performed in Caucasian population							Meta-analysis performed in Asian population						
			Studies	Cases/Controls	Frequency(%)[b]	Genetic models	OR (95% CI)	*P* value	I^2(%)	Studies	Cases/Controls	Frequency(%)[b]	Genetic models	OR (95% CI)	*P* value	I^2(%)
ABCB1	rs1045642(p.Ile1145Ile,c.3435T>C)	T/C														
ACE	287-bp repeat	287-bp ins/-														
AGER	rs1800624(-374T>A, Mun1)	A/T								3	1656/1693	34.41	Allelic	1.18(1.04-1.33)	0.010	16
AGER	rs1800625(-429T>C, Alu1)	C/T								3	1656/1693	31.19	Allelic	1.16(0.78-1.74)	0.468	91
AGER	rs2070600(p.Gly82Ser,c.244G>A)	A/G								3	1656/1693	38.36	Allelic	1.17(0.99-1.38)	0.067	52
AHR	rs2066853(Arg554Lys,Ex10+501G>A)	A/G								3	1382/1381	37.33	Allelic	0.99(0.86-1.14)	0.901	38
ALDH2	rs671(p.Glu487Lys/Glu504Lys,c.1510G>A)	A/G								3	1610/2059	NA	Dominant	0.88(0.61-1.26)	0.476	83
APEX1	rs1760944(c.-468T>G,-141T>G)	A/C								5	3071/3038	49.95	Allelic	1.20(1.12-1.29)	9.14×10^{-7}	0
APEX1	rs1048945(p.Gln51His,c.153G>C)	C/G	3	745/816	4.72	Allelic	0.93(0.67-1.31)	0.684	0							
APEX1	rs1130409(p.Asp148Glu,c.444T>G)	G/T	8	1807/3065	47.91	Allelic	0.94(0.85-1.04)	0.249	19	10	4220/5054	39.20	Allelic	1.02(0.92-1.12)	0.729	58
APEX1	rs2307486(p.Ile64Val,c.190A>G)	G/A								3	1941/2095	4.89	Allelic	0.90(0.73-1.11)	0.307	0
ATM	rs609429(IVS48+238C>G)	G/C														
ATM	rs664677(IVS21T-77C)	C/T														
ATM	rs189037(c.-111G>A,-4519G>A)	A/G								5	3036/3415	42.68	Allelic	1.09(1.00-1.18)	0.050	29
ATM	rs664143(IVS34G+60A)	G/A														
AURKA	rs2273535(p.Phe31Ile,c.91T>A)	A/T	3	2301/2069	48.28	Allelic	0.96(0.86-1.06)	0.421	0							
AURKA	rs1047972(p.Ile57Val,c.169A>G)	A/G	3	2463/2283	15.75	Allelic	0.96(0.86-1.07)	0.425	0							
AXIN2	rs2240308(p.Pro50Ser,c.148C>T)	T/C														
BCL2	rs2279115(c.-938C>A)	A/C								3	1537/2057	41.15	Allelic	0.76(0.51-1.13)	0.170	93
BIRC5	rs9904341(c.-31G>C）	C/G														
BRCA2	rs144848(p.Asn372His,c.1114A>C)	C/A														
CASC8	rs1447295(C>A)	A/C														
CASC8	rs6983267(T>G)	G/T														
CASP5	rs523104(p.Leu318Val,c.748C>G)	C/G														
CASP8	rs3834129(-652 6N del,-/CTTACT)	-/CTTACT								3	2301/2263	23.84	Allelic	0.88(0.72-1.07)	0.207	73
CASP8	rs1045485(p.Asp302His,c.853G>C)	C/G	3	758/964	10.79	Allelic	1.27(0.94-1.73)	0.125	45							
CCND1	rs678653(G>C)	C/G														
CCND1	rs9344(rs603965,p.Pro241Pro,c.723G>A)	A/G	6	10204/11646	48.88	Allelic	0.97(0.88-1.07)	0.517	62							
CD3EAP	rs967591(c.-21G>A)	A/G														
CDKN1A	rs1801270(p.Ser31Arg,c.93C>A,6829C>A)	A/C	7	3335/4082	7.06	Allelic	1.12(0.89-1.41)	0.340	62							
CDKN2A	rs3731249(p.Ala148Thr,c.442G>A)	A/G	3	2875/2707	1.75	Dominant	1.38(0.81-2.35)	0.230	56							
Chr8q24	rs16901979(C>A)	A/C														
CHRNA3	rs12914385(2594C>T)	T/C	3	4640/2157	37.00	Allelic	1.20(0.91-1.58)	0.195	82							
CHRNA3	rs3743073(A>C)	G/T														
CHRNA3	rs578776(C>T)	T/C														
CHRNA3	rs938682(T>C, T78896547C)	C/T														
CHRNA3	rs6495309(4064C>T)	T/C								3	2635/2767	47.83	Allelic	0.83(0.76-0.91)	6.17×10^{-5}	27
CHRNA5	rs142774214(rs3841324,-62ins/del,22bp indel)	22-bp ins/-														
CHRNA5	rs16969968(p.Asp398Asn, c.1192G>A)	A/G	6	3305/59780	33.76	Allelic	1.35(1.27-1.44)	2.03×10^{-21}	0	4	2811/2556	3.85	Allelic	0.78(0.33-1.85)	0.574	92
CLPTM1L	rs402710(C>T)	T/C	4	1801/1908	38.29	Allelic	0.86(0.78-0.94)	0.002	0	9	5413/6143	31.25	Allelic	0.89(0.82-0.97)	0.010	51
COMT	rs4680(p.Val158Met,c.472G>A)	A/G														
CRP	rs2808630(T>C)	C/T														
CTLA4	rs3087243(g.6230G>A,CT60)	A/G														
CTLA4	rs231775(p.Thr17Ala,c.49A>G)	G/A								3	2426/2403	67.23	Allelic	1.08(0.72-1.63)	0.712	95
CTLA4	rs5742909(g.319C>T,-318C>T)	T/C														
CXCL12	rs1801157(801G>A)	A/G														
CXCR2	rs1126579(C>T)	T/C														
CYP17A1	rs743572(T-34C,A1/A2)	G/A														
CYP1A1	rs1799814(m4,Thr461Asn,C>A)	A/C	4	1522/1585	5.93	Allelic	1.30(0.49-3.41)	0.598	94							
CYP1A1	rs1800031(m3,T5639C)	C/T														
CYP1A1	rs2606345(IVS1+606T>G)	G/T	3	2770/3609	34.44	Allelic	1.00(0.91-1.11)	0.937	35							
CYP1A1	rs4646903(m1,MspI,T3801C)	C/T	21	5083/6022	11.45	Allelic	1.12(1.03-1.21)	0.011	0	15	2418/3107	36.39	Allelic	1.18(1.01-1.38)	0.036	73
CYP1A1	rs1048943(*2C,m2, p.Ile462Val,c.1384A>G)	G/A	15	3375/3871	6.99	Allelic	1.27(1.02-1.59)	0.037	54	21	4265/5242	24.90	Allelic	1.17(1.02-1.33)	0.024	73
CYP1A2	rs2069514(CYP1A2*1C,-3860G>A)	A/G	3	761/1186		Dominant	1.06(0.50-2.25)	0.873	26							
CYP1A2	rs2069526(-739T>G)	G/T														
CYP1A2	rs35694136(-2467delT)	-2467delT/-														
CYP1A2	rs762551(CYP1A2*1F,c.A-164C)	C/A	3	869/1468	26.77	Allelic	1.38(1.12-1.71)	0.002	57							
CYP1B1	rs10012(p.Arg48Gly,c.142C>G)	G/C														
CYP1B1	rs1056827(p.Ala119Ser,c.355G>T, m2)	T/G	3	975/1684	29.42	Allelic	0.99(0.85-1.16)	0.904	21							
CYP1B1	rs1800440(p.Asn453Ser,Ex3+315A>G)	G/A	4	2809/3687	19.01	Allelic	0.95(0.81-1.11)	0.530	47							
CYP1B1	rs1056836(CYP1B1*3,p.Leu432Val,c.1294C>G)	G/C	6	1849/2655	43.71	Allelic	1.12(1.02-1.22)	0.013	0	3	770/764	13.68	Allelic	1.07(0.82-1.39)	0.633	37
CYP2A13	rs8192789(p.Arg257Cys,3375C>T)	T/C														
CYP2A6	non*4/*4(*1/*4, a deletion of the CYP2A6 gene)	del/- (*4/non*4)								6	2517/2264	14.66	Allelic	0.81(0.64-1.03)	0.089	71
CYP2A6	rs1801272(p.Leu160His,Ex3-15T>A)	A/T	3	2411/2644	3.99	Dominant	0.66(0.52-0.84)	0.001	0							
CYP2A6	rs5031016(p.Ile471Thr,c1412T>C,wt/*7)	C/T														
CYP2C9	rs1799853(p.Arg144Cys,c.430C>T)	T/C	3	604/1054		Dominant	1.19(0.85-1.68)	0.319	48							
CYP2D6	phenotype (EM/HEM/PM)	NA	8	1327/1787	5.54	PM vs EM	0.90(0.60-1.34)	0.595	7							
CYP2D6	rs1065852(p.Pro34Ser,188C>T)	C/T														
CYP2E1	rs2031920(RsaI, c1>c2, -1053C>T)	T/C	6	665/1224	4.90	Allelic	0.61(0.42-0.90)	0.013	0	11	3370/3951	23.40	Allelic	0.89(0.77-1.04)	0.152	66
CYP2E1	rs3813867(g.3739G>C,c.-1293G>C)	C/G														
CYP2E1	rs6413432(c.967+1143T>A,7632T>A)	A/T	4	441/538	11.52	Allelic	0.79(0.58-1.06)	0.121	0	6	1964/2085	26.55	Allelic	0.78(0.70-0.86)	1.31×10^{-6}	0
CYP3A4	rs2740574(-392A>G,*1A/*1B)	G/A	3	1665/1979		Dominant	1.08(0.84-1.39)	0.561	6							
CYP3A5	rs776746(A6986G,CYP3A5*3,22892A>G)	A/G														
DRD2	rs1079597(G>A,TaqI-)	A/G	3	461/537		Dominant	1.04(0.79-1.37)	0.781	0							
DRD2	rs1800497(p.Glu714Lys,A2/A1,C>T)	C/T	3	510/621		Recessive	1.00(0.69-1.44)	0.986	0							
EGF	rs4444903(+61A>G)	G/A														
ELANE	rs351107(-903T>G,Rep_a)	G/T	3	745/762	5.31	Allelic	0.55(0.34-0.87)	0.011	29							
ELANE	rs7254054(-741G>A,Rep_b)	A/G	3	754/750	27.20	Allelic	0.77(0.61-0.97)	0.030	46							

Gene	Variant	Alleles[a]	Meta-analysis performed in Caucasian population							Meta-analysis performed in Asian population						
			Studies	Cases/Controls	Frequency(%)[b]	Genetic models	OR (95% CI)	*P* value	I^2(%)	Studies	Cases/Controls	Frequency(%)[b]	Genetic models	OR (95% CI)	*P* value	I^2 (%)
EPHX1	rs1051740(p.Tyr113His,c.337T>C)	C/T	12	2560/5862	31.06	Allelic	0.88(0.74-1.04)	0.123	77							
EPHX1	rs2234922(p.His139Arg,c.416A>G)	G/A	13	4410/7749	18.91	Allelic	1.12(0.94-1.34)	0.195	79							
ERCC1	rs11615(rs3177700, p.Asn118Asn,c.354T>C)	C/T	7	3544/4862	39.68	Allelic	0.89(0.79-1.01)	0.064	60	5	2187/2196	76.66	Allelic	0.91(0.79-1.06)	0.224	49
ERCC1	rs16979802(15310G>C)	C/G	3	1311/2114	21.81	Allelic	0.87(0.74-1.03)	0.102	0							
ERCC1	rs2298881(262G>T, C>A)	T/G								3	2305/2315	42.07	Allelic	1.00(0.90-1.11)	0.986	33
ERCC1	rs3212948(C>G)	G/C	3	1034/1446	42.98	Allelic	1.01(0.84-1.20)	0.960	55							
ERCC1	rs3212955(A>G)	G/A	3	1287/1857	24.21	Allelic	1.05(0.93-1.20)	0.415	9							
ERCC1	rs3212961(IVS5+33C＞A,17677C>A)	A/C	3	771/883	14.84	Allelic	0.93(0.77-1.13)	0.485	0	3	1436/1428	48.28	Allelic	0.96(0.82-1.12)	0.584	37
ERCC1	rs3212986(8092C>A,14443C>A)	A/C	4	3091/3489	25.24	Allelic	1.00(0.92-1.08)	0.986	0							
ERCC1	rs735482(p.Lys261Thr,A>C)	C/A	3	1287/2365	14.63	Allelic	0.94(0.82-1.08)	0.390	0							
ERCC2	rs1052555(Asp711Asp,G>A)	A/G														
ERCC2	rs171140(A>C)	C/A														
ERCC2	rs13181(p.Lys751Gln,c.2251A>C)	C/A	19	5967/8851	36.08	Allelic	1.10(1.03-1.18)	0.003	32	15	5878/6060	7.90	Allelic	1.12(0.95-1.33)	0.170	61
ERCC2	rs1799787(IVS19-70C＞T,G>A)	T/C								3	688/756	7.34	Allelic	0.90(0.51-1.62)	0.733	74
ERCC2	rs1799793(p.Asp312Asn,c.981G>A)	A/G	15	5165/7680	34.91	Allelic	1.02(0.96-1.08)	0.542	11	10	4797/5010	5.76	Allelic	1.00(0.79-1.28)	0.976	68
ERCC2	rs238406(p.Arg156Arg,C>A)	A/C	3	1150/2080	42.76	Allelic	1.13(1.01-1.25)	0.026	0							
ERCC4	rs1799801(p.Ser835Ser,Ex11-247T＞C)	C/T														
ERCC4	rs1800067(Arg415Gln)	A/G	5	1521/2420	6.78	Allelic	1.10(0.92-1.32)	0.286	0							
ERCC5	rs1047768(p.His46His,335C>T)	T/C	3	1331/2136	42.51	Allelic	0.86(0.72-1.04)	0.114	63							
ERCC5	rs17655(p.Asp1104His,c.3310G>C)	C/G	4	1249/2203	22.95	Allelic	0.95(0.78-1.14)	0.570	47	5	2024/2683	56.71	Allelic	0.90(0.75-1.08)	0.248	71
ERCC6	rs3793784(-6530C>G)	G/C														
ERCC6	rs2228528(p.Gly399Asp,G1275A)	T/C								3	1713/1887	43.91	Allelic	1.03(0.92-1.17)	0.591	31
EXO1	rs1047840(p.Glu589Lys,c.1765G>A)	A/G														
FANCG	rs2237857(p.Thr297Ile,C1382T)	T/C														
FAS	rs2234767(-1377G/A)	A/G								3	1857/2149	36.81	Allelic	1.12(0.94-1.35)	0.216	72
FASLG	rs763110(-844C/T)	T/C								3	1857/2149	30.97	Allelic	0.77(0.58-1.01)	0.063	86
FGFR4	rs351855(p.Gly388Arg,G>A)	A/G														
GPC5	rs2352028(C>T)	T/C								3	1648/2002	21.65	Allelic	0.97(0.87-1.09)	0.611	0
GPX1	rs1050450(p.Pro200Leu,c.599C>T)	T/C	4	976/1339	34.06	Allelic	1.04(0.75-1.44)	0.811	81							
GSTM1	Present/Null(Large gene deletion)	NA	58	16976/20622	51.28	Null vs present	1.08(1.03-1.13)	0.001	7	36	11012/9982	50.56	Null vs presrnt	1.27(1.14-1.41)	9.06×10^{-6}	65
GSTM3	rs1799735(c.468+21delAGG,3bp deletion,*A/*B)	delAGG/-	6	1427/1613	15.25	Allelic	1.00(0.87-1.15)	0.956	0							
GSTP1	rs1138272(p.Ala114Val,c.341C>T)	T/C	6	1479/1573	7.63	Allelic	1.24(0.93-1.65)	0.149	53							
GSTP1	rs1695 (p.Ile105Val, c.313A>G)	G/A	26	9151/10531	32.85	Allelic	1.04(0.98-1.09)	0.224	29	11	2307/2430	18.87	Allelic	1.14(1.01-1.29)	0.036	27
GSTT1	Present/Null(Large gene deletion)	NA	38	12560/15525	18.88	Null vs present	1.01(0.92-1.10)	0.910	45	14	7043/5289	49.57	Null vs presrnt	1.15(1.03-1.28)	0.010	34
HIF1A	rs11549465(p.Pro582Ser,c.1744C>T)	T/C														
HIF1A	rs11549467(p.Ala588Thr, c.1762G>A)	A/G														
HRAS1	common/rare (HRAS1 VNTR alleles, loss of heterozygosity in the 15p15.5 region)	NA	4	746/1174	7.03	rare vs common	2.55(1.01-6.45)	0.048	69							
HYKK	rs931794(A>G)	G/A														
IFNG	rs2069705(-1615T>C)	C/T														
IGFBP3	rs2854744(C-202A)	C/A								3	789/960	27.71	Allelic	0.79(0.52-1.20)	0.262	86
IL10	rs1800871(c.-854T>C,-819C>T)	T/C	5	1741/2296	6.82	Allelic	1.03(0.81-1.31)	0.820	0	4	959/1367	69.09	Allelic	0.84(0.54-1.32)	0.447	91
IL10	rs1800872(g.4433A>C,-592C>A)	A/C	3	2368/2869	22.85	Allelic	0.98(0.89-1.08)	0.677	0	3	842/1257	66.31	Allelic	0.76(0.62-0.93)	0.009	56
IL10	rs1800896(g.3943A>G,-1082A>G)	G/A	6	2190/2729	47.62	Allelic	1.08(0.92-1.27)	0.347	49	3	627/1029	10.11	Allelic	1.83(0.75-4.49)	0.188	88
IL13	rs1800925(-1069C>T,-1112C>T)	T/C														
IL13	rs20541(Arg130Gln,+2044G>A)	A/G														
IL17A	rs2275913(-73G>A,-152G>A)	A/G														
IL1B	rs1143634(p.Phe105Phe,Ex5+14C>T)	T/C	3	4045/3627	23.55	Allelic	1.02(0.94-1.10)	0.666	0							
IL1B	rs1143627(g.4970C>T,-31T>C)	C/T	4	3681/4142	33.23	Allelic	0.98(0.87-1.10)	0.757	60	3	1352/1906	48.87	Allelic	0.89(0.71-1.10)	0.277	75
IL1B	rs16944(C-511T)	T/C								7	2391/2858	47.10	Allelic	0.92(0.78-1.09)	0.343	75
IL4	rs2243250(-509C>T)	T/C								3	1691/1737	78.12	Allelic	1.18(0.88-1.59)	0.276	76
IL6	rs1800795(G-174C)	C/G	8	4385/5232	43.43	Allelic	1.01(0.95-1.07)	0.721	0							
IL6	rs1800796(-572C>G,634C>G)	G/C								8	2782/3195	22.35	Allelic	1.04(0.83-1.31)	0.713	82
IL8	rs4073(T-251A)	T/A	4	4308/4773	49.36	Allelic	1.00(0.94-1.06)	0.973	0							
LIG1	rs156641(G>A)	A/G	3	1000/1940	34.48	Allelic	1.09(0.90-1.32)	0.396	44							
LIG1	rs20579(-7C>T)	A/G	3	1140/2079	12.84	Allelic	1.25(0.84-1.86)	0.279	80							
LIG1	rs20581(p.Asp802Asp,G>A)	A/G														
LIG1	rs3730931(IVS9-21A>G)	G/A	3	1145/2084	12.00	Allelic	1.15(0.78-1.70)	0.479	77							
LIG1	rs439132(A>G)	G/A														
LIG1	rs20580(p.Ala170Ala,c.417C>A)	A/C	3	1412/2214	48.85	Allelic	1.01(0.88-1.17)	0.876	47							
LIG4	rs1805388(p.Thr9Ile,Ex2+54C>T)	T/C														
LTA	rs909253(A+252G)	G/A	4	234/596	31.12	Allelic	1.10(0.87-1.41)	0.428	0							
MAPKAPK2	CNV-30450(2copies/3copies/4copies)	NA								3	2332/2480	9.76	4 copies vs 2 copies	1.60(1.04-2.45)	0.031	81
MBD4	rs140693(p.Glu346Lys,G1212A)	A/G								4	2295/2469	35.62	Allelic	0.99(0.86-1.13)	0.881	57
MCP1	rs1024611(-2518A>G,PvuII)	G/A														
MDM2	rs117039649(G>C,SNP285)	C/G														
MDM2	rs2279744(SNP309,c.14+309T>G)	G/T	7	5541/7830	37.31	Allelic	1.00(0.93-1.08)	0.979	45	8	5013/5834	49.07	Allelic	1.13(1.03-1.24)	0.011	64
MDM2	rs769412(p.Glu184Glu,2164A>G,SNP354)	G/A	3	1574/1715	29.53	Allelic	1.01(0.90-1.13)	0.889	0							
MGMT	rs12268840(C>T)	T/C	3	247/355	32.82	Allelic	0.88(0.64-1.19)	0.396	24							
MGMT	rs12917(p.Leu115Phe,c.343C>T)	T/C	5	1930/3022	14.36	Allelic	1.08(0.94-1.24)	0.292	14							
MGMT	rs1803965(Leu53Leu,171C>T,TaqI)	T/C														
MGMT	rs2308321(p.Ile174Val,Ile143Val,c.520A>G)	G/A	5	1942/2110	12.68	Allelic	1.02(0.85-1.23)	0.809	33							
MGMT	rs2308327(p.Lys178Arg,c.533A>G)	G/A	5	759/880	20.45	Allelic	0.73(0.50-1.07)	0.109	68							
MIR146A	rs2910164(C>G)	C/G								4	2807/2841	49.03	Allelic	1.12(1.04-1.21)	0.002	0
MIR196A2	rs11614913(T>C)	C/T								4	2376/2413	45.86	Allelic	1.14(1.05-1.23)	0.002	0
MLH1	rs1799977(p.Ile219Val,c.655A>G)	G/A								3	1713/1874	3.98	Allelic	1.03(0.59-1.79)	0.927	69
MLH1	rs1800734(-93G>A)	A/G								4	1756/1809	57.19	Allelic	1.03(0.86-1.23)	0.745	69
MLH3	rs175080(p.Pro844Leu,C2645T)	T/C														
MMP1	rs1799750(-16071G/2G,insG)	2G/1G	6	3607/3073	49.40	Allelic	1.05(0.89-1.23)	0.580	74							
MMP2	rs243865(-1306C>T)	T/C														
MMP3	rs3025058(-16125A/6A,-11715A/6A)	5A/6A	3	2759/2021	48.74	Allelic	1.02(0.93-1.10)	0.726	0							
MMP9	rs3918242(g.3430C>T,-1562C>T)	T/C														

Gene	Variant	Alleles[a]	Meta-analysis performed in Caucasian population							Meta-analysis performed in Asian population						
			Studies	Cases/Controls	Frequency(%)[b]	Genetic models	OR (95% CI)	*P* value	I^2(%)	Studies	Cases/Controls	Frequency(%)[b]	Genetic models	OR (95% CI)	*P* value	I^2(%)
MPO	rs2333227(G-463A)	A/G	16	7248/8119	23.27	Allelic	0.93(0.85-1.03)	0.152	60	8	2030/2185	11.95	Allelic	0.85(0.69-1.04)	0.112	50
MSH3	rs26279(p.Ala1045Thr,c.3133G>A)	G/A														
MSH6	rs1042821(p.Gly39Glu,c.116G>A)	A/G														
MTHFR	rs1801131(p.Ala429Glu,c.1286A>C,A1298C)	C/A	5	2127/2179	31.30	Allelic	1.10(1.00-1.20)	0.043	0	5	1946/2740	19.20	Allelic	1.04(0.94-1.16)	0.442	0
MTHFR	rs1801133(p.Ala222Val,Ex4+79C＞T)	T/C	7	2219/3028	31.46	Allelic	0.99(0.81-1.23)	0.953	72	8	6421/5172	42.56	Allelic	1.10(0.96-1.25)	0.186	77
MTR	rs1805087(Asp919Gly,A2756G)	G/A								3	1453/2187	13.33	Allelic	1.13(0.97-1.32)	0.128	16
MTRR	rs1801394(Ile49Met,A66G)	G/A														
MUTYH	rs3219489(p.Gln324His,c.972G>C)	C/G														
MYCL	L/S(EcoRI)	NA	4	202/548	49.36	S vs L	1.03(0.81-1.30)	0.821	0							
NAT2	Rapid/slow (Acetylation Phenotype)	NA	11	2148/2887	64.39	Slow vs rapid	1.06(0.91-1.23)	0.482	28	6	1599/1965	24.73	Slow vs rapid	0.98(0.66-1.46)	0.915	79
NAT2	rs1799929(p.Leu161Leu,481C>T)	T/C														
NAT2	rs1799930(p.Arg197Gln,590G>A,NAT2*6B)	A/G	4	964/936		Dominant	0.88(0.64-1.21)	0.422	61							
NBN	rs1805794(p.Gln185Glu,Ex5-32C>G,605C>G)	G/C	3	688/1528	39.95	Allelic	1.00(0.86-1.16)	0.989	0	5	2890/3160	54.73	Allelic	0.93(0.73-1.19)	0.556	90
NFKB1	rs28362491(-94ins/delATTG)	-94insATTG/-														
NOD2	rs2066847(3020insC,WT/insC,L1007fs)	3020insC/-														
NQO1	rs1800566(p.Pro187Ser,c.648C>T,609C>T,*2)	T/C	10	3181/3582	22.79	Allelic	1.06(0.97-1.15)	0.231	0	8	1482/2325	42.37	Allelic	0.92(0.73-1.16)	0.492	81
OGG1	rs1052133(p.Ser326Cys,c.977C>G,8055C>G)	G/C	11	4089/5931	22.05	Allelic	0.98(0.85-1.14)	0.827	69	18	7311/7683	53.05	Allelic	1.06(0.99-1.13)	0.080	42
PADPRP	A/B(a193-bp deletion)	193-bp del/-	3	217/213		Dominant	1.02(0.41-2.53)	0.965	75							
PARP1	rs1136410(p.Val762Ala,Ex17+8T＞C)	C/T								5	2900/2567	43.83	Allelic	1.03(0.89-1.19)	0.724	66
PCNA	rs25406(2232C>T)	T/C	3	746/854	40.11	Allelic	0.95(0.80-1.14)	0.597	35							
PDCD5	rs1862214(G>A)	G/C														
POLD1	rs1726801(p.Arg119His,c.356G>A)	A/G								3	1263/1420	19.30	Allelic	1.11(0.96-1.28)	0.168	0
POLI	rs3730668(-78G>T)	T/G														
POLI	rs8305(Thr706Ala,2180A>G)	G/A														
POLL	rs3730477(p.Arg438Trp,1683C>T)	T/C														
PON1	rs662(p.Gln192Arg,c.575A>G)	A/G														
PPARG	rs1801282(p.Pro12Ala,c.36C>G)	G/C	3	2178/2659	18.11	Allelic	1.00(0.89-1.14)	0.961	0							
PPP1R13L	rs6966(T>A)	T/A														
PPP1R13L	rs1970764(IVS1-4364A>G)	G/A														
PROM1	rs2240688(A>C)	C/A								3	2332/2457	27.37	Allelic	0.83(0.76-0.91)	6.92×10^{-5}	0
PTGS2	rs20417(-765G>C)	C/G														
PTGS2	rs689466(g.38139393T>C,-1195A>G)	G/A														
PTGS2	rs5275(c.*427T>C,8473T>C)	C/T	5	4378/4847	35.47	Allelic	1.06(0.85-1.33)	0.590	91	5	2245/2941	20.10	Allelic	0.87(0.74-1.03)	0.097	60
RAD23B	rs1805329(p.Ala249Val,Ex7+65C＞T)	T/C	3	535/767	23.79	Allelic	0.97(0.73-1.28)	0.815	51							
REV1	rs3087386(p.Phe257Ser,892T>C)	C/T								3	1437/2503	33.72	Allelic	1.03(0.93-1.14)	0.544	0
REV3L	rs462779(p.Thr1146Ile,4259C>T)	T/C								4	1937/2335	39.36	Allelic	1.11(1.02-1.22)	0.021	0
REV3L	rs465646(460T>C)	C/T								3	1296/1511	18.73	Allelic	0.83(0.71-0.97)	0.016	14
SFTPB	wildtype/variation(deletions or insertions)	NA	3	157/240	5.83	Variation vs wild	1.92(1.11-3.33)	0.020	0							
SOD2	rs4880(rs1799725,Ala16Val)	T/C	4	3185/3966	48.20	Allelic	1.17(1.10-1.25)	2.24×10^{-6}	0	3	551/683	NA	Recessive	1.06(0.82-1.37)	0.671	0
SULT1A1	rs9282861(p.Arg213His,c.638G>A,*1/*2)	A/G	3	865/1152	29.95	Allelic	1.11(0.92-1.35)	0.275	44							
TDG	rs4135113(p.Gly199Ser,c.595G>A)	A/G														
TERT	rs2736098(p.Ala305Ala,c.915G>A)	A/G								6	3829/3992	35.11	Allelic	1.20(1.06-1.37)	0.004	72
TGFB1	rs1800469(C-509T)	T/C								4	1091/1238	49.31	Allelic	0.95(0.85-1.07)	0.428	3
TGFB1	rs1800470(p.Leu10Pro,c.29C>T)	C/T								4	935/1031	49.13	Allelic	1.03(0.91-1.17)	0.668	0
TNF	rs1799724(-857C>T,-1036C>T)	C/T														
TNF	rs1800629(G-308A,*1/*2)	A/G	8	2576/3091	16.68	Allelic	0.97(0.86-1.10)	0.668	10							
TNF	rs361525(A-417G,-238G>A)	A/G	3	575/584	3.94	Allelic	1.05(0.67-1.65)	0.834	0							
TNFRSF10A	rs2230229(p.Arg441Lys,c.1322G>A)	G/A	4	1183/1637	14.87	Allelic	1.01(0.71-1.44)	0.948	72							
TP53	rs1042522(p.Pro72Arg,c.215C>G)	C/G	23	10470/12364	26.37	Allelic	1.06(0.98-1.14)	0.178	63	18	10520/9977	40.03	Allelic	1.08(0.99-1.17)	0.068	69
TP53	rs12951053(IVS7+92T>G)	G/T														
TP53	rs1625895(IVS6+62G>A,A2>A1)	A/G	5	2382/2504	12.68	Allelic	1.17(0.93-1.49)	0.185	67							
TP53	rs1642785(IVS2+38G>C,1827G>C)	C/G								3	132/107	53.74	Allelic	0.82(0.57-1.17)	0.272	0
TP53	rs17878362(IVS3 16bp Del/Ins)	16-bp ins/-	4	4196/4327	12.97	Allelic	1.13(0.96-1.34)	0.152	64							
TP53BP1	rs560191(p.Asp353Glu,c.1059C>G）	G/C														
TP63	rs10937405(C>T)	T/C														
TP73	G4C14/A4T14	A4T14/G4C14								6	2000/2172	24.19	Allelic	0.99(0.81-1.21)	0.942	73
TYMS	28-bp tandem repeat	NA														
UGT1A6	rs1105879(p.Arg184Ser,c.552A>C)	G/T														
UGT1A6	rs6759892(p.Ser7Ala,c.19T>G)	G/T														
VEGFA	rs2010963(634G>C)	C/G														
VEGFA	rs3025039(C936T)	T/C								3	753/754	17.31	Allelic	1.10(0.84-1.45)	0.487	43
VEGFA	rs699947(C-2578A)	A/C								3	735/660	22.95	Allelic	0.97(0.75-1.25)	0.829	43
VEGFA	rs833061(-460T>C)	C/T								3	967/930	29.62	Allelic	0.85(0.68-1.05)	0.123	52
vWF	rs9295740(G>A)	A/G														
WWOX	CNV-67048(2copies/1copy/0copy)	NA								4	2942/3074	2.86	0 copy vs 2 copies	2.06(1.58-2.70)	1.20×10^{-7}	0
XPA	rs1800975(c.-4A>G,23G>A)	A/G	9	3294/4385	35.01	Allelic	1.05(0.94-1.16)	0.404	53							
XPC	PAT-/+(24660_24664delGTAAC, inspoly(AT))	inspoly(AT)/-														
XPC	rs2228000(p.Ala499Val,Ex9-377C>T)	T/C								5	2238/2062	31.13	Allelic	1.06(0.95-1.18)	0.310	23
XPC	rs2228001(p.Gln939Lys,Ex16+211A＞C)	C/A	6	1919/3265	37.81	Allelic	1.05(0.97-1.15)	0.236	7	8	3696/3729	37.92	Allelic	1.03(0.96-1.10)	0.401	0
XRCC1	rs1001581(c.145-216G>A)	T/C														
XRCC1	rs1799782(p.Arg194Trp,c.580C>T,Ex6-22C＞T)	T/C	12	4740/6868	6.96	Allelic	0.84(0.72-0.98)	0.028	28	15	5844/6259	31.07	Allelic	0.92(0.81-1.03)	0.152	74
XRCC1	rs2030404(A>G)	G/A														
XRCC1	rs2256507(G>A)	A/G														
XRCC1	rs25486(A>G)	G/A														
XRCC1	rs25487(p.Gln399Arg,c.1196A>G)	A/G	18	7846/10215	34.51	Allelic	1.00(0.95-1.05)	0.958	11	22	8845/9585	25.49	Allelic	1.04(0.96-1.14)	0.344	65
XRCC1	rs25489(p.Arg280His,c.839G>A,Ex9+16G>A)	A/G	9	4038/4481	5.27	Allelic	1.06(0.87-1.29)	0.594	36	9	3784/3993	10.32	Allelic	0.92(0.70-1.19)	0.513	81
XRCC1	rs2682562(A>G)	G/A														
XRCC1	rs2682585(p.His6Arg,c.17A>G)	A/G														
XRCC1	rs2854510(A>G)	G/A														
XRCC1	rs3213255(A>G)	G/A														
XRCC1	rs3213403(A>G)	G/A														

Gene	Variant	Alleles[a]	Meta-analysis performed in Caucasian population							Meta-analysis performed in Asian population						
			Studies	Cases/Controls	Frequency(%)[b]	Genetic models	OR (95% CI)	*P* value	I^2(%)	Studies	Cases/Controls	Frequency(%)[b]	Genetic models	OR (95% CI)	*P* value	I^2 (%)
XRCC1	rs3547(p.Gln632Gln,c.1896G>A)	A/G														
XRCC1	rs3213245(-77T>C)	C/T								4	2686/2755	9.87	Allelic	1.33(1.04-1.69)	0.021	74
XRCC2	rs3218536(p.Arg188His,c.563G>A)	A/G	3	645/1373	8.08	Allelic	1.23(0.54-2.77)	0.625	88							
XRCC3	rs1799794(4541A>G)	G/A														
XRCC3	rs1799796(17893A>G,IVS5-14)	G/A	3	665/1672	31.19	Allelic	0.98(0.85-1.14)	0.827	0							
XRCC3	rs861539(p.Thr241Met,c.722C>T)	T/C	9	2271/3339	36.63	Allelic	0.97(0.90-1.06)	0.541	0	7	3805/4330	6.91	Allelic	1.20(0.98-1.47)	0.072	63

Chr=chromosome; OR=odds ratio, 95%CI = 95% confidence interval; bp=base pair; VNTR=variable number of tandem repeats; ins=insertion; NA = Not Applicable.

[a] Minor alleles/major alleles (per Caucasian).

[b] Frequency of minor allele or effect genotype(s) in controls in specific ethnicity.

Supplementary Table S4. Credibility assessment for variants with significant associations with lung cancer risk in subgroup meta-analyses by ethnicity

Genes	Variants	Alleles[a]	Sub-group	Number evaluated		Lung cancer risk meta-analysis					Venice criteria grades[d]	Credibility of evidence[e]	Amount of evidence[f]		Replication[g]	Protection from bias[h]			Sensitivity analysis[i]: Initial study influence		Sensitivity analysis[i]: Deviation from HWE	Low OR[j]	Modified P^k	Excess P^l	Begg's P^m
				Studies	Cases/Controls	Genetic models	OR(95%CI)[b]	*P* value	I^2 (%)	P_Q[c]			Numbers	Grade		Grade	Reasons for bias	Reason for bias exemption	OR(95%CI)	*P* value					
APEX1	rs1130409	G/T	Caucasian	7	1807/3065	Recessive	0.84(0.72-0.97)	0.021	0	0.695	AAA	Strong	1119	A	A	A			0.81(0.68-0.95)	0.011	No	No	0.719	0.573	0.764
CHRNA5	rs16969968	A/G	Caucasian	6	3305/59780	Allelic	1.35(1.27-1.44)	2.03×10^{-21}	0	0.958	AAA	Strong	43126	A	A	A			1.35(1.26-1.44)	0.000	None	No	0.963	1.000	0.990
CLPTM1L	rs402710	T/C	Caucasian	4	1801/1908	Allelic	0.86(0.78-0.94)	0.002	0	0.532	AAA	Strong	2717	A	A	A			0.87(0.78-0.97)	0.010	None	No	0.678	1.000	0.734
CYP1A1	rs4646903	C/T	Caucasian	21	5083/6022	Allelic	1.12(1.03-1.21)	0.011	0	0.779	AAC	Weak	2633	A	A	C	Low OR,small study,publication bias		1.12(1.03-1.22)	0.011	None	Yes	0.002	1.000	0.002
CYP1A1	rs1048943	G/A	Caucasian	15	3375/3871	Allelic	1.27(1.02-1.59)	0.037	54	0.006	ACC	Weak	1088	A	C	C	Excess of significant studies		1.27(1.00-1.60)	0.050	None	No	0.264	0.091	0.295
CYP1A2	rs762551	C/A	Caucasian	3	869/1468	Recessive	1.69(1.20-2.36)	0.002	30	0.232	BBA	Moderate	237	B	B	A			1.84(1.13-3.00)	0.015	None	No	0.504	1.000	1.000
CYP1B1	rs1056836	G/C	Caucasian	6	1849/2655	Dominant	1.18(1.04-1.34)	0.010	0	0.856	AAB	Moderate	3408	A	A	B	Missing information		1.16(1.02-1.33)	0.026	NA	No	0.822	0.607	0.711
CYP2A6	rs1801272	A/T	Caucasian	3	2411/2644	carriers vs non-carriers	0.66(0.52-0.84)	0.001	0	0.674	BAB	Moderate	307	B	A	B	Missing information		0.65(0.51-0.83)	0.001	NA	No	0.929	1.000	1.000
CYP2E1	rs2031920	T/C	Caucasian	6	665/1224	Allelic	0.61(0.42-0.90)	0.013	0	0.456	BAB	Moderate	160	B	A	B	Missing information		0.57(0.39-0.85)	0.006	NA	No	0.767	0.588	0.837
ELANE	rs351107	G/T	Caucasian	3	745/762	Allelic	0.55(0.34-0.87)	0.011	29	0.246	BBC	Weak	126	B	B	C	First study		0.57(0.24-1.38)	0.213	None	No	0.794	0.565	1.000
ELANE	rs7254054	A/G	Caucasian	3	754/750	Allelic	0.77(0.61-0.97)	0.030	46	0.155	BBC	Weak	735	B	B	C	First study,small study		0.84(0.53-1.32)	0.444	NA	No	0.022	0.567	0.296
ERCC2	rs238406	A/C	Caucasian	3	1150/2080	Allelic	1.13(1.01-1.25)	0.026	0	0.457	AAC	Weak	2826	A	A	C	First study,HWE,low OR		1.10(0.98-1.24)	0.110	Yes	Yes	0.158	1.000	0.157
ERCC2	rs13181	C/A	Caucasian	18	5967/8851	Recessive	1.15(1.04-1.29)	0.009	16	0.258	AAA	Strong	2209	A	A	A			1.15(1.03-1.29)	0.017	No	No	0.191	0.206	0.495
GSTM1	Present/Null	NA	Caucasian	58	16976/20622	Allelic	1.08(1.03-1.13)	0.001	7	0.332	AAC	Weak	19614	A	A	C	Low OR		1.08(1.03-1.13)	0.001	None	Yes	0.452	0.383	0.448
HRAS1	common/rare (HRAS1 VNTR)	NA	Caucasian	4	746/1174	rare vs common	2.55(1.01-6.45)	0.048	69	0.023	BCC	Weak	305	B	C	C	First study		2.02(0.66-6.21)	0.221	None	No	0.561	0.346	0.734
MTHFR	rs1801131	C/A	Caucasian	5	2127/2179	Allelic	1.10(1.00-1.20)	0.043	0	0.862	AAC	Weak	2814	A	A	C	HWE,low OR	Highly consistent OR	1.13(1.01-1.25)	0.029	Yes	Yes	0.800	1.000	0.797
SFTPB	wild type/variation	NA	Caucasian	3	157/240	Allelic	1.92(1.11-3.33)	0.020	0	0.960	CAC	Weak	59	C	A	C	Publication bias		NA		None	No	0.495	1.000	0.049
SOD2	rs4880	T/C	Caucasian	4	3185/3966	Allelic	1.17(1.10-1.25)	2.24×10^{-6}	0	0.973	AAA	Strong	7146	A	A	A			1.17(1.08-1.27)	0.000	None	No	0.407	1.000	0.406
XRCC1	rs1799782	T/C	Caucasian	12	4740/6868	Allelic	0.84(0.72-0.98)	0.028	28	0.172	ABA	Moderate	1510	A	B	A			0.84(0.72-0.99)	0.039	No	No	0.927	0.636	0.790
AGER	rs1800624	A/T	Asian	3	1656/1693	Additive	1.18(1.04-1.33)	0.010	16	0.305	AAC	Weak	2292	A	A	C	First study		1.20(0.90-1.60)	0.215	None	No	0.480	1.000	1.000
APEX1	rs1760944	A/C	Asian	5	3071/3038	Allelic	1.20(1.12-1.29)	9.14×10^{-7}	0	0.717	AAA	Strong	5851	A	A	A			1.17(1.07-1.28)	0.001	None	No	0.223	0.401	0.462
ATM	rs189037	A/G	Asian	5	3036/3415	Additive	1.09(1.00-1.18)	0.050	29	0.227	ABC	Moderate	5653	A	B	C	Low OR	Highly consistent OR	1.12(1.02-1.22)	0.016	None	Yes	0.195	1.000	0.806
CHRNA3	rs6495309	T/C	Asian	3	2635/2767	Allelic	0.83(0.76-0.91)	6.17×10^{-5}	27	0.254	ABA	Moderate	4924	A	B	A			0.83(0.76-0.91)	0.000	None	No	0.758	1.000	1.000
CLPTM1L	rs402710	T/C	Asian	8	5413/6143	Dominant	0.84(0.77-0.92)	1.53×10^{-4}	17	0.296	AAA	Strong	5150	A	A	A			0.82(0.74-0.91)	0.000	No	No	0.934	1.000	0.711
CYP1A1	rs4646903	C/T	Asian	15	2418/3107	Allelic	1.18(1.01-1.38)	0.036	73	0.000	ACC	Weak	4263	A	C	C	First study,small study		1.17(0.99-1.38)	0.073	No	No	0.037	0.156	0.113
CYP1A1	rs1048943	G/A	Asian	21	4265/5242	Allelic	1.17(1.02-1.33)	0.024	73	0.000	ACC	Weak	5007	A	C	C	Excess of significant studies		1.15(1.00-1.32)	0.048	No	No	0.213	0.025	0.740
CYP2A6	non*4/*4	del/-(*4/non*4)	Asian	6	2517/2264	Recessive	0.52(0.36-0.75)	0.001	0	0.454	BAA	Moderate	128	B	A	A			0.53(0.34-0.83)	0.006	No	No	0.961	0.636	0.707
CYP2E1	rs6413432	A/T	Asian	6	1964/2085	Allelic	0.78(0.70-0.86)	1.31×10^{-6}	0	0.824	AAA	Strong	1956	A	A	A			0.77(0.70-0.86)	0.000	No	No	0.440	0.680	0.707
FASLG	rs763110	T/C	Asian	3	1857/2149	Recessive	0.77(0.60-0.99)	0.039	16	0.303	BAC	Weak	351	B	A	C	First study		0.80(0.50-1.27)	0.344	None	No	0.959	0.543	1.000
GSTM1	Present/Null	NA	Asian	36	11012/9982	Allelic	1.27(1.14-1.41)	9.06×10^{-6}	65	0.000	ACC	Weak	11195	A	C	C	Excess of significant studies		1.26(1.13-1.41)	0.000	None	No	0.543	0.023	0.967
GSTP1	rs1695	G/A	Asian	11	2307/2430	Allelic	1.14(1.01-1.29)	0.036	27	0.187	ABC	Weak	1860	A	B	C	Low OR		1.16(1.01-1.35)	0.038	No	Yes	0.914	0.293	0.755
GSTT1	Present/Null	NA	Asian	14	7043/5289	Allelic	1.15(1.03-1.28)	0.010	34	0.105	ABA	Moderate	6305	A	B	A			1.15(1.03-1.29)	0.014	None	No	0.805	0.431	0.827
IL10	rs1800872	A/C	Asian	3	842/1257	Allelic	0.76(0.62-0.93)	0.009	56	0.106	ACC	Weak	2644	A	C	C	First study		0.81(0.64-1.02)	0.068	None	No	0.420	1.000	0.296
MAPKAPK2	CNV-30450	NA	Asian	3	2332/2480	4 copies vs 2 copies	1.60(1.04-2.45)	0.031	81	0.005	BCB	Weak	586	B	C	B	Missing information		NA		None	No	0.947	0.298	1.000
MDM2	rs2279744	G/T	Asian	8	5013/5834	Allelic	1.13(1.03-1.24)	0.011	64	0.006	ACC	Weak	11054	A	C	C	Low OR,small study		1.15(1.03-1.27)	0.010	None	Yes	0.097	0.724	0.458
MIR146A	rs2910164	C/G	Asian	4	2807/2841	Recessive	1.23(1.09-1.39)	0.001	0	0.594	AAA	Strong	1481	A	A	A			1.28(1.11-1.48)	0.001	None	No	0.861	0.622	1.000
MIR196A2	rs11614913	C/T	Asian	4	2376/2413	Dominant	1.22(1.07-1.38)	0.002	0	0.444	AAA	Strong	3448	A	A	A			1.32(1.11-1.56)	0.002	None	No	0.173	1.000	0.308
PROM1	rs2240688	C/A	Asian	3	2332/2457	Additive	0.83(0.76-0.91)	6.92×10^{-5}	0	0.991	AAB	Moderate	2459	A	A	B	Missing information		NA		None	No	0.379	0.281	0.296
REV3L	rs462779	T/C	Asian	4	1937/2335	Additive	1.11(1.02-1.22)	0.021	0	0.911	AAC	Strong	3433	A	A	C	Low OR	Highly consistent OR	1.13(1.00-1.27)	0.043	None	Yes	0.921	1.000	0.734
REV3L	rs465646	C/T	Asian	3	1296/1511	Allelic	0.83(0.71-0.97)	0.016	14	0.311	BAB	Moderate	982	B	A	B	Missing information		NA		None	No	0.288	1.000	1.000
TERT	rs2736098	A/G	Asian	5	3829/3992	Dominant	1.26(1.14-1.39)	1.03×10^{-5}	0	0.896	AAA	Strong	3696	A	A	A			1.27(1.13-1.43)	0.000	None	No	0.550	0.379	1.000
VEGFA	rs833061	C/T	Asian	3	967/930	Recessive	0.65(0.46-0.93)	0.018	0	0.800	BAC	Weak	146	B	A	C	First study		0.70(0.46-1.07)	0.104	None	No	0.709	1.000	1.000
WWOX	CNV-67048	NA	Asian	4	2942/3074	0 copy vs 2 copies	2.06(1.58-2.70)	1.20×10^{-7}	0	0.911	BAB	Moderate	247	B	A	B	Missing information		NA		None	No	0.648	1.000	1.000
XRCC1	rs25487	A/G	Asian	22	8845/9585	Recessive	1.25(1.00-1.56)	0.049	60	0.000	ACC	Weak	1128	A	C	C	First study,HWE, excess of significant studies		1.24(0.99-1.57)	0.063	Yes	No	0.795	0.046	0.721
XRCC1	rs3213245	C/T	Asian	4	2686/2755	Allelic	1.33(1.04-1.69)	0.021	74	0.010	ACC	Weak	1234	A	C	C	First study		1.27(0.91-1.78)	0.167	None	No	0.773	0.651	0.734

NA=Not Applicable; VNTR=variable number of tandem repeats.

[a] Minor alleles/major alleles (per Caucasian)

[b] Summary ORs were calculated based on the DerSimonian-Lairs random-effects model

[c] *P*-value of heterogeneity between studies

[d] Venice criteria grades contained assessments of three aspects: the amount of evidence, extent of replication, and protection from bias, and each assessment was assigned three levels (A, B, or C).

[e] Credibility of evidence was evaluated based on Venice criteria with three aspects (the amount of evidence, extent of replication, and protection from bias), and was categorized as strong (all three aspect grades were A), moderate (any grade was B, but not C) or weak (any grade was C).

[f] Amount of evidence, depending on total sample size of the smallest genetic group among cases and controls in each meta-analysis, was graded as A (sample size>1000), B (sample size between 100 and 1000), or C (sample size <100).

[g] Extent of replication, depending on between-study heterogeneity, was graded as A (I^2<25%), B (I^2 between 25% and 50%), or C (I^2>50%).

[h] Protection from bias, considering various potential sources of bias in meta-analysis, was graded as A when there was no demonstrable bias and bias was unlikely invalidate the association, was graded as B when there was insufficient information for identifying evidence (i.e. missing information for evaluating Hardy-Weinberg equilibrium (HWE) among controls in an individual study) although there was no obvious bias, and C when the bias was evident and/or was likely to explain the presence of association. For the C grade, it was assigned if a meta-analysis had any of the following potential sources of bias: (1) magnitude of the association was low; (2) sensitivity analysis altered the statistical significance; (3) potential small study effect; (4) possible existence of an excess of significant findings (the number of observed significant studies more than the number of expected significant studies); (5) potential publication bias.

First study=Significance lost excluding the first published or first positive report; Low OR=Low magnitude of the association (i.e. 0.87<OR<1.15); Small study=Possible existence of potential small study effect (modified Egger's test, *p*<0.10); Excess of significant studies=Possible existence of an excess of significant findings (excess significance test, *p*<0.10); Publication bias= Possible existence of potential publication bias (Begg's test, *p*<0.10); Missing information= Insufficient information for bias evaluation; HWE=Significance lost excluding studies with controls violating HWE; Highly consistent OR=A meta-analysis with a low OR was not considered if there is a highly consistent OR across studies enrolled in this meta-analysis

[i] A sensitivity analysis was performed to examine whether the significant summary ORs were robust after excluding the first published or first positive report (defined as the earliest study reported a significant association), or excluding studies with controls violating HWE. None=A meta-analysis didn't include studies with controls violating HWE or evaluated association for a variant with a phenotype change; NO=Without the influence from studies with controls violating HWE; NA=Insufficient information for evaluation of influence from first published or first reported positive study; Yes=Evidence of influence from studies with controls violating HWE.

[j] Low OR (i.e. 0.87<OR<1.15; No=Association without a low OR;Yes=Association with a low OR)

[k] *P* value for potential small study effect based on the modified Egger's test. (*P*<0.10 is usually considered evidence for significant evidence of small study effect.)

[l] *P* value for possible existing an excess of significant findings based on the excess significance test. (*P*<0.10 is usually considered evidence for significant evidence of an excess of significant findings.)

[m] *P* value for potential publication bias of studies based on the Begg's test. (*P*<0.10 is usually considered evidence for significant evidence of publication bias).

Supplementary Table S5. Subgroup meta-analyses stratified by different histological types of lung cancer for variants with sufficient data under different genetic models

(Continued on next two pages)

Genes	Sub-group	Variants	Comparison[a]	Results of meta-analysis using allelic model						Results of meta-analysis using dominant model						Results of meta-analysis using recessive model					
				Studies	Cases/Controls	OR(95% CI)	*P* value	I^2(%)	P_Q[b]	Studies	Cases/Controls	OR(95% CI)	*P* value	I^2(%)	P_Q[b]	Studies	Cases/Controls	OR(95% CI)	*P* value	I^2(%)	P_Q[b]
CHRNA5	SCLC	rs16969968(p.Asp398Asn,c.1192G>A)	A vs G	3	513/3253	1.55(1.15-2.10)	0.004	55	0.109												
CYP1A1	SCLC	rs4646903(m1,MspI,T3801C)	C vs T	14	325/2955	1.05(0.84-1.32)	0.650	0	0.879	17	494/3409	1.00(0.79-1.28)	0.979	0	0.77	12	273/2545	1.71(1.08-2.71)	0.021	0	0.90
CYP1A1	SCLC	rs1048943(*2C,m2,p.Ile462Val,c.1384A>G)	G vs A	11	402/2172	1.06(0.74-1.53)	0.752	46	0.050	11	422/2218	0.83(0.53-1.29)	0.403	40	0.08	7	285/1143	1.93(0.79-4.72)	0.149	47	0.08
CYP2D6	SCLC	phenotype(EM/HEM/PM)	PM vs EM	4	93/433	1.01(0.31-3.27)	0.986	0	0.438	5	117/509	0.80(0.50-1.28)	0.349	0	0.73	4	16/228	1.12(0.35-3.58)	0.849	0	0.44
CYP2E1	SCLC	rs6413432(c.967+1143T>A,7632T>A)	A vs T	6	254/1809	0.91(0.54-1.52)	0.714	56	0.044	6	254/1809	1.05(0.56-1.97)	0.886	61	0.03	5	250/1771	0.70(0.23-2.07)	0.514	24	0.26
CYP2E1	SCLC	rs2031920(RsaI,c1>c2,-1053C>T)	T vs C	6	390/3110	0.80(0.56-1.14)	0.215	57	0.041	6	390/3110	0.70(0.46-1.07)	0.102	56	0.05	5	346/2404	1.45(0.47-4.53)	0.521	69	0.01
ERCC2	SCLC	rs1799793(p.Asp312Asn,c.981G>A)	A vs G	3	303/1321	1.20(0.90-1.60)	0.221	0	0.571												
GSTM1	SCLC	Present/Null(Large gene deletion)	Null vs present	26	1224/7255	1.30(1.09-1.56)	0.004	43	0.010												
GSTP1	SCLC	rs1695(p.Ile105Val,c.313A>G)	G vs A	8	533/3555	1.12(0.97-1.29)	0.122	0	0.847	10	678/3932	1.02(0.86-1.22)	0.811	0	0.45	8	533/3555	1.34(1.00-1.79)	0.054	0	0.62
GSTT1	SCLC	Present/Null(Large gene deletion)	Null vs present	16	762/4200	1.07(0.86-1.32)	0.561	0	0.952												
MDM2	SCLC	rs2279744(SNP309,c.14+309T>G)	G vs T	3	265/2309	0.85(0.70-1.04)	0.117	13	0.318	3	265/2309	0.77(0.58-1.03)	0.078	0	0.92	3	265/2309	0.81(0.46-1.42)	0.453	59	0.09
MPO	SCLC	rs2333227(G-463A)	A vs G	3	392/2783	0.90(0.70-1.16)	0.420	30	0.242	3	392/2783	0.91(0.66-1.26)	0.570	40	0.19	3	392/2783	0.74(0.43-1.29)	0.289	0	0.81
NAT2	SCLC	Rapid/slow(Acetylation Phenotype)	Slow vs rapid	4	234/1993	1.26(0.89-1.77)	0.195	0	0.867												
NQO1	SCLC	rs1800566(p.Pro187Ser,c.648C>T,609C>T,*2)	T vs C	3	205/792	1.68(1.05-2.68)	0.029	56	0.102	4	264/1152	1.58(0.98-2.58)	0.063	59	0.06	3	205/792	2.32(0.93-5.78)	0.071	0	0.80
OGG1	SCLC	rs1052133(p.Ser326Cys,c.977C>G,8055C>G)	G vs C	4	376/2129	0.88(0.71-1.08)	0.225	26	0.254	3	165/1444	0.71(0.43-1.18)	0.192	37	0.20	4	220/2474	1.14(0.65-1.99)	0.66	53	0.09
PADPRP	SCLC	A/B (a 193-bp deletion)	Del vs non-del							3	30/339	0.81(0.34-1.95)	0.641	0	0.66						
TNF	SCLC	rs1800629(G-308A,*1/*2)	A vs G	3	107/512	0.87(0.55-1.38)	0.555	0	0.913	3	107/512	0.89(0.53-1.49)	0.659	0	0.75	3	107/512	0.83(0.18-3.84)	0.813	0	0.74
TP53	SCLC	rs1042522(p.Pro72Arg,c.215C>G)	C vs G	15	835/4556	0.97(0.72-1.29)	0.814	80	0.000	15	714/4453	0.93(0.64-1.35)	0.706	73	0.00	14	624/3871	1.18(0.72-1.91)	0.515	65	0.00
XPC	SCLC	PAT-/+ (24660_24664delGTAAC, inspoly(AT))	Ins vs non-ins	3	208/1340	0.86(0.58-1.27)	0.443	69	0.040	3	208/1340	0.72(0.48-1.09)	0.122	46	0.16	3	208/1340	1.05(0.56-1.95)	0.882	52	0.13
XPC	SCLC	rs2228001(p.Gln939Lys,Ex16+211A＞C)	C vs A	3	347/2108	0.84(0.71-1.00)	0.046	0	0.457												
XRCC1	SCLC	rs1799782(p.Arg194Trp,c.580C>T,Ex6-22C＞T)	T vs C	3	289/1429	0.91(0.73-1.12)	0.364	0	0.519												
XRCC1	SCLC	rs25487(p.Gln399Arg,c.1196A>G)	A vs G	5	470/2097	0.61(0.33-1.12)	0.109	91	0.000	4	259/1412	0.43(0.12-1.56)	0.197	94	0.00	4	259/1412	0.60(0.29-1.24)	0.167	42	0.16
ABCB1	NSCLC	rs1045642(p.Ile1145Ile,c.3435T>C)	T vs C	3	312/331	1.48(0.57-3.82)	0.421	93	0.000	3	312/331	1.29(0.43-3.86)	0.651	89	0.00	3	312/331	1.66(0.54-5.12)	0.38	85.60	0.00
ACE	NSCLC	287-bp repeat (I/D)	Ins vs del	3	450/603	0.97(0.72-1.32)	0.859	59	0.086	3	450/603	0.97(0.74-1.27)	0.823	2	0.36	3	450/603	0.86(0.43-1.72)	0.66	71.00	0.03
APEX1	NSCLC	rs1760944(c.-468T>G,-141T>G)	A vs C	3	330/1017	1.07(0.81-1.41)	0.636	53	0.120	3	330/1017	1.11(0.61-2.03)	0.738	73	0.02	3	330/1017	1.16(0.84-1.60)	0.37	0.00	0.38
APEX1	NSCLC	rs1130409(p.Asp148Glu,c.444T>G)	G vs T	9	2902/3665	0.92(0.82-1.03)	0.151	57	0.018	9	1999/3100	0.94(0.77-1.14)	0.506	58	0.01	8	1900/2980	0.85(0.69-1.05)	0.14	44.50	0.08
BIRC5	NSCLC	rs9904341(c.-31G>C）	C vs G	4	857/916	1.15(0.71-1.85)	0.576	89	0.000	4	857/916	1.04(0.58-1.86)	0.887	83	0.00	4	857/916	1.60(0.88-2.93)	0.13	76.10	0.01
CCND1	NSCLC	rs9344(rs603965,p.Pro241Pro,c.723G>A,c.870G>A)	A vs G	3	724/1264	1.00(0.76-1.32)	0.991	73	0.026	3	724/1264	0.94(0.59-1.52)	0.815	76	0.02	3	724/1264	1.04(0.69-1.56)	0.86	64.60	0.06
CDKN1A	NSCLC	rs1801270(p.Ser31Arg,c.93C>A,6829C>A)	A vs C	5	1163/2043	1.15(0.86-1.54)	0.344	50	0.090	5	1163/2043	1.26(0.93-1.72)	0.140	42	0.14	4	1054/1948	0.71(0.44-1.15)	0.17	0.00	0.97
CHRNA5	NSCLC	rs16969968(p.Asp398Asn,c.1192G>A)	A vs G	6	3201/4736	1.36(1.24-1.48)	1.48×10^{-11}	13	0.329	4	2225/3577	1.50(1.28-1.75)	4.03×10^{-7}	18	0.30	4	2225/3577	1.49(1.24-1.79)	1.71×10^{-5}	0.00	0.80
CLPTM1L	NSCLC	rs402710(C>T)	T vs C	6	2940/4040	0.85(0.79-0.91)	1.13×10^{-5}	0	0.666	5	2257/3195	0.84(0.72-0.97)	0.018	30	0.22	5	2257/3195	0.78(0.62-0.99)	0.04	30.70	0.22
CTLA4	NSCLC	rs5742909(g.319C>T,-318C>T)	T vs C	3	361/549	0.50(0.11-2.31)	0.376	95	0.000	3	361/549	0.48(0.10-2.21)	0.343	94	0.00	3	361/549	0.58(0.04-8.50)	0.69	77.90	0.01
CTLA4	NSCLC	rs231775(p.Thr17Ala,c.49A>G)	G vs A	4	590/800	0.92(0.45-1.87)	0.814	94	0.000	4	590/800	1.01(0.44-2.33)	0.985	92	0.00	4	590/800	0.92(0.36-2.37)	0.86	87.60	0.00
CXCL12	NSCLC	rs1801157(801G>A)	A vs G	3	467/815	1.33(0.75-2.37)	0.325	89	0.000	3	467/815	1.47(0.74-2.94)	0.275	86	0.00	3	467/815	1.43(0.56-3.65)	0.46	81.60	0.00
CYP1A1	NSCLC	rs4646903(m1,MspI,T3801C)	C vs T	24	3032/5165	1.11(1.03-1.21)	0.009	0	0.458	34	4465/6895	1.17(1.06-1.30)	0.003	28	0.07	22	2789/4778	1.18(0.95-1.46)	0.14	16.60	0.24
CYP1A1	NSCLC	rs1048943(*2C,m2,p.Ile462Val,c.1384A>G)	G vs A	22	4672/6125	1.10(0.92-1.32)	0.284	79	0.000	26	5123/6735	1.11(0.90-1.36)	0.322	78	0.00	19	4264/5386	1.24(0.88-1.76)	0.23	55.10	0.00
CYP1A2	NSCLC	rs2069526(-739T>G)	G vs T							3	495/537	1.73(0.79-3.80)	0.173	69	0.04						
CYP1A2	NSCLC	rs35694136(-2467delT)	Del vs non-del							3	390/494	1.30(0.62-2.73)	0.481	76	0.02						
CYP1A2	NSCLC	rs2069514(CYP1A2*1C,-3860G>A)	A vs G							3	440/425	1.39(0.30-6.44)	0.677	92	0.00						
CYP1A2	NSCLC	rs762551(CYP1A2*1F,c.A-164C)	C vs A	3	636/691	0.88(0.58-1.34)	0.550	84	0.002	4	729/802	0.78(0.48-1.29)	0.334	79	0.00	4	718/887	0.99(0.64-1.53)	0.95	55.90	0.08
CYP1B1	NSCLC	rs1056836(CYP1B1*3,p.Leu432Val,c.1294C>G)	G vs C	3	640/737	1.14(0.95-1.37)	0.164	0	0.695	3	640/737	1.20(0.95-1.52)	0.131	0	0.64	3	640/737	1.10(0.74-1.62)	0.65	0.00	0.92
CYP2A6	NSCLC	non*4/*4(*1/*4, a deletion of the CYP2A6 gene)	Del vs non-del							4	979/1432	1.07(0.67-1.69)	0.784	75	0.01						
CYP2C9	NSCLC	rs1799853(p.Arg144Cys,c.430C>T)	T vs C							3	518/1096	1.06(0.54-2.07)	0.871	73	0.03						
CYP2D6	NSCLC	rs1065852(p.Pro34Ser,188C>T)	C vs T							3	571/636	0.83(0.58-1.18)	0.299	45	0.16						
CYP2D6	NSCLC	phenotype(EM/HEM/PM)	PM vs EM	6	611/1424	0.84(0.38-1.88)	0.676	50	0.076	7	682/1500	0.98(0.80-1.20)	0.825	0	0.52	7	661/1474	0.76(0.35-1.65)	0.48	55.60	0.04
CYP2E1	NSCLC	rs3813867(g.3739G>C,c.-1293G>C)	C vs G							4	900/971	0.97(0.58-1.64)	0.921	59	0.06						
CYP2E1	NSCLC	rs6413432(c.967+1143T>A,7632T>A)	A vs T	6	1290/1809	0.80(0.71-0.91)	4.90×10^{-4}	0	0.868	7	1372/1890	0.84(0.72-0.98)	0.022	0	0.44	5	1240/1771	0.51(0.25-1.04)	0.06	40.30	0.15
CYP2E1	NSCLC	rs2031920(RsaI,c1>c2,-1053C>T)	T vs C	9	2512/4101	0.92(0.78-1.08)	0.301	61	0.009	10	2648/4280	0.90(0.77-1.04)	0.151	34	0.14	8	2309/3395	1.04(0.56-1.94)	0.89	71.30	0.00
CYP3A4	NSCLC	rs2740574(–392A>G,*1A/*1B)	G vs A	3	923/845	0.94(0.64-1.39)	0.756	37	0.203	3	923/845	1.03(0.74-1.44)	0.868	0	0.55						
EPHX1	NSCLC	rs1051740(p.Tyr113His,c.337T>C)	C vs T	5	742/1343	1.48(0.94-2.33)	0.094	89	0.000	5	742/1343	1.74(0.92-3.29)	0.087	89	0.00	5	742/1343	1.67(0.91-3.08)	0.10	73.10	0.01
EPHX1	NSCLC	rs2234922(p.His139Arg,c.416A>G)	G vs A	4	1710/2512	1.18(0.77-1.79)	0.454	86	0.000	4	1710/2512	1.28(0.76-2.14)	0.354	86	0.00	4	1710/2512	0.99(0.49-1.98)	0.98	65.60	0.03
ERCC1	NSCLC	rs11615(rs3177700,p.Asn118Asn,c.354T>C)	C vs T	3	780/811	0.68(0.58-0.81)	1.01×10^{-5}	13	0.316	3	780/811	0.49(0.36-0.68)	1.73×10^{-5}	0	0.63	3	780/811	0.69(0.56-0.85)	0.00	0.00	0.50
ERCC2	NSCLC	rs1799793(p.Asp312Asn,c.981G>A)	A vs G	8	2884/2833	1.03(0.80-1.33)	0.832	81	0.000	7	1882/2148	1.11(0.91-1.36)	0.294	44	0.10	6	2643/3168	1.52(0.95-2.45)	0.08	63.30	0.01
ERCC2	NSCLC	rs13181(p.Lys751Gln,c.2251A>C)	C vs A	8	2770/2856	1.19(1.04-1.36)	0.014	39	0.116	6	1672/2075	1.32(1.14-1.53)	1.80×10^{-4}	0	0.85	7	2438/3112	1.37(1.12-1.68)	0.00	0.00	0.74
ERCC5	NSCLC	rs17655(p.Asp1104His,c.3310G>C)	C vs G							3	1641/1204	0.95(0.54-1.67)	0.851	87	0.00						
FAS	NSCLC	rs2234767(-1377G/A)	A vs G	3	2482/1844	1.12(0.88-1.42)	0.354	58	0.092	3	2482/1844	1.12(0.84-1.49)	0.460	50	0.14	3	3194/3064	1.51(1.23-1.87)	1.26×10^{-4}	0.00	0.45
FASLG	NSCLC	rs763110(-844C/T)	T vs C							3	3167/3057	0.74(0.47-1.17)	0.202	94	0.00						
FGFR4	NSCLC	rs351855(p.Gly388Arg,G>A)	A vs G	3	985/1230	0.76(0.68-0.86)	1.97×10^{-5}	0	0.590	3	985/1230	0.70(0.58-0.84)	1.14×10^{-4}	0	0.62	3	985/1230	0.75(0.51-1.10)	0.14	36.60	0.21
GSTM1	NSCLC	Present/Null (Large gene deletion)	Null vs present	60	11551/15673	1.18(1.08-1.29)	1.56×10^{-4}	62	0.000												
GSTM3	NSCLC	rs1799735(c.468+21delAGG,3bp deletion,*A/*B)	Del vs non-del	4	760/1174	1.00(0.84-1.20)	0.976	0	0.878	4	760/1174	0.89(0.72-1.10)	0.278	0	0.46	4	760/1174	1.88(0.94-3.76)	0.07	37.70	0.19
GSTP1	NSCLC	rs1695(p.Ile105Val,c.313A>G)	G vs A	21	5642/7022	1.07(0.99-1.16)	0.086	38	0.043	27	6767/8503	1.05(0.96-1.14)	0.281	32	0.06	21	5642/7022	1.16(1.00-1.35)	0.05	17.40	0.23
GSTT1	NSCLC	Present/Null(Large gene deletion)	Null vs present	31	6368/9045	1.00(0.89-1.13)	0.979	45	0.004												
HYKK	NSCLC	rs931794(A>G)	G vs A	4	1548/2464	1.25(1.13-1.37)	9.08×10^{-6}	0	0.880	4	1548/2464	1.30(1.14-1.48)	1.09×10^{-4}	0	0.90	4	1548/2464	1.37(1.13-1.65)	0.00	0.00	0.49
IL10	NSCLC	rs1800871(c.-854T>C,-819C>T)	T vs C	4	753/1428	0.95(0.71-1.28)	0.753	68	0.024	4	753/1428	0.87(0.54-1.41)	0.576	75	0.01	4	753/1428	0.99(0.63-1.56)	0.98	51.60	0.10
IL10	NSCLC	rs1800896(g.3943A>G,-1082A>G)	G vs A	3	306/523	1.74(0.75-4.04)	0.202	89	0.000	3	306/523	1.61(0.46-5.64)	0.459	91	0.00						
IL17A	NSCLC	rs2275913(-73G>A,-152G>A)	A vs G	3	780/998	1.37(0.95-1.98)	0.093	81	0.005	3	780/998	1.36(0.84-2.20)	0.206	82	0.00	3	780/998	1.72(1.12-2.65)	0.01	31.00	0.24
IL1B	NSCLC	rs1143627(g.4970C>T,-31T>C)	C vs T	5	1915/2551	0.82(0.67-0.99)	0.043	78	0.001	5	1915/2551	0.79(0.58-1.08)	0.146	78	0.00	5	1915/2551	0.70(0.52-0.92)	0.01	65.50	0.02
IL1B	NSCLC	rs16944(C-511T)	T vs C	6	2147/2798	0.89(0.74-1.09)	0.257	80	0.000	6	2147/2798	0.92(0.69-1.21)	0.542	77	0.00	6	2147/2798	0.78(0.57-1.07)	0.12	74.40	0.00
IL4	NSCLC	rs2243250(-509C>T)	T vs C	3	1892/2295	1.10(0.84-1.44)	0.477	82	0.004	3	1892/2295	1.16(0.75-1.80)	0.502	73	0.03	3	1892/2295	1.04(0.72-1.50)	0.86	78.80	0.01
IL6	NSCLC	rs1800795(G-174C)	C vs G	4	567/725	1.35(0.87-2.08)	0.184	81	0.001	4	567/725	1.20(0.68-2.12)	0.523	79	0.00	4	567/725	1.82(0.98-3.37)	0.06	59.40	0.06
IL6	NSCLC	rs1800796(-572C>G,634C>G)	G vs C	3	1333/1452	0.75(0.57-0.99)	0.039	77	0.013	4	1416/1614	0.82(0.65-1.04)	0.095	54	0.09	3	1333/1452	0.67(0.36-1.24)	0.20	62.80	0.07
IL8	NSCLC	rs4073(T-251A)	T vs A	4	1997/2751	1.07(0.83-1.39)	0.593	81	0.001	4	1997/2751	1.04(0.78-1.38)	0.789	57	0.07	4	1997/2751	1.11(0.78-1.57)	0.56	75.80	0.01
LTA	NSCLC	rs909253(A+252G)	G vs A	4	663/1297	0.92(0.80-1.06)	0.235	0	0.573	4	663/1297	0.95(0.74-1.22)	0.702	28	0.24	4	663/1297	0.78(0.57-1.05)	0.10	0.00	0.86
MDM2	NSCLC	rs2279744(SNP309,c.14+309T>G)	G vs T	10	4170/5502	1.08(0.88-1.33)	0.472	91	0.000	10	4170/5502	1.10(0.80-1.51)	0.559	90	0.00	10	4170/5502	1.19(0.93-1.51)	0.16	77.40	0.00
MIR146A	NSCLC	rs2910164(C>G)	C vs G	4	880/1094	1.28(1.11-1.46)	4.63×10^{-4}	0	0.391	4	880/1094	1.41(1.13-1.74)	0.002	0	0.45	5	1832/2190	1.28(1.11-1.48)	0.00	0.00	0.72

Genes	Sub-group	Variants	Comparison[a]	Results of meta-analysis using allelic model						Results of meta-analysis using dominant model						Results of meta-analysis using recessive model					
				Studies	Cases/Controls	OR(95% CI)	*P* value	I^2(%)	P_Q[b]	Studies	Cases/Controls	OR(95% CI)	*P* value	I^2(%)	P_Q[b]	Studies	Cases/Controls	OR(95% CI)	*P* value	I^2(%)	P_Q[b]
MIR196A2	NSCLC	rs11614913(T>C)	C vs T	5	1320/1762	1.02(0.85-1.22)	0.869	62	0.032	5	1320/1762	1.18(0.88-1.59)	0.274	54	0.07	5	1320/1762	0.91(0.69-1.19)	0.47	55.10	0.06
MMP1	NSCLC	rs1799750(-16071G/2G,insG)	2G vs 1G	3	485/688	0.97(0.75-1.26)	0.832	50	0.134	4	569/770	1.10(0.74-1.63)	0.642	45	0.14	3	485/688	0.94(0.63-1.41)	0.77	50.90	0.13
MMP2	NSCLC	rs243865(-1306C>T)	T vs C	3	1216/1729	0.61(0.40-0.92)	0.019	83	0.003	3	1216/1729	0.57(0.36-0.92)	0.021	83	0.00	3	1216/1729	0.54(0.30-0.99)	0.05	0.00	0.72
MPO	NSCLC	rs2333227(G-463A)	A vs G	10	5036/6646	1.06(0.95-1.18)	0.294	54	0.020	11	5124/6727	1.03(0.93-1.14)	0.633	32	0.14	10	5036/6646	1.38(1.03-1.86)	0.03	51.20	0.03
MTHFR	NSCLC	rs1801131(p.Ala429Glu,c.1286A>C,A1298C)	C vs A	4	785/1111	1.09(0.94-1.27)	0.240	0	0.654	4	785/1111	1.10(0.90-1.33)	0.363	0	0.69	4	785/1111	1.16(0.85-1.57)	0.35	0.00	0.59
MTHFR	NSCLC	rs1801133(p.Ala222Val,Ex4+79C＞T)	T vs C	6	1273/1813	1.09(0.83-1.42)	0.543	78	0.000	6	1273/1813	1.07(0.72-1.59)	0.740	78	0.00	6	1273/1813	1.23(0.84-1.79)	0.29	57.70	0.04
NAT2	NSCLC	Rapid/slow	Slow vs rapid	11	2041/3282	0.94(0.75-1.17)	0.572	58	0.008												
NQO1	NSCLC	rs1800566(p.Pro187Ser,c.648C>T,609C>T,*2)	T vs C	6	1511/2708	1.10(0.87-1.38)	0.442	74	0.002	8	1914/3400	1.08(0.91-1.28)	0.402	41	0.10	6	1511/2708	1.13(0.72-1.77)	0.60	60.60	0.03
OGG1	NSCLC	rs1052133(p.Ser326Cys,c.977C>G,8055C>G)	G vs C	17	6874/7869	1.07(0.98-1.17)	0.141	60	0.001	17	5948/7259	1.08(0.95-1.24)	0.248	54	0.01	17	6279/8214	1.16(1.02-1.32)	0.02	35.70	0.07
PADPRP	NSCLC	A/B(a 193-bp deletion)	Del vs non-del							4	258/339	1.22(0.78-1.90)	0.382	35	0.20						
PARP1	NSCLC	rs1136410(p.Val762Ala,Ex17+8T＞C)	C vs T	3	2157/2095	1.08(0.89-1.32)	0.416	79	0.009												
PTGS2	NSCLC	rs5275(c.*427T>C,8473T>C)	C vs T	4	1143/1319	1.04(0.61-1.76)	0.889	93	0.000	4	1143/1319	1.07(0.62-1.87)	0.800	89	0.00	4	1143/1319	1.02(0.40-2.62)	0.97	83.40	0.00
SOD2	NSCLC	rs4880(rs1799725,Ala16Val)	T vs C	6	3133/4255	1.25(1.08-1.45)	0.002	72	0.003	6	3133/4255	1.32(1.12-1.55)	0.001	38	0.15	7	3321/4587	1.24(1.00-1.53)	0.05	69.70	0.00
SULT1A1	NSCLC	rs9282861(p.Arg213His,c.638G>A,*1/*2)	A vs G							3	977/1476	1.44(0.87-2.41)	0.159	84	0.00						
TERT	NSCLC	rs2736098(p.Ala305Ala,c.915G>A)	A vs G	4	2002/2490	1.30(1.19-1.42)	2.59×10^{-9}	0	0.818	4	2002/2490	1.27(1.12-1.43)	1.18×10^{-4}	0	0.90	4	2002/2490	1.69(1.43-1.99)	7.97×10^{-10}	0.00	0.78
TNF	NSCLC	rs1800629(G-308A,*1/*2)	A vs G	6	1052/1767	1.67(1.01-2.76)	0.044	89	0.000	6	1052/1767	1.75(0.99-3.09)	0.055	89	0.00	6	1052/1767	2.29(1.21-4.33)	0.01	22.80	0.26
TNF	NSCLC	rs361525(A-417G,-238G>A)	A vs G	4	641/779	1.13(0.48-2.68)	0.778	91	0.000	4	641/779	1.04(0.43-2.49)	0.933	89	0.00						
TP53	NSCLC	rs1625895(IVS6+62G>A,A2>A1)	A vs G	3	263/495	0.94(0.64-1.38)	0.754	42	0.178	3	263/495	0.87(0.48-1.55)	0.629	64	0.06	3	263/495	1.19(0.09-16.66)	0.90	72.50	0.03
TP53	NSCLC	rs1042522(p.Pro72Arg,c.215C>G)	C vs G	28	8459/11237	0.98(0.88-1.10)	0.780	83	0.000	30	8908/11898	1.00(0.88-1.13)	0.986	75	0.00	28	8459/11237	1.02(0.83-1.24)	0.87	78.50	0.00
TP63	NSCLC	rs10937405(C>T)	T vs C	3	3587/8484	0.87(0.82-0.92)	9.91×10^{-7}	0	0.595	3	3587/8484	0.84(0.78-0.92)	6.93×10^{-5}	0	0.51	3	3587/8484	0.80(0.72-0.89)	6.49×10^{-5}	0.00	0.62
TP73	NSCLC	G4C14/A4T14	AT vs GC	4	1012/1210	0.90(0.70-1.15)	0.382	66	0.032	5	1946/2349	1.04(0.85-1.27)	0.735	56	0.06	4	1012/1210	0.75(0.35-1.59)	0.45	77.20	0.00
TYMS	NSCLC	28-bp tandem repeat	2R vs 3R (2R vs Non-2R)	3	808/1326	0.89(0.75-1.04)	0.142	0	0.391	3	808/1326	0.84(0.69-1.01)	0.065	0	0.52	3	808/1326	1.07(0.67-1.72)	0.78	0.00	0.53
VEGFA	NSCLC	rs2010963(634G>C)	C vs G	4	2728/2372	1.10(0.93-1.29)	0.264	62	0.047	4	2728/2372	1.12(0.92-1.37)	0.267	49	0.12	5	2828/2522	1.11(0.87-1.42)	0.42	45.40	0.12
VEGFA	NSCLC	rs833061(-460T>C)	C vs T	4	2727/2372	1.00(0.92-1.08)	0.987	0	0.847	4	2727/2372	1.04(0.92-1.17)	0.519	0	0.62	4	2727/2372	0.94(0.81-1.09)	0.42	0.00	0.54
VEGFA	NSCLC	rs3025039(C936T)	T vs C	3	2403/2034	1.04(0.92-1.17)	0.556	0	0.591	5	2640/2356	1.04(0.91-1.18)	0.563	0	0.45	3	2403/2034	1.12(0.73-1.71)	0.60	0.00	0.47
XPA	NSCLC	rs1800975(c.-4A>G,23G>A)	A vs G	4	1637/1793	1.01(0.86-1.19)	0.902	63	0.042	5	1851/1978	0.99(0.79-1.23)	0.910	60	0.04	5	1851/1978	1.11(0.87-1.41)	0.40	41.30	0.15
XPC	NSCLC	PAT-/+ (24660_24664delGTAAC, inspoly(AT))	Ins vs non-ins	3	967/1340	1.17(0.98-1.39)	0.092	55	0.110	3	967/1340	1.10(0.86-1.42)	0.455	51	0.13	3	967/1340	1.46(1.17-1.81)	0.00	0.00	0.48
XPC	NSCLC	rs2228000(p.Ala499Val,Ex9-377C>T)	T vs C	3	1492/1630	1.04(0.93-1.16)	0.514	0	0.567	3	1492/1630	1.03(0.89-1.19)	0.678	0	0.72	3	1492/1630	1.10(0.86-1.39)	0.45	0.00	0.40
XPC	NSCLC	rs2228001(p.Gln939Lys,Ex16+211A＞C)	C vs A	4	2491/2316	1.05(0.96-1.14)	0.303	0	0.744	3	1489/1631	1.05(0.91-1.22)	0.481	0	0.39	3	1489/1631	1.18(0.96-1.45)	0.12	0.00	0.69
XRCC1	NSCLC	rs2256507(G>A)	A vs G	3	431/779	1.23(0.93-1.62)	0.143	50	0.134	3	431/779	1.20(0.89-1.63)	0.236	34	0.22	3	431/779	1.68(1.03-2.74)	0.04	7.80	0.34
XRCC1	NSCLC	rs25486(A>G)	G vs A	3	373/795	1.06(0.69-1.61)	0.795	77	0.013	3	373/795	1.19(0.84-1.69)	0.334	21	0.28	3	373/795	1.13(0.49-2.58)	0.78	77.60	0.01
XRCC1	NSCLC	rs1001581(c.145–216G>A)	T vs C	4	574/994	1.27(1.04-1.56)	0.018	40	0.172	4	574/994	1.35(0.98-1.88)	0.069	55	0.08	4	574/994	1.36(1.02-1.83)	0.04	0.00	0.71
XRCC1	NSCLC	rs3547(p.Gln632Gln,c.1896G>A)	A vs G	3	374/794	1.06(0.85-1.32)	0.592	0	0.850	3	374/794	1.02(0.78-1.34)	0.868	0	0.99						
XRCC1	NSCLC	rs25489(p.Arg280His,c.839G>A,Ex9+16G>A)	A vs G	8	3183/3560	0.93(0.70-1.23)	0.607	74	0.000	7	3441/4871	0.94(0.69-1.28)	0.683	78	0.00	6	2085/2779	0.87(0.45-1.71)	0.69	0.00	0.47
XRCC1	NSCLC	rs1799782(p.Arg194Trp,c.580C>T,Ex6-22C＞T)	T vs C	11	3418/4189	0.93(0.81-1.06)	0.285	40	0.081	10	3717/5540	0.92(0.81-1.05)	0.223	19	0.27	9	2320/3408	1.15(0.90-1.47)	0.25	0.00	0.48
XRCC1	NSCLC	rs25487(p.Gln399Arg,c.1196A>G)	A vs G	19	7715/10261	1.04(0.96-1.14)	0.330	62	0.000	18	6716/9600	1.06(0.95-1.18)	0.284	52	0.01	17	6617/9480	1.08(0.92-1.27)	0.35	47.60	0.02
XRCC1	NSCLC	rs3213245(-77T>C)	C vs T							3	1744/2178	1.50(1.29-1.75)	1.89×10^{-7}	0	0.68						
XRCC3	NSCLC	rs861539(p.Thr241Met,c.722C>T)	T vs C	5	2287/2150	1.02(0.91-1.15)	0.760	0	0.531	4	1288/1489	0.90(0.75-1.08)	0.266	0	0.43	3	1189/1369	1.12(0.75-1.68)	0.57	19.90	0.29
APEX1	AD	rs1130409(p.Asp148Glu,c.444T>G)	G vs T	5	1561/2309	0.96(0.81-1.14)	0.637	64	0.026	5	877/1744	0.97(0.69-1.36)	0.850	70	0.01	4	809/1624	0.96(0.66-1.39)	0.813	58	0.07
BIRC5	AD	rs9904341(c.-31G>C）	C vs G	3	312/818	1.70(1.22-2.37)	0.002	57	0.097	3	312/818	1.86(1.09-3.18)	0.024	50	0.14	3	312/818	2.07(1.38-3.10)	4.41×10^{-4}	32	0.23
CDKN1A	AD	rs1801270(p.Ser31Arg,c.93C>A,6829C>A)	A vs C	5	523/2043	1.18(0.86-1.62)	0.306	36	0.180	5	523/2043	1.32(0.99-1.77)	0.063	7	0.37	4	465/1948	0.66(0.35-1.23)	0.190	0	0.80
CHRNA5	AD	rs16969968(p.Asp398Asn,c.1192G>A)	A vs G	4	1507/2834	1.37(1.14-1.64)	0.001	33	0.214												
CXCL12	AD	rs1801157(801G>A)	A vs G	3	251/815	1.16(0.68-1.99)	0.579	74	0.021	3	251/815	1.29(0.76-2.19)	0.339	55	0.11	3	251/815	1.16(0.37-3.60)	0.802	71	0.03
CYP1A1	AD	rs4646903(m1,MspI,T3801C)	C vs T	17	1001/3991	0.95(0.83-1.08)	0.417	0	0.543	23	1496/4974	0.98(0.85-1.12)	0.735	0	0.86	16	1012/3754	0.95(0.72-1.25)	0.691	0	0.87
CYP1A1	AD	rs1048943(*2C,m2,p.Ile462Val,c.1384A>G)	G vs A	15	1225/4175	1.01(0.80-1.27)	0.926	63	0.001	18	1813/5085	1.06(0.81-1.39)	0.672	68	0.00	11	1043/3146	1.26(0.75-2.11)	0.385	45	0.05
CYP2A6	AD	non*4/*4(*1/*4,a deletion of the CYP2A6 gene)	Del vs non-del (*4 vs non*4)							3	454/1300	1.07(0.80-1.45)	0.639	0	0.57						
CYP2D6	AD	phenotype(EM/HEM/PM)	PM vs EM	5	234/1317	1.42(0.75-2.69)	0.288	0	0.463	5	234/1317	0.93(0.69-1.25)	0.624	0	0.95	6	261/1367	1.20(0.59-2.44)	0.613	23	0.26
CYP2E1	AD	rs6413432(c.967+1143T>A,7632T>A)	A vs T	6	500/1809	0.79(0.66-0.95)	0.011	0	0.664	6	500/1809	0.85(0.63-1.16)	0.304	26	0.24	5	481/1771	0.51(0.20-1.32)	0.166	37	0.17
CYP2E1	AD	rs2031920(RsaI,c1>c2,-1053C>T)	T vs C	8	1250/3903	0.83(0.67-1.04)	0.109	62	0.011	8	1250/3903	0.82(0.65-1.03)	0.091	51	0.05	7	1129/3197	0.78(0.34-1.82)	0.572	63	0.01
EPHX1	AD	rs1051740(p.Tyr113His,c.337T>C)	C vs T							3	209/902	1.00(0.53-1.91)	0.990	66	0.05						
ERCC2	AD	rs1799793(p.Asp312Asn,c.981G>A)	A vs G	6	1568/2449	1.12(0.68-1.85)	0.659	90	0.000	5	816/1764	1.40(0.98-2.02)	0.067	62	0.03	5	1013/2401	1.89(0.65-5.50)	0.243	79	0.00
ERCC2	AD	rs13181(p.Lys751Gln,c.2251A>C)	C vs A	5	1416/1915	1.14(0.89-1.46)	0.294	46	0.118	4	664/1230	1.35(1.06-1.70)	0.013	0	0.64	5	960/2267	1.26(0.86-1.85)	0.242	0	0.61
GSTM1	AD	Present/Null (Large gene deletion)	Null vs present	42	4199/12206	1.17(1.05-1.31)	0.005	45	0.001												
GSTP1	AD	rs1695 (p.Ile105Val, c.313A>G)	G vs A	13	2509/5211	0.96(0.89-1.04)	0.362	2	0.430	18	3060/6381	0.93(0.85-1.02)	0.104	0	0.66	13	2509/5211	1.05(0.85-1.31)	0.642	18	0.26
GSTT1	AD	Present/Null (Large gene deletion)	Null vs present	24	2906/7999	1.08(0.91-1.28)	0.404	51	0.002												
IL10	AD	rs1800871(c.-854T>C,-819C>T)	T vs C	3	317/1172	0.84(0.48-1.48)	0.550	58	0.091	3	317/1172	1.00(0.77-1.28)	0.968	0	0.38	3	317/1172	0.83(0.19-3.56)	0.801	41	0.18
IL17A	AD	rs2275913(-73G>A,-152G>A)	A vs G	3	469/998	1.43(0.85-2.41)	0.184	88	0.000	3	469/998	1.40(0.70-2.82)	0.340	89	0.00	3	469/998	1.84(1.11-3.06)	0.018	36	0.21
IL6	AD	rs1800795(G-174C)	C vs G	3	128/475	0.97(0.72-1.29)	0.810	0	0.701	3	128/475	0.79(0.51-1.22)	0.291	0	0.89	3	128/475	1.21(0.58-2.52)	0.613	29	0.25
IL6	AD	rs1800796(-572C>G,634C>G)	G vs C							3	678/1414	0.83(0.68-1.00)	0.055	6	0.35						
LTA	AD	rs909253(A+252G)	G vs A	4	360/1247	0.92(0.73-1.16)	0.468	21	0.285	4	360/1247	0.93(0.67-1.29)	0.644	26	0.25	4	360/1247	0.80(0.55-1.18)	0.263	0	0.62
MDM2	AD	rs2279744(SNP309,c.14+309T>G)	G vs T	6	1714/4083	1.20(1.00-1.44)	0.052	76	0.001	6	1714/4083	1.28(0.95-1.72)	0.111	76	0.00	6	1714/4083	1.28(1.04-1.56)	0.018	46	0.10
MIR146A	AD	rs2910164(C>G)	C vs G	3	442/965	1.26(1.04-1.51)	0.018	15	0.309	3	442/965	1.42(1.06-1.90)	0.018	4	0.35	4	907/2061	1.33(1.12-1.59)	0.001	0	0.94
MIR196A2	AD	rs11614913(T>C)	C vs T	3	516/1205	1.00(0.79-1.26)	0.999	56	0.104	3	516/1205	1.06(0.66-1.72)	0.804	63	0.07	3	516/1205	0.92(0.67-1.26)	0.591	38	0.20
MMP2	AD	rs243865(-1306C>T)	T vs C	3	476/1729	0.69(0.41-1.16)	0.161	79	0.008	3	476/1729	0.67(0.36-1.27)	0.223	83	0.00	3	476/1729	0.62(0.28-1.40)	0.247	0	1.00
MPO	AD	rs2333227(G-463A)	A vs G	7	2191/5068	1.04(0.96-1.14)	0.337	0	0.514	7	2191/5068	1.02(0.92-1.13)	0.746	0	0.74	7	2191/5068	1.25(0.99-1.56)	0.059	0	0.56
MTHFR	AD	rs1801133(p.Ala222Val,Ex4+79C＞T)	T vs C	3	629/1326	0.84(0.56-1.27)	0.405	85	0.001	3	629/1326	0.79(0.35-1.79)	0.575	90	0.00	3	629/1326	0.94(0.74-1.19)	0.591	0	0.40
NAT2	AD	Rapid/slow (Acetylation Phenotype)	Slow vs rapid	9	851/2963	0.95(0.71-1.26)	0.704	51	0.038												
NQO1	AD	rs1800566(p.Pro187Ser,c.648C>T,609C>T,*2)	T vs C	3	544/1798	0.94(0.77-1.16)	0.571	11	0.325	5	707/2490	0.91(0.76-1.09)	0.287	0	0.77	3	544/1798	1.14(0.34-3.85)	0.836	58	0.09
OGG1	AD	rs1052133(p.Ser326Cys,c.977C>G,8055C>G)	G vs C	12	4039/6332	1.10(0.99-1.22)	0.077	53	0.015	11	3287/5647	1.12(0.93-1.34)	0.230	52	0.02	12	3603/6677	1.25(1.10-1.43)	0.001	20	0.25
PADPRP	AD	A/B (a 193-bp deletion)	Del vs non-del							4	129/339	1.65(0.81-3.35)	0.165	51	0.10						
PARP1	AD	rs1136410(p.Val762Ala,Ex17+8T＞C)	C vs T	3	1459/2095	1.09(0.89-1.33)	0.408	74	0.022												
SOD2	AD	rs4880(rs1799725,Ala16Val)	T vs C	4	1219/2761	1.20(1.08-1.32)	3.80×10^{-4}	0	0.405	4	1219/2761	1.23(1.04-1.45)	0.017	0	0.47	5	1324/3093	1.26(1.00-1.58)	0.052	44	0.13
TERT	AD	rs2736098(p.Ala305Ala,c.915G>A)	A vs G	4	1214/2490	1.40(1.26-1.54)	4.97×10^{-11}	0	0.891	4	1214/2490	1.37(1.19-1.58)	1.78×10^{-5}	0	0.96	4	1214/2490	1.89(1.57-2.28)	2.10×10^{-11}	0	0.78
TNF	AD	rs1800629(G-308A,*1/*2)	A vs G	5	484/1486	1.81(0.83-3.93)	0.133	90	0.000	5	484/1486	1.95(0.81-4.68)	0.135	90	0.00	4	458/1460	2.71(0.99-7.43)	0.054	39	0.18
TP53	AD	rs1625895(IVS6+62G>A,A2>A1)	A vs G	3	89/495	0.92(0.43-2.00)	0.840	63	0.068	3	89/495	0.90(0.31-2.58)	0.839	71	0.03	3	89/495	1.69(0.22-13.20)	0.619	41	0.18
TP53	AD	rs1042522(p.Pro72Arg,c.215C>G)	C vs G	21	3367/8584	1.08(0.98-1.20)	0.130	54	0.002	22	3523/9166	1.06(0.93-1.22)	0.369	51	0.00	22	3504/8822	1.20(1.05-1.38)	0.008	16	0.25
TP63	AD	rs10937405(C>T)	T vs C	3	1158/8484	0.82(0.75-0.90)	2.91×10^{-5}	0	0.898	3	1158/8484	0.79(0.69-0.90)	3.34×10^{-4}	0	0.69	3	1158/8484	0.75(0.62-0.90)	0.002	0	0.58
TP73	AD	G4C14/A4T14	AT vs GC							3	617/1532	1.06(0.82-1.38)	0.659	21	0.28						
XPA	AD	rs1800975(c.-4A>G,23G>A)	A vs G	3	637/1517	1.07(0.93-1.22)	0.334	0	0.990	4	715/1702	1.03(0.86-1.24)	0.743	0	0.66	4	715/1702	1.17(0.86-1.58)	0.324	36	0.20

Genes	Sub-group	Variants	Comparison[a]	Results of meta-analysis using allelic model						Results of meta-analysis using dominant model						Results of meta-analysis using recessive model					
				Studies	Cases/Controls	OR(95% CI)	*P* value	I^2(%)	P_Q[b]	Studies	Cases/Controls	OR(95% CI)	*P* value	I^2(%)	P_Q[b]	Studies	Cases/Controls	OR(95% CI)	*P* value	I^2(%)	P_Q[b]
XPC	AD	PAT-/+ (24660_24664delGTAAC, inspoly(AT))	Ins vs non-ins	3	398/1340	1.21(1.03-1.42)	0.020	0	0.442	3	398/1340	1.23(0.88-1.72)	0.218	47	0.15	3	398/1340	1.40(1.05-1.88)	0.024	0	0.99
XPC	AD	rs2228001(p.Gln939Lys,Ex16+211A＞C)	C vs A	3	1318/2108	1.06(0.96-1.17)	0.273	0	0.577												
XRCC1	AD	rs25489(p.Arg280His,c.839G>A,Ex9+16G>A)	A vs G	3	1067/1457	0.75(0.42-1.36)	0.343	83	0.002	3	795/2864	0.70(0.34-1.43)	0.322	86	0.00						
XRCC1	AD	rs1799782(p.Arg194Trp,c.580C>T,Ex6-22C＞T)	T vs C	5	1142/1829	0.95(0.80-1.14)	0.594	23	0.265	5	880/3276	0.97(0.80-1.19)	0.784	0	0.63	4	390/1144	1.40(0.82-2.38)	0.213	0	0.59
XRCC1	AD	rs25487(p.Gln399Arg,c.1196A>G)	A vs G	10	2359/6365	1.01(0.89-1.14)	0.917	58	0.011	10	1675/5800	1.04(0.88-1.24)	0.642	49	0.04	9	1607/5680	1.06(0.85-1.31)	0.599	22	0.25
XRCC1	AD	rs3213245(-77T>C)	C vs T							3	860/2178	1.55(1.29-1.87)	4.72×10^{-6}	0	0.76						
XRCC3	AD	rs861539(p.Thr241Met,c.722C>T)	T vs C	3	1108/1578	1.07(0.89-1.28)	0.494	33	0.227	3	424/1013	0.87(0.65-1.18)	0.377	27	0.26						
BIRC5	SCC	rs9904341(c.-31G>C）	C vs G	3	399/818	1.31(0.89-1.95)	0.175	73	0.024	3	399/818	1.03(0.78-1.35)	0.850	0	0.43	3	399/818	1.93(0.88-4.25)	0.102	82	0.00
CCND1	SCC	rs9344(rs603965,p.Pro241Pro,c.723G>A,c.870G>A)	A vs G	3	359/1264	1.05(0.70-1.57)	0.825	78	0.010	3	359/1264	0.96(0.55-1.69)	0.899	70	0.04	3	359/1264	1.11(0.58-2.10)	0.758	77	0.01
CDKN1A	SCC	rs1801270(p.Ser31Arg,c.93C>A,6829C>A)	A vs C	5	597/2043	1.12(0.78-1.60)	0.536	46	0.117	5	597/2043	1.20(0.80-1.81)	0.387	45	0.12	4	546/1948	0.88(0.48-1.60)	0.667	0	0.88
CXCL12	SCC	rs1801157(801G>A)	A vs G	3	198/815	1.30(0.65-2.60)	0.460	88	0.000	3	198/815	1.40(0.59-3.30)	0.444	85	0.00	3	198/815	1.51(0.52-4.35)	0.448	76	0.02
CYP1A1	SCC	rs4646903(m1,MspI,T3801C)	C vs T	17	1021/3959	1.45(1.26-1.67)	3.77×10^{-7}	21	0.215	24	1739/5212	1.51(1.27-1.79)	3.13×10^{-6}	39	0.03	16	976/3722	1.68(1.15-2.47)	0.008	34	0.09
CYP1A1	SCC	rs1048943(*2C,m2,p.Ile462Val,c.1384A>G)	G vs A	16	1572/3981	1.24(1.04-1.49)	0.020	58	0.002	20	2336/5091	1.23(0.98-1.56)	0.078	66	0.00	13	1365/3242	1.65(1.05-2.60)	0.030	53	0.01
CYP1A2	SCC	rs35694136(-2467delT)	Del vs non-del							3	309/494	1.58(0.97-2.56)	0.065	39	0.20						
CYP1A2	SCC	rs2069514(CYP1A2*1C,-3860G>A)	A vs G							3	292/411	1.13(0.39-3.27)	0.818	84	0.00						
CYP1A2	SCC	rs762551(CYP1A2*1F,c.A-164C)	C vs A							3	291/409	0.61(0.32-1.16)	0.132	66	0.06	3	305/494	1.05(0.53-2.10)	0.884	68	0.04
CYP2A6	SCC	non*4/*4(*1/*4,a deletion of the CYP2A6 gene)	Del vs non-del							4	516/1432	1.07(0.59-1.93)	0.826	79	0.00						
CYP2D6	SCC	phenotype (EM/HEM/PM)	PM vs EM	5	341/1153	0.63(0.30-1.31)	0.216	0	0.500	6	412/1229	1.04(0.81-1.34)	0.765	0	0.43	6	364/1203	0.57(0.29-1.14)	0.111	0	0.53
CYP2E1	SCC	rs6413432(c.967+1143T>A,7632T>A)	A vs T	6	715/1809	0.76(0.65-0.88)	3.98×10^{-4}	0	0.911	6	715/1809	0.77(0.64-0.92)	0.005	0	0.89	5	684/1771	0.52(0.34-0.82)	0.004	0	0.47
CYP2E1	SCC	rs2031920(RsaI,c1>c2,-1053C>T)	T vs C	7	962/3722	0.93(0.82-1.06)	0.287	0	0.661	7	962/3722	0.94(0.80-1.10)	0.414	0	0.55	6	880/3016	1.09(0.53-2.22)	0.818	57	0.04
ERCC2	SCC	rs1799793(p.Asp312Asn,c.981G>A)	A vs G	4	684/1704	1.00(0.57-1.77)	0.999	81	0.001	3	434/1019	1.20(0.69-2.07)	0.525	67	0.05	4	895/2039	4.62(0.7-30.53)	0.112	72	0.01
ERCC2	SCC	rs13181(p.Lys751Gln,c.2251A>C)	C vs A	3	476/1171	1.19(0.72-1.98)	0.496	57	0.098							3	698/1523	1.84(0.65-5.23)	0.250	48	0.14
GSTM1	SCC	Present/Null(Large gene deletion)	Null vs present	42	4704/12325	1.23(1.08-1.40)	0.002	66	0.000												
GSTP1	SCC	rs1695(p.Ile105Val, c.313A>G)	G vs A	12	1951/5051	1.01(0.93-1.09)	0.899	0	0.550	17	2502/6290	1.03(0.91-1.15)	0.658	23	0.19	12	1951/5051	1.14(0.92-1.41)	0.233	21	0.24
GSTT1	SCC	Present/Null(Large gene deletion)	Null vs present	23	2823/7727	1.07(0.93-1.24)	0.354	36	0.047												
IL10	SCC	rs1800871(c.-854T>C,-819C>T)	T vs C	3	165/1187	1.37(0.89-2.11)	0.149	55	0.106	3	165/1187	1.56(0.81-3.00)	0.183	66	0.05	3	165/1187	1.30(0.75-2.28)	0.350	0	0.81
IL1B	SCC	rs1143627(g.4970C>T,-31T>C)	C vs T	3	527/2067	0.90(0.78-1.04)	0.154	0	0.371	3	527/2067	0.92(0.67-1.28)	0.634	43	0.17	3	527/2067	0.77(0.54-1.10)	0.154	38	0.20
IL1B	SCC	rs16944(C-511T)	T vs C	3	544/2091	1.03(0.90-1.19)	0.663	0	0.583	3	544/2091	1.15(0.87-1.52)	0.311	31	0.23	3	544/2091	0.97(0.76-1.23)	0.770	0	0.40
IL6	SCC	rs1800795(G-174C)	C vs G	4	297/689	1.41(0.76-2.60)	0.277	85	0.000	4	297/689	1.48(0.75-2.91)	0.260	76	0.01	4	297/689	1.53(0.60-3.90)	0.373	69	0.02
IL6	SCC	rs1800796(-572C>G,634C>G)	G vs C							3	491/1452	0.71(0.49-1.03)	0.072	66	0.05						
IL8	SCC	rs4073(T-251A)	T vs A	3	1090/2541	1.05(0.79-1.38)	0.742	65	0.056	3	1090/2541	1.05(0.73-1.53)	0.790	51	0.13	3	1090/2541	1.01(0.72-1.43)	0.942	50	0.14
LTA	SCC	rs909253(A+252G)	G vs A	4	186/1260	0.97(0.77-1.23)	0.812	0	0.774	4	186/1260	1.07(0.73-1.58)	0.730	24	0.27	4	186/1260	0.74(0.42-1.29)	0.282	0	0.71
MDM2	SCC	rs2279744(SNP309,c.14+309T>G)	G vs T	7	1708/4408	1.11(0.94-1.30)	0.229	71	0.002	7	1708/4408	1.15(0.89-1.49)	0.283	70	0.00	7	1708/4408	1.15(0.94-1.39)	0.169	42	0.11
MIR146A	SCC	rs2910164(C>G)	C vs G													3	796/1751	1.32(0.93-1.86)	0.116	50	0.13
MMP2	SCC	rs243865(-1306C>T)	T vs C	3	740/1729	0.56(0.37-0.84)	0.005	73	0.023	3	740/1729	0.51(0.33-0.79)	0.002	71	0.03	3	740/1729	0.51(0.24-1.11)	0.089	0	0.50
MPO	SCC	rs2333227(G-463A)	A vs G	6	1572/4855	0.98(0.83-1.15)	0.791	53	0.061	6	1572/4855	0.95(0.79-1.14)	0.559	49	0.08	6	1572/4855	1.17(0.81-1.70)	0.396	34	0.18
NAT2	SCC	Rapid/slow(Acetylation Phenotype)	Slow vs rapid	10	1063/3136	0.94(0.72-1.24)	0.677	47	0.049												
NQO1	SCC	rs1800566(p.Pro187Ser,c.648C>T,609C>T,*2)	T vs C	3	408/1798	1.16(0.96-1.41)	0.125	0	0.707	5	648/2490	1.16(0.96-1.39)	0.127	0	0.82	3	408/1798	1.19(0.65-2.20)	0.578	0	0.94
OGG1	SCC	rs1052133(p.Ser326Cys,c.977C>G,8055C>G)	G vs C	9	2108/5170	1.03(0.95-1.11)	0.544	0	0.515	8	1858/4485	1.02(0.87-1.19)	0.843	15	0.31	9	1949/5515	1.09(0.92-1.28)	0.332	0	0.48
PADPRP	SCC	A/B(a 193-bp deletion)	Del vs non-del							4	103/339	0.87(0.53-1.41)	0.561	0	0.47						
PTGS2	SCC	rs5275(c.*427T>C,8473T>C)	C vs T							3	540/1105	0.91(0.74-1.13)	0.401	0	0.56						
SOD2	SCC	rs4880(rs1799725,Ala16Val)	T vs C	4	574/2761	1.54(1.03-2.31)	0.035	83	0.001	4	574/2761	1.44(1.13-1.84)	0.003	3	0.38	5	657/3093	1.50(0.89-2.52)	0.128	80	0.00
TERT	SCC	rs2736098(p.Ala305Ala,c.915G>A)	A vs G	4	788/2490	1.14(1.01-1.29)	0.031	0	0.597	4	788/2490	1.12(0.95-1.33)	0.179	0	0.76	4	788/2490	1.33(1.05-1.69)	0.019	0	0.61
TNF	SCC	rs1800629(G-308A,*1/*2)	A vs G	5	273/1500	1.85(1.17-2.93)	0.009	66	0.019	5	273/1500	1.98(1.24-3.16)	0.004	57	0.06	5	273/1500	2.38(0.82-6.91)	0.112	22	0.28
TP53	SCC	rs1042522(p.Pro72Arg,c.215C>G)	C vs G	21	3135/8809	0.99(0.84-1.17)	0.941	84	0.000	22	3350/9391	1.04(0.88-1.22)	0.654	69	0.00	22	3173/9047	0.95(0.70-1.30)	0.744	82	0.00
TP73	SCC	G4C14/A4T14	AT vs GC							3	438/1532	1.05(0.59-1.85)	0.881	83	0.00						
VEGFA	SCC	rs2010963(634G>C)	C vs G													3	381/920	0.84(0.58-1.21)	0.356	16	0.31
VEGFA	SCC	rs3025039(C936T)	T vs C							3	322/754	0.96(0.69-1.35)	0.812	24	0.27						
XPA	SCC	rs1800975(c.-4A>G,23G>A)	A vs G							3	631/1245	1.15(0.93-1.42)	0.211	0	0.38	3	631/1245	1.11(0.70-1.77)	0.650	68	0.05
XPC	SCC	PAT-/+ (24660_24664delGTAAC, inspoly(AT))	Ins vs non-ins	3	561/1340	1.12(0.88-1.42)	0.369	65	0.059	3	561/1340	1.00(0.80-1.24)	0.970	11	0.32	3	561/1340	1.43(0.94-2.19)	0.095	61	0.08
XPC	SCC	rs2228001(p.Gln939Lys,Ex16+211A＞C)	C vs A	3	789/2108	1.00(0.89-1.13)	0.995	0	0.427												
XRCC1	SCC	rs1799782(p.Arg194Trp,c.580C>T,Ex6-22C＞T)	T vs C	3	490/1429	0.88(0.73-1.06)	0.185	0	0.551	3	1147/2876	0.86(0.70-1.05)	0.136	0	0.85						
XRCC1	SCC	rs25487(p.Gln399Arg,c.1196A>G)	A vs G	7	2142/5112	0.99(0.86-1.13)	0.833	60	0.021	7	1923/4547	0.96(0.78-1.18)	0.713	60	0.02	6	1892/4427	1.03(0.82-1.28)	0.824	28	0.23

SCLC=small cell lung cancer; NSCLC=non-small cell lung cancer; AD=adenocarcinoma; SCC=squamous cell carcinoma; Del=deletion; Ins=insertion.

[a] Allelic contrast or phenotype trait for common variants; genetic comparison for rare variants or variants only with genotype group data

[b] *P* value of the test for between-study heterogeneity

Supplementary Table S6. Credibility assessment for variants with significant associations with lung cancer risk in subgroup meta-analyses by histological types

Genes	Sub-group	Variants	Alleles[a]	Number evaluated: Studies	Number evaluated: Cases/Controls	Lung cancer risk meta-analysis: Genetic models	OR(95% CI)[b]	*P* value	I^2 (%)	P_Q[c]	Venice criteria grades[d]	Credibility of evidence[e]	Amount of evidence[f]: Numbers	Amount of evidence: Grade	Replication[g]	Protection from bias[h]: Grade	Reasons for bias	Reason for bias exemption	Sensitivity analysis[i], Initial study influence: OR(95%CI)	*P* value	Deviation from HWE	Low OR[j]	Modified P^k	Excess P^l	Begg's P^m
CHRNA5	SCLC	rs16969968	A/G	3	513/3253	Allelic	1.55(1.15-2.10)	0.004	55	0.109	ACC	Weak	1813	A	C	C	Small study		1.37(1.12-1.67)	0.002	None	No	0.001	0.558	0.296
CYP1A1	SCLC	rs4646903	C/T	12	273/2545	Recessive	1.71(1.08-2.71)	0.021	0	0.904	BAA	Moderate	176	B	A	A			1.69(1.06-2.68)	0.026	NO	No	0.523	1.000	0.244
GSTM1	SCLC	Present/Null	NA	26	1224/7255	Null vs present	1.30(1.09-1.56)	0.004	43	0.01	ABA	Moderate	4063	A	B	A			1.28(1.06-1.54)	0.01	None	No	0.976	0.311	1.000
NQO1	SCLC	rs1800566	T/C	3	205/792	Allelic	1.68(1.05-2.68)	0.029	56	0.102	BCC	Weak	342	B	C	C	First study		1.44(0.95-2.17)	0.085	None	No	0.259	0.613	0.296
XPC	SCLC	rs2228001	C/A	3	347/2108	Allelic	0.84(0.71-1.00)	0.046	0	0.457	AAC	Weak	1843	A	A	C	First study		0.88(0.72-1.07)	0.192	None	No	0.218	1.000	1.000
CHRNA5	NSCLC	rs16969968	A/G	6	3201/4736	Allelic	1.36(1.24-1.48)	1.48×10^{-11}	13	0.329	AAA	Strong	4303	A	A	A			1.34(1.23-1.45)	2.90×10^{-12}	None	No	0.736	0.672	0.707
CLPTM1L	NSCLC	rs402710	T/C	6	2940/4040	Allelic	0.85(0.79-0.91)	1.13×10^{-5}	0	0.666	AAA	Strong	4634	A	A	A			0.81(0.74-0.89)	7.73×10^{-6}	NO	No	0.650	1.000	1.000
CYP1A1	NSCLC	rs4646903	C/T	24	3032/5165	Allelic	1.11(1.03-1.21)	0.009	0	0.458	AAC	Weak	3650	A	A	C	Low OR		1.09(1.01-1.19)	0.038	NO	Yes	0.986	0.194	0.785
CYP2E1	NSCLC	rs6413432	A/T	6	1290/1809	Allelic	0.80(0.71-0.91)	4.90×10^{-4}	0	0.868	AAA	Strong	1372	A	A	A			0.81(0.71-0.92)	0.001	NO	No	0.880	1.000	1.000
ERCC1	NSCLC	rs11615	C/T	3	780/811	Allelic	0.68(0.58-0.81)	1.01×10^{-5}	13	0.316	AAA	Strong	2162	A	A	A			0.75(0.61-0.91)	0.004	None	No	0.311	0.547	0.296
ERCC2	NSCLC	rs13181	C/A	8	2770/2856	Allelic	1.19(1.04-1.36)	0.014	39	0.116	ABC	Weak	2381	A	B	C	HWE		1.22(1.06-1.40)	0.006	Yes	No	0.776	0.665	1.000
FAS	NSCLC	rs2234767	A/G	3	3194/3064	Recessive	1.51(1.23-1.87)	1.26×10^{-4}	0	0.447	BAC	Weak	396	B	A	C	First study		1.38(0.95-2.00)	0.093	None	No	0.478	1.000	0.296
FGFR4	NSCLC	rs351855	A/G	3	985/1230	Allelic	0.76(0.68-0.86)	1.97×10^{-5}	0	0.59	AAA	Strong	1779	A	A	A			0.74(0.64-0.86)	5.19×10^{-5}	None	No	0.323	0.590	1.000
GSTM1	NSCLC	Present/Null	NA	60	11551/15673	Null vs present	1.18(1.08-1.29)	1.56×10^{-4}	62	0	ACA	Weak	13759	A	C	A			1.17(1.08-1.28)	3.23×10^{-4}	None	No	0.119	0.213	0.448
HYKK	NSCLC	rs931794	G/A	4	1548/2464	Allelic	1.25(1.13-1.37)	9.08×10^{-6}	0	0.88	AAA	Strong	2718	A	A	A			1.25(1.07-1.46)	0.004	None	No	0.902	0.630	0.734
IL17A	NSCLC	rs2275913	A/G	3	780/998	Recessive	1.72(1.12-2.65)	0.013	31	0.235	BBB	Moderate	164	B	B	B	Missing information		2.01(1.40-2.87)	1.41×10^{-4}	NA	No	0.405	0.566	0.296
IL1B	NSCLC	rs1143627	C/T	5	1915/2551	Allelic	0.82(0.67-0.99)	0.043	78	0.001	ACC	Weak	4019	A	C	C	First study, HWE ,small study, publication bias		0.83(0.66-1.05)	0.117	Yes	No	0.019	0.676	0.027
IL6	NSCLC	rs1800796	G/C	3	1333/1452	Allelic	0.75(0.57-0.99)	0.039	77	0.013	ACC	Weak	1473	A	C	C	Small study		0.75(0.57-0.99)	0.039	None	No	0.036	0.227	0.296
MIR146A	NSCLC	rs2910164	C/G	4	880/1094	Allelic	1.28(1.11-1.46)	4.63×10^{-4}	0	0.391	AAA	Strong	1765	A	A	A			1.28(1.11-1.46)	4.63×10^{-4}	None	No	0.327	0.629	0.734
MMP2	NSCLC	rs243865	T/C	3	1216/1729	Allelic	0.61(0.40-0.92)	0.019	83	0.003	BCC	Weak	829	B	C	C	First study, small study		0.72(0.33-1.59)	0.419	None	No	0.025	0.456	0.296
MPO	NSCLC	rs2333227	A/G	10	5036/6646	Recessive	1.38(1.03-1.86)	0.033	51	0.031	BCA	Weak	576	B	C	A			1.41(1.03-1.94)	0.035	None	No	0.881	0.700	0.858
OGG1	NSCLC	rs1052133	G/C	17	6279/8214	Recessive	1.16(1.02-1.32)	0.02	36	0.072	ABC	Weak	2814	A	B	C	Excess of significant studies		1.15(1.01-1.32)	0.039	NO	No	0.920	0.084	0.967
SOD2	NSCLC	rs4880	T/C	6	3133/4255	Allelic	1.25(1.08-1.45)	0.002	72	0.003	ACC	Weak	7713	A	C	C	Small study		1.31(1.07-1.60)	0.008	NO	No	0.090	1.000	0.133
TERT	NSCLC	rs2736098	A/G	4	2002/2490	Allelic	1.30(1.19-1.42)	2.59×10^{-9}	0	0.818	AAA	Strong	3368	A	A	A			1.29(1.17-1.42)	2.09×10^{-7}	None	No	0.775	1.000	0.734
TNF	NSCLC	rs1800629	A/G	6	1052/1767	Allelic	1.67(1.01-2.76)	0.044	89	0	BCC	Weak	829	B	C	C	First study		1.82(0.91-3.65)	0.091	None	No	0.432	0.209	0.452
TP63	NSCLC	rs10937405	T/C	3	3587/8484	Allelic	0.87(0.82-0.92)	9.91×10^{-7}	0	0.595	AAB	Moderate	10101	A	A	B	Missing information		NA		None	No	0.402	1.000	1.000
XPC	NSCLC	PAT-/+	Ins/non-ins	3	967/1340	Recessive	1.46(1.17-1.81)	0.001	0	0.483	BAA	Moderate	383	B	A	A			1.36(1.05-1.76)	0.020	None	No	0.888	0.591	1.000
XRCC1	NSCLC	rs2256507	A/G	3	431/779	Recessive	1.68(1.03-2.74)	0.038	8	0.338	CAB	Weak	80	C	A	B	Missing information		NA		None	No	0.281	0.499	1.000
XRCC1	NSCLC	rs1001581	T/C	4	574/994	Allelic	1.27(1.04-1.56)	0.018	40	0.172	ABC	Weak	1134	A	B	C	First study		1.28(0.87-1.89)	0.218	None	No	0.187	0.592	0.734
XRCC1	NSCLC	rs3213245	C/T	3	1744/2178	Dominant	1.50(1.29-1.75)	1.89×10^{-7}	0	0.683	BAA	Moderate	847	B	A	A			1.53(1.26-1.84)	1.14×10^{-5}	None	No	0.228	0.575	0.296
BIRC5	AD	rs9904341	C/G	3	312/818	Allelic	1.70(1.22-2.37)	0.002	57	0.097	ACA	Weak	1142	A	C	A			2.03(1.46-2.83)	2.33×10^{-5}	None	No	0.189	0.562	0.296
CHRNA5	AD	rs16969968	A/G	4	1507/2834	Allelic	1.37(1.14-1.64)	0.001	33	0.214	ABA	Moderate	1867	A	B	A			1.32(1.16-1.51)	3.06×10^{-5}	None	No	0.861	1.000	0.734
CYP2E1	AD	rs6413432	A/T	6	500/1809	Allelic	0.79(0.66-0.95)	0.011	0	0.664	AAA	Strong	1040	A	A	A			0.80(0.67-0.97)	0.019	NO	No	0.882	1.000	0.707
ERCC2	AD	rs13181	C/A	4	664/1230	Dominant	1.35(1.06-1.70)	0.013	0	0.635	BAA	Moderate	607	B	A	A			1.39(1.08-1.80)	0.010	None	No	0.489	1.000	0.734
GSTM1	AD	Present/Null	NA	42	4199/12206	Null vs present	1.17(1.05-1.31)	0.005	45	0.001	ABC	Weak	8165	A	B	C	Small study,excess of significant studies		1.14(1.03-1.26)	0.011	None	No	0.075	0.039	0.146
IL17A	AD	rs2275913	A/G	3	469/998	Recessive	1.84(1.11-3.06)	0.018	36	0.211	BBB	Moderate	132	B	B	B	Missing information		2.19(1.47-3.26)	1.21×10^{-4}	NA	No	0.570	0.567	1.000
MDM2	AD	rs2279744	G/T	6	1714/4083	Recessive	1.28(1.04-1.56)	0.018	46	0.098	ABA	Moderate	1296	A	B	A			1.34(1.06-1.70)	0.015	None	No	0.792	0.668	0.707
MIR146A	AD	rs2910164	C/G	3	442/965	Allelic	1.26(1.04-1.51)	0.018	15	0.309	AAC	Weak	1311	A	A	C	First study		1.33(0.89-1.99)	0.161	None	No	0.202	1.000	0.296
OGG1	AD	rs1052133	G/C	12	3603/6677	Recessive	1.25(1.10-1.43)	0.001	20	0.246	AAA	Strong	2238	A	A	A			1.25(1.09-1.44)	0.002	None	No	0.777	0.263	0.945
SOD2	AD	rs4880	T/C	4	1219/2761	Allelic	1.20(1.08-1.32)	3.80×10^{-4}	0	0.405	AAC	Weak	4235	A	A	C	First study		1.28(0.98-1.68)	0.075	NO	No	0.104	0.150	0.308
TERT	AD	rs2736098	A/G	4	1214/2490	Allelic	1.40(1.26-1.54)	4.97×10^{-11}	0	0.891	AAA	Strong	2771	A	A	A			1.39(1.24-1.55)	2.68×10^{-8}	None	No	0.533	1.000	0.308
TP53	AD	rs1042522	C/G	22	3504/8822	Recessive	1.20(1.05-1.38)	0.008	16	0.245	AAA	Strong	1835	A	A	A			1.20(1.04-1.39)	0.015	NO	No	0.210	1.000	0.143
TP63	AD	rs10937405	T/C	3	1158/8484	Allelic	0.82(0.75-0.90)	2.91×10^{-5}	0	0.898	AAB	Moderate	8115	A	A	B	Missing information		NA		None	No	0.168	1.000	0.296
XPC	AD	PAT-/+	Ins/non-ins	3	398/1340	Allelic	1.21(1.03-1.42)	0.02	0	0.442	AAC	Weak	1391	A	A	C	First study		1.18(0.94-1.47)	0.149	None	No	0.999	0.575	1.000
XRCC1	AD	rs3213245	C/T	3	860/2178	Dominant	1.55(1.29-1.87)	4.72×10^{-6}	0	0.758	BAA	Moderate	630	B	A	A			1.58(1.25-2.00)	1.25×10^{-4}	None	No	0.192	0.557	0.296
CYP1A1	SCC	rs4646903	C/T	17	1021/3959	Allelic	1.45(1.26-1.67)	3.77×10^{-7}	21	0.215	AAA	Strong	2102	A	A	A			1.42(1.22-1.65)	6.56×10^{-6}	NO	No	0.571	0.415	0.232
CYP1A1	SCC	rs1048943	G/A	16	1572/3981	Allelic	1.24(1.04-1.49)	0.02	58	0.002	ACC	Weak	2328	A	C	C	Small study		1.23(1.01-1.49)	0.038	NO	No	0.073	0.282	0.444
CYP2E1	SCC	rs6413432	A/T	6	715/1809	Allelic	0.76(0.65-0.88)	3.98×10^{-4}	0	0.911	AAA	Strong	1133	A	A	A			0.76(0.65-0.89)	0.001	NO	No	0.389	1.000	0.260
GSTM1	SCC	Present/Null	NA	42	4704/12325	Null vs present	1.23(1.08-1.40)	0.002	66	0	ACC	Weak	8383	A	C	C	Excess of significant studies		1.23(1.08-1.41)	0.003	None	No	0.291	0.025	0.965
MMP2	SCC	rs243865	T/C	3	740/1729	Allelic	0.56(0.37-0.84)	0.005	73	0.023	BCC	Weak	723	B	C	C	First study, small study		0.66(0.32-1.33)	0.242	None	No	0.046	0.448	1.000
SOD2	SCC	rs4880	T/C	4	574/2761	Allelic	1.54(1.03-2.31)	0.035	83	0.001	ACC	Weak	3495	A	C	C	First study		1.93(0.75-4.99)	0.174	NO	No	0.319	0.314	0.734
TERT	SCC	rs2736098	A/G	4	788/2490	Allelic	1.14(1.01-1.29)	0.031	0	0.597	AAC	Weak	2329	A	A	C	Low OR		1.17(1.03-1.33)	0.017	None	Yes	0.389	0.535	1.000
TNF	SCC	rs1800629	A/G	5	273/1500	Allelic	1.85(1.17-2.93)	0.009	66	0.019	BCC	Weak	488	B	C	C	First study		1.98(0.95-4.16)	0.070	None	No	0.720	1.000	1.000

SCLC=small cell lung cancer; NSCLC=non-small cell lung cancer; AD=adenocarcinoma; SCC=squamous cell carcinoma; Ins=insertion; NA=Not Applicable.

[a] Minor alleles/major alleles (per Caucasian)

[b] Summary ORs were calculated based on the DerSimonian-Lairs random-effects model

[c] *P*-value of heterogeneity between studies

[d] Venice criteria grades contained assessments of three aspects: the amount of evidence, extent of replication, and protection from bias, and each assessment was assigned three levels (A, B, or C).

[e] Credibility of evidence was evaluated based on Venice criteria with three aspects (the amount of evidence, extent of replication, and protection from bias), and was categorized as strong (all three aspect grades were A), moderate (any grade was B, but not C) or weak (any grade was C).

[f] Amount of evidence, depending on total sample size of the smallest genetic group among cases and controls in each meta-analysis, was graded as A (sample size>1000), B (sample size between 100 and 1000), or C (sample size <100).

[g] Extent of replication, depending on between-study heterogeneity, was graded as A (I^2<25%), B (I^2 between 25% and 50%), or C (I^2>50%).

[h] Protection from bias, considering various potential sources of bias in meta-analysis, was graded as A when there was no demonstrable bias and bias was unlikely invalidate the association, was graded as B when there was insufficient information for identifying evidence (i.e. missing information for evaluating Hardy-Weinberg equilibrium (HWE) among controls in an individual study) although there was no obvious bias, and C when the bias was evident and/or was likely to explain the presence of association. For the C grade, it was assigned if a meta-analysis had any of the following potential sources of bias: (1) magnitude of the association was low; (2) sensitivity analysis altered the statistical significance; (3) potential small study effect; (4) possible existence of an excess of significant findings (the number of observed significant studies more than the number of expected significant studies); (5) potential publication bias.

Small study=Possible existence of potential small study effect (modified Egger's test, *p*<0.10); First study=Significance lost excluding the first published or first positive report; Low OR=Low magnitude of the association (i.e. 0.87<OR<1.15); HWE=Significance lost excluding studies with controls violating HWE; Missing information= Insufficient information for bias evaluation; Publication bias= Possible existence of potential publication bias (Begg's test, *p*<0.10); Excess of significant studies=Possible existence of an excess of significant findings (excess significance test, *p*<0.10).

[i] A sensitivity analysis was performed to examine whether the significant summary ORs were robust after excluding the first published or first positive report (defined as the earliest study reported a significant association), or excluding studies with controls violating HWE. None=A meta-analysis didn't include studies with controls violating HWE or evaluated association for a variant with a phenotype change; NO=Without the influence from studies with controls violating HWE; Yes=Evidence of influence from studies with controls violating HWE; NA=Insufficient information for evaluation of influence from first published/positive study or studies with controls violating HWE.

[j] Low OR (i.e. 0.87<OR<1.15; No=Association without a low OR; Yes=Association with a low OR)

[k] *P* value for potential small study effect based on the modified Egger's test. (*P*<0.10 is usually considered evidence for significant evidence of small study effect.)

[l] *P* value for possible existing an excess of significant findings based on the excess significance test. (*P*<0.10 is usually considered evidence for significant evidence of an excess of significant findings.)

[m] *P* value for potential publication bias of studies based on the Begg's test. (*P*<0.10 is usually considered evidence for significant evidence of publication bias.)

Supplementary Table S7. Subgroup meta-analyses stratified by smoking status for variants with sufficient data under different genetic models (Continued on next page)

Genes	Sub-group	Variants	Comparison[a]	Results of meta-analysis using allelic model						Results of meta-analysis using dominant model						Results of meta-analysis using recessive model					
				Studies	Cases/Controls	OR(95% CI)	*P* value	I^2(%)	P_Q[b]	Studies	Cases/Controls	OR(95% CI)	*P* value	I^2(%)	P_Q[b]	Studies	Cases/Controls	OR(95% CI)	*P* value	I^2(%)	P_Q[b]
APEX1	Smokers	rs1760944(c.-468T>G,-141T>G)	A vs C	3	655/647	1.37(1.11-1.69)	0.003	43	0.174												
APEX1	Smokers	rs1130409(p.Asp148Glu,c.444T>G)	G vs T	7	1604/1983	0.97(0.86-1.10)	0.678	39	0.133	11	4074/3854	1.15(0.97-1.36)	0.100	64	0.002	6	1462/1811	1.03(0.79-1.34)	0.827	54	0.054
CCND1	Smokers	rs9344(rs603965,p.Pro241Pro,c.870G>A)	A vs G	4	1250/1709	1.14(0.86-1.53)	0.368	82	0.001	4	1250/1709	1.17(0.74-1.86)	0.511	78	0.004	4	1250/1709	1.21(0.78-1.86)	0.393	81	0.002
CHRNA5	Smokers	rs16969968(p.Asp398Asn,c.1192G>A)	A vs G	4	2828/3738	1.30(1.20-1.42)	2.77×10^{-10}	0	0.626	4	2828/3738	1.42(1.26-1.59)	4.52×10^{-9}	0	0.611	4	2828/3738	1.43(1.21-1.69)	2.08×10^{-5}	0	0.842
CYP1A1	Smokers	rs4646903(m1,MspI,T3801C)	C vs T	7	1034/1087	1.30(1.02-1.64)	0.033	46	0.088	21	3225/3067	1.38(1.14-1.67)	0.001	58	0.000	9	1154/1218	1.57(1.03-2.38)	0.034	18	0.285
CYP1A1	Smokers	rs1048943(*2C,m2,p.Ile462Val,c.1384A>G)	G vs A	8	1166/1586	1.25(0.97-1.62)	0.081	62	0.010	14	1888/2413	1.42(1.04-1.93)	0.028	78	0.000	8	1166/1586	1.51(0.75-3.03)	0.251	45	0.077
CYP1A2	Smokers	rs2069526(-739T>G)	G vs T							3	412/401	1.89(0.96-3.71)	0.065	46	0.155						
CYP1A2	Smokers	rs2069514(CYP1A2*1C,-3860G>A)	A vs G							4	534/435	1.40(0.47-4.16)	0.547	85	0.000						
CYP1A2	Smokers	rs762551(CYP1A2*1F,c.A-164C)	C vs A							3	507/544	0.90(0.42-1.94)	0.783	83	0.003	3	553/555	0.80(0.37-1.73)	0.573	75	0.018
CYP1B1	Smokers	rs10012(p.Arg48Gly,c.142C>G)	G vs C							3	542/528	1.86(0.94-3.68)	0.075	81	0.005						
CYP1B1	Smokers	rs1056827(p.Ala119Ser,c.355G>T,m2)	T vs G							3	495/491	1.48(0.72-3.04)	0.286	83	0.003						
CYP1B1	Smokers	rs1800440(p.Asn453Ser,Ex3+315A>G)	G vs A							3	447/449	0.70(0.51-0.95)	0.024	0	0.481						
CYP1B1	Smokers	rs1056836(CYP1B1*3,p.Leu432Val,c.1294C>G)	G vs C							5	622/684	1.28(1.01-1.62)	0.045	0	0.616						
CYP2A6	Smokers	rs5031016(p.Ile471Thr,c1412T>C,wt/*7)	C vs T	3	1527/1138	0.57(0.33-1.00)	0.048	73	0.025	3	1527/1138	0.58(0.34-0.98)	0.043	66	0.055						
CYP2A6	Smokers	non*4/*4(*1/*4,a deletion of the CYP2A6 gene)	*4 vs non*4	3	1339/848	0.71(0.59-0.85)	2.30×10^{-4}	13	0.319	4	1449/1022	0.80(0.54-1.20)	0.281	70	0.019	3	1339/848	0.44(0.28-0.71)	0.001	0	0.768
CYP2C9	Smokers	rs1799853(p.Arg144Cys,c.430C>T)	T vs C							3	637/858	1.22(0.87-1.69)	0.249	24	0.266						
CYP2E1	Smokers	rs3813867(g.3739G>C,c.-1293G>C)	C vs G							4	595/639	1.07(0.67-1.70)	0.788	24	0.267						
CYP2E1	Smokers	rs6413432(c.967+1143T>A,7632T>A)	A vs T	3	796/791	0.75(0.63-0.90)	0.002	2	0.360	6	960/944	0.57(0.37-0.87)	0.010	55	0.047						
CYP2E1	Smokers	rs2031920(RsaI,c1>c2,-1053C>T)	T vs C	3	1064/1220	0.76(0.65-0.90)	0.001	0	0.727	10	1880/1949	0.76(0.63-0.91)	0.003	15	0.304						
EPHX1	Smokers	rs1051740(p.Tyr113His,c.337T>C)	C vs T	5	840/1270	0.87(0.57-1.33)	0.526	88	0.000	6	885/1315	0.98(0.56-1.75)	0.956	88	0.000	6	1074/1364	0.99(0.60-1.63)	0.971	66	0.011
EPHX1	Smokers	rs2234922(p.His139Arg,c.416A>G)	G vs A	5	817/1230	1.26(0.89-1.78)	0.200	76	0.002	6	862/1275	1.34(0.87-2.05)	0.182	77	0.001	5	909/845	1.42(1.02-1.97)	0.039	0	0.535
ERCC1	Smokers	rs2298881(262G>T,C>A)	T vs G							3	1600/2132	0.98(0.74-1.30)	0.878	65	0.059						
ERCC1	Smokers	rs3212986(8092C>A,14443C>A)	A vs C	3	1701/2361	1.01(0.92-1.12)	0.801	0	0.479	3	1701/2361	1.03(0.91-1.17)	0.614	0	0.844	3	1701/2361	1.00(0.70-1.43)	0.982	46	0.155
ERCC1	Smokers	rs3212961(IVS5+33C＞A,17677C>A)	A vs C	3	1125/1074	0.93(0.81-1.07)	0.305	0	0.658	3	1190/1067	0.93(0.77-1.13)	0.478	0	0.465						
ERCC1	Smokers	rs11615(rs3177700,p.Asn118Asn,c.354T>C)	C vs T	5	2011/2486	0.84(0.69-1.04)	0.112	76	0.003	4	1861/2316	0.77(0.47-1.25)	0.290	80	0.002	4	1861/2316	0.77(0.58-1.03)	0.083	68	0.024
ERCC2	Smokers	rs1799793(p.Asp312Asn,c.981G>A)	A vs G	5	1877/2526	0.98(0.88-1.10)	0.712	24	0.261	6	1973/2609	0.91(0.79-1.05)	0.178	16	0.314	5	1877/2526	1.10(0.91-1.32)	0.323	0	0.577
ERCC2	Smokers	rs13181(p.Lys751Gln,c.2251A>C)	C vs A	5	1629/2452	1.07(0.93-1.24)	0.341	49	0.101	5	1576/2363	1.03(0.85-1.25)	0.772	39	0.159	5	1551/2341	1.12(0.93-1.34)	0.232	0	0.491
ERCC4	Smokers	rs1800067(Arg415Gln)	A vs G	3	1110/1807	1.09(0.89-1.34)	0.404	0	0.650												
ERCC5	Smokers	rs17655(p.Asp1104His,c.3310G>C)	C vs G	3	1092/1743	0.98(0.77-1.26)	0.886	57	0.096												
EXO1	Smokers	rs1047840(p.Glu589Lys,c.1765G>A)	A vs G							3	884/840	1.23(0.80-1.90)	0.342	79	0.008						
GSTM1	Smokers	Present/Null(Large gene deletion)	Null vs present	45	9276/9005	1.20(1.09-1.32)	2.16×10^{-4}	51	0.000												
GSTM3	Smokers	rs1799735(c.468+21delAGG,3bp deletion,*A/*B)	Del vs non-del							3	645/624	0.93(0.66-1.31)	0.686	41	0.183						
GSTP1	Smokers	rs1138272(p.Ala114Val,c.341C>T)	T vs C							3	924/1026	1.63(1.28-2.08)	9.17×10^{-5}	0	0.459						
GSTP1	Smokers	rs1695(p.Ile105Val,c.313A>G)	G vs A	8	2830/2178	1.29(1.09-1.53)	0.003	59	0.017	20	5159/4713	1.22(1.07-1.38)	0.003	46	0.014	8	2830/2178	1.37(1.07-1.77)	0.014	23	0.250
GSTT1	Smokers	Present/Null (Large gene deletion)	Null vs present	27	7032/6386	1.08(0.95-1.22)	0.263	41	0.014												
IL1B	Smokers	rs1143627(g.4970C>T,-31T>C)	C vs T							3	2180/1934	1.12(0.99-1.27)	0.086	0	0.408						
IL6	Smokers	rs1800795(G-174C)	C vs G							3	2471/2002	0.93(0.82-1.06)	0.295	0	0.462						
IL8	Smokers	rs4073(T-251A)	T vs A													3	2605/2081	1.00(0.88-1.14)	0.983	0	0.615
LIG1	Smokers	rs20580(p.Ala170Ala,c.417C>A)	A vs C	4	1367/2175	1.06(0.96-1.18)	0.256	6	0.364	3	1225/2004	1.11(0.94-1.31)	0.228	0	0.516	3	1225/2004	1.15(0.98-1.37)	0.095	0	0.898
MDM2	Smokers	rs2279744(SNP309,c.14+309T>G)	G vs T	4	2941/4189	1.01(0.80-1.28)	0.932	90	0.000	4	2941/4189	0.96(0.71-1.31)	0.790	88	0.000	4	2941/4189	1.12(0.83-1.51)	0.466	78	0.004
MMP1	Smokers	rs1799750(-16071G/2G,insG)	2G vs 1G													3	2400/1389	1.32(0.85-2.04)	0.211	84	0.002
MPO	Smokers	rs2333227(G-463A)	A vs G	9	2987/2720	0.99(0.86-1.14)	0.893	50	0.045	9	2987/2720	1.00(0.87-1.13)	0.946	21	0.254	10	3190/2887	1.06(0.68-1.65)	0.814	70	0.001
MTHFR	Smokers	rs1801131(p.Ala429Glu,c.1286A>C,A1298C)	C vs A	3	1245/1383	1.14(1.01-1.27)	0.031	0	0.871	3	1245/1383	1.16(0.99-1.35)	0.060	0	0.999	4	1423/1587	1.18(0.94-1.48)	0.164	0	0.559
MTHFR	Smokers	rs1801133(p.Ala222Val,Ex4+79C＞T)	T vs C	4	1531/1947	0.93(0.79-1.08)	0.324	40	0.171	4	1531/1947	0.87(0.74-1.03)	0.100	23	0.274	5	1840/2117	1.27(0.82-1.97)	0.287	70	0.011
NAT2	Smokers	rs1799930(p.Arg197Gln,590G>A,NAT2*6B)	A vs G	3	428/476	0.81(0.66-0.99)	0.044	0	0.453	4	662/570	0.91(0.63-1.32)	0.621	59	0.064	3	428/476	0.82(0.52-1.30)	0.402	0	0.402
NAT2	Smokers	Rapid/slow (Acetylation Phenotype)	Slow vs rapid	7	1290/1379	1.08(0.80-1.44)	0.623	55	0.037												
NBN	Smokers	rs1805794(p.Gln185Glu,Ex5-32C>G,605C>G)	G vs C													3	1226/1220	0.83(0.71-0.98)	0.030	0	0.554
NQO1	Smokers	rs1800566(p.Pro187Ser,c.648C>T,609C>T,*2)	T vs C	5	1687/1677	1.10(0.90-1.36)	0.355	58	0.049	13	3231/3021	1.04(0.90-1.20)	0.586	33	0.118	5	1687/1677	1.17(0.72-1.93)	0.527	42	0.140
OGG1	Smokers	rs1052133(p.Ser326Cys,c.977C>G,8055C>G)	G vs C	13	4847/3994	0.99(0.86-1.15)	0.900	73	0.000	13	4829/3926	0.99(0.83-1.17)	0.901	55	0.009	12	4704/3834	1.06(0.82-1.37)	0.668	67	0.000
PADPRP	Smokers	A/B(a 193-bp deletion)	Del vs non-del							3	265/202	1.30(0.65-2.58)	0.458	63	0.069						
PTGS2	Smokers	rs5275(c.*427T>C,8473T>C)	C vs T							4	2629/2077	1.06(0.65-1.70)	0.826	90	0.000						
SOD2	Smokers	rs4880(rs1799725,Ala16Val)	T vs C	3	1444/1188	1.25(1.06-1.48)	0.007	33	0.224	3	1444/1188	1.58(1.00-2.48)	0.049	70	0.037	5	1681/1456	1.23(1.04-1.46)	0.013	0	0.438
SULT1A1	Smokers	rs9282861(p.Arg213His,c.638G>A,*1/*2)	A vs G							5	1381/1377	1.23(0.81-1.89)	0.335	83	0.000						
TP53	Smokers	rs1042522(p.Pro72Arg,c.215C>G)	C vs G	7	1887/1830	1.15(0.90-1.48)	0.271	81	0.000	9	2747/2426	1.23(1.01-1.50)	0.035	57	0.017	10	2771/2299	1.07(0.75-1.55)	0.701	76	0.000
XPA	Smokers	rs1800975(c.-4A>G,23G>A)	A vs G	3	1459/2213	0.96(0.78-1.18)	0.674	74	0.021	4	1678/2350	0.98(0.74-1.29)	0.865	71	0.017	3	1459/2213	1.04(0.69-1.57)	0.851	76	0.016
XPC	Smokers	rs2228001(p.Gln939Lys,Ex16+211A＞C)	C vs A	3	1092/1742	0.98(0.87-1.09)	0.689	0	0.582												
XRCC1	Smokers	rs25489(p.Arg280His,c.839G>A,Ex9+16G>A)	A vs G	3	724/762	1.35(1.00-1.81)	0.048	0	0.589	6	3197/2724	1.09(0.91-1.31)	0.356	8	0.366	3	724/762	1.45(0.36-5.85)	0.600	0	0.518
XRCC1	Smokers	rs1799782(p.Arg194Trp,c.580C>T,Ex6-22C＞T)	T vs C	6	1597/1987	0.73(0.55-0.97)	0.028	40	0.137	7	3832/3699	0.77(0.60-0.99)	0.038	49	0.066	5	1310/1554	0.74(0.37-1.45)	0.375	0	0.904
XRCC1	Smokers	rs25487(p.Gln399Arg,c.1196A>G)	A vs G	12	4087/3678	0.93(0.84-1.02)	0.138	33	0.124	15	4650/4287	0.98(0.90-1.06)	0.582	0	0.692	12	4087/3678	0.79(0.63-1.00)	0.050	41	0.068
XRCC3	Smokers	rs861539(p.Thr241Met,c.722C>T)	T vs C	3	1312/1557	1.05(0.81-1.36)	0.710	73	0.005	7	1475/1698	1.13(0.83-1.54)	0.451	71	0.002	5	1312/1557	1.09(0.72-1.64)	0.686	44	0.127
APEX1	Non-smokers	rs1130409(p.Asp148Glu,c.444T>G)	G vs T	5	789/1103	1.04(0.91-1.19)	0.545	0	0.443	10	1544/2531	1.02(0.89-1.17)	0.770	0	0.664	5	789/1103	1.16(0.91-1.48)	0.222	0	0.457
ATM	Non-smokers	rs189037(c.-111G>A,−4519G>A)	A vs G	3	1323/1550	1.18(1.03-1.36)	0.018	42	0.178	3	1323/1550	1.15(0.95-1.38)	0.147	25	0.262	3	1323/1550	1.40(1.16-1.69)	3.87×10^{-4}	0	0.369
CCND1	Non-smokers	rs9344(rs603965,p.Pro241Pro,c.723G>A)	A vs G	3	161/262	1.21(0.79-1.84)	0.384	51	0.131	3	161/262	1.16(0.60-2.22)	0.657	28	0.248	4	371/433	1.37(0.85-2.19)	0.197	44	0.149
CYP1A1	Non-smokers	rs4646903(m1,MspI,T3801C)	C vs T	6	412/869	0.94(0.75-1.19)	0.611	18	0.295	19	998/2214	0.95(0.78-1.15)	0.586	20	0.208	8	508/1081	1.07(0.74-1.55)	0.716	0	0.461
CYP1A1	Non-smokers	rs1048943(*2C,m2,p.Ile462Val,c.1384A>G)	G vs A	4	446/561	1.16(0.86-1.56)	0.339	43	0.152	10	926/1327	1.01(0.76-1.35)	0.927	51	0.032	4	446/561	1.93(1.03-3.63)	0.042	32	0.218
CYP1B1	Non-smokers	rs1056836(CYP1B1*3,p.Leu432Val,c.1294C>G)	G vs C							4	396/536	1.03(0.67-1.57)	0.908	37	0.189						
CYP2E1	Non-smokers	rs6413432(c.967+1143T>A,7632T>A)	A vs T							5	315/560	0.72(0.54-0.97)	0.028	0	0.959						
CYP2E1	Non-smokers	rs2031920(RsaI,c1>c2,-1053C>T)	T vs C	3	304/695	0.70(0.54-0.90)	0.005	0	0.863	9	593/1181	0.71(0.55-0.91)	0.008	12	0.336						
ERCC1	Non-smokers	rs11615(rs3177700,p.Asn118Asn,c.354T>C)	C vs T	3	731/958	0.85(0.72-0.99)	0.042	0	0.449	3	731/958	0.68(0.47-1.00)	0.051	0	0.422	3	731/958	0.86(0.71-1.05)	0.136	0	0.585
ERCC2	Non-smokers	rs1799793(p.Asp312Asn,c.981G>A)	A vs G							3	404/399	1.31(0.82-2.09)	0.263	32	0.231						
ERCC2	Non-smokers	rs13181(p.Lys751Gln,c.2251A>C)	C vs A							3	478/469	1.88(1.36-2.58)	1.11×10^{-4}	0	0.550	3	421/444	0.95(0.33-2.70)	0.916	0	0.441
GSTM1	Non-smokers	Present/Null (Large gene deletion)	Null vs present	32	1924/4718	1.37(1.16-1.61)	1.60×10^{-4}	41	0.009												
GSTP1	Non-smokers	rs1695(p.Ile105Val,c.313A>G)	G vs A	3	380/763	0.96(0.78-1.18)	0.690	0	0.861	14	836/1890	1.06(0.81-1.39)	0.666	45	0.034	3	380/763	1.03(0.62-1.74)	0.899	0	0.540
GSTT1	Non-smokers	Present/Null (Large gene deletion)	Null vs present	18	965/2958	1.26(0.95-1.67)	0.105	40	0.041												
IL10	Non-smokers	rs1800872(g.4433A>C,-592C>A)	A vs C							3	424/959	1.08(0.41-2.84)	0.874	88	0.000						
IL10	Non-smokers	rs1800871(c.-854T>C,-819C>T)	T vs C	3	506/909	1.22(0.54-2.77)	0.630	95	0.000	3	506/909	0.90(0.37-2.22)	0.823	88	0.000	3	506/909	1.76(0.51-6.12)	0.373	95	0.000

Genes	Sub-group	Variants	Comparison[a]	Results of meta-analysis using allelic model						Results of meta-analysis using dominant model						Results of meta-analysis using recessive model					
				Studies	Cases/Controls	OR(95% CI)	*P* value	I^2(%)	P_Q[b]	Studies	Cases/Controls	OR(95% CI)	*P* value	I^2(%)	P_Q[b]	Studies	Cases/Controls	OR(95% CI)	*P* value	I^2(%)	P_Q[b]
IL1B	Non-smokers	rs1143627(g.4970C>T,-31T>C)	C vs T	3	509/1568	1.20(0.91-1.59)	0.204	70	0.036	4	523/1827	1.27(0.93-1.75)	0.139	43	0.151	3	509/1568	1.15(0.82-1.62)	0.423	34	0.218
IL6	Non-smokers	rs1800796(-572C>G,634C>G)	G vs C	5	1234/2035	0.88(0.66-1.16)	0.360	75	0.003	3	1234/2035	0.88(0.62-1.26)	0.499	78	0.001	5	1234/2035	0.78(0.56-1.09)	0.145	0	0.722
MDM2	Non-smokers	rs2279744(SNP309,c.14+309T>G)	G vs T	6	1642/3277	1.13(0.94-1.35)	0.188	66	0.012	6	1642/3277	1.22(0.95-1.58)	0.124	61	0.025	6	1642/3277	1.17(0.92-1.48)	0.200	39	0.146
MIR146A	Non-smokers	rs2910164(C>G)	C vs G													3	538/717	1.29(0.99-1.67)	0.062	3	0.355
MIR196A2	Non-smokers	rs11614913(T>C)	C vs T	3	369/529	0.79(0.45-1.39)	0.407	84	0.002	3	369/529	1.28(0.93-1.75)	0.129	0	0.903	3	369/529	0.54(0.21-1.41)	0.208	85	0.001
MMP1	Non-smokers	rs1799750(-16071G/2G,insG)	2G vs 1G													3	304/665	0.95(0.70-1.28)	0.718	0	0.774
MPO	Non-smokers	rs2333227(G-463A)	A vs G	4	384/848	0.84(0.63-1.12)	0.233	0	0.986	4	384/848	0.83(0.61-1.15)	0.265	0	0.980	5	495/1001	1.14(0.70-1.86)	0.591	0	0.868
MTHFR	Non-smokers	rs1801131(p.Ala429Glu,c.1286A>C,A1298C)	C vs A													3	339/407	1.46(0.83-2.57)	0.187	0	0.594
MTHFR	Non-smokers	rs1801133(p.Ala222Val,Ex4+79C＞T)	T vs C	3	251/353	1.05(0.81-1.35)	0.730	0	0.843	3	251/353	1.08(0.77-1.50)	0.664	0	0.635	4	404/562	1.19(0.79-1.81)	0.412	0	0.884
NAT2	Non-smokers	Rapid/slow (Acetylation Phenotype)	Slow vs rapid	7	673/1108	1.12(0.72-1.74)	0.603	70	0.003												
NQO1	Non-smokers	rs1800566(p.Pro187Ser,c.648C>T,609C>T,*2)	T vs C	3	243/563	1.05(0.63-1.76)	0.850	67	0.049	9	461/1293	1.08(0.84-1.39)	0.562	4	0.399	3	243/563	1.03(0.35-3.01)	0.957	33	0.224
OGG1	Non-smokers	rs1052133(p.Ser326Cys,c.977C>G,8055C>G)	G vs C	10	1966/2674	1.14(1.04-1.24)	0.007	0	0.785	11	2068/2809	1.19(1.02-1.39)	0.025	0	0.867	10	1966/2674	1.13(0.93-1.38)	0.232	35	0.129
PADPRP	Non-smokers	A/B(a 193-bp deletion)	Del vs non-del							3	23/90	0.80(0.23-2.72)	0.717	0	0.517						
PTGS2	Non-smokers	rs5275(c.*427T>C,8473T>C)	C vs T							4	604/1846	1.01(0.68-1.49)	0.969	65	0.037						
SOD2	Non-smokers	rs4880(rs1799725,Ala16Val)	T vs C													4	271/765	1.00(0.60-1.68)	0.993	48	0.125
SULT1A1	Non-smokers	rs9282861(p.Arg213His,c.638G>A,*1/*2)	A vs G							4	370/675	1.35(1.00-1.83)	0.051	0	0.609						
TP53	Non-smokers	rs1042522(p.Pro72Arg,c.215C>G)	C vs G	9	1526/2415	0.93(0.73-1.18)	0.538	75	0.000	11	1717/2780	0.87(0.66-1.14)	0.316	63	0.002	11	1882/2887	1.28(1.01-1.61)	0.040	39	0.088
XRCC1	Non-smokers	rs25489(p.Arg280His,c.839G>A,Ex9+16G>A)	A vs G							5	666/1266	0.68(0.34-1.37)	0.279	81	0.000						
XRCC1	Non-smokers	rs1799782(p.Arg194Trp,c.580C>T,Ex6-22C＞T)	T vs C							4	552/1313	1.16(0.92-1.48)	0.218	0	0.624	3	395/468	1.39(0.48-4.05)	0.547	42	0.180
XRCC1	Non-smokers	rs25487(p.Gln399Arg,c.1196A>G)	A vs G	7	823/1644	1.01(0.76-1.34)	0.956	72	0.002	10	956/1923	1.04(0.74-1.46)	0.839	69	0.001	7	823/1644	1.04(0.63-1.70)	0.892	57	0.031
XRCC1	Non-smokers	rs3213245(-77T>C)	C vs T							3	977/1310	1.43(1.17-1.75)	4.56×10^{-4}	0	0.530						
XRCC3	Non-smokers	rs861539(p.Thr241Met,c.722C>T)	T vs C							3	180/281	0.87(0.42-1.82)	0.713	48	0.145						

Del=deletion.

[a] Allelic contrast or phenotype trait for common variants; genetic comparison for rare variants or variants only with genotype group data

[b] *P*-value of heterogeneity between studies

Supplementary Table S8. Credibility assessment for variants with significant associations with lung cancer risk in subgroup meta-analyses by smoking status

Genes	Sub-group	Variants	Alleles[a]	Number evaluated		Lung cancer risk meta-analysis					Venice criteria grades[d]	Credibility of evidence[e]	Amount of evidence[f]		Replication[g]	Protection from bias[h]			Sensitivity analysis[i]			Low OR[j]	Modified P^k	Excess P^l	Begg's P^m
				Studies	Cases/Controls	Genetic models	OR(95% CI)[b]	P value	I^2 (%)	P_Q[c]			Numbers	Grade		Grade	Reasons for bias	Reason for bias exemption	Initial study influence: OR(95%CI)	Initial study influence: P value	Deviation from HWE				
APEX1	Smokers	rs1760944	A/C	3	655/647	Allelic	1.37(1.11-1.69)	0.003	43	0.174	ABA	Moderate	1391	A	B	A			1.50(1.25-1.80)	9.90×10^{-6}	None	No	0.215	1.000	1.000
CHRNA5	Smokers	rs16969968	A/G	4	2828/3738	Allelic	1.30(1.20-1.42)	2.77×10^{-10}	0	0.626	AAC	Weak	3925	A	A	C	Small study		1.29(1.19-1.41)	1.27×10^{-9}	None	No	0.093	0.317	0.174
CYP1A1	Smokers	rs4646903	C/T	7	1034/1087	Allelic	1.30(1.02-1.64)	0.033	46	0.088	BBA	Moderate	749	B	B	A			1.35(1.01-1.81)	0.043	NO	No	0.145	0.355	0.230
CYP1A1	Smokers	rs1048943	G/A	14	1888/2413	Dominant	1.42(1.04-1.93)	0.028	78	0	ACC	Weak	1493	A	C	C	First study,HWE,small study, excess of significant studies		1.38(1.00-1.92)	0.052	Yes	No	0.060	0.098	0.189
CYP1B1	Smokers	rs1800440	G/A	3	447/449	Dominant	0.70(0.51-0.95)	0.024	0	0.481	BAC	Weak	252	B	A	C	First study		0.82(0.53-1.28)	0.388	None	No	0.364	0.523	1.000
CYP1B1	Smokers	rs1056836	G/C	5	622/684	Dominant	1.28(1.01-1.62)	0.045	0	0.616	BAC	Weak	773	B	A	C	First study		1.28(0.99-1.66)	0.064	NO	No	0.881	1.000	0.806
CYP2A6	Smokers	rs5031016	C/T	3	1527/1138	Allelic	0.57(0.33-1.00)	0.048	73	0.025	BCC	Weak	465	B	C	C	First study		0.46(0.17-1.24)	0.125	NO	No	0.587	1.000	0.296
CYP2A6	Smokers	non*4/*4	*4/non*4	3	1339/848	Allelic	0.71(0.59-0.85)	2.30×10^{-4}	13	0.319	BAA	Moderate	809	B	A	A			0.59(0.43-0.80)	0.001	NO	No	0.150	0.253	1.000
CYP2E1	Smokers	rs6413432	A/T	3	796/791	Allelic	0.75(0.63-0.90)	0.002	2	0.36	BAA	Moderate	691	B	A	A			0.74(0.62-0.88)	0.001	None	No	0.433	1.000	0.296
CYP2E1	Smokers	rs2031920	T/C	3	1064/1220	Allelic	0.76(0.65-0.90)	0.001	0	0.727	BAA	Moderate	770	B	A	A			0.78(0.66-0.92)	0.003	None	No	0.638	1.000	0.296
EPHX1	Smokers	rs2234922	G/A	5	909/845	Recessive	1.42(1.02-1.97)	0.039	0	0.535	BAC	Weak	195	B	A	C	First study		1.41(0.99-1.99)	0.055	NO	No	0.835	0.455	0.806
GSTM1	Smokers	Present/Null	NA	45	9276/9005	Null vs present	1.20(1.09-1.32)	2.16×10^{-4}	51	0	ACC	Weak	9092	A	C	C	Small study		1.19(1.08-1.31)	4.79×10^{-4}	None	No	0.070	0.354	0.304
GSTP1	Smokers	rs1138272	T/C	3	924/1026	Dominant	1.63(1.28-2.08)	9.17×10^{-5}	0	0.459	BAA	Moderate	315	B	A	A			1.64(1.01-2.68)	0.046	None	No	0.600	1.000	1.000
GSTP1	Smokers	rs1695	G/A	8	2830/2178	Allelic	1.29(1.09-1.53)	0.003	59	0.017	ACA	Weak	3118	A	C	A			1.25(1.05-1.50)	0.015	NO	No	0.298	0.474	0.536
MTHFR	Smokers	rs1801131	C/A	3	1245/1383	Allelic	1.14(1.01-1.27)	0.031	0	0.871	AAC	Weak	1728	A	A	C	First study,low OR		1.09(0.89-1.34)	0.423	NO	Yes	0.773	0.519	1.000
NAT2	Smokers	rs1799930	A/G	3	428/476	Allelic	0.81(0.66-0.99)	0.044	0	0.453	BAC	Weak	514	B	A	C	Small study		0.76(0.61-0.96)	0.020	None	No	0.067	0.460	0.296
NBN	Smokers	rs1805794	G/C	3	1226/1220	Recessive	0.83(0.71-0.98)	0.03	0	0.554	BAA	Moderate	930	B	A	A			0.81(0.66-0.98)	0.030	None	No	0.240	0.519	0.296
SOD2	Smokers	rs4880	T/C	3	1444/1188	Allelic	1.25(1.06-1.48)	0.007	33	0.224	ABC	Weak	2650	A	B	C	First study		1.60(0.76-3.39)	0.215	NO	No	0.263	1.000	0.296
TP53	Smokers	rs1042522	C/G	9	2747/2426	Dominant	1.23(1.01-1.50)	0.035	57	0.017	ACC	Weak	2971	A	C	C	First study, excess of significant studies		1.21(0.98-1.49)	0.084	NO	No	0.884	0.016	0.677
XRCC1	Smokers	rs25489	A/G	3	724/762	Allelic	1.35(1.00-1.81)	0.048	0	0.589	BAC	Weak	192	B	A	C	First study,HWE,small study		1.50(0.99-2.27)	0.054	Yes	No	0.012	1.000	0.296
XRCC1	Smokers	rs1799782	T/C	6	1597/1987	Allelic	0.73(0.55-0.97)	0.028	40	0.137	BBC	Weak	445	B	B	C	First study,HWE		0.75(0.54-1.04)	0.087	Yes	No	0.410	0.321	1.000
XRCC1	Smokers	rs25487	A/G	12	4087/3678	Recessive	0.79(0.63-1.00)	0.05	41	0.068	BBC	Weak	885	B	B	C	First study,HWE		0.80(0.61-1.04)	0.100	Yes	No	0.345	1.000	0.451
ATM	Non-smokers	rs189037	A/G	3	1323/1550	Allelic	1.18(1.03-1.36)	0.018	42	0.178	ABC	Weak	2565	A	B	C	First study,small study		1.15(0.93-1.43)	0.203	None	No	0.077	0.576	1.000
CYP1A1	Non-smokers	rs1048943	G/A	4	446/561	Recessive	1.93(1.03-3.63)	0.042	32	0.218	CBC	Weak	68	C	B	C	First study		2.08(0.94-4.60)	0.069	None	No	0.684	0.199	0.734
CYP2E1	Non-smokers	rs6413432	A/T	5	315/560	Dominant	0.72(0.54-0.97)	0.028	0	0.959	BAA	Moderate	378	B	A	A			0.72(0.53-0.96)	0.026	NO	No	0.837	0.489	0.806
CYP2E1	Non-smokers	rs2031920	T/C	3	304/695	Allelic	0.70(0.54-0.90)	0.005	0	0.863	BAA	Moderate	375	B	A	A			0.70(0.54-0.90)	0.005	None	No	0.145	1.000	1.000
ERCC1	Non-smokers	rs11615	C/T	3	731/958	Allelic	0.85(0.72-0.99)	0.042	0	0.449	AAA	Strong	2550	A	A	A			0.78(0.62-0.98)	0.031	None	No	0.898	0.483	1.000
ERCC2	Non-smokers	rs13181	C/A	3	478/469	Dominant	1.88(1.36-2.58)	1.11×10^{-4}	0	0.55	BAA	Moderate	249	B	A	A			1.91(1.25-2.92)	0.003	None	No	0.295	0.251	0.296
GSTM1	Non-smokers	Present/Null	NA	32	1924/4718	Null vs present	1.37(1.16-1.61)	1.60×10^{-4}	41	0.009	ABA	Moderate	3345	A	B	A			1.40(1.18-1.65)	1.18×10^{-4}	None	No	0.408	0.116	0.212
OGG1	Non-smokers	rs1052133	G/C	10	1966/2674	Allelic	1.14(1.04-1.24)	0.007	0	0.785	AAC	Weak	4670	A	A	C	Low OR,publication bias		1.15(1.04-1.26)	0.004	NO	Yes	0.169	1.000	0.074
TP53	Non-smokers	rs1042522	C/G	11	1882/2887	Recessive	1.28(1.01-1.61)	0.04	39	0.088	ABA	Moderate	1006	A	B	A			1.29(1.00-1.65)	0.049	NO	No	0.355	0.215	0.586
XRCC1	Non-smokers	rs3213245	C/T	3	977/1310	Dominant	1.43(1.17-1.75)	4.56×10^{-4}	0	0.53	BAA	Moderate	506	B	A	A			1.39(1.09-1.77)	0.009	None	No	0.107	0.618	0.296

NA=Not Applicable.

[a] Minor alleles/major alleles (per Caucasian)

[b] Summary ORs were calculated based on the DerSimonian-Lairs random-effects model

[c] *P*-value of heterogeneity between studies

[d] Venice criteria grades contained assessments of three aspects: the amount of evidence, extent of replication, and protection from bias, and each assessment was assigned three levels (A, B, or C).

[e] Credibility of evidence was evaluated based on Venice criteria with three aspects (the amount of evidence, extent of replication, and protection from bias), and was categorized as strong (all three aspect grades were A), moderate (any grade was B, but not C) or weak (any grade was C).

[f] Amount of evidence, depending on total sample size of the smallest genetic group among cases and controls in each meta-analysis, was graded as A (sample size>1000), B (sample size between 100 and 1000), or C (sample size <100).

[g] Extent of replication, depending on between-study heterogeneity, was graded as A (I^2<25%), B (I^2 between 25% and 50%), or C (I^2>50%)

[h] Protection from bias, considering various potential sources of bias in meta-analysis, was graded as A when there was no demonstrable bias and bias was unlikely invalidate the association, was graded as B when there was insufficient information for identifying evidence (i.e. missing information for evaluating Hardy-Weinberg equilibrium (HWE) among controls in an individual study) although there was no obvious bias, and C when the bias was evident and/or was likely to explain the presence of association. For the C grade, it was assigned if a meta-analysis had any of the following potential sources of bias: (1) magnitude of the association was low; (2) sensitivity analysis altered the statistical significance; (3) potential small study effect; (4) possible existence of an excess of significant findings (the number of observed significant studies more than the number of expected significant studies); (5) potential publication bias.

Small study=Possible existence of potential small study effect (modified Egger's test, *p*<0.10); First study=Significance lost excluding the first published or first positive report; HWE=Significance lost excluding studies with controls violating HWE; Excess of significant studies=Possible existence of an excess of significant findings (excess significance test, *p*<0.10); Low OR=Low magnitude of the association (i.e. 0.87<OR<1.15); Publication bias= Possible existence of potential publication bias (Begg's test, *p*<0.10).

[i] A sensitivity analysis was performed to examine whether the significant summary ORs were robust after excluding the first published or first positive report (defined as the earliest study reported a significant association), or excluding studies with controls violating HWE. None=A meta-analysis didn't include studies with controls violating HWE or evaluated association for a variant with a phenotype change; NO=Without the influence from studies with controls violating HWE; Yes=Evidence of influence from studies with controls violating HWE.

[j] Low OR (i.e. 0.87<OR<1.15; No=Association without a low OR;Yes=Association with a low OR)

[k] *P* value for potential small study effect based on the modified Egger's test. (*P*<0.10 is usually considered evidence for significant evidence of small study effect.)

[l] *P* value for possible existing an excess of significant findings based on the excess significance test. (*P*<0.10 is usually considered evidence for significant evidence of an excess of significant findings.)

[m] *P* value for potential publication bias of studies based on the Begg's test. (*P*<0.10 is usually considered evidence for significant evidence of publication bias.)

Supplementary Table S9. Credibility assessment for variants with non-significant associations with lung cancer risk in meta-analyses for all available data (Continued on next page)

Gene	Variant	Ethnicity	Comparison[a]	Number evaluated		Lung cancer risk meta-analysis					Begg P	Evaluation of the evidence[d]			Grade of evidence[e]	Credibility of evidence[f]
				Studies	Cases/Controls	OR(95% CI)[b]	*P* value	Q	P_Q[c]	I^2(%)		Power	Replication	Bias		
ERCC1	rs16979802(15310G>C)	Caucasian	C vs G	3	1311/2114	0.87(0.74-1.03)	0.102	0.87	0.647	0	1.000	A	A	A	AAA	Strong
ERCC1	rs2298881(262G>T,C>A)	All	T vs G	4	3049/3791	0.99(0.91-1.07)	0.723	3.50	0.320	14	0.308	A	A	A	AAA	Strong
ERCC1	rs735482(p.Lys261Thr,A>C)	All	C vs A	5	1682/2797	1.02(0.91-1.15)	0.709	4.34	0.362	8	0.462	A	A	A	AAA	Strong
POLI	rs3730668(-78G>T)	All	T vs G	3	672/823	0.94(0.80-1.09)	0.397	0.63	0.731	0	1.000	A	A	A	AAA	Strong
PPARG	rs1801282(p.Pro12Ala,c.36C>G)	All	G vs C	4	2476/3377	1.00(0.88-1.12)	0.956	0.82	0.844	0	0.734	A	A	A	AAA	Strong
PTGS2	rs20417(-765G>C)	All	C vs G	4	2355/2731	0.96(0.86-1.08)	0.505	1.84	0.606	0	1.000	A	A	A	AAA	Strong
TNF	rs1799724(-857C>T,-1036C>T)	All	C vs T	3	2080/2164	0.98(0.86-1.11)	0.727	1.80	0.406	0	1.000	A	A	A	AAA	Strong
ERCC2	rs1799793(p.Asp312Asn,c.981G>A)	All	A vs G	26	10209/12967	1.02(0.95-1.09)	0.637	43.81	0.011	43	0.628	A	B	A	ABA	Moderate
TYMS	28-bp tandem repeat	All	2R vs 3R (2R vs Non-2R)	4	2052/2861	0.99(0.90-1.08)	0.742	1.79	0.618	0	1.000	A	A	B	AAB	Moderate
XPC	rs2228000(p.Ala499Val,Ex9-377C>T)	All	T vs C	6	2981/3537	1.05(0.97-1.14)	0.205	5.17	0.395	3	1.000	B	A	A	BAA	Moderate
XRCC3	rs861539(p.Thr241Met,c.722C>T)	All	T vs C	18	6407/8356	1.04(0.95-1.13)	0.414	27.73	0.048	39	0.705	A	B	A	ABA	Moderate
ABCB1	rs1045642(p.Ile1145Ile,c.3435T>C)	All	T vs C	4	1025/1080	1.30(0.79-2.16)	0.307	28.98	0.000	90	0.308	C	C	A	CCA	Weak
ACE	287-bp repeat(I/D)	All	Ins vs del	8	1730/8359	1.01(0.85-1.21)	0.885	19.95	0.006	65	0.536	A	C	C	ACC	Weak
AGER	rs1800625(-429T>C,Alu1)	Asian	C vs T	3	1656/1693	1.16(0.78-1.74)	0.468	20.99	0.000	91	1.000	C	C	A	CCA	Weak
AGER	rs2070600(p.Gly82Ser,c.244G>A)	All	A vs G	4	2104/2178	1.10(0.93-1.31)	0.270	7.15	0.067	58	0.089	C	C	C	CCC	Weak
AHR	rs2066853(Arg554Lys,Ex10+501G>A)	All	A vs G	5	1749/1925	0.96(0.84-1.10)	0.567	5.82	0.213	31	1.000	C	B	A	CBA	Weak
ALDH2	rs671(p.Glu487Lys/Glu504Lys,c.1510G>A)	All	A vs G	3	1555/2079	1.36(0.74-2.50)	0.322	29.62	0.000	93	1.000	C	C	A	CCA	Weak
APEX1	rs1048945(p.Gln51His,c.153G>C)	Caucasian	C vs G	3	745/816	0.93(0.67-1.31)	0.684	0.30	0.859	0	1.000	C	A	A	CAA	Weak
APEX1	rs2307486(p.Ile64Val,c.190A>G)	All	G vs A	4	2281/2505	0.82(0.63-1.06)	0.121	6.35	0.096	53	0.089	C	C	C	CCC	Weak
ATM	rs609429(IVS48+238C>G)	All	(GG+GC) vs CC	3	1490/1501	0.82(0.66-1.03)	0.086	3.02	0.221	34	1.000	C	B	A	CBA	Weak
ATM	rs664143(IVS34G+60A)	All	(GG+GA) vs AA	3	1465/1465	0.93(0.79-1.10)	0.392	1.61	0.447	0	0.296	C	A	C	CAC	Weak
AURKA	rs2273535(p.Phe31Ile,c.91T>A)	Caucasian	A vs T	3	2301/2069	0.96(0.86-1.06)	0.421	0.63	0.730	0	0.296	C	A	A	CAA	Weak
AURKA	rs1047972(p.Ile57Val,c.169A>G)	Caucasian	A vs G	3	2463/2283	0.96(0.86-1.07)	0.425	1.65	0.437	0	0.296	C	A	A	CAA	Weak
BRCA2	rs144848(p.Asn372His,c.1114A>C)	All	C vs A	4	1586/2513	1.00(0.82-1.21)	0.959	6.90	0.075	57	0.734	C	C	A	CCA	Weak
CASC8	rs1447295(C>A)	All	A vs C	3	1541/2057	1.00(0.88-1.14)	0.991	1.52	0.469	0	1.000	C	A	A	CAA	Weak
CASP5	rs523104(p.Leu318Val,c.748C>G)	All	C vs G	3	758/943	1.12(0.98-1.29)	0.105	1.18	0.554	0	1.000	C	A	A	CAA	Weak
CASP8	rs3834129(-6526Ndel,-/CTTACT)	All	Del vs non-del	4	2737/2696	0.95(0.77-1.17)	0.631	16.45	0.001	82	0.308	C	C	A	CCA	Weak
CASP8	rs1045485(p.Asp302His,c.853G>C)	All	C vs G	4	1054/1259	1.20(0.97-1.49)	0.101	4.02	0.260	25	1.000	C	B	A	CBA	Weak
CCND1	rs678653(G>C)	All	C vs G	3	1323/1901	1.03(0.85-1.24)	0.773	5.03	0.081	60	1.000	C	C	A	CCA	Weak
CCND1	rs9344(rs603965,p.Pro241Pro,c.723G>A)	All	A vs G	11	6058/7315	1.02(0.92-1.12)	0.696	30.90	0.001	68	0.533	C	C	C	CCC	Weak
CDKN1A	rs1801270(p.Ser31Arg,c.93C>A,6829C>A)	All	A vs C	9	4039/4804	1.04(0.89-1.21)	0.663	16.82	0.032	52	0.029	C	C	C	CCC	Weak
CDKN2A	rs3731249(p.Ala148Thr,c.442G>A)	Caucasian	(AA+AG) vs GG	3	2875/2707	1.38(0.81-2.35)	0.230	4.56	0.102	56	0.296	C	C	A	CCA	Weak
CHRNA3	rs3743073(A>C)	All	G vs T	3	1391/1500	1.27(0.89-1.83)	0.191	23.05	0.000	91	0.296	A	C	C	ACC	Weak
COMT	rs4680(p.Val158Met,c.472G>A)	All	A vs G	5	1497/1958	1.09(0.84-1.43)	0.517	21.84	0.000	82	0.806	B	C	C	BCC	Weak
CRP	rs2808630(T>C)	All	C vs T	3	826/895	0.82(0.68-1.01)	0.056	2.79	0.248	28	1.000	C	B	A	CBA	Weak
CTLA4	rs3087243(g.6230G>A,CT60)	All	A vs G	3	1132/1220	0.99(0.86-1.14)	0.887	1.05	0.591	0	0.296	C	A	A	CAA	Weak
CTLA4	rs231775(p.Thr17Ala,c.49A>G)	All	G vs A	6	2828/2953	0.88(0.64-1.21)	0.445	63.12	0.000	92	0.707	C	C	C	CCC	Weak
CTLA4	rs5742909(g.319C>T,-318C>T)	All	T vs C	4	1205/1349	0.68(0.30-1.56)	0.364	48.66	0.000	94	0.308	C	C	A	CCA	Weak
CXCL12	rs1801157(801G>A)	All	A vs G	3	489/815	1.29(0.76-2.18)	0.351	17.39	0.000	89	1.000	B	C	A	BCA	Weak
CYP17A1	rs743572(T-34C,A1/A2)	All	A2 vs A1 (G vs A)	4	1245/1689	1.03(0.89-1.19)	0.701	4.80	0.187	38	0.308	C	B	A	CBA	Weak
CYP1A1	rs1799814(m4,Thr461Asn,C>A)	All	A vs C	5	1712/1967	1.22(0.57-2.62)	0.617	51.87	0.000	92	0.806	B	C	A	BCA	Weak
CYP1A1	rs1800031(m3,T5639C)	All	(CC+CT) vs TT	3	500/919	1.32(0.83-2.11)	0.248	0.63	0.730	0	0.296	C	A	B	CAB	Weak
CYP1A1	rs2606345(IVS1+606T>G)	Caucasian	G vs T	3	2770/3609	1.00(0.91-1.11)	0.937	3.05	0.217	35	1.000	C	B	A	CBA	Weak
CYP1A2	rs2069514(CYP1A2*1C,-3860G>A)	All	A vs G	5	925/1383	1.55(0.64-3.74)	0.327	32.99	0.000	88	0.221	A	C	A	ACA	Weak
CYP1A2	rs2069526(-739T>G)	All	G vs T	3	396/494	1.70(0.77-3.76)	0.189	11.15	0.004	82	1.000	A	C	A	ACA	Weak
CYP1A2	rs35694136(-2467delT)	All	Carriers vs non-carriers	4	811/1254	1.16(0.67-2.02)	0.594	11.94	0.008	75	0.734	A	C	C	ACC	Weak
CYP1B1	rs1056827(p.Ala119Ser,c.355G>T,m2)	All	T vs G	5	1501/2245	1.16(0.90-1.50)	0.253	18.67	0.001	79	1.000	C	C	A	CCA	Weak
CYP2A13	rs8192789(p.Arg257Cys,3375C>T)	All	(TT+TC) vs CC	3	1517/2231	0.81(0.57-1.15)	0.238	3.54	0.171	43	1.000	C	B	B	CBB	Weak
CYP2C9	rs1799853(p.Arg144Cys,c.430C>T)	All	(TT+TC) vs CC	4	756/1293	1.14(0.85-1.55)	0.381	4.57	0.206	34	1.000	C	B	A	CBA	Weak
CYP2D6	Phenotype (EM/HEM/PM)	All	PM vs EM	9	1627/1970	0.80(0.53-1.22)	0.296	9.90	0.272	19	0.348	C	A	A	CAA	Weak
CYP2D6	rs1065852(p.Pro34Ser,188C>T)	All	C vs T	3	581/636	0.86(0.57-1.30)	0.463	9.94	0.007	80	0.296	C	C	C	CCC	Weak
CYP2E1	rs3813867(g.3739G>C,c.-1293G>C)	All	C vs G	4	893/1029	0.94(0.56-1.56)	0.803	8.50	0.037	65	0.734	C	C	C	CCC	Weak
CYP3A4	rs2740574(–392A>G,*1A/*1B)	All	G vs A	4	1283/956	1.20(0.73-1.97)	0.465	9.14	0.028	67	1.000	A	C	C	ACC	Weak
CYP3A5	rs776746(A6986G,CYP3A5*3,22892A>G)	All	A vs G	4	1166/929	0.65(0.36-1.16)	0.144	25.41	0.000	88	0.308	A	C	A	ACA	Weak
DRD2	rs1079597(G>A,TaqI-)	All	A vs G	3	393/509	1.09(0.86-1.38)	0.499	1.53	0.465	0	0.296	C	A	A	CAA	Weak
DRD2	rs1800497(p.Glu714Lys,A2/A1,C>T)	All	C vs T	3	451/607	1.05(0.85-1.29)	0.667	0.48	0.786	0	1.000	C	A	A	CAA	Weak
EPHX1	rs1051740(p.Tyr113His,c.337T>C)	All	C vs T	21	3461/7217	1.05(0.89-1.24)	0.564	106.98	0.000	81	0.057	C	C	C	CCC	Weak
ERCC1	rs3212948(C>G)	All	G vs C	5	2144/2544	1.04(0.89-1.22)	0.591	10.24	0.037	61	0.462	A	C	A	ACA	Weak
ERCC1	rs3212955(A>G)	All	G vs A	4	2271/2845	1.08(0.98-1.18)	0.109	2.42	0.490	0	0.734	C	A	A	CAA	Weak
ERCC1	rs3212961(IVS5+33C＞A,17677C>A)	All	A vs C	6	2207/2311	0.94(0.86-1.03)	0.167	4.90	0.428	0	1.000	C	A	A	CAA	Weak
ERCC1	rs3212986(8092C>A,14443C>A)	All	A vs C	5	4079/4475	1.00(0.94-1.08)	0.931	1.63	0.804	0	0.806	C	A	A	CAA	Weak
ERCC2	rs1052555(Asp711Asp,G>A)	All	A vs G	3	511/751	1.09(0.85-1.40)	0.493	2.95	0.228	32	0.296	C	B	A	CBA	Weak
ERCC2	rs171140(A>C)	All	C vs A	3	511/751	1.04(0.87-1.26)	0.645	0.61	0.736	0	1.000	C	A	A	CAA	Weak
ERCC2	rs1799787(IVS19-70C＞T,G>A)	All	T vs C	6	1792/2807	1.03(0.83-1.27)	0.824	10.74	0.057	54	1.000	A	C	A	ACA	Weak
ERCC4	rs1799801(p.Ser835Ser,Ex11-247T＞C)	All	C vs T	3	1009/1758	1.02(0.88-1.17)	0.835	2.16	0.339	8	1.000	C	A	A	CAA	Weak
ERCC4	rs1800067(Arg415Gln)	Caucasian	A vs G	5	1521/2420	1.10(0.92-1.32)	0.286	1.19	0.880	0	0.806	C	A	A	CAA	Weak
ERCC5	rs17655(p.Asp1104His,c.3310G>C)	All	C vs G	10	3528/4481	0.96(0.84-1.10)	0.525	29.83	0.000	70	1.000	A	C	C	ACC	Weak
ERCC6	rs2228528(p.Gly399Asp,G1275A)	All	T vs C	4	1856/2059	1.05(0.95-1.15)	0.365	2.91	0.406	0	0.734	C	A	A	CAA	Weak
EXO1	rs1047840(p.Glu589Lys,c.1765G>A)	All	A vs G	3	1114/1166	1.23(0.96-1.57)	0.099	6.15	0.046	68	1.000	C	C	A	CCA	Weak
FANCG	rs2237857(p.Thr297Ile,C1382T)	All	T vs C	3	1553/1774	0.98(0.68-1.41)	0.903	4.52	0.104	56	1.000	C	C	A	CCA	Weak
GPC5	rs2352028(C>T)	Asian	T vs C	3	1648/2002	0.97(0.87-1.09)	0.611	1.43	0.490	0	1.000	C	A	A	CAA	Weak
GPX1	rs1050450(p.Pro200Leu,c.599C>T)	Caucasian	T vs C	4	976/1339	1.04(0.75-1.44)	0.811	16.02	0.001	81	0.734	C	C	A	CCA	Weak
GSTM3	rs1799735(c.468+21delAGG,3bp deletion,*A/*B)	All	Del vs non-del	8	1749/2069	0.97(0.86-1.09)	0.608	3.43	0.842	0	0.902	C	A	A	CAA	Weak
HIF1A	rs11549465(p.Pro582Ser,c.1744C>T)	All	T vs C	3	509/566	1.19(0.50-2.86)	0.690	18.09	0.000	89	1.000	A	C	A	ACA	Weak
IFNG	rs2069705(-1615T>C)	All	C vs T	3	2119/2654	1.02(0.93-1.11)	0.727	2.01	0.366	0	1.000	C	A	A	CAA	Weak
IGFBP3	rs2854744(C-202A)	Asian	C vs A	3	789/960	0.79(0.52-1.20)	0.262	13.99	0.001	86	0.296	C	C	A	CCA	Weak
IL10	rs1800871(c.-854T>C,-819C>T)	All	T vs C	11	3269/4627	0.93(0.76-1.14)	0.488	37.41	0.000	73	0.640	A	C	C	ACC	Weak
IL13	rs1800925(-1069C>T,-1112C>T)	All	T vs C	3	1788/1920	1.02(0.91-1.13)	0.785	0.91	0.635	0	0.296	C	A	A	CAA	Weak
IL13	rs20541(Arg130Gln,+2044G>A)	All	A vs G	3	1877/2020	1.01(0.90-1.12)	0.921	0.93	0.628	0	1.000	C	A	A	CAA	Weak
IL1B	rs1143634(p.Phe105Phe,Ex5+14C>T)	All	T vs C	5	4626/4116	1.05(0.95-1.17)	0.317	5.76	0.218	31	0.462	C	B	A	CBA	Weak
IL1B	rs16944(C-511T)	All	T vs C	11	4866/5910	0.94(0.84-1.06)	0.332	36.62	0.000	73	1.000	C	C	A	CCA	Weak
IL4	rs2243250(-509C>T)	All	T vs C	5	3496/4037	1.09(0.92-1.31)	0.320	14.46	0.006	72	0.462	A	C	A	ACA	Weak
IL6	rs1800795(G-174C)	All	C vs G	10	4619/5490	1.08(0.94-1.24)	0.287	28.73	0.001	69	0.858	C	C	A	CCA	Weak
IL8	rs4073(T-251A)	All	T vs A	7	4787/5310	1.02(0.90-1.15)	0.764	18.80	0.005	68	1.000	C	C	A	CCA	Weak
LIG1	rs20579(-7C>T)	All	A vs G	5	1818/3139	1.12(0.84-1.51)	0.440	23.17	0.000	83	0.806	C	C	C	CCC	Weak
LIG1	rs20581(p.Asp802Asp,G>A)	All	A vs G	4	828/1386	1.02(0.90-1.17)	0.726	0.96	0.810	0	0.734	C	A	A	CAA	Weak
LIG1	rs3730931(IVS9-21A>G)	All	G vs A	4	1399/2364	1.05(0.80-1.38)	0.715	9.55	0.023	69	0.308	C	C	A	CCA	Weak
LIG1	rs439132(A>G)	All	G vs A	3	739/1220	1.21(0.74-1.96)	0.444	6.66	0.036	70	1.000	A	C	C	ACC	Weak
LIG1	rs20580(p.Ala170Ala,c.417C>A)	All	A vs C	6	2357/3592	0.92(0.77-1.10)	0.370	24.88	0.000	80	0.133	C	C	A	CCA	Weak
LIG4	rs1805388(p.Thr9Ile,Ex2+54C>T)	All	T vs C	3	515/1246	1.07(0.77-1.50)	0.676	4.84	1.499	59	1.000	C	C	A	CCA	Weak
LTA	rs909253(A+252G)	All	G vs A	7	941/1683	0.93(0.83-1.05)	0.263	4.55	0.603	0	0.035	C	A	C	CAC	Weak
MBD4	rs140693(p.Glu346Lys,G1212A)	Asian	A vs G	4	2295/2469	0.99(0.86-1.13)	0.881	7.04	0.071	57	1.000	C	C	A	CCA	Weak
MCP1	rs1024611(-2518A>G,PvuII)	All	G vs A	3	1985/1981	0.76(0.53-1.09)	0.134	13.04	0.001	85	1.000	C	C	A	CCA	Weak
MDM2	rs117039649(G>C,SNP285)	All	C vs G	3	1846/4400	1.10(0.89-1.37)	0.374	0.51	0.773	0	1.000	C	A	A	CAA	Weak
MDM2	rs769412(p.Glu184Glu,2164A>G,SNP354)	All	G vs A	4	1691/1915	1.02(0.91-1.14)	0.785	1.58	0.663	0	0.734	C	A	A	CAA	Weak
MGMT	rs12268840(C>T)	Caucasian	T vs C	3	247/355	0.88(0.64-1.19)	0.396	2.64	0.267	24	1.000	C	A	A	CAA	Weak
MGMT	rs12917(p.Leu115Phe,c.343C>T)	All	T vs C	7	2552/3836	1.05(0.95-1.17)	0.335	5.87	0.438	0	0.548	C	A	A	CAA	Weak
MGMT	rs1803965(Leu53Leu,171C>T,TaqI)	All	T vs C	3	1060/1149	0.96(0.80-1.15)	0.671	0.75	0.686	0	0.296	C	A	A	CAA	Weak
MGMT	rs2308321(p.Ile174Val,Ile143Val,c.520A>G)	All	G vs A	6	2023/2191	1.05(0.86-1.28)	0.649	7.97	0.158	37	0.452	C	B	A	CBA	Weak
MGMT	rs2308327(p.Lys178Arg,c.533A>G)	Caucasian	G vs A	5	759/880	0.73(0.50-1.07)	0.109	12.48	0.014	68	0.086	C	C	C	CCC	Weak
MLH1	rs1799977(p.Ile219Val,c.655A>G)	All	G vs A	5	2147/2355	0.92(0.72-1.18)	0.531	7.91	0.095	49	0.806	C	B	A	CBA	Weak
MLH3	rs175080(p.Pro844Leu,C2645T)	All	T vs C	3	1363/1540	1.03(0.79-1.33)	0.837	5.85	0.054	66	0.296	C	C	A	CCA	Weak
MMP1	rs1799750(-16071G/2G,insG)	All	2G vs 1G	10	4787/4446	1.08(0.96-1.22)	0.197	27.39	0.001	67	0.474	C	C	A	CCA	Weak
MMP3	rs3025058(-16125A/6A,-11715A/6A)	All	5A vs 6A	5	3043/2422	1.08(0.93-1.27)	0.322	8.27	0.082	52	0.027	C	C	C	CCC	Weak
MMP9	rs3918242(g.3430C>T,-1562C>T)	All	T vs C	3	1205/1099	0.89(0.69-1.14)	0.357	3.40	0.182	41	1.000	C	B	C	CBC	Weak
MSH3	rs26279(p.Ala1045Thr,c.3133G>A)	All	G vs A	4	1649/1849	0.90(0.75-1.08)	0.251	6.83	0.077	56	0.734	C	C	A	CCA	Weak
MSH6	rs1042821(p.Gly39Glu,c.116G>A)	All	A vs G	3	1454/1620	0.95(0.83-1.08)	0.415	2.30	0.317	13	0.296	C	A	A	CAA	Weak
MTHFR	rs1801133(p.Ala222Val,Ex4+79C＞T)	All	T vs C	16	8692/8412	1.10(0.98-1.24)	0.125	69.01	0.000	78	0.192	A	C	C	ACC	Weak
MTR	rs1805087(Asp919Gly,A2756G)	All	G vs A	4	2488/3335	1.06(0.93-1.22)	0.387	4.61	0.202	35	1.000	C	B	A	CBA	Weak
MUTYH	rs3219489(p.Gln324His,c.972G>C)	All	C vs G	4	1250/1137	1.10(0.86-1.39)	0.455	8.17	0.043	63	1.000	C	C	A	CCA	Weak
MYCL	L/S(EcoRI)	All	S vs L	9	636/1004	1.05(0.91-1.22)	0.530	3.99	0.858	0	1.000	C	A	A	CAA	Weak
NAT2	Rapid/slow(Acetylation Phenotype)	All	Slow vs rapid	17	3747/4852	1.06(0.90-1.25)	0.502	38.19	0.001	58	0.064	C	C	C	CCC	Weak
NAT2	rs1799929(p.Leu161Leu,481C>T)	All	T vs C	3	576/686	1.11(0.87-1.41)	0.407	4.14	0.126	52	1.000	C	C	A	CCA	Weak
NFKB1	rs28362491(-94ins/delATTG)	All	Ins vs del	3	935/935	1.02(0.72-1.43)	0.919	11.51	0.003	83	0.296	C	C	C	CCC	Weak

Gene	Variant	Ethnicity	Comparison[a]	Number evaluated		Lung cancer risk meta-analysis					Begg P	Evaluation of the evidence[d]			Grade of evidence[e]	Credibility of evidence[f]
				Studies	Cases/Controls	OR(95% CI)[b]	*P* value	Q	P_Q[c]	I^2(%)		Power	Replication	Bias		
PADPRP	A/B(a 193-bp deletion)	All	Del vs non-del	3	288/292	1.13(0.70-1.82)	0.617	6.42	0.040	69	0.296	C	C	A	CCA	Weak
PARP1	rs1136410(p.Val762Ala,Ex17+8T＞C)	All	C vs T	6	3192/2874	1.01(0.89-1.15)	0.842	12.64	0.027	60	1.000	C	C	A	CCA	Weak
PCNA	rs25406(2232C>T)	All	T vs C	4	970/1302	0.91(0.77-1.06)	0.231	4.99	0.172	40	0.734	C	B	A	CBA	Weak
POLD1	rs1726801(p.Arg119His,c.356G>A)	Asian	A vs G	3	1263/1420	1.11(0.96-1.28)	0.168	1.61	0.448	0	0.296	C	A	C	CAC	Weak
POLI	rs8305(Thr706Ala,2180A>G)	All	G vs A	4	1655/2593	1.09(0.98-1.20)	0.111	2.17	0.537	0	0.734	C	A	A	CAA	Weak
POLL	rs3730477(p.Arg438Trp,1683C>T)	All	T vs C	3	1356/1542	1.29(0.69-2.40)	0.425	4.07	0.131	51	1.000	C	C	A	CCA	Weak
PPP1R13L	rs6966(T>A)	All	T vs A	5	1120/1361	1.09(0.92-1.29)	0.302	6.96	0.138	43	0.806	C	B	A	CBA	Weak
PPP1R13L	rs1970764(IVS1-4364A>G)	All	G vs A	3	678/726	1.12(0.90-1.39)	0.313	3.24	0.198	38	1.000	C	B	A	CBA	Weak
PTGS2	rs5275(c.*427T>C,8473T>C)	All	C vs T	11	6813/7988	0.95(0.83-1.09)	0.493	58.16	0.000	83	0.755	C	C	C	CCC	Weak
RAD23B	rs1805329(p.Ala249Val,Ex7+65C＞T)	All	T vs C	4	653/878	1.10(0.80-1.51)	0.572	9.39	0.025	68	0.089	C	C	C	CCC	Weak
REV1	rs3087386(p.Phe257Ser,892T>C)	All	C vs T	3	1437/1818	1.03(0.92-1.14)	0.658	0.51	0.775	0	1.000	C	A	A	CAA	Weak
SULT1A1	rs9282861(p.Arg213His,c.638G>A,*1/*2)	All	A vs G	6	1965/2286	1.20(0.97-1.48)	0.094	18.08	0.003	72	1.000	C	C	A	CCA	Weak
TDG	rs4135113(p.Gly199Ser,c.595G>A)	All	A vs G	3	289/318	0.85(0.51-1.42)	0.531	1.45	0.485	0	1.000	C	A	A	CAA	Weak
TGFB1	rs1800469(C-509T)	Asian	T vs C	4	1091/1238	0.95(0.85-1.07)	0.428	3.10	0.377	3	0.089	C	A	C	CAC	Weak
TGFB1	rs1800470(p.Leu10Pro,c.29C>T)	All	C vs T	7	1398/1553	1.16(0.97-1.39)	0.104	15.61	0.016	62	0.764	C	C	A	CCA	Weak
TNF	rs361525(A-417G,-238G>A)	All	A vs G	6	1047/1099	1.07(0.55-2.08)	0.847	33.99	0.000	85	0.133	C	C	C	CCC	Weak
TNFRSF10A	rs2230229(p.Arg441Lys,c.1322G>A)	All	G vs A	5	1479/1932	1.06(0.80-1.39)	0.702	11.74	0.019	66	0.806	C	C	A	CCA	Weak
TP53	rs1625895(IVS6+62G>A,A2>A1)	All	A vs G	8	2666/2847	1.10(0.90-1.34)	0.351	17.49	0.014	60	0.386	C	C	A	CCA	Weak
TP53	rs1642785(IVS2+38G>C,1827G>C)	All	C vs G	5	534/621	1.05(0.88-1.25)	0.614	3.00	0.559	0	0.086	C	A	C	CAC	Weak
TP53	rs17878362(IVS316bpDel/Ins)	Caucasian	Ins vs del	4	4196/4327	1.13(0.96-1.34)	0.152	8.22	0.042	64	0.734	C	C	A	CCA	Weak
TP53BP1	rs560191(p.Asp353Glu,c.1059C>G）	All	G vs C	3	1393/1373	0.94(0.69-1.30)	0.723	15.68	0.000	87	1.000	C	C	A	CCA	Weak
TP73	G4C14/A4T14	All	AT vs GC	7	3054/3311	1.04(0.88-1.24)	0.647	23.42	0.001	74	0.024	C	C	C	CCC	Weak
UGT1A6	rs1105879(p.Arg184Ser,c.552A>C)	All	G vs T	3	253/250	3.03(0.79-11.62)	0.106	36.84	0.000	95	0.296	A	C	A	ACA	Weak
VEGFA	rs2010963(634G>C)	All	C vs G	5	2989/2522	1.12(0.99-1.27)	0.083	7.23	0.124	45	0.806	C	B	A	CBA	Weak
VEGFA	rs3025039(C936T)	All	T vs C	6	2887/2506	1.01(0.87-1.17)	0.917	7.10	0.213	30	1.000	C	B	A	CBA	Weak
VEGFA	rs699947(C-2578A)	All	A vs C	4	879/804	0.97(0.81-1.15)	0.712	3.60	0.308	17	0.308	C	A	A	CAA	Weak
vWF	rs9295740(G>A)	All	A vs G	3	2221/2334	1.02(0.92-1.13)	0.685	0.59	0.744	0	0.296	C	A	A	CAA	Weak
XRCC1	rs2030404(A>G)	All	G vs A	3	514/751	0.98(0.83-1.17)	0.836	2.05	0.359	2	1.000	C	A	A	CAA	Weak
XRCC1	rs25486(A>G)	All	G vs A	3	507/795	0.99(0.74-1.34)	0.955	5.17	0.076	61	1.000	C	C	A	CCA	Weak
XRCC1	rs2682562(A>G)	All	G vs A	3	513/751	1.00(0.79-1.25)	0.974	0.70	0.705	0	1.000	C	A	A	CAA	Weak
XRCC1	rs2682585(p.His6Arg,c.17A>G)	All	A vs G	3	514/749	1.09(0.88-1.35)	0.416	1.91	0.385	0	1.000	C	A	A	CAA	Weak
XRCC1	rs2854510(A>G)	All	G vs A	3	513/751	0.97(0.79-1.20)	0.777	0.48	0.785	0	0.296	C	A	A	CAA	Weak
XRCC1	rs3213403(A>G)	All	G vs A	3	511/751	0.98(0.72-1.33)	0.880	2.19	0.335	9	1.000	C	A	A	CAA	Weak
XRCC1	rs3547(p.Gln632Gln,c.1896G>A)	All	A vs G	5	881/1201	1.00(0.86-1.17)	0.991	1.17	0.884	0	0.806	C	A	A	CAA	Weak
XRCC2	rs3218536(p.Arg188His,c.563G>A)	Caucasian	A vs G	3	645/1373	1.23(0.54-2.77)	0.625	16.79	0.000	88	1.000	A	C	C	ACC	Weak
XRCC3	rs1799796(17893A>G,IVS5-14)	All	G vs A	5	1478/3040	1.02(0.92-1.12)	0.756	3.45	0.486	0	0.806	C	A	A	CAA	Weak

Ins=insertion; Del=deletion; NA=Not Applicable.

[a] Allelic contrast (minor alleles vs major alleles) used in meta-analysis

[b] Summary ORs were calculated based on the DerSimonian-Lairs random-effects model

[c] *P*-value of heterogeneity between studies based on the Q statistic across crudes OR calculated for each enrolled study

[d] Evaluation of the evidence for the non-significant associations based on three aspects: statistical power, the degree of heterogeneity across studies, and potential bias assessment. Assessment of each aspect was assigned three levels (A, B, or C).

Statistical power, calculated by using SNP tools (see Methods in manuscript), was graded as A (Power≥90%), B (Power between 80% and 90%), or C (Power<80%). Replication, depending on the degree of heterogeneity across studies, was graded as A (I^2<25%), B (I^2 between 25% and 50%), or C (I^2>50%). Bias, considering various potential sources of bias in meta-analysis, was graded as A when there was no demonstrable bias and bias was unlikely alter the direction of summary ORs of non-significant associations, was graded as B when there was insufficient information for identifying evidence (i.e. missing information for evaluating Hardy-Weinberg equilibrium (HWE) among controls in an individual study) although there was no obvious bias, and graded as C when the bias was evident and/or was likely to explain the presence of non-significant association. For the C grade of assessment for potential bias, it was assigned if a meta-analysis had any of the following potential sources of bias: (1) the association became statistical significant after excluding studies that showed HWE deviation in controls; (2) potential small study effect (modified Egger's test, *p*<0.10); (3) possible existence of an excess of significant findings (excess significance test, *p*<0.10); (4) potential publication bias (Begg's test, p<0.10).

[e] Grade of evidence based on three aspects, including the evaluations of statistical power, replication of evidence, and potential bias.

[f] Credibility of evidence for non-significant association based on the ratings of evidence with three aspects, and was categorized as strong (all three aspect grades were A), moderate (any grade was B, but not C) or weak (any grade was C).

Supplementary literatures

Lists of 1,018 eligible studies enrolled in this comprehensive research synopsis (pp 77-133)

1. Zupa A, *et al.* GSTM1 and NAT2 polymorphisms and colon, lung and bladder cancer risk: a case-control study. *Anticancer Res* **29**, 1709-1714 (2009).
2. Zu Y, *et al.* Genetic variation in a miR-335 binding site in BIRC5 alters susceptibility to lung cancer in Chinese Han populations. *Biochem Biophys Res Commun* **430**, 529-534 (2013).
3. Zienolddiny S, *et al.* The TERT-CLPTM1L lung cancer susceptibility variant associates with higher DNA adduct formation in the lung. *Carcinogenesis* **30**, 1368-1371 (2009).
4. Zienolddiny S, Ryberg D, Maggini V, Skaug V, Canzian F, Haugen A. Polymorphisms of the interleukin-1 beta gene are associated with increased risk of non-small cell lung cancer. *Int J Cancer* **109**, 353-356 (2004).
5. Zienolddiny S, *et al.* A comprehensive analysis of phase I and phase II metabolism gene polymorphisms and risk of non-small cell lung cancer in smokers. *Carcinogenesis* **29**, 1164-1169 (2008).
6. Zienolddiny S, *et al.* Polymorphisms of DNA repair genes and risk of non-small cell lung cancer. *Carcinogenesis* **27**, 560-567 (2006).
7. Zhu Y, Spitz MR, Lei L, Mills GB, Wu X. A single nucleotide polymorphism in the matrix metalloproteinase-1 promoter enhances lung cancer susceptibility. *Cancer Res* **61**, 7825-7829 (2001).
8. Zhu X, Fan W, Li D. Intronic boundary mutation rs430397 cannot affect alternate splicing and is an indecisive risk factor for non-small cell lung cancer. *Chest* **142**, 1691-1692 (2012).
9. Zhou Y, *et al.* Functional haplotypes in the promoter of matrix metalloproteinase-2 and lung cancer susceptibility. *Carcinogenesis* **26**, 1117-1121 (2005).
10. Zhou W, *et al.* Gene-smoking interaction associations for the ERCC1 polymorphisms in the risk of lung cancer. *Cancer Epidemiol Biomarkers Prev* **14**, 491-496 (2005).
11. Zhou W, *et al.* Polymorphisms in the DNA repair genes XRCC1 and ERCC2, smoking, and lung cancer risk. *Cancer Epidemiol Biomarkers Prev* **12**, 359-365 (2003).
12. Zhou W, *et al.* Gene-environment interaction for the ERCC2 polymorphisms and cumulative cigarette smoking exposure in lung cancer. *Cancer Res* **62**, 1377-1381 (2002).
13. Zhou M, Wan HY, Gao BL, Ding YJ, Jun RX. Genetic polymorphisms of XPD and CDA and lung cancer risk. *Oncol Lett* **4**, 247-251 (2012).
14. Zhong S, *et al.* Glutathione S-transferase mu locus: use of genotyping and phenotyping assays to assess association with lung cancer susceptibility. *Carcinogenesis* **12**, 1533-1537 (1991).
15. Zhong R, *et al.* Genetic variations in TERT-CLPTM1L locus are associated with risk of lung cancer in Chinese population. *Mol Carcinog* **52 Suppl 1**, E118-126 (2013).
16. Zheng Y, *et al.* GPC5 rs2352028 polymorphism and risk of lung cancer in Han Chinese. *Cancer Invest* **30**, 13-19 (2012).
17. Zheng X, *et al.* Functionally significant nicotine acetylcholine receptor subunit

alpha5 promoter haplotypes are associated with susceptibility to lung cancer in Chinese. *Cancer* **117**, 4714-4723 (2011).
18. Zheng MF, Ji Y, Wu XB, Ye SG, Chen JY. EphB4 gene polymorphism and protein expression in non-small-cell lung cancer. *Mol Med Rep* **6**, 405-408 (2012).
19. Zheng J, *et al.* Functional genetic variations in the IL-23 receptor gene are associated with risk of breast, lung and nasopharyngeal cancer in Chinese populations. *Carcinogenesis* **33**, 2409-2416 (2012).
20. Zhao Z, *et al.* Significant association of 5p15.33 (TERT-CLPTM1L genes) with lung cancer in Chinese Han population. *Exp Lung Res* **39**, 91-98 (2013).
21. Zhao MM, *et al.* Genetic variations in TERT-CLPTM1L genes and risk of lung cancer in a Chinese population. *Asian Pac J Cancer Prev* **15**, 2809-2813 (2014).
22. Zhao H, Spitz MR, Gwyn KM, Wu X. Microsomal epoxide hydrolase polymorphisms and lung cancer risk in non-Hispanic whites. *Mol Carcinog* **33**, 99-104 (2002).
23. Zhao B, *et al.* Dietary isothiocyanates, glutathione S-transferase -M1, -T1 polymorphisms and lung cancer risk among Chinese women in Singapore. *Cancer Epidemiol Biomarkers Prev* **10**, 1063-1067 (2001).
24. Zhang Z, Yu D, Yuan J, Guo Y, Wang H, Zhang X. Cigarette smoking strongly modifies the association of complement factor H variant and the risk of lung cancer. *Cancer Epidemiol* **36**, e111-115 (2012).
25. Zhang Z, *et al.* Genetic variants in MUC4 gene are associated with lung cancer risk in a Chinese population. *PLoS One* **8**, e77723 (2013).
26. Zhang YH, *et al.* Polymorphism rs7214723 in CAMKK1 and lung cancer risk in Chinese population. *Tumour Biol* **34**, 3147-3152 (2013).
27. Zhang Y, *et al.* Genetic polymorphisms of TERT and CLPTM1L and risk of lung cancer: A case-control study in northeast Chinese male population. *Med Oncol* **31**, (2014).
28. Zhang Y, *et al.* Association between polymorphisms in COMT, PLCH1, and CYP17A1, and non-small-cell lung cancer risk in Chinese nonsmokers. *Clin Lung Cancer* **14**, 45-49 (2013).
29. Zhang Y, *et al.* Association between C3orf21, TP63 polymorphisms and environment and NSCLC in never-smoking Chinese population. *Gene* **497**, 93-97 (2012).
30. Zhang X, *et al.* Functional polymorphisms in cell death pathway genes FAS and FASL contribute to risk of lung cancer. *J Med Genet* **42**, 479-484 (2005).
31. Zhang X, *et al.* Polymorphisms in DNA base excision repair genes ADPRT and XRCC1 and risk of lung cancer. *Cancer Res* **65**, 722-726 (2005).
32. Zhang X, *et al.* Genetic polymorphisms in cell cycle regulatory genes MDM2 and TP53 are associated with susceptibility to lung cancer. *Hum Mutat* **27**, 110-117 (2006).
33. Zhang X, Li X, Wu Z, Lin F, Zhou H. The p73 G4C14-to-A4T14 polymorphism is associated with risk of lung cancer in the Han nationality of North China. *Mol Carcinog* **52**, 387-391 (2013).
34. Zhang X, *et al.* Polymorphisms on 8q24 are associated with lung cancer risk and survival in Han Chinese. *PLoS One* **7**, e41930 (2012).
35. Zhang W, Weissfeld JL, Romkes M, Land SR, Grandis JR, Siegfried JM. Association of the EGFR intron 1 CA repeat length with lung cancer risk. *Mol Carcinog* **46**, 372-380 (2007).
36. Zhang W, *et al.* Mutation and polymorphism in the EGFR-TK domain associated

with lung cancer. *J Thorac Oncol* **1**, 635-647 (2006).
37. Zhang S, *et al.* A variant in the CHEK2 promoter at a methylation site relieves transcriptional repression and confers reduced risk of lung cancer. *Carcinogenesis* **31**, 1251-1258 (2010).
38. Zhang S, *et al.* REV3L 3'UTR 460 T>C polymorphism in microRNA target sites contributes to lung cancer susceptibility. *Oncogene* **32**, 242-250 (2013).
39. Zhang M, *et al.* Candidate variants at 6p21.33 and 6p22.1 and risk of non-small cell lung cancer in a Chinese population. *Int J Mol Epidemiol Genet* **1**, 11-18 (2010).
40. Zhang J, *et al.* CYP19A1 gene polymorphisms and risk of lung cancer. *J Int Med Res* **41**, 735-742 (2013).
41. Zhang J, *et al.* The functional polymorphism in the matrix metalloproteinase-7 promoter increases susceptibility to esophageal squamous cell carcinoma, gastric cardiac adenocarcinoma and non-small cell lung carcinoma. *Carcinogenesis* **26**, 1748-1753 (2005).
42. Zhang HT, *et al.* Mutational analysis of the transforming growth factor beta receptor type I gene in primary non-small cell lung cancer. *Lung Cancer* **40**, 281-287 (2003).
43. Zhang H, *et al.* Common genetic variants in 53BP1 associated with nonsmall-cell lung cancer risk in Han Chinese. *Arch Med Res* **45**, 84-89 (2014).
44. Zhai R, *et al.* Vascular endothelial growth factor genotypes, haplotypes, gender, and the risk of non-small cell lung cancer. *Clin Cancer Res* **14**, 612-617 (2008).
45. Zejnilovic J, Akev N, Yilmaz H, Isbir T. Association between manganese superoxide dismutase polymorphism and risk of lung cancer. *Cancer Genet Cytogenet* **189**, 1-4 (2009).
46. Zambon L, Honma HN, Lourenco GJ, Saad IA, Mussi RK, Lima CS. A polymorphism in the angiogenesis inhibitor, endostatin, in lung cancer susceptibility. *Lung Cancer* **59**, 276-278 (2008).
47. Yu X, Rao J, Lin J, Zhang Z, Cao L, Zhang X. Tag SNPs in complement receptor-1 contribute to the susceptibility to non-small cell lung cancer. *Mol Cancer* **13**, 56 (2014).
48. Yu P, Liu YP, Zhang JD, Qu XJ, Jin B, Zhang Y. Correlation between PARP-1 Val762Ala polymorphism and the risk of lung cancer in a Chinese population. *Tumour Biol*, (2014).
49. Yu D, *et al.* Characterization of functional excision repair cross-complementation group 1 variants and their association with lung cancer risk and prognosis. *Clin Cancer Res* **14**, 2878-2886 (2008).
50. Yu C, *et al.* Correlation between a single nucleotide polymorphism in the matrix metalloproteinase-2 promoter and risk of lung cancer. *Cancer Res* **62**, 6430-6433 (2002).
51. Young RP, Hopkins RJ, Whittington CF, Hay BA, Epton MJ, Gamble GD. Individual and cumulative effects of GWAS susceptibility loci in lung cancer: associations after sub-phenotyping for COPD. *PLoS One* **6**, e16476 (2011).
52. Young RP, Hopkins RJ, Hay BA, Whittington CF, Epton MJ, Gamble GD. FAM13A locus in COPD is independently associated with lung cancer - Evidence of a molecular genetic link between COPD and lung cancer. *Appl Clin Genet* **4**, 1-10 (2011).
53. Young RP, Hopkins RJ, Hay BA, Gamble GD. GSTM1 null genotype in COPD and lung cancer: Evidence of a modifier or confounding effect? *Appl Clin Genet* **4**, 137-144 (2011).

54. Young RP, *et al.* A gene-based risk score for lung cancer susceptibility in smokers and ex-smokers. *Postgrad Med J* **85**, 515-524 (2009).
55. Young RP, Hopkins RJ, Hay BA, Epton MJ, Black PN, Gamble GD. Lung cancer gene associated with COPD: triple whammy or possible confounding effect? *Eur Respir J* **32**, 1158-1164 (2008).
56. You W, *et al.* No association between TGFBR1*6A and lung cancer. *J Thorac Oncol* **2**, 657-659 (2007).
57. Yoshida K, *et al.* Lung cancer susceptibility among atomic bomb survivors in relation to CA repeat number polymorphism of epidermal growth factor receptor gene and radiation dose. *Carcinogenesis* **30**, 2037-2041 (2009).
58. Yoon KA, *et al.* Genetic polymorphisms in the Rb-binding zinc finger gene RIZ and the risk of lung cancer. *Carcinogenesis* **28**, 1971-1977 (2007).
59. Yoon KA, Kim JH, Gil HJ, Hwang H, Hwangbo B, Lee JS. CYP1B1, CYP1A1, MPO, and GSTP1 polymorphisms and lung cancer risk in never-smoking Korean women. *Lung Cancer* **60**, 40-46 (2008).
60. Yoon KA, *et al.* Novel polymorphisms in the SUV39H2 histone methyltransferase and the risk of lung cancer. *Carcinogenesis* **27**, 2217-2222 (2006).
61. Yoon KA, Gil HJ, Han J, Park J, Lee JS. Genetic polymorphisms in the polycomb group gene EZH2 and the risk of lung cancer. *J Thorac Oncol* **5**, 10-16 (2010).
62. Yongjun Zhang M, Zhang A, Hua Shi M, Xiangming Kong M. Association between TGM5, PPAP2B and PSMA4 polymorphisms and NSCLC in never-smoking Chinese population. *J Cancer Res Ther* **9**, 660-663 (2013).
63. Yin ZH, Li MC, Cui ZS, He QC, Zhou BS. Relationship between ERCC2 polymorphism and risk of lung cancer in Chinese nonsmoker. *Chin J Cancer Res* **19**, 184-188 (2007).
64. Yin ZH, *et al.* TP63 gene polymorphisms, cooking oil fume exposure and risk of lung adenocarcinoma in Chinese non-smoking females. *Asian Pac J Cancer Prev* **14**, 6519-6522 (2014).
65. Yin Z, *et al.* ERCC2, ERCC1 polymorphisms and haplotypes, cooking oil fume and lung adenocarcinoma risk in Chinese non-smoking females. *J Exp Clin Cancer Res* **28**, 153 (2009).
66. Yin Z, *et al.* Genetic polymorphisms of TERT and CLPTM1L, cooking oil fume exposure, and risk of lung cancer: A case-control study in a Chinese non-smoking female population. *Med Oncol* **31**, (2014).
67. Yin L, Pu Y, Liu TY, Tung YH, Chen KW, Lin P. Genetic polymorphisms of NAD(P)H quinone oxidoreductase, CYP1A1 and microsomal epoxide hydrolase and lung cancer risk in Nanjing, China. *Lung Cancer* **33**, 133-141 (2001).
68. Yin JY, Han LF, Huang Q, Xu XJ, Zhou HH, Liu ZQ. ABCC1 polymorphism Arg723Gln (2168G> A) is associated with lung cancer susceptibility in a Chinese population. *Clin Exp Pharmacol Physiol* **38**, 632-637 (2011).
69. Yin J, *et al.* HapMap-based study identifies risk sub-region on chromosome 19q13.3 in relation to lung cancer among Chinese. *Cancer Epidemiol* **37**, 923-929 (2013).
70. Yin J, *et al.* Hapmap-based evaluation of ERCC2, PPP1R13L, and ERCC1 and lung cancer risk in a Chinese population. *Environ Mol Mutagen* **53**, 239-245 (2012).
71. Yin J, *et al.* A specific diplotype defined by PPP1R13L rs1970764, CD3EAP rs967591 and ERCC1 rs11615 and lung cancer risk in a Chinese population.

Lung Cancer **76**, 286-291 (2012).
72. Yin J, *et al.* HapMap-based study of a region encompassing ERCC1 and ERCC2 related to lung cancer susceptibility in a Chinese population. *Mutat Res* **713**, 1-7 (2011).
73. Yin J, Vogel U, Ma Y, Qi R, Wang H. HapMap-based study of the DNA repair gene ERCC2 and lung cancer susceptibility in a Chinese population. *Carcinogenesis* **30**, 1181-1185 (2009).
74. Yin J, Vogel U, Ma Y, Qi R, Wang H. Haplotypes of nine single nucleotide polymorphisms on chromosome 19q13.2-3 associated with susceptibility of lung cancer in a Chinese population. *Mutat Res* **641**, 12-18 (2008).
75. Yin J, Vogel U, Ma Y, Qi R, Sun Z, Wang H. A haplotype encompassing the variant allele of DNA repair gene polymorphism ERCC2/XPD Lys751Gln but not the variant allele of Asp312Asn is associated with risk of lung cancer in a northeastern Chinese population. *Cancer Genet Cytogenet* **175**, 47-51 (2007).
76. Yin J, Vogel U, Ma Y, Qi R, Sun Z, Wang H. The DNA repair gene XRCC1 and genetic susceptibility of lung cancer in a northeastern Chinese population. *Lung Cancer* **56**, 153-160 (2007).
77. Yin J, *et al.* Effects of PPP1R13L and CD3EAP variants on lung cancer susceptibility among nonsmoking Chinese women. *Gene* **524**, 228-231 (2013).
78. Yeh KT, Chen JC, Chen CM, Wang YF, Lee TP, Chang JG. CYP3A5*1 is an inhibitory factor for lung cancer in Taiwanese. *Kaohsiung J Med Sci* **19**, 201-207 (2003).
79. Yaren A, Oztop I, Turgut S, Turgut G, Degirmencioglu S, Demirpence M. Angiotensin-converting enzyme gene polymorphism is associated with anemia in non-small-cell lung cancer. *Exp Biol Med* **233**, 32-37 (2008).
80. Yang XR, *et al.* CYP1A1 and GSTM1 polymorphisms in relation to lung cancer risk in Chinese women. *Cancer Lett* **214**, 197-204 (2004).
81. Yang S, *et al.* Association of miR-502-binding site single nucleotide polymorphism in the 3'-untranslated region of SET8 and TP53 codon 72 polymorphism with non-small cell lung cancer in Chinese population. *Acta Biochim Biophys Sin* **46**, 149-154 (2014).
82. Yang R, *et al.* Functional genetic polymorphisms in PP2A subunit genes confer increased risks of lung cancer in southern and eastern Chinese. *PLoS One* **8**, e77285 (2013).
83. Yang P, *et al.* Alpha1-antitrypsin deficiency carriers, tobacco smoke, chronic obstructive pulmonary disease, and lung cancer risk. *Arch Intern Med* **168**, 1097-1103 (2008).
84. Yang P, *et al.* Alpha1-antitrypsin and neutrophil elastase imbalance and lung cancer risk. *Chest* **128**, 445-452 (2005).
85. Yang P, Bamlet WR, Ebbert JO, Taylor WR, de Andrade M. Glutathione pathway genes and lung cancer risk in young and old populations. *Carcinogenesis* **25**, 1935-1944 (2004).
86. Yang M, *et al.* Functional FEN1 polymorphisms are associated with DNA damage levels and lung cancer risk. *Hum Mutat* **30**, 1320-1328 (2009).
87. Yang M, Choi Y, Hwangbo B, Lee JS. Combined effects of genetic polymorphisms in six selected genes on lung cancer susceptibility. *Lung Cancer* **57**, 135-142 (2007).
88. Yang L, *et al.* Effects of a functional variant c.353T>C in snai1 on risk of two contextual diseases. Chronic obstructive pulmonary disease and lung cancer. *Am J Respir Crit Care Med* **189**, 139-148 (2014).

89. Yang L, Shi GL, Song CX, Xu SF. Relationship between genetic polymorphism of MCP-1 and non-small-cell lung cancer in the Han nationality of North China. *Genet Mol Res* **9**, 765-771 (2010).
90. Yang L, *et al.* Functional polymorphisms of CHRNA3 predict risks of chronic obstructive pulmonary disease and lung cancer in Chinese. *PLoS One* **7**, e46071 (2012).
91. Yang L, *et al.* A functional copy number variation in the WWOX gene is associated with lung cancer risk in Chinese. *Hum Mol Genet* **22**, 1886-1894 (2013).
92. Yang L, *et al.* A common genetic variant (97906C>A) of DAB2IP/AIP1 is associated with an increased risk and early onset of lung cancer in Chinese males. *PLoS One* **6**, e26944 (2011).
93. Yang L, *et al.* A functional polymorphism at microRNA-629-binding site in the 3'-untranslated region of NBS1 gene confers an increased risk of lung cancer in Southern and Eastern Chinese population. *Carcinogenesis* **33**, 338-347 (2012).
94. Yang J, Lan H, Huang X, Liu B, Tong Y. MicroRNA-126 inhibits tumor cell growth and its expression level correlates with poor survival in non-small cell lung cancer patients. *PLoS One* **7**, e42978 (2012).
95. Yang H, Spitz MR, Stewart DJ, Lu C, Gorlov IP, Wu X. ATM sequence variants associate with susceptibility to non-small cell lung cancer. *Int J Cancer* **121**, 2254-2259 (2007).
96. Yan F, Xu JF, Liu XF, Li XH. Interaction between smoking and CYP2C19*3 polymorphism increased risk of lung cancer in a Chinese population. *Tumour Biol* **35**, 5295-5298 (2014).
97. Yadav DS, *et al.* Polymorphisms of glutathione-S-transferase genes and the risk of aerodigestive tract cancers in the Northeast Indian population. *Genet Test Mol Biomarkers* **14**, 715-723 (2010).
98. Xue X, *et al.* The joint effect of hOGG1, APE1, and ADPRT polymorphisms and cooking oil fumes on the risk of lung adenocarcinoma in Chinese non-smoking females. *PLoS One* **8**, e71157 (2013).
99. Xu X, Kelsey KT, Wiencke JK, Wain JC, Christiani DC. Cytochrome P450 CYP1A1 MspI polymorphism and lung cancer susceptibility. *Cancer Epidemiol Biomarkers Prev* **5**, 687-692 (1996).
100. Xu P, *et al.* Genetic Variation in BCL2 3'-UTR Was Associated with Lung Cancer Risk and Prognosis in Male Chinese Population. *PLoS One* **8**, (2013).
101. Xu M, *et al.* Serum C-reactive protein and risk of lung cancer: a case-control study. *Med Oncol* **30**, 319 (2013).
102. Xu LL, *et al.* Counterpoint: the myeloperoxidase -463G-->a polymorphism does not decrease lung cancer susceptibility in Caucasians. *Cancer Epidemiol Biomarkers Prev* **11**, 1555-1559 (2002).
103. Xu HL, *et al.* Effects of polymorphisms in translesion DNA synthesis genes on lung cancer risk and prognosis in Chinese men. *Cancer Epidemiol* **37**, 917-922 (2013).
104. Xing D, Tan W, Wei Q, Lin D. Polymorphisms of the DNA repair gene XPD and risk of lung cancer in a Chinese population. *Lung Cancer* **38**, 123-129 (2002).
105. Xie C, *et al.* Sipa1 promoter polymorphism predicts risk and metastasis of lung cancer in Chinese. *Mol Carcinog* **52 Suppl 1**, E110-117 (2013).
106. Xiao Q, *et al.* 9q33.3, A stress-related chromosome region, contributes to reducing lung squamous cell carcinoma risk. *J Thorac Oncol* **9**, 1041-1047 (2014).

107. Xiao G, Zhang T, Yao J, Ren J, Cao W, Wu G. The association between RASSF1 gene polymorphisms and lung cancer susceptibility among people in Hubei Province of China. *J Huazhong Univ Sci Technolog Med Sci* **29**, 646-649 (2009).
108. Wu X, *et al.* XPA polymorphism associated with reduced lung cancer risk and a modulating effect on nucleotide excision repair capacity. *Carcinogenesis* **24**, 505-509 (2003).
109. Wu X, *et al.* p53 Genotypes and Haplotypes Associated With Lung Cancer Susceptibility and Ethnicity. *J Natl Cancer Inst* **94**, 681-690 (2002).
110. Wu X, *et al.* Associations between cytochrome P4502E1 genotype, mutagen sensitivity, cigarette smoking and susceptibility to lung cancer. *Carcinogenesis* **18**, 967-973 (1997).
111. Wu X, Hudmon KS, Detry MA, Chamberlain RM, Spitz MR. D2 dopamine receptor gene polymorphisms among African-Americans and Mexican-Americans: a lung cancer case-control study. *Cancer Epidemiol Biomarkers Prev* **9**, 1021-1026 (2000).
112. Wu X, Gwyn K, Amos CI, Makan N, Hong WK, Spitz MR. The association of microsomal epoxide hydrolase polymorphisms and lung cancer risk in African-Americans and Mexican-Americans. *Carcinogenesis* **22**, 923-928 (2001).
113. Wu X, *et al.* Cytochrome P450 2E1 DraI polymorphisms in lung cancer in minority populations. *Cancer Epidemiol Biomarkers Prev* **7**, 13-18 (1998).
114. Wu W, *et al.* Genetic variants in GTF2H1 and risk of lung cancer: a case-control analysis in a Chinese population. *Lung Cancer* **63**, 180-186 (2009).
115. Wu MF, *et al.* Increased expression of cytochrome P4501B1 in peripheral leukocytes from lung cancer patients. *Toxicol Lett* **150**, 211-219 (2004).
116. Wu KS, Zhou X, Zheng F, Xu XQ, Lin YH, Yang J. Influence of interleukin-1 beta genetic polymorphism, smoking and alcohol drinking on the risk of non-small cell lung cancer. *Clin Chim Acta* **411**, 1441-1446 (2010).
117. Wu H, *et al.* Association between the telomerase reverse transcriptase (TERT) rs2736098 polymorphism and cancer risk: evidence from a case-control study of non-small-cell lung cancer and a meta-analysis. *PLoS One* **8**, e76372 (2013).
118. Wu C, *et al.* Genetic variants on chromosome 15q25 associated with lung cancer risk in Chinese populations. *Cancer Res* **69**, 5065-5072 (2009).
119. Wright CM, *et al.* Genetic association study of CYP1A1 polymorphisms identifies risk haplotypes in nonsmall cell lung cancer. *Eur Respir J* **35**, 152-159 (2010).
120. Woodson K, *et al.* Effect of vitamin intervention on the relationship between GSTM1, smoking, and lung cancer risk among male smokers. *Cancer Epidemiol Biomarkers Prev* **8**, 965-970 (1999).
121. Wolf CR, *et al.* Relationship between the debrisoquine hydroxylase polymorphism and cancer susceptibility. *Carcinogenesis* **13**, 1035-1038 (1992).
122. Wojas-Krawczyk K, *et al.* CYP1A1, GSTT1, and GSTM1 polymorphisms and lung cancer in eastern Poland. *Adv Clin Exp Med* **18**, 461-471 (2009).
123. Wikman H, *et al.* Relevance of N-acetyltransferase 1 and 2 (NAT1, NAT2) genetic polymorphisms in non-small cell lung cancer susceptibility. *Pharmacogenetics* **11**, 157-168 (2001).
124. Wikman H, *et al.* hOGG1 polymorphism and loss of heterozygosity (LOH): significance for lung cancer susceptibility in a caucasian population. *Int J Cancer* **88**, 932-937 (2000).
125. Wiencke JK, Spitz MR, McMillan A, Kelsey KT. Lung cancer in

Mexican-Americans and African-Americans is associated with the wild-type genotype of the NAD(P)H: quinone oxidoreductase polymorphism. *Cancer Epidemiol Biomarkers Prev* **6**, 87-92 (1997).
126. Weston A*, et al.* Allelic frequency of a p53 polymorphism in human lung cancer. *Cancer Epidemiol Biomarkers Prev* **1**, 481-483 (1992).
127. Weston A*, et al.* Determination of the allelic frequencies of an L-myc and a p53 polymorphism in human lung cancer. *Carcinogenesis* **15**, 583-587 (1994).
128. Weston A*, et al.* Relationship of H-ras-1, L-myc, and p53 polymorphisms with lung cancer risk and prognosis. *Environ Health Perspect* **98**, 61-67 (1992).
129. Wei C*, et al.* A case-control study of a sex-specific association between a 15q25 variant and lung cancer risk. *Cancer Epidemiol Biomarkers Prev* **20**, 2603-2609 (2011).
130. Wauters E*, et al.* The TERT-CLPTM1L locus for lung cancer predisposes to bronchial obstruction and emphysema. *Eur Respir J* **38**, 924-931 (2011).
131. Watanabe J*, et al.* An Rsa I polymorphism in the CYP2E1 gene does not affect lung cancer risk in a Japanese population. *Jpn J Cancer Res* **86**, 245-248 (1995).
132. Watanabe J*, et al.* Association of CYP1B1 genetic polymorphism with incidence to breast and lung cancer. *Pharmacogenetics* **10**, 25-33 (2000).
133. Wassenaar CA, Dong Q, Wei Q, Amos CI, Spitz MR, Tyndale RF. Relationship between CYP2A6 and CHRNA5-CHRNA3-CHRNB4 variation and smoking behaviors and lung cancer risk. *J Natl Cancer Inst* **103**, 1342-1346 (2011).
134. Wassenaar CA, Dong Q, Amos CI, Spitz MR, Tyndale RF. Pilot study of CYP2B6 genetic variation to explore the contribution of nitrosamine activation to lung carcinogenesis. *Int J Mol Sci* **14**, 8381-8392 (2013).
135. Wang YC, Chen CY, Chen SK, Chang YY, Lin P. p53 codon 72 polymorphism in Taiwanese lung cancer patients: association with lung cancer susceptibility and prognosis. *Clin Cancer Res* **5**, 129-134 (1999).
136. Wang Y, Zhou F, Wu Y, Xu D, Li W, Liang S. The relationship between three heat shock protein 70 gene polymorphisms and susceptibility to lung cancer. *Clin Chem Lab Med* **48**, 1657-1663 (2010).
137. Wang Y, Zhang Y, Gu C, Bao W, Bao Y. Neuronal acetylcholine receptor subunit alpha-9 (CHRNA9) polymorphisms are associated with NSCLC risk in a Chinese population. *Med Oncol* **31**, 932 (2014).
138. Wang Y, Spitz MR, Tsou AM, Zhang K, Makan N, Wu X. Sulfotransferase (SULT) 1A1 polymorphism as a predisposition factor for lung cancer: a case-control analysis. *Lung Cancer* **35**, 137-142 (2002).
139. Wang Y, Spitz MR, Schabath MB, Ali-Osman F, Mata H, Wu X. Association between glutathione S-transferase p1 polymorphisms and lung cancer risk in Caucasians: a case-control study. *Lung Cancer* **40**, 25-32 (2003).
140. Wang Y*, et al.* XRCC3 genetic polymorphism, smoking, and lung carcinoma risk in minority populations. *Cancer* **98**, 1701-1706 (2003).
141. Wang Y*, et al.* No association between the C-1562T polymorphism in the promoter of matrix metalloproteinase-9 gene and non-small cell lung carcinoma. *Lung Cancer* **49**, 155-161 (2005).
142. Wang Y, Broderick P, Matakidou A, Vijayakrishnan J, Eisen T, Houlston RS. Variation in TP63 is associated with lung adenocarcinoma in the UK population. *Cancer Epidemiol Biomarkers Prev* **20**, 1453-1462 (2011).
143. Wang Y, Broderick P, Matakidou A, Eisen T, Houlston RS. Chromosome 15q25 (CHRNA3-CHRNA5) variation impacts indirectly on lung cancer risk. *PLoS One* **6**, e19085 (2011).

144. Wang Y, Broderick P, Matakidou A, Eisen T, Houlston RS. Role of 5p15.33 (TERT-CLPTM1L), 6p21.33 and 15q25.1 (CHRNA5-CHRNA3) variation and lung cancer risk in never-smokers. *Carcinogenesis* **31**, 234-238 (2010).
145. Wang X, *et al.* Cancer stem cell marker Musashi-1 rs2522137 genotype is associated with an increased risk of lung cancer. *PLoS One* **9**, e95915 (2014).
146. Wang X, *et al.* RAGE genetic polymorphisms are associated with risk, chemotherapy response and prognosis in patients with advanced NSCLC. *PLoS One* **7**, e43734 (2012).
147. Wang W, Spitz MR, Yang H, Lu C, Stewart DJ, Wu X. Genetic variants in cell cycle control pathway confer susceptibility to lung cancer. *Clin Cancer Res* **13**, 5974-5981 (2007).
148. Wang SL, Lee H, Chen KW, Tsai KJ, Chen CY, Lin P. Cytochrome P4502E1 genetic polymorphisms and lung cancer in a Taiwanese population. *Lung Cancer* **26**, 27-34 (1999).
149. Wang M, *et al.* Identification of a novel tumor suppressor gene p34 on human chromosome 6q25.1. *Cancer Res* **67**, 93-99 (2007).
150. Wang LI, *et al.* Manganese superoxide dismutase alanine-to-valine polymorphism at codon 16 and lung cancer risk. *J Natl Cancer Inst* **93**, 1818-1821 (2001).
151. Wang LI, Giovannucci EL, Hunter D, Neuberg D, Su L, Christiani DC. Dietary intake of Cruciferous vegetables, Glutathione S-transferase (GST) polymorphisms and lung cancer risk in a Caucasian population. *Cancer Causes Control* **15**, 977-985 (2004).
152. Wang LE, Cheng L, Spitz MR, Wei Q. Fas A670G polymorphism, apoptotic capacity in lymphocyte cultures, and risk of lung cancer. *Lung Cancer* **42**, 1-8 (2003).
153. Wang L, Lu J, An J, Shi Q, Spitz MR, Wei Q. Polymorphisms of cytosolic serine hydroxymethyltransferase and risk of lung cancer: a case-control analysis. *Lung Cancer* **57**, 143-151 (2007).
154. Wang L, Liu H, Zhang Z, Spitz MR, Wei Q. Association of genetic variants of O6-methylguanine-DNA methyltransferase with risk of lung cancer in non-Hispanic Whites. *Cancer Epidemiol Biomarkers Prev* **15**, 2364-2369 (2006).
155. Wang L, *et al.* The association of c.1471G>A genetic polymorphism in XRCC1 gene with lung cancer susceptibility in Chinese Han population. *Tumour Biol* **35**, 5389-5393 (2014).
156. Wang J, *et al.* Association of GSTM1, CYP1A1 and CYP2E1 genetic polymorphism with susceptibility to lung adenocarcinoma: A case-control study in Chinese population. *Cancer Sci* **94**, 448-452 (2003).
157. Wang J, Deng Y, Cheng J, Ding J, Tokudome S. GST genetic polymorphisms and lung adenocarcinoma susceptibility in a Chinese population. *Cancer Lett* **201**, 185-193 (2003).
158. Wang HT, *et al.* PARP-1 rs3219073 polymorphism may contribute to susceptibility to lung cancer. *Genet Test Mol Biomarkers* **18**, 1-5 (2014).
159. Wang H, *et al.* The genetic variant rs401681C/T is associated with the risk of non-small cell lung cancer in a Chinese mainland population. *Genet Mol Res* **12**, 67-73 (2013).
160. Wang H, *et al.* Genetic variant in the 3'-untranslated region of VEGFR1 gene influences chronic obstructive pulmonary disease and lung cancer development in Chinese population. *Mutagenesis* **29**, 311-317 (2014).
161. Wang H, *et al.* Substantial reduction in risk of lung adenocarcinoma associated

with genetic polymorphism in CYP2A13, the most active cytochrome P450 for the metabolic activation of tobacco-specific carcinogen NNK. *Cancer Res* **63**, 8057-8061 (2003).

162. Wang H, Li L, Ding L, Zhang Z, Pu C. Association of genetic polymorphisms in the paraoxonase 1 gene with the risk and prognosis of non-small cell lung cancer in Chinese Han population. *J Investig Med* **60**, 592-597 (2012).
163. Wang H, *et al.* Genetic susceptibility of lung cancer associated with common variants in the 3' untranslated regions of the adenosine triphosphate-binding cassette B1 (ABCB1) and ABCC1 candidate transporter genes for carcinogen export. *Cancer* **115**, 595-607 (2009).
164. Wang H, *et al.* Beta-2 adrenergic receptor gene (ADRB2) polymorphism and risk for lung adenocarcinoma: a case-control study in a Chinese population. *Cancer Lett* **240**, 297-305 (2006).
165. Wang F, *et al.* TERT rs2736100T/G polymorphism upregulates interleukin 6 expression in non-small cell lung cancer especially in adenocarcinoma. *Tumour Biol* **35**, 4667-4672 (2014).
166. Walsh KM, *et al.* Fine-mapping of the 5p15.33, 6p22.1-p21.31, and 15q25.1 regions identifies functional and histology-specific lung cancer susceptibility loci in African-Americans. *Cancer Epidemiol Biomarkers Prev* **22**, 251-260 (2013).
167. Walsh KM, *et al.* Association study of nicotinic acetylcholine receptor genes identifies a novel lung cancer susceptibility locus near CHRNA1 in African-Americans. *Oncotarget* **3**, 1428-1438 (2012).
168. Voho A, *et al.* EPHX1 gene polymorphisms and individual susceptibility to lung cancer. *Cancer Lett* **237**, 102-108 (2006).
169. Vogel U, Overvad K, Wallin H, Tjonneland A, Nexo BA, Raaschou-Nielsen O. Combinations of polymorphisms in XPD, XPC and XPA in relation to risk of lung cancer. *Cancer Lett* **222**, 67-74 (2005).
170. Vogel U, Nexo BA, Wallin H, Overvad K, Tjonneland A, Raaschou-Nielsen O. No association between base excision repair gene polymorphisms and risk of lung cancer. *Biochem Genet* **42**, 453-460 (2004).
171. Vogel U, *et al.* Two regions in chromosome 19q13.2-3 are associated with risk of lung cancer. *Mutat Res* **546**, 65-74 (2004).
172. Vogel U, *et al.* Polymorphisms in genes involved in the inflammatory response and interaction with NSAID use or smoking in relation to lung cancer risk in a prospective study. *Mutat Res* **639**, 89-100 (2008).
173. Vineis P, *et al.* Genetic susceptibility according to three metabolic pathways in cancers of the lung and bladder and in myeloid leukemias in nonsmokers. *Ann Oncol* **18**, 1230-1242 (2007).
174. Vinci S, *et al.* Genetic variants in miR-146a, miR-149, miR-196a2, miR-499 and their influence on relative expression in lung cancers. *Clin Chem Lab Med* **49**, 2073-2080 (2011).
175. van der Knaap R, Siemes C, Coebergh JW, van Duijn CM, Hofman A, Stricker BH. Renin-angiotensin system inhibitors, angiotensin I-converting enzyme gene insertion/deletion polymorphism, and cancer: the Rotterdam Study. *Cancer* **112**, 748-757 (2008).
176. Unsal M, Kara N, Karakus N, Tural S, Elbistan M. Effects of leptin and leptin receptor gene polymorphisms on lung cancer. *Tumour Biol* **35**, 10231-10236 (2014).
177. Ulybina YM, *et al.* Coding polymorphisms in Casp5, Casp8 and DR4 genes may play a role in predisposition to lung cancer. *Cancer Lett* **278**, 183-191 (2009).

178. Uematsu F, *et al.* Restriction fragment length polymorphism of the human CYP2E1 (cytochrome P450IIE1) gene and susceptibility to lung cancer: possible relevance to low smoking exposure. *Pharmacogenetics* **4**, 58-63 (1994).
179. Tsuneoka Y, Fukushima K, Matsuo Y, Ichikawa Y, Watanabe Y. Genotype analysis of the CYP2C19 gene in the Japanese population. *Life Sci* **59**, 1711-1715 (1996).
180. Tseng RC, Hsieh FJ, Shih CM, Hsu HS, Chen CY, Wang YC. Lung cancer susceptibility and prognosis associated with polymorphisms in the nonhomologous end-joining pathway genes: a multiple genotype-phenotype study. *Cancer* **115**, 2939-2948 (2009).
181. Tsai YY, *et al.* Genetic susceptibility and dietary patterns in lung cancer. *Lung Cancer* **41**, 269-281 (2003).
182. Tournel G, *et al.* Molecular analysis of the CYP2F1 gene: identification of a frequent non-functional allelic variant. *Mutat Res* **617**, 79-89 (2007).
183. Togo AV, *et al.* L-MYC polymorphism in cancer patients, healthy blood donors and elderly, tumor-free individuals in Russia. *Int J Cancer* **85**, 747-750 (2000).
184. To-Figueras J, *et al.* Polymorphism of glutathione S-transferase M3: Interaction with glutathione S-transferase MI and lung cancer susceptibility. *Biomarkers* **5**, 73-80 (2000).
185. To-Figueras J, Gene M, Gomez-Catalan J, Pique E, Borrego N, Corbella J. Lung cancer susceptibility in relation to combined polymorphisms of microsomal epoxide hydrolase and glutathione S-transferase P1. *Cancer Lett* **173**, 155-162 (2001).
186. To-Figueras J, *et al.* Genetic polymorphism of glutathione S-transferase P1 gene and lung cancer risk. *Cancer Causes Control* **10**, 65-70 (1999).
187. To-Figueras J, *et al.* Glutathione S-transferase M1 (GSTM1) and T1 (GSTT1) polymorphisms and lung cancer risk among Northwestern Mediterraneans. *Carcinogenesis* **18**, 1529-1533 (1997).
188. To-Figueras J, *et al.* Glutathione-S-Transferase M1 and codon 72 p53 polymorphisms in a northwestern Mediterranean population and their relation to lung cancer susceptibility. *Cancer Epidemiol Biomarkers Prev* **5**, 337-342 (1996).
189. Timofeeva MN, *et al.* Genetic polymorphisms in 15q25 and 19q13 loci, cotinine levels, and risk of lung cancer in EPIC. *Cancer Epidemiol Biomarkers Prev* **20**, 2250-2261 (2011).
190. Timofeeva MN, *et al.* CYP450 polymorphisms as risk factors for early-onset lung cancer: gender-specific differences. *Carcinogenesis* **30**, 1161-1169 (2009).
191. Timofeeva M, *et al.* Genetic polymorphisms of MPO, GSTT1, GSTM1, GSTP1, EPHX1 and NQO1 as risk factors of early-onset lung cancer. *Int J Cancer* **127**, 1547-1561 (2010).
192. Tilak AR, Kumar S, Pant MC, Mathur N, Kumar A. Polymorphism Arg72Pro of p53 confers susceptibility to squamous cell carcinoma of lungs in a North Indian population. *DNA Cell Biol* **32**, 66-72 (2013).
193. Tilak AR, *et al.* Association of functionally important polymorphism of microsomal epoxide hydrolase gene (EPHX1) with lung cancer susceptibility. *Cancer Invest* **29**, 411-418 (2011).
194. Tian T, *et al.* A functional genetic variant in microRNA-196a2 is associated with increased susceptibility of lung cancer in Chinese. *Cancer Epidemiol Biomarkers Prev* **18**, 1183-1187 (2009).
195. Tian F, Zhang Y, Ren Y, Shen L, Wu W, Zhou B. N-Acetyltransferase 2 (NAT2)

gene polymorphism and exposure to smoking in lung cancer of Chinese males. *Med Oncol* **31**, (2014).
196. Thanopoulou E*, et al.* The single nucleotide polymorphism g.1548A >G (K469E) of the ICAM-1 gene is associated with worse prognosis in non-small cell lung cancer. *Tumour Biol* **33**, 1429-1436 (2012).
197. Ter-Minassian M*, et al.* Apoptosis gene polymorphisms, age, smoking and the risk of non-small cell lung cancer. *Carcinogenesis* **29**, 2147-2152 (2008).
198. Tekpli X, Landvik NE, Skaug V, Gulsvik A, Haugen A, Zienolddiny S. Functional effect of polymorphisms in 15q25 locus on CHRNA5 mRNA, bulky DNA adducts and TP53 mutations. *Int J Cancer* **132**, 1811-1820 (2013).
199. Teixeira AL*, et al.* Influence of TGFB1+869T>C functional polymorphism in non-small cell lung cancer (NSCLC) risk. *J Cancer Res Clin Oncol* **137**, 435-439 (2011).
200. Tefre T*, et al.* Genotyping of the CYP2D6 gene in Norwegian lung cancer patients and controls. *Pharmacogenetics* **4**, 47-57 (1994).
201. Tefre T, Borresen AL, Aamdal S, Brogger A. Studies of the L-myc DNA polymorphism and relation to metastasis in Norwegian lung cancer patients. *Br J Cancer* **61**, 809-812 (1990).
202. Tastemir-Korkmaz D, Demirhan O, Kuleci S, Hasturk S. There is no significant association between death receptor 4 (DR4) gene polymorphisms and lung cancer in Turkish population. *Pathol Oncol Res* **19**, 779-784 (2013).
203. Taniguchi K*, et al.* Polymorphisms in the promoter region of the neutrophil elastase gene are associated with lung cancer development. *Clin Cancer Res* **8**, 1115-1120 (2002).
204. Tang J, Zhao J, Zhao J. The relationship between genetic variants of XRCC1 gene and lung cancer susceptibility in Chinese Han population. *Med Oncol* **31**, (2014).
205. Tang H*, et al.* LAPTM4B polymorphism is associated with non-small cell lung cancer susceptibility and prognosis. *Oncol Rep* **31**, 2454-2460 (2014).
206. Tang DL*, et al.* Associations between both genetic and environmental biomarkers and lung cancer: evidence of a greater risk of lung cancer in women smokers. *Carcinogenesis* **19**, 1949-1953 (1998).
207. Tanaka Y*, et al.* Nonsynonymous single nucleotide polymorphisms in DNA damage repair pathways and lung cancer risk. *Cancer* **116**, 896-902 (2010).
208. Tanaka D*, et al.* Polymorphism of dihydropyrimidine dehydrogenase (DPYD) Cys29Arg and risk of six malignancies in Japanese. *Nagoya J Med Sci* **67**, 117-124 (2005).
209. Tan XL, Moslehi R, Han W, Spivack SD. Haplotype-tagging single nucleotide polymorphisms in the GSTP1 gene promoter and susceptibility to lung cancer. *Cancer Detect Prev* **32**, 403-415 (2009).
210. Tan W, Chen GF, Xing DY, Song CY, Kadlubar FF, Lin DX. Frequency of CYP2A6 gene deletion and its relation to risk of lung and esophageal cancer in the Chinese population. *Int J Cancer* **95**, 96-101 (2001).
211. Tamaki Y*, et al.* Association between cancer risk and drug-metabolizing enzyme gene (CYP2A6, CYP2A13, CYP4B1, SULT1A1, GSTM1, and GSTT1) polymorphisms in cases of lung cancer in Japan. *Drug Metab Pharmacokinet* **26**, 516-522 (2011).
212. Tamai S*, et al.* Restriction fragment length polymorphism analysis of the L-myc gene locus in a case-control study of lung cancer. *Int J Cancer* **46**, 411-415 (1990).

213. Taioli E, Ford J, Trachman J, Li Y, Demopoulos R, Garte S. Lung cancer risk and CYP1A1 genotype in African Americans. *Carcinogenesis* **19**, 813-817 (1998).
214. Szymanowska A, *et al.* Increased risk of non-small cell lung cancer and frequency of somatic TP53 gene mutations in Pro72 carriers of TP53 Arg72Pro polymorphism. *Lung Cancer* **52**, 9-14 (2006).
215. Swartz MD, *et al.* Investigating multiple candidate genes and nutrients in the folate metabolism pathway to detect genetic and nutritional risk factors for lung cancer. *PLoS One* **8**, e53475 (2013).
216. Svartberg J, *et al.* Single-nucleotide polymorphism, rs1799941 in the Sex Hormone-Binding Globulin (SHBG) gene, related to both serum testosterone and SHBG levels and the risk of myocardial infarction, type 2 diabetes, cancer and mortality in men: The Tromso Study. *Andrology* **2**, 212-218 (2014).
217. Suzuki T, *et al.* Impact of one-carbon metabolism-related gene polymorphisms on risk of lung cancer in Japan: a case control study. *Carcinogenesis* **28**, 1718-1725 (2007).
218. Sung JS, Jin L, Jo U, Park KH, Kim YH. Association between -276 C/T polymorphism of the ERBB3 gene and lung cancer risk in a Korean population. *Anticancer Res* **32**, 4433-4437 (2012).
219. Sung JS, *et al.* Putative association of the single nucleotide polymorphisms in RASSF1A promoter with Korean lung cancer. *Lung Cancer* **61**, 301-308 (2008).
220. Sunaga N, *et al.* Contribution of the NQO1 and GSTT1 polymorphisms to lung adenocarcinoma susceptibility. *Cancer Epidemiol Biomarkers Prev* **11**, 730-738 (2002).
221. Sun Y, Zhang YJ, Kong XM. No association of XRCC1 and CLPTM1L polymorphisms with non-small cell lung cancer in a non-smoking Han Chinese population. *Asian Pac J Cancer Prev* **14**, 5171-5174 (2013).
222. Sun T, *et al.* Functional genetic variations in cytotoxic T-lymphocyte antigen 4 and susceptibility to multiple types of cancer. *Cancer Res* **68**, 7025-7034 (2008).
223. Sun T, *et al.* Haplotypes in matrix metalloproteinase gene cluster on chromosome 11q22 contribute to the risk of lung cancer development and progression. *Clin Cancer Res* **12**, 7009-7017 (2006).
224. Sun T, *et al.* A six-nucleotide insertion-deletion polymorphism in the CASP8 promoter is associated with susceptibility to multiple cancers. *Nat Genet* **39**, 605-613 (2007).
225. Sun SF, Huang DB, Cao C, Deng ZC. Polymorphism of VEGF-460C/T associated with the risk and clinical characteristics of lung cancer in Chinese population. *Med Oncol* **30**, 410 (2013).
226. Sun JZ, Yang XX, Hu NY, Li X, Li FX, Li M. Genetic variants in MMP9 and TCF2 contribute to susceptibility to lung cancer. *Chin J Cancer Res* **23**, 183-187 (2011).
227. Sun GF, Shimojo N, Pi JB, Lee S, Kumagai Y. Gene deficiency of glutathione S-transferase mu isoform associated with susceptibility to lung cancer in a Chinese population. *Cancer Lett* **113**, 169-172 (1997).
228. Sugimura H, *et al.* Association of Ile462Val (Exon 7) polymorphism of cytochrome P450 IA1 with lung cancer in the Asian population: further evidence from a case-control study in Okinawa. *Cancer Epidemiol Biomarkers Prev* **7**, 413-417 (1998).
229. Sugimura H, *et al.* Cytochrome P-450 IA1 genotype in lung cancer patients and controls in Rio de Janeiro, Brazil. *Cancer Epidemiol Biomarkers Prev* **3**, 145-148 (1994).

230. Sugimura H, *et al.* hOGG1 Ser326Cys polymorphism and lung cancer susceptibility. *Cancer Epidemiol Biomarkers Prev* **8**, 669-674 (1999).
231. Sugimura H, *et al.* CYP1A1 and CYP2E1 polymorphism and lung cancer, case-control study in Rio de Janeiro, Brazil. *Pharmacogenetics* **5 Spec No**, S145-148 (1995).
232. Su XL, Bin B, Cui HW, Ran MR. Cytochrome P450 2E1 RsaI/PstI and DraI polymorphisms are risk factors for lung cancer in Mongolian and Han population in inner Mongolia. *Chin J Cancer Res* **23**, 107-111 (2011).
233. Su L, *et al.* Genotypes and haplotypes of matrix metalloproteinase 1, 3 and 12 genes and the risk of lung cancer. *Carcinogenesis* **27**, 1024-1029 (2006).
234. Su L, *et al.* No association between the p21 codon 31 serine-arginine polymorphism and lung cancer risk. *Cancer Epidemiol Biomarkers Prev* **12**, 174-175 (2003).
235. Stucker I, *et al.* Genetic polymorphisms of glutathione S-transferases as modulators of lung cancer susceptibility. *Carcinogenesis* **23**, 1475-1481 (2002).
236. Stucker I, *et al.* CYP2D6 genotype and lung cancer risk according to histologic type and tobacco exposure. *Carcinogenesis* **16**, 2759-2764 (1995).
237. Strazisar M, Mlakar V, Rott T, Glavac D. Somatic alterations of the serine/threonine kinase LKB1 gene in squamous cell (SCC) and large cell (LCC) lung carcinoma. *Cancer Invest* **27**, 407-416 (2009).
238. Stankovic MM, *et al.* TNF-alpha-308 promotor polymorphism in patients with chronic obstructive pulmonary disease and lung cancer. *Neoplasma* **56**, 348-352 (2009).
239. Sreelekha TT, Rajesh M, Anil Kumar V, Madhavan J, Balaram P. CYP1A1m2 polymorphisms regulate estrogen and interleukin-6 in lung cancer. *Mol Med Report* **3**, 971-976 (2010).
240. Sreeja L, *et al.* Prognostic importance of DNA repair gene polymorphisms of XRCC1 Arg399Gln and XPD Lys751Gln in lung cancer patients from India. *J Cancer Res Clin Oncol* **134**, 645-652 (2008).
241. Sreeja L, Syamala V, Raveendran PB, Santhi S, Madhavan J, Ankathil R. p53 Arg72Pro polymorphism predicts survival outcome in lung cancer patients in Indian population. *Cancer Invest* **26**, 41-46 (2008).
242. Sreeja L, *et al.* Glutathione S-transferase M1, T1 and P1 polymorphisms: susceptibility and outcome in lung cancer patients. *J Exp Ther Oncol* **7**, 73-85 (2008).
243. Sreeja L, Syamala V, Hariharan S, Madhavan J, Devan SC, Ankathil R. Possible risk modification by CYP1A1, GSTM1 and GSTT1 gene polymorphisms in lung cancer susceptibility in a South Indian population. *J Hum Genet* **50**, 618-627 (2005).
244. Spitz MR, *et al.* Modulation of nucleotide excision repair capacity by XPD polymorphisms in lung cancer patients. *Cancer Res* **61**, 1354-1357 (2001).
245. Spitz MR, *et al.* Case-control study of the D2 dopamine receptor gene and smoking status in lung cancer patients. *J Natl Cancer Inst* **90**, 358-363 (1998).
246. Spitz MR, *et al.* Variants in inflammation genes are implicated in risk of lung cancer in never smokers exposed to second-hand smoke. *Cancer Discov* **1**, 420-429 (2011).
247. Spitz MR, *et al.* Dietary intake of isothiocyanates: evidence of a joint effect with glutathione S-transferase polymorphisms in lung cancer risk. *Cancer Epidemiol Biomarkers Prev* **9**, 1017-1020 (2000).
248. Spitz MR, *et al.* Role of selected genetic variants in lung cancer risk in African

Americans. *J Thorac Oncol* **8**, 391-397 (2013).
249. Spinola M, *et al.* Association of the PDCD5 locus with lung cancer risk and prognosis in smokers. *J Clin Oncol* **24**, 1672-1678 (2006).
250. Spinola M, *et al.* Genome-wide single nucleotide polymorphism analysis of lung cancer risk detects the KLF6 gene. *Cancer Lett* **251**, 311-316 (2007).
251. Spinola M, *et al.* Functional FGFR4 Gly388Arg polymorphism predicts prognosis in lung adenocarcinoma patients. *J Clin Oncol* **23**, 7307-7311 (2005).
252. Spinola M, *et al.* A new polymorphism (Ser362Thr) of the L-myc gene is not associated with lung adenocarcinoma risk and prognosis. *Eur J Cancer Prev* **13**, 87-89 (2004).
253. Soylemez F, Akbas E, Seyrek E, Mutluhan H, Camdeviren H. The relationship between the risk of lung cancer and the exon 5 (ILE105VAL) polymorphism of glutathione S-transferase P1 (GSTP1) gene. *Turkish Journal of Cancer* **36**, 5-10 (2006).
254. Souto-Garcia A, Fernandez-Somoano A, Pascual T, Alvarez-Avellon SM, Tardon A. Association of p21 Ser31Arg and p53 Arg72Pro polymorphisms with lung cancer risk in CAPUA study. *Lung Cancer Targets Ther* **3**, 69-78 (2012).
255. Sorensen M, Raaschou-Nielsen O, Hansen RD, Tjonneland A, Overvad K, Vogel U. Interactions between the OGG1 Ser326Cys polymorphism and intake of fruit and vegetables in relation to lung cancer. *Free Radic Res* **40**, 885-891 (2006).
256. Sorensen M, Raaschou-Nielsen O, Brasch-Andersen C, Tjonneland A, Overvad K, Autrup H. Interactions between GSTM1, GSTT1 and GSTP1 polymorphisms and smoking and intake of fruit and vegetables in relation to lung cancer. *Lung Cancer* **55**, 137-144 (2007).
257. Sorensen M, Autrup H, Tjonneland A, Overvad K, Raaschou-Nielsen O. Genetic polymorphisms in CYP1B1, GSTA1, NQO1 and NAT2 and the risk of lung cancer. *Cancer Lett* **221**, 185-190 (2005).
258. Sorensen M, Autrup H, Tjonneland A, Overvad K, Raaschou-Nielsen O. A genetic polymorphism in prostaglandin synthase 2 (8473, T->C) and the risk of lung cancer. *Cancer Lett* **226**, 49-54 (2005).
259. Sorensen M, Autrup H, Tjonneland A, Overvad K, Raaschou-Nielsen O. Glutathione S-transferase T1 null-genotype is associated with an increased risk of lung cancer. *Int J Cancer* **110**, 219-224 (2004).
260. Song N, Tan W, Xing D, Lin D. CYP 1A1 polymorphism and risk of lung cancer in relation to tobacco smoking: a case-control study in China. *Carcinogenesis* **22**, 11-16 (2001).
261. Son JW, *et al.* Polymorphisms in the caspase-8 gene and the risk of lung cancer. *Cancer Genet Cytogenet* **169**, 121-127 (2006).
262. Sobti RC, Sharma S, Joshi A, Jindal SK, Janmeja A. Genetic polymorphism of the CYP1A1, CYP2E1, GSTM1 and GSTT1 genes and lung cancer susceptibility in a north indian population. *Mol Cell Biochem* **266**, 1-9 (2004).
263. Sobti RC, Sharma S, Joshi A, Jindal SK, Janmeja A. CYP1A1 and CYP2D6 polymorphism and risk of lung cancer in a North Indian population. *Biomarkers* **8**, 415-428 (2003).
264. Sobti RC, *et al.* Effects of cyclin D1 (CCND1) polymorphism on susceptibility to lung cancer in a North Indian population. *Cancer Genet Cytogenet* **170**, 108-114 (2006).
265. Sobti RC, *et al.* Impact of interaction of polymorphic forms of p53 codon 72 and N-acetylation gene (NAT2) on the risk of lung cancer in the North Indian population. *DNA Cell Biol* **28**, 443-449 (2009).

266. Sobti RC, *et al.* Combined effect of GSTM1, GSTT1 and GSTP1 polymorphisms on histological subtypes of lung cancer. *Biomarkers* **13**, 282-295 (2008).
267. Sobti RC, *et al.* No association of DNA ligase-I polymorphism with the risk of lung cancer in north-Indian population. *DNA Cell Biol* **25**, 484-489 (2006).
268. Slovakova P, Majerova L, Matakova T, Skerenova M, Kavcova E, Halasova E. Mismatch repair gene polymorphisms and association with lung cancer development. *Adv Exp Med Biol* **833**, 15-22 (2015).
269. Sjalander A, Birgander R, Rannug A, Alexandrie AK, Tornling G, Beckman G. Association between the p21 codon 31 A1 (arg) allele and lung cancer. *Hum Hered* **46**, 221-225 (1996).
270. Sinues B, *et al.* MDR-1 C3435T genetic polymorphism and tobacco-related lung cancer. *Oncology* **64**, 183-185 (2003).
271. Singh AP, *et al.* Polymorphism in cytochrome P450 1A2 and their interaction with risk factors in determining risk of squamous cell lung carcinoma in men. *Cancer Biomark* **8**, 351-359 (2010).
272. Siemianowicz K, *et al.* Methylenetetrahydrofolate reductase gene C677T and A1298C polymorphisms in patients with small cell and non-small cell lung cancer. *Oncol Rep* **10**, 1341-1344 (2003).
273. Siemianowicz K, *et al.* Fibronectin gene polymorphism in patients with lung cancer. *Oncol Rep* **8**, 1289-1292 (2001).
274. Shiraishi K, *et al.* Contribution of nicotine acetylcholine receptor polymorphisms to lung cancer risk in a smoking-independent manner in the Japanese. *Carcinogenesis* **30**, 65-70 (2009).
275. Shin MC, *et al.* Glu346Lys polymorphism in the methyl-CpG binding domain 4 gene and the risk of primary lung cancer. *Jpn J Clin Oncol* **36**, 483-488 (2006).
276. Shih CM, Lin PT, Wang HC, Huang WC, Wang YC. Lack of evidence of association of p21WAF1/CIP1 polymorphism with lung cancer susceptibility and prognosis in Taiwan. *Jpn J Cancer Res* **91**, 9-15 (2000).
277. Shih CM, *et al.* The involvement of genetic polymorphism of IL-10 promoter in non-small cell lung cancer. *Lung Cancer* **50**, 291-297 (2005).
278. Shih CM, *et al.* Association of TNF-alpha polymorphism with susceptibility to and severity of non-small cell lung cancer. *Lung Cancer* **52**, 15-20 (2006).
279. Shih CM, *et al.* Association of L-myc polymorphism with lung cancer susceptibility and prognosis in relation to age-selected controls and stratified cases. *Lung Cancer* **36**, 125-132 (2002).
280. Shih CM, *et al.* Association of polymorphisms in the genes of the urokinase plasminogen activation system with susceptibility to and severity of non-small cell lung cancer. *Clin Chim Acta* **412**, 194-198 (2011).
281. Shih CM, Chen CY, Lee IH, Kao WT, Wang YC. A polymorphism in the hMLH1 gene (-93G-->A) associated with lung cancer susceptibility and prognosis. *Int J Mol Med* **25**, 165-170 (2010).
282. Shiels MS, *et al.* Genetic variation in innate immunity and inflammation pathways associated with lung cancer risk. *Cancer* **118**, 5630-5636 (2012).
283. Shields PG, *et al.* Lung cancer, race, and a CYP1A1 genetic polymorphism. *Cancer Epidemiol Biomarkers Prev* **2**, 481-485 (1993).
284. Shieh JM, *et al.* Lack of association of C-Met-N375S sequence variant with lung cancer susceptibility and prognosis. *Int J Med Sci* **10**, 988-994 (2013).
285. Shi Y, *et al.* C10ORF97 is a novel tumor-suppressor gene of non-small-cell lung cancer and a functional variant of this gene increases the risk of non-small-cell

lung cancer. *Oncogene* **30**, 4107-4117 (2011).
286. Shi WX, Chen SQ. Frequencies of poor metabolizers of cytochrome P450 2C19 in esophagus cancer, stomach cancer, lung cancer and bladder cancer in Chinese population. *World J Gastroenterol* **10**, 1961-1963 (2004).
287. Shi W, Yang B, Li X, Sun S, Wang L, Jiao S. The effect of lysyl oxidase polymorphism on susceptibility and prognosis of nonsmall cell lung cancer. *Tumour Biol* **33**, 2379-2383 (2012).
288. Shi Q, Zhang Z, Neumann AS, Li G, Spitz MR, Wei Q. Case-control analysis of thymidylate synthase polymorphisms and risk of lung cancer. *Carcinogenesis* **26**, 649-656 (2005).
289. Shi Q, *et al.* Polymorphisms of methionine synthase and methionine synthase reductase and risk of lung cancer: a case-control analysis. *Pharmacogenet Genomics* **15**, 547-555 (2005).
290. Shi Q, *et al.* Sex differences in risk of lung cancer associated with methylene-tetrahydrofolate reductase polymorphisms. *Cancer Epidemiol Biomarkers Prev* **14**, 1477-1484 (2005).
291. Shen M, *et al.* Polymorphisms in innate immunity genes and lung cancer risk in Xuanwei, China. *Environ Mol Mutagen* **50**, 285-290 (2009).
292. Shen M, *et al.* Polymorphisms in folate metabolic genes and lung cancer risk in Xuan Wei, China. *Lung Cancer* **49**, 299-309 (2005).
293. Shen M, *et al.* Polymorphisms in the DNA base excision repair genes APEX1 and XRCC1 and lung cancer risk in Xuan Wei, China. *Anticancer Res* **25**, 537-542 (2005).
294. Shen M, *et al.* Polymorphisms in the DNA nucleotide excision repair genes and lung cancer risk in Xuan Wei, China. *Int J Cancer* **116**, 768-773 (2005).
295. Shen L, Yin Z, Wu W, Ren Y, Li X, Zhou B. Single nucleotide polymorphism in ATM gene, cooking oil fumes and lung adenocarcinoma susceptibility in Chinese female non-smokers: A case-control study. *PLoS One* **9**, (2014).
296. Shen H, Wang L, Spitz MR, Hong WK, Mao L, Wei Q. A novel polymorphism in human cytosine DNA-methyltransferase-3B promoter is associated with an increased risk of lung cancer. *Cancer Res* **62**, 4992-4995 (2002).
297. Shen H, Spitz MR, Wang LE, Hong WK, Wei Q. Polymorphisms of methylene-tetrahydrofolate reductase and risk of lung cancer: a case-control study. *Cancer Epidemiol Biomarkers Prev* **10**, 397-401 (2001).
298. Shen H, Spitz MR, Qiao Y, Zheng Y, Hong WK, Wei Q. Polymorphism of DNA ligase I and risk of lung cancer--a case-control analysis. *Lung Cancer* **36**, 243-247 (2002).
299. Shen B, Zhu Q, Zheng MQ, Chen J, Shi MQ, Feng JF. CHRNA5 polymorphism and susceptibility to lung cancer in a Chinese population. *Braz J Med Biol Res* **46**, 79-84 (2013).
300. Shen B, Shi MQ, Zheng MQ, Hu SN, Chen J, Feng JF. Correlation between polymorphisms of nicotine acetylcholine acceptor subunit CHRNA3 and lung cancer susceptibility. *Mol Med Rep* **6**, 1389-1392 (2012).
301. Shaw GL, *et al.* Frequency of the variant allele CYP2D6(C) among North American Caucasian lung cancer patients and controls. *Lung Cancer* **17**, 61-68 (1997).
302. Shaw GL, *et al.* Genetic polymorphism of CYP2D6 and lung cancer risk. *Cancer Epidemiol Biomarkers Prev* **7**, 215-219 (1998).
303. Shao M, *et al.* Polymorphisms in excision repair cross-complementing group 4 (ERCC4) and susceptibility to primary lung cancer in a Chinese Han population.

Lung Cancer **60**, 332-339 (2008).
304. Shah PP, *et al.* Interaction of cytochrome P4501A1 genotypes with other risk factors and susceptibility to lung cancer. *Mutat Res* **639**, 1-10 (2008).
305. Shah PP, *et al.* Association of functionally important polymorphisms in cytochrome P4501B1 with lung cancer. *Mutat Res* **643**, 4-10 (2008).
306. Shaffi SM, Shah MA, Bhat IA, Koul P, Ahmad SN, Siddiqi MA. CYP1A1 polymorphisms and risk of lung cancer in the ethnic Kashmiri population. *Asian Pac J Cancer Prev* **10**, 651-656 (2009).
307. Sgambato A, *et al.* Glutathione S-transferase (GST) polymorphisms as risk factors for cancer in a highly homogeneous population from southern Italy. *Anticancer Res* **22**, 3647-3652 (2002).
308. Seow A, *et al.* NAT2 slow acetylator genotype is associated with increased risk of lung cancer among non-smoking Chinese women in Singapore. *Carcinogenesis* **20**, 1877-1881 (1999).
309. Seow A, *et al.* Cytochrome P4501A2 (CYP1A2) activity and lung cancer risk: a preliminary study among Chinese women in Singapore. *Carcinogenesis* **22**, 673-677 (2001).
310. Seow A, *et al.* Joint effect of asthma/atopy and an IL-6 gene polymorphism on lung cancer risk among lifetime non-smoking Chinese women. *Carcinogenesis* **27**, 1240-1244 (2006).
311. Seifart C, Seifart U, Plagens A, Wolf M, von Wichert P. Surfactant protein B gene variations enhance susceptibility to squamous cell carcinoma of the lung in German patients. *Br J Cancer* **87**, 212-217 (2002).
312. Seifart C, *et al.* TNF-alpha, TNF-beta, IL-6, and IL-10 polymorphisms in patients with lung cancer. *Dis Markers* **21**, 157-165 (2005).
313. Schwartz AG, Cote ML, Wenzlaff AS, Land S, Amos CI. Racial differences in the association between SNPs on 15q25.1, smoking behavior, and risk of non-small cell lung cancer. *J Thorac Oncol* **4**, 1195-1201 (2009).
314. Schneider J, Classen V, Bernges U, Philipp M. XRCC1 polymorphism and lung cancer risk in relation to tobacco smoking. *Int J Mol Med* **16**, 709-716 (2005).
315. Schneider J, Bernges U, Philipp M, Woitowitz HJ. GSTM1, GSTT1, and GSTP1 polymorphism and lung cancer risk in relation to tobacco smoking. *Cancer Lett* **208**, 65-74 (2004).
316. Schneider J, Bernges U. CYP1A1 and CYP1B1 polymorphisms as modifying factors in patients with pneumoconiosis and occupationally related tumours: A pilot study. *Mol Med Report* **2**, 1023-1028 (2009).
317. Schenk S, Schraml P, Bendik I, Ludwig CU. A novel polymorphism in the promoter of the RAGE gene is associated with non-small cell lung cancer. *Lung Cancer* **32**, 7-12 (2001).
318. Schabath MB, *et al.* A myeloperoxidase polymorphism associated with reduced risk of lung cancer. *Lung Cancer* **37**, 35-40 (2002).
319. Sauter W, *et al.* Matrix metalloproteinase 1 (MMP1) is associated with early-onset lung cancer. *Cancer Epidemiol Biomarkers Prev* **17**, 1127-1135 (2008).
320. Sarlinova M, *et al.* Polymorphisms of DNA repair genes and lung cancer in chromium exposure. *Adv Exp Med Biol* **833**, 1-8 (2015).
321. San Jose C, Cabanillas A, Benitez J, Carrillo JA, Jimenez M, Gervasini G. CYP1A1 gene polymorphisms increase lung cancer risk in a high-incidence region of Spain: a case control study. *BMC Cancer* **10**, 463 (2010).
322. Sameni S, Ghayumi MA, Mortazavi G, Faghih Z, Kashef MA, Ghaderi A. Lack

of association between interleukin-13 gene polymorphisms (-1055 C/T and +2044 G/A) in Iranian patients with lung cancer. *Mol Biol Rep* **36**, 1001-1005 (2009).
323. Saldivar SJ, *et al.* An association between a NQO1 genetic polymorphism and risk of lung cancer. *Mutat Res* **582**, 71-78 (2005).
324. Salagovic J, *et al.* Genetic polymorphism of glutathione S-transferases M1 and T1 as a risk factor in lung and bladder cancers. *Neoplasma* **45**, 312-317 (1998).
325. Sakoda LC, *et al.* Chromosome 15q24-25.1 variants, diet, and lung cancer susceptibility in cigarette smokers. *Cancer Causes Control* **22**, 449-461 (2011).
326. Sakoda LC, *et al.* Germ line variation in nucleotide excision repair genes and lung cancer risk in smokers. *Int J Mol Epidemiol Genet* **3**, 1-17 (2012).
327. Sakiyama T, *et al.* Association of amino acid substitution polymorphisms in DNA repair genes TP53, POLI, REV1 and LIG4 with lung cancer risk. *Int J Cancer* **114**, 730-737 (2005).
328. Saikia BJ, Phukan RK, Sharma SK, Sekhon GS, Mahanta J. Interaction of XRCC1 and XPD gene polymorphisms with lifestyle and environmental factors regarding susceptibility to lung cancer in a high incidence population in North East India. *Asian Pac J Cancer Prev* **15**, 1993-1999 (2014).
329. Saarikoski ST, *et al.* Combined effect of polymorphic GST genes on individual susceptibility to lung cancer. *Int J Cancer* **77**, 516-521 (1998).
330. Saarikoski ST, *et al.* Role of NAT2 deficiency in susceptibility to lung cancer among asbestos-exposed individuals. *Pharmacogenetics* **10**, 183-185 (2000).
331. Ryk C, Kumar R, Thirumaran RK, Hou SM. Polymorphisms in the DNA repair genes XRCC1, APEX1, XRCC3 and NBS1, and the risk for lung cancer in never- and ever-smokers. *Lung Cancer* **54**, 285-292 (2006).
332. Ryberg D, *et al.* Allele diversity of the H-ras-1 variable number of tandem repeats in Norwegian lung cancer patients. *Environ Health Perspect* **98**, 187-189 (1992).
333. Ryberg D, *et al.* Genotypes of glutathione transferase M1 and P1 and their significance for lung DNA adduct levels and cancer risk. *Carcinogenesis* **18**, 1285-1289 (1997).
334. Ryan BM, *et al.* MDM2 SNP285 does not antagonize the effect of SNP309 in lung cancer. *Int J Cancer* **131**, 2710-2716 (2012).
335. Rusin M, *et al.* Intronic Polymorphism (1541-1542deIGT) of the Constitutive Heat Shock Protein 70 Gene Has Functional Significance and Shows Evidence of Association with Lung Cancer Risk. *Mol Carcinog* **39**, 155-163 (2004).
336. Rudd MF, *et al.* Variants in the GH-IGF axis confer susceptibility to lung cancer. *Genome Res* **16**, 693-701 (2006).
337. Ruano-Ravina A, Pereyra MF, Castro MT, Perez-Rios M, Abal-Arca J, Barros-Dios JM. Genetic susceptibility, residential radon, and lung cancer in a radon prone area. *J Thorac Oncol* **9**, 1073-1080 (2014).
338. Ruano-Ravina A, Figueiras A, Loidi L, Barros-Dios JM. GSTM1 and GSTT1 polymorphisms, tobacco and risk of lung cancer: a case-control study from Galicia, Spain. *Anticancer Res* **23**, 4333-4337 (2003).
339. Rotunno M, *et al.* Inherited polymorphisms in the RNA-mediated interference machinery affect microRNA expression and lung cancer survival. *Br J Cancer* **103**, 1870-1874 (2010).
340. Rotunno M, *et al.* Phase I metabolic genes and risk of lung cancer: multiple polymorphisms and mRNA expression. *PLoS One* **4**, e5652 (2009).
341. Rotunno M, *et al.* GSTM1 and GSTT1 copy numbers and mRNA expression in

lung cancer. *Mol Carcinog* **51 Suppl 1**, E142-150 (2012).
342. Rosvold EA, McGlynn KA, Lustbader ED, Buetow KH. Identification of an NAD(P)H:quinone oxidoreductase polymorphism and its association with lung cancer and smoking. *Pharmacogenetics* **5**, 199-206 (1995).
343. Rosell R, *et al.* Genetic susceptibility associated with rare HRAS1 variable number of tandem repeats alleles in Spanish non-small cell lung cancer patients. *Clin Cancer Res* **5**, 1849-1854 (1999).
344. Rogers C, *et al.* Two novel insertion polymorphisms of the BRM gene are associated with loss of BRM expression and lung cancer risk. *Cancer Res* **70**, (2010).
345. Risch A, *et al.* Glutathione-S-transferase M1, M3, T1 and P1 polymorphisms and susceptibility to non-small-cell lung cancer subtypes and hamartomas. *Pharmacogenetics* **11**, 757-764 (2001).
346. Ribeiro R, *et al.* A functional polymorphism in the promoter region of leptin gene increases susceptibility for non-small cell lung cancer. *Eur J Cancer* **42**, 1188-1193 (2006).
347. Reszka E, Wasowicz W, Gromadzinska J. Antioxidant defense markers modulated by glutathione S-transferase genetic polymorphism: Results of lung cancer case-control study. *Genes and Nutrition* **2**, 287-294 (2007).
348. Ren YW, *et al.* P53 Arg72Pro and MDM2 SNP309 polymorphisms cooperate to increase lung adenocarcinoma risk in Chinese female non-smokers: a case control study. *Asian Pac J Cancer Prev* **14**, 5415-5420 (2013).
349. Ren JH, *et al.* Association between CHRNA3 rs1051730 genotype and lung cancer risk in Chinese Han population: a case-control study. *J Huazhong Univ Sci Technolog Med Sci* **33**, 897-901 (2013).
350. Razmkhah M, Doroudchi M, Ghayumi SM, Erfani N, Ghaderi A. Stromal cell-derived factor-1 (SDF-1) gene and susceptibility of Iranian patients with lung cancer. *Lung Cancer* **49**, 311-315 (2005).
351. Ratnasinghe D, *et al.* Polymorphisms of the DNA repair gene XRCC1 and lung cancer risk. *Cancer Epidemiol Biomarkers Prev* **10**, 119-123 (2001).
352. Ratnasinghe D, *et al.* Influence of antioxidants and the CYP1A1 isoleucine to valine polymorphism on the smoking--lung cancer association. *Anticancer Res* **21**, 1295-1299 (2001).
353. Ratnasinghe D, *et al.* Glutathione peroxidase codon 198 polymorphism variant increases lung cancer risk. *Cancer Res* **60**, 6381-6383 (2000).
354. Ramzy MM, Solliman MEDM, Abdel-Hafiz HA, Salah R. Genetic polymorphism of GSTM1 and GSTP1 in lung cancer in Egypt. *Int J Collab Res Intern Med Public Health* **3**, 41-51 (2011).
355. Rafrafi A, *et al.* Association of IL-8 gene polymorphisms with non small cell lung cancer in Tunisia: A case control study. *Hum Immunol* **74**, 1368-1374 (2013).
356. Raaschou-Nielsen O, Sorensen M, Overvad K, Tjonneland A, Vogel U. Polymorphisms in nucleotide excision repair genes, smoking and intake of fruit and vegetables in relation to lung cancer. *Lung Cancer* **59**, 171-179 (2008).
357. Raaschou-Nielsen O, *et al.* GPX1 Pro198Leu polymorphism, interactions with smoking and alcohol consumption, and risk for lung cancer. *Cancer Lett* **247**, 293-300 (2007).
358. Quinones L, *et al.* CYP1A1, CYP2E1 and GSTM1 genetic polymorphisms. The effect of single and combined genotypes on lung cancer susceptibility in Chilean people. *Cancer Lett* **174**, 35-44 (2001).

359. Qiuling S, Yuxin Z, Suhua Z, Cheng X, Shuguang L, Fengsheng H. Cyclin D1 gene polymorphism and susceptibility to lung cancer in a Chinese population. *Carcinogenesis* **24**, 1499-1503 (2003).
360. Qiu F, *et al.* A functional polymorphism in the promoter of ERK5 gene interacts with tobacco smoking to increase the risk of lung cancer in Chinese populations. *Mutagenesis* **28**, 561-567 (2013).
361. Qian J, *et al.* Association between polymorphisms in the GSTA4 gene and risk of lung cancer: a case-control study in a Southeastern Chinese population. *Mol Carcinog* **48**, 253-259 (2009).
362. Qian B, Zhang H, Zhang L, Zhou X, Yu H, Chen K. Association of genetic polymorphisms in DNA repair pathway genes with non-small cell lung cancer risk. *Lung Cancer* **73**, 138-146 (2011).
363. Putra AC, Tanimoto K, Arifin M, Hiyama K. Hypoxia-inducible factor-1alpha polymorphisms are associated with genetic aberrations in lung cancer. *Respirology* **16**, 796-802 (2011).
364. Popanda O, *et al.* Specific combinations of DNA repair gene variants and increased risk for non-small cell lung cancer. *Carcinogenesis* **25**, 2433-2441 (2004).
365. Popanda O, *et al.* Elevated risk of squamous-cell carcinoma of the lung in heavy smokers carrying the variant alleles of the TP53 Arg72Pro and p21 Ser31Arg polymorphisms. *Lung Cancer* **55**, 25-34 (2007).
366. Piskac-Collier AL, *et al.* Variants in folate pathway genes as modulators of genetic instability and lung cancer risk. *Genes Chromosomes Cancer* **50**, 1-12 (2011).
367. Pisani P, *et al.* GSTM1 and CYP1A1 polymorphisms, tobacco, air pollution, and lung cancer: a study in rural Thailand. *Cancer Epidemiol Biomarkers Prev* **15**, 667-674 (2006).
368. Pine SR, *et al.* MDM2 SNP309 and SNP354 are not associated with lung cancer risk. *Cancer Epidemiol Biomarkers Prev* **15**, 1559-1561 (2006).
369. Pinarbasi H, Silig Y, Cetinkaya O, Seyfikli Z, Pinarbasi E. Strong association between the GSTM1-null genotype and lung cancer in a Turkish population. *Cancer Genet Cytogenet* **146**, 125-129 (2003).
370. Pierce LM, *et al.* Relationships of TP53 codon 72 and HRAS1 polymorphisms with lung cancer risk in an ethnically diverse population. *Cancer Epidemiol Biomarkers Prev* **9**, 1199-1204 (2000).
371. Piao JM, *et al.* Glutathione-S-transferase (GSTM1, GSTT1) null phenotypes and risk of lung cancer in a Korean population. *Asian Pac J Cancer Prev* **14**, 7165-7169 (2013).
372. Piao JM, *et al.* p53 codon 72 polymorphism and the risk of lung cancer in a Korean population. *Lung Cancer* **73**, 264-267 (2011).
373. Phang BH, Chua HW, Li H, Linn YC, Sabapathy K. Characterization of novel and uncharacterized p53 SNPs in the chinese population - Intron 2 SNP co-segregates with the common codon 72 polymorphism. *PLoS One* **6**, (2011).
374. Persson I, *et al.* Genetic polymorphism of xenobiotic metabolizing enzymes among Chinese lung cancer patients. *Int J Cancer* **81**, 325-329 (1999).
375. Persson I, *et al.* Genetic polymorphism of cytochrome P4502E1 in a Swedish population. Relationship to incidence of lung cancer. *FEBS Lett* **319**, 207-211 (1993).
376. Perez-Morales R, *et al.* Genetic susceptibility to lung cancer based on candidate genes in a sample from the Mexican Mestizo population: a case-control study.

Lung **192**, 167-173 (2014).
377. Perera FP, *et al.* Associations between carcinogen-DNA damage, glutathione S-transferase genotypes, and risk of lung cancer in the prospective Physicians' Health Cohort Study. *Carcinogenesis* **23**, 1641-1646 (2002).
378. Pavanello S, *et al.* Role of CYP1A2 polymorphisms on lung cancer risk in a prospective study. *Cancer Genet* **205**, 278-284 (2012).
379. Paulus JK, Zhou W, Kraft P, Johnson BE, Lin X, Christiani DC. Haplotypes of estrogen receptor-beta and risk of non-small cell lung cancer in women. *Lung Cancer* **71**, 258-263 (2011).
380. Park SL, *et al.* Associations between variants of the 8q24 chromosome and nine smoking-related cancer sites. *Cancer Epidemiol Biomarkers Prev* **17**, 3193-3202 (2008).
381. Park SL, *et al.* Associations between NBS1 polymorphisms, haplotypes and smoking-related cancers. *Carcinogenesis* **31**, 1264-1271 (2010).
382. Park SH, *et al.* -93G->A polymorphism of hMLH1 and risk of primary lung cancer. *Int J Cancer* **112**, 678-682 (2004).
383. Park SH, *et al.* Polymorphisms in the FAS and FASL genes and risk of lung cancer in a Korean population. *Lung Cancer* **54**, 303-308 (2006).
384. Park SH, *et al.* MDM2 309T>G polymorphism and risk of lung cancer in a Korean population. *Lung Cancer* **54**, 19-24 (2006).
385. Park KH, *et al.* Single nucleotide polymorphisms of the TGFB1 gene and lung cancer risk in a Korean population. *Cancer Genet Cytogenet* **169**, 39-44 (2006).
386. Park JY, *et al.* Polymorphisms of the DNA repair gene xeroderma pigmentosum group A and risk of primary lung cancer. *Cancer Epidemiol Biomarkers Prev* **11**, 993-997 (2002).
387. Park JY, *et al.* Caspase 9 promoter polymorphisms and risk of primary lung cancer. *Hum Mol Genet* **15**, 1963-1971 (2006).
388. Park JY, *et al.* Impact of smoking on lung cancer risk is stronger in those with the homozygous aldehyde dehydrogenase 2 null allele in a Japanese population. *Carcinogenesis* **31**, 660-665 (2010).
389. Park JY, *et al.* Lys751Gln polymorphism in the DNA repair gene XPD and risk of primary lung cancer. *Lung Cancer* **36**, 15-16 (2002).
390. Park JY, *et al.* Polymorphism of the DNA repair gene XRCC1 and risk of primary lung cancer. *Cancer Epidemiol Biomarkers Prev* **11**, 23-27 (2002).
391. Park JY, Chen L, Lee J, Sellers T, Tockman MS. Polymorphisms in the promoter region of neutrophil elastase gene and lung cancer risk. *Lung Cancer* **48**, 315-321 (2005).
392. Park JY, Chen L, Elahi A, Lazarus P, Tockman MS. Genetic analysis of microsomal epoxide hydrolase gene and its association with lung cancer risk. *Eur J Cancer Prev* **14**, 223-230 (2005).
393. Park JM, *et al.* No association between polymorphisms in the histone deacetylase genes and the risk of lung cancer. *Cancer Epidemiol Biomarkers Prev* **14**, 1841-1843 (2005).
394. Park JM, *et al.* Relationship between cyclooxygenase 8473T>C polymorphism and the risk of lung cancer: A case-control study. *BMC Cancer* **6**, (2006).
395. Park JH, *et al.* Myeloperoxidase -463G>A polymorphism and risk of primary lung cancer in a Korean population. *Cancer Detect Prev* **30**, 257-261 (2006).
396. Park J, Chen L, Tockman MS, Elahi A, Lazarus P. The human 8-oxoguanine DNA N-glycosylase 1 (hOGG1) DNA repair enzyme and its association with lung cancer risk. *Pharmacogenetics* **14**, 103-109 (2004).

397. Papadakis ED, Soulitzis N, Spandidos DA. Association of p53 codon 72 polymorphism with advanced lung cancer: the Arg allele is preferentially retained in tumours arising in Arg/Pro germline heterozygotes. *Br J Cancer* **87**, 1013-1018 (2002).
398. Pandima Devi K, Sivamaruthi B, Kiruthiga PV, Karutha Pandian S. Study of p53 codon 72 polymorphism and codon 249 mutations in Southern India in relation to age, alcohol drinking and smoking habits. *Hum Exp Toxicol* **29**, 451-458 (2010).
399. Pan H, *et al.* Contributory role of five common polymorphisms of RAGE and APE1 genes in lung cancer among Han Chinese. *PLoS One* **8**, e69018 (2013).
400. Pan C, Zhu G, Yan Z, Zhou Y, Liu Z. Glutathione S-transferase T1 and M1 polymorphisms are associated with lung cancer risk in a gender-specific manner. *Oncol Res Treat* **37**, 164-169 (2014).
401. Pachouri SS, Sobti RC, Kaur P, Singh J, Gupta SK. Impact of polymorphism in sulfotransferase gene on the risk of lung cancer. *Cancer Genet Cytogenet* **171**, 39-43 (2006).
402. Pachouri SS, Sobti RC, Kaur P, Singh J. Contrasting impact of DNA repair gene XRCC1 polymorphisms Arg399Gln and Arg194Trp on the risk of lung cancer in North-Indian population. *DNA Cell Biol* **26**, 186-191 (2007).
403. Ozturk O, Isbir T, Yaylim I, Kocaturk CI, Gurses A. GST M1 and CYP1A1 gene polymorphism and daily fruit consumption in Turkish patients with non-small cell lung carcinomas. *In Vivo* **17**, 625-632 (2003).
404. Ozen F, Polat F, Arslan S, Ozdemir O. Combined germline variations of thrombophilic genes promote genesis of lung cancer. *Asian Pac J Cancer Prev* **14**, 5449-5454 (2013).
405. Ozbey U, Yuce H, Namli M, Elkiran T. Investigation of differences in P53 Gene polymorphisms between schizophrenia and lung cancer patients in the Turkish population. *Genetics Research International* **2011**, (2011).
406. Oyama T, Kawamoto T, Mizoue T, Yasumoto K, Kodama Y, Mitsudomi T. N-acetylation polymorphism in patients with lung cancer and its association with p53 gene mutation. *Anticancer Res* **17**, 577-581 (1997).
407. Oyama T, *et al.* Cytochrome P4S0 2E1 polymorphism as a risk factor for lung cancer: In relation to p53 gene mutation. *Anticancer Res* **17**, 583-587 (1997).
408. Ouyang FD, *et al.* Polymorphisms of DNA repair genes XPD, XRCC1, and OGG1, and lung adenocarcinoma susceptibility in Chinese population. *Tumour Biol* **34**, 2843-2848 (2013).
409. Osawa Y, *et al.* NAT2 and CYP1A2 polymorphisms and lung cancer risk in relation to smoking status. *Asian Pac J Cancer Prev* **8**, 103-108 (2007).
410. Osawa K, *et al.* APEX1 Asp148Glu gene polymorphism is a risk factor for lung cancer in relation to smoking in Japanese. *Asian Pac J Cancer Prev* **11**, 1181-1186 (2010).
411. Oltulu YM, *et al.* Investigation of NF-kappaB1 and NF-kappaBIA gene polymorphism in non-small cell lung cancer. *Biomed Res Int* **2014**, 530381 (2014).
412. Okasaka T, *et al.* hOGG1 Ser326Cys polymorphism and risk of lung cancer by histological type. *J Hum Genet* **54**, 739-745 (2009).
413. Oh SS, *et al.* Single nucleotide polymorphisms of 8 inflammation-related genes and their associations with smoking-related cancers. *Int J Cancer* **127**, 2169-2182 (2010).
414. Noda N, Matsuzoe D, Konno T, Kawahara K, Yamashita Y, Shirakusa T. Risk for

K-ras gene mutations in smoking-induced lung cancer is associated with cytochrome P4501A1 and glutathione S-transferase micro1 polymorphisms. *Oncol Rep* **12**, 773-779 (2004).
415. Niu X, *et al.* Association of the CHRNA3 locus with lung cancer risk and prognosis in Chinese Han population. *J Thorac Oncol* **5**, 658-666 (2010).
416. Nikishina MV, Vavilin VA, Makarova SI, Lyakhovich VV. Analysis of associations of NAT2 gene polymorphisms with the risk of lung cancer. *Bull Exp Biol Med* **143**, 83-86 (2007).
417. Nichenametla SN, Muscat JE, Liao JG, Lazarus P, Richie JP, Jr. A functional trinucleotide repeat polymorphism in the 5'-untranslated region of the glutathione biosynthetic gene GCLC is associated with increased risk for lung and aerodigestive tract cancers. *Mol Carcinog* **52**, 791-799 (2013).
418. Ng DP, Tan KW, Zhao B, Seow A. CYP1A1 polymorphisms and risk of lung cancer in non-smoking Chinese women: influence of environmental tobacco smoke exposure and GSTM1/T1 genetic variation. *Cancer Causes Control* **16**, 399-405 (2005).
419. Nazar-Stewart V, Vaughan TL, Stapleton P, Van Loo J, Nicol-Blades B, Eaton DL. A population-based study of glutathione S-transferase M1, T1 and P1 genotypes and risk for lung cancer. *Lung Cancer* **40**, 247-258 (2003).
420. Natukula K, Jamil K, Pingali UR, Attili VS, Madireddy UR. The codon 399 Arg/Gln XRCC1 polymorphism is associated with lung cancer in Indians. *Asian Pac J Cancer Prev* **14**, 5275-5279 (2013).
421. Nanba K, *et al.* The allelic distribution of a single nucleotide polymorphism in the PDCD5 gene locus of Japanese non-small cell lung cancer patients. *Mol Med Report* **1**, 667-671 (2008).
422. Nakane H, *et al.* Impact of metallothionein gene polymorphisms on the risk of lung cancer in a Japanese population. *Mol Carcinog*, (2014).
423. Nakachi K, Imai K, Hayashi S, Kawajiri K. Polymorphisms of the CYP1A1 and glutathione S-transferase genes associated with susceptibility to lung cancer in relation to cigarette dose in a Japanese population. *Cancer Res* **53**, 2994-2999 (1993).
424. Nakachi K, Imai K, Hayashi -i S, Watanabe J, Kawajiri K. Genetic susceptibility to squamous cell carcinoma of the lung in relation to cigarette smoking dose. *Cancer Res* **51**, 5177-5180 (1991).
425. Nakachi K, Hayashi S, Kawajiri K, Imai K. Association of cigarette smoking and CYP1A1 polymorphisms with adenocarcinoma of the lung by grades of differentiation. *Carcinogenesis* **16**, 2209-2213 (1995).
426. Naik NA, *et al.* Vascular endothelial growth factor A gene (VEGFA) polymorphisms and expression of VEGFA gene in lung cancer patients of Kashmir Valley (India). *Tumour Biol* **33**, 833-839 (2012).
427. Nadji SA, *et al.* An increased lung cancer risk associated with codon 72 polymorphism in the TP53 gene and human papillomavirus infection in Mazandaran province, Iran. *Lung Cancer* **56**, 145-151 (2007).
428. Nacak M, Nacak I, Sanl SM, Ozkur M, Pektas M, Aynacioglu AT. Association of angiotensin converting enzyme gene insertion/deletion polymorphism with lung cancer in turkey. *Cancer Genet Cytogenet* **198**, 22-26 (2010).
429. Myneni AA, *et al.* Genetic polymorphisms of TERT and CLPTM1L and risk of lung cancer-A case-control study in a Chinese population. *Lung Cancer* **80**, 131-137 (2013).
430. Murata M, Tagawa M, Kimura H, Kakisawa K, Shirasawa H, Fujisawa T.

Correlation of the mutation of p53 gene and the polymorphism at codon 72 in smoking-related non-small cell lung cancer patients. *Int J Oncol* **12**, 577-581 (1998).
431. Motovali-Bashi M, Rezaei H, Dehghanian F, Rezaei H. Association between XPD (Lys751G1n) polymorphism and lung cancer risk: A population-based study in Iran. *Cell Journal* **16**, 309-314 (2014).
432. Motovali-Bashi M, Biglari M, Hojati Z, Hemati S, Khodadad K. Role of CYP1A1 MspI polymorphism in CYP1A1 gene with susceptibility to lung cancer in Iranian patients. *J Res Med Sci* **17**, S242-S246 (2012).
433. Mota P, Moura DS, Vale MG, Coimbra H, Carvalho L, Regateiro F. CYP1A1 m1 and m2 polymorphisms: genetic susceptibility to lung cancer. *Rev Port Pneumol* **16**, 89-98 (2010).
434. Mostaid MS, Ahmed MU, Islam MS, Bin Sayeed MS, Hasnat A. Lung cancer risk in relation to TP53 codon 47 and codon 72 polymorphism in Bangladeshi population. *Tumour Biol* **35**, 10309-10317 (2014).
435. Moreira A, *et al.* Glutathione S-transferase mu polymorphism and susceptibility to lung cancer in the Portuguese population. *Teratog Carcinog Mutagen* **16**, 269-274 (1996).
436. Moon JW, *et al.* Promoter -202 A/C polymorphism of insulin-like growth factor binding protein-3 gene and non-small cell lung cancer risk. *Int J Cancer* **118**, 353-356 (2006).
437. Miyamoto M, *et al.* CYP2A6 gene deletion reduces susceptibility to lung cancer. *Biochem Biophys Res Commun* **261**, 658-660 (1999).
438. Miyaishi A, *et al.* MUTYH Gln324His gene polymorphism and genetic susceptibility for lung cancer in a Japanese population. *J Exp Clin Cancer Res* **28**, 10 (2009).
439. Mita Y, *et al.* Missense polymorphisms of PTPRJ and PTPN13 genes affect susceptibility to a variety of human cancers. *J Cancer Res Clin Oncol* **136**, 249-259 (2010).
440. Misra RR, *et al.* Variation in the promoter region of the myeloperoxidase gene is not directly related to lung cancer risk among male smokers in Finland. *Cancer Lett* **164**, 161-167 (2001).
441. Misra RR, *et al.* Polymorphisms in the DNA repair genes XPD, XRCC1, XRCC3, and APE/ref-1, and the risk of lung cancer among male smokers in Finland. *Cancer Lett* **191**, 171-178 (2003).
442. Minegishi Y, *et al.* Susceptibility to lung cancer and genetic polymorphisms in the alcohol metabolite-related enzymes alcohol dehydrogenase 3, aldehyde dehydrogenase 2, and cytochrome P450 2E1 in the Japanese population. *Cancer* **110**, 353-362 (2007).
443. Miller DP, *et al.* Combinations of the variant genotypes of GSTP1, GSTM1, and p53 are associated with an increased lung cancer risk. *Cancer Res* **62**, 2819-2823 (2002).
444. Miller DP, *et al.* An association between glutathione S-transferase P1 gene polymorphism and younger age at onset of lung carcinoma. *Cancer* **107**, 1570-1577 (2006).
445. Michiels S, *et al.* Polymorphism discovery in 62 DNA repair genes and haplotype associations with risks for lung and head and neck cancers. *Carcinogenesis* **28**, 1731-1739 (2007).
446. Miao R, *et al.* Tagging single nucleotide polymorphisms in MBD4 are associated with risk of lung cancer in a Chinese population. *Lung Cancer* **62**, 281-286

(2008).
447. Mei C, *et al.* Polymorphisms in DNA repair genes of XRCC1, XPA, XPC, XPD and associations with lung cancer risk in Chinese people. *Thoracic Cancer* **5**, 232-242 (2014).
448. Mechanic LE, *et al.* Common genetic variation in TP53 is associated with lung cancer risk and prognosis in African Americans and somatic mutations in lung tumors. *Cancer Epidemiol Biomarkers Prev* **16**, 214-222 (2007).
449. McKay JD, *et al.* Sequence variants of NAT1 and NAT2 and other xenometabolic genes and risk of lung and aerodigestive tract cancers in Central Europe. *Cancer Epidemiol Biomarkers Prev* **17**, 141-147 (2008).
450. Matullo G, *et al.* DNA repair polymorphisms and cancer risk in non-smokers in a cohort study. *Carcinogenesis* **27**, 997-1007 (2006).
451. Matakova T, *et al.* Gene polymorphisms of biotransforming enzymes (GSTs) and their association with lung cancer in the Slovakian population. *Eur J Med Res* **14 Suppl 4**, 275-279 (2009).
452. Marsit CJ, Wiencke JK, Liu M, Kelsey KT. The race associated allele of Semaphorin 3B (SEMA3B) T415I and its role in lung cancer in African-Americans and Latino-Americans. *Carcinogenesis* **26**, 1446-1449 (2005).
453. Marin MS, Lopez-Cima MF, Garcia-Castro L, Pascual T, Marron MG, Tardon A. Poly (AT) polymorphism in intron 11 of the XPC DNA repair gene enhances the risk of lung cancer. *Cancer Epidemiol Biomarkers Prev* **13**, 1788-1793 (2004).
454. Marchand LL, Seifried A, Lum A, Wilkens LR. Association of the myeloperoxidase -463G-> A polymorphism with lung cancer risk. *Cancer Epidemiol Biomarkers Prev* **9**, 181-184 (2000).
455. Mansoor Q, Fayyaz S, Farooqi AA, Bhatti S, Ismail M. Re-evaluating the FGFR4 (G388R) germline mutation in different cancers in Pakistani population. *J Exp Ther Oncol* **10**, 215-217 (2013).
456. Manenti G, *et al.* A V141L polymorphism of the human LRMP gene is associated with survival of lung cancer patients. *Carcinogenesis* **27**, 1386-1390 (2006).
457. Manenti G, *et al.* Association of chromosome 12p genetic polymorphisms with lung adenocarcinoma risk and prognosis. *Carcinogenesis* **18**, 1917-1920 (1997).
458. Malats N, *et al.* Lung cancer risk in nonsmokers and GSTM1 and GSTT1 genetic polymorphism. *Cancer Epidemiol Biomarkers Prev* **9**, 827-833 (2000).
459. Ma H, *et al.* Tagging single nucleotide polymorphisms in excision repair cross-complementing group 1 (ERCC1) and risk of primary lung cancer in a Chinese population. *Pharmacogenet Genomics* **17**, 417-423 (2007).
460. Ma H, *et al.* ERCC6/CSB gene polymorphisms and lung cancer risk. *Cancer Lett* **273**, 172-176 (2009).
461. Lubinski J, *et al.* The 3020insC allele of NOD2 predisposes to cancers of multiple organs. *Hered Cancer Clin Pract* **3**, 59-63 (2005).
462. Lu X, *et al.* The SNP rs402710 in 5p15.33 Is Associated with Lung Cancer Risk: A Replication Study in Chinese Population and a Meta-Analysis. *PLoS One* **8**, (2013).
463. Lu W, Xing D, Qi J, Tan W, Miao X, Lin D. Genetic polymorphism in myeloperoxidase but not GSTM1 is associated with risk of lung squamous cell carcinoma in a Chinese population. *Int J Cancer* **102**, 275-279 (2002).
464. Lu J, *et al.* Functional characterization of a promoter polymorphism in APE1/Ref-1 that contributes to reduced lung cancer susceptibility. *FASEB J* **23**,

3459-3469 (2009).
465. Lu J, *et al.* The polymorphism and haplotypes of PIN1 gene are associated with the risk of lung cancer in Southern and Eastern Chinese populations. *Hum Mutat* **32**, 1299-1308 (2011).
466. Loriot MA, *et al.* Genetic polymorphisms of cytochrome P450 2A6 in a case-control study on lung cancer in a French population. *Pharmacogenetics* **11**, 39-44 (2001).
467. Lopez-Cima MF, *et al.* Polymorphisms in XPC, XPD, XRCC1, and XRCC3 DNA repair genes and lung cancer risk in a population of northern Spain. *BMC Cancer* **7**, 162 (2007).
468. Lopez-Cima MF, Alvarez-Avellon SM, Pascual T, Fernandez-Somoano A, Tardon A. Genetic polymorphisms in CYP1A1, GSTM1, GSTP1 and GSTT1 metabolic genes and risk of lung cancer in Asturias. *BMC Cancer* **12**, 433 (2012).
469. London SJ, Yuan JM, Coetzee GA, Gao YT, Ross RK, Yu MC. CYP1A1 I462V genetic polymorphism and lung cancer risk in a cohort of men in Shanghai, China. *Cancer Epidemiol Biomarkers Prev* **9**, 987-991 (2000).
470. London SJ, *et al.* Isothiocyanates, glutathione S-transferase M1 and T1 polymorphisms, and lung-cancer risk: a prospective study of men in Shanghai, China. *Lancet* **356**, 724-729 (2000).
471. London SJ, Sullivan-Klose T, Daly AK, Idle JR. Lung cancer risk in relation to the CYP2C9 genetic polymorphism among Caucasians in Los Angeles County. *Pharmacogenetics* **7**, 401-404 (1997).
472. London SJ, Smart J, Daly AK. Lung cancer risk in relation to genetic polymorphisms of microsomal epoxide hydrolase among African-Americans and Caucasians in Los Angeles County. *Lung Cancer* **28**, 147-155 (2000).
473. London SJ, Lehman TA, Taylor JA. Myeloperoxidase genetic polymorphism and lung cancer risk. *Cancer Res* **57**, 5001-5003 (1997).
474. London SJ, Daly AK, Leathart JB, Navidi WC, Idle JR. Lung cancer risk in relation to the CYP2C9*1/CYP2C9*2 genetic polymorphism among African-Americans and Caucasians in Los Angeles County, California. *Pharmacogenetics* **6**, 527-533 (1996).
475. London SJ, *et al.* Lung cancer risk in African-Americans in relation to a race-specific CYP1A1 polymorphism. *Cancer Res* **55**, 6035-6037 (1995).
476. London SJ, Daly AK, Cooper J, Navidi WC, Carpenter CL, Idle JR. Polymorphism of glutathione S-transferase M1 and lung cancer risk among African-Americans and Caucasians in Los Angeles County, California. *J Natl Cancer Inst* **87**, 1246-1253 (1995).
477. London SJ, *et al.* Lung cancer risk in relation to the CYP2E1 Rsa I genetic polymorphism among African-Americans and Caucasians in Los Angeles County. *Pharmacogenetics* **6**, 151-158 (1996).
478. Lo YL, *et al.* A polymorphism in the APE1 gene promoter is associated with lung cancer risk. *Cancer Epidemiol Biomarkers Prev* **18**, 223-229 (2009).
479. Lo YL, *et al.* ATM polymorphisms and risk of lung cancer among never smokers. *Lung Cancer* **69**, 148-154 (2010).
480. Lo YL, *et al.* Polymorphisms of MLH1 and MSH2 genes and the risk of lung cancer among never smokers. *Lung Cancer* **72**, 280-286 (2011).
481. Liu SG, Yuan SH, Wu HY, Huang CS, Liu J. The programmed cell death 6 interacting protein insertion/deletion polymorphism is associated with non-small cell lung cancer risk in a Chinese Han population. *Tumour Biol* **35**, 8679-8683

(2014).
482. Liu L, *et al.* Variants in the 5'-upstream region of GPC5 confer risk of lung cancer in never smokers. *Cancer Epidemiol* **38**, 66-72 (2014).
483. Liu L, *et al.* A functional polymorphism (-1607 1G->2G) in the matrix metalloproteinase-1 promoter is associated with development and progression of lung cancer. *Cancer* **117**, 5172-5181 (2011).
484. Liu H, *et al.* Methyl-CpG binding domain 1 gene polymorphisms and lung cancer risk in a Chinese population. *Biomarkers* **13**, 607-617 (2008).
485. Liu H, *et al.* Association of polymorphisms in one-carbon metabolizing genes and lung cancer risk: a case-control study in Chinese population. *Lung Cancer* **61**, 21-29 (2008).
486. Liu H, Jiao Y, Guan Y, Lao Y, Zhao C, Fan H. The DNMT3B -579 G>T promoter polymorphism and risk of lung cancer. *Exp Ther Med* **3**, 525-529 (2012).
487. Liu G, *et al.* MPO and SOD2 polymorphisms, gender, and the risk of non-small cell lung carcinoma. *Cancer Lett* **214**, 69-79 (2004).
488. Liu G, *et al.* The SOD2 Val/Val genotype enhances the risk of nonsmall cell lung carcinoma by p53 and XRCC1 polymorphisms. *Cancer* **101**, 2802-2808 (2004).
489. Liu G, *et al.* Genetic polymorphisms of MDM2, cumulative cigarette smoking and nonsmall cell lung cancer risk. *Int J Cancer* **122**, 915-918 (2008).
490. Liu G, *et al.* Differential association of the codon 72 p53 and GSTM1 polymorphisms on histological subtype of non-small cell lung carcinoma. *Cancer Res* **61**, 8718-8722 (2001).
491. Liu D, *et al.* Association of glutathione S-transferase M1 polymorphisms and lung cancer risk in a Chinese population. *Clin Chim Acta* **414**, 188-190 (2012).
492. Liu D, *et al.* Association between p53 codon 72 genetic polymorphisms and tobacco use and lung cancer risk in a Chinese population. *Mol Biol Rep* **40**, 645-649 (2013).
493. Liu D, Li L, Yang Y, Liu W, Wu J. The Axin2 rs2240308 polymorphism and susceptibility to lung cancer in a Chinese population. *Tumour Biol*, (2014).
494. Liu CS, *et al.* Interaction of methylenetetrahydrofolate reductase genotype and smoking habit in Taiwanese lung cancer patients. *Cancer Genomics Proteomics* **6**, 325-329 (2009).
495. Liu CJ, *et al.* Interaction of cyclooxygenase 2 genotype and smoking habit in Taiwanese lung cancer patients. *Anticancer Res* **30**, 1195-1199 (2010).
496. Liu CJ, *et al.* The joint effect of hOGG1 single nucleotide polymorphism and smoking habit on lung cancer in Taiwan. *Anticancer Res* **30**, 4141-4145 (2010).
497. Liu B, *et al.* A functional copy-number variation in MAPKAPK2 predicts risk and prognosis of lung cancer. *Am J Hum Genet* **91**, 384-390 (2012).
498. Liu B, *et al.* A functional variant (-1304T>G) in the MKK4 promoter contributes to a decreased risk of lung cancer by increasing the promoter activity. *Carcinogenesis* **31**, 1405-1411 (2010).
499. Lindstedt BA, Ryberg D, Zienolddiny S, Khan H, Haugen A. Hras1 VNTR alleles as susceptibility markers for lung cancer: relationship to microsatellite instability in tumors. *Anticancer Res* **19**, 5523-5527 (1999).
500. Lind H, Zienolddiny S, Ryberg D, Skaug V, Phillips DH, Haugen A. Interleukin 1 receptor antagonist gene polymorphism and risk of lung cancer: a possible interaction with polymorphisms in the interleukin 1 beta gene. *Lung Cancer* **50**, 285-290 (2005).
501. Lind H, Zienolddiny S, Ekstrom PO, Skaug V, Haugen A. Association of a functional polymorphism in the promoter of the MDM2 gene with risk of

nonsmall cell lung cancer. *Int J Cancer* **119**, 718-721 (2006).
502. Lin Z, *et al.* A variant of the Cockayne syndrome B gene ERCC6 confers risk of lung cancer. *Hum Mutat* **29**, 113-122 (2008).
503. Lin P, *et al.* Association of CYP1A1 and microsomal epoxide hydrolase polymorphisms with lung squamous cell carcinoma. *Br J Cancer* **82**, 852-857 (2000).
504. Lin P, Hsueh YM, Ko JL, Liang YF, Tsai KJ, Chen CY. Analysis of NQO1, GSTP1, and MnSOD genetic polymorphisms on lung cancer risk in Taiwan. *Lung Cancer* **40**, 123-129 (2003).
505. Lin J, *et al.* Systematic evaluation of apoptotic pathway gene polymorphisms and lung cancer risk. *Carcinogenesis* **33**, 1699-1706 (2012).
506. Lin J, *et al.* CYP1A1 Ile462Val polymorphism and the risk of non-small cell lung cancer in a Chinese population. *Tumori* **100**, 547-552 (2014).
507. Lin IH, *et al.* Smoking, green tea consumption, genetic polymorphisms in the insulin-like growth factors and lung cancer risk. *PLoS One* **7**, e30951 (2012).
508. Lim YJ, *et al.* Epidermal growth factor gene polymorphism is different between schizophrenia and lung cancer patients in Korean population. *Neurosci Lett* **374**, 157-160 (2005).
509. Lim WY, *et al.* Female reproductive factors, gene polymorphisms in the estrogen metabolism pathway, and risk of lung cancer in Chinese women. *Am J Epidemiol* **175**, 492-503 (2012).
510. Lim WY, *et al.* Polymorphisms in inflammatory pathway genes, host factors and lung cancer risk in Chinese female never-smokers. *Carcinogenesis* **32**, 522-529 (2011).
511. Liloglou T, Walters M, Maloney P, Youngson J, Field JK. A T2517C polymorphism in the GSTM4 gene is associated with risk of developing lung cancer. *Lung Cancer* **37**, 143-146 (2002).
512. Liang J, *et al.* Vascular endothelial growth factor polymorphisms and risk of lung cancer. *Chinese-German Journal of Clinical Oncology* **8**, 269-272 (2009).
513. Liang J, *et al.* Relationship between gene polymorphisms of two cytokine genes (TNF-alpha and IL-6) and occurring of lung cancers in the ethnic group Han of China. *Mol Biol Rep* **40**, 1541-1546 (2013).
514. Liang G, *et al.* Sequence variations in the DNA repair gene XPD and risk of lung cancer in a Chinese population. *Int J Cancer* **105**, 669-673 (2003).
515. Liang G, Pu Y, Yin L. Rapid detection of single nucleotide polymorphisms related with lung cancer susceptibility of Chinese population. *Cancer Lett* **223**, 265-274 (2005).
516. Liang G, Miao X, Zhou Y, Tan W, Lin D. A functional polymorphism in the SULT1A1 gene (G638A) is associated with risk of lung cancer in relation to tobacco smoking. *Carcinogenesis* **25**, 773-778 (2004).
517. Li Z, *et al.* Genetic polymorphism of DNA base-excision repair genes (APE1, OGG1 and XRCC1) and their correlation with risk of lung cancer in a Chinese population. *Arch Med Res* **42**, 226-234 (2011).
518. Li Z, Bao S, Xu X, Bao Y, Zhang Y. Polymorphisms of CHRNA5-CHRNA3-CHRNB4 gene cluster and NSCLC risk in Chinese population. *Transl Oncol* **5**, 448-452 (2012).
519. Li Y, *et al.* Genetic polymorphism in the 3'-untranslated region of the E-cadherin gene is associated with risk of different cancers. *Mol Carcinog* **50**, 857-862 (2011).
520. Li Y, *et al.* Cystic fibrosis transmembrane conductance regulator gene mutation

and lung cancer risk. *Lung Cancer* **70**, 14-21 (2010).
521. Li Y, *et al.* Correlation of polymorphisms of the vascular endothelial growth factor gene and the risk of lung cancer in an ethnic han group of North China. *Exp Ther Med* **3**, 673-676 (2012).
522. Li Y, *et al.* Polymorphisms of CAK genes and risk for lung cancer: a case-control study in Chinese population. *Lung Cancer* **58**, 171-183 (2007).
523. Li Y, *et al.* The role of leptin receptor gene polymorphisms in determining the susceptibility and prognosis of NSCLC in Chinese patients. *J Cancer Res Clin Oncol* **138**, 311-316 (2012).
524. Li Y, *et al.* TP53 genetic polymorphisms, interactions with lifestyle factors and lung cancer risk: a case control study in a Chinese population. *BMC Cancer* **13**, 607 (2013).
525. Li X, Zhang J, Su C, Zhao X, Tang L, Zhou C. The association between polymorphisms in the DNA nucleotide excision repair genes and RRM1 gene and lung cancer risk. *Thoracic Cancer* **3**, 239-248 (2012).
526. Li X, Yang XX, Hu NY, Sun JZ, Li FX, Li M. A risk-associated single nucleotide polymorphism of SMAD7 is common to colorectal, gastric, and lung cancers in a Han Chinese population. *Mol Biol Rep* **38**, 5093-5097 (2011).
527. Li X, *et al.* The interaction of APEX1 variant with polycyclic aromatic hydrocarbons on increasing chromosome damage and lung cancer risk among male Chinese. *Mol Carcinog*, (2014).
528. Li X, *et al.* Interleukin-4 -590T/C polymorphism influences the susceptibility to nonsmall cell lung cancer. *DNA Cell Biol* **31**, 797-800 (2012).
529. Li W, *et al.* Polymorphisms in GSTM1, CYP1A1, CYP2E1, and CYP2D6 are associated with susceptibility and chemotherapy response in non-small-cell lung cancer patients. *Lung* **190**, 91-98 (2012).
530. Li M, *et al.* XRCC1 polymorphisms, cooking oil fume and lung cancer in Chinese women nonsmokers. *Lung Cancer* **62**, 145-151 (2008).
531. Li Lian X, *et al.* The NAD(P)H:quinone oxidoreductase 1 gene polymorphism and lung cancer: Differential susceptibility based on smoking behavior. *Cancer Epidemiol Biomarkers Prev* **10**, 303-309 (2001).
532. Li G, Zhai X, Zhang Z, Chamberlain RM, Spitz MR, Wei Q. MDM2 gene promoter polymorphisms and risk of lung cancer: a case-control analysis. *Carcinogenesis* **27**, 2028-2033 (2006).
533. Li G, Wang LE, Chamberlain RM, Amos CI, Spitz MR, Wei Q. p73 G4C14-to-A4T14 polymorphism and risk of lung cancer. *Cancer Res* **64**, 6863-6866 (2004).
534. Li C, Yin Z, Wu W, Li X, Ren Y, Zhou B. Genetic variations in TERT-CLPTM1L genes and risk of lung cancer in Chinese women nonsmokers. *PLoS One* **8**, e64988 (2013).
535. Lewis SJ, Cherry NM, Niven RM, Barber PV, Povey AC. GSTM1, GSTT1 and GSTP1 polymorphisms and lung cancer risk. *Cancer Lett* **180**, 165-171 (2002).
536. Lewis SJ, Cherry NM, Niven RM, Barber PV, Povey AC. Polymorphisms in the NAD(P)H: quinone oxidoreductase gene and small cell lung cancer risk in a UK population. *Lung Cancer* **34**, 177-183 (2001).
537. Leng S, *et al.* Defining a gene promoter methylation signature in sputum for lung cancer risk assessment. *Clin Cancer Res* **18**, 3387-3395 (2012).
538. Leng S, *et al.* Discovery of common SNPs in the miR-205/200 familyregulatedepithelial to mesenchymal transition pathway and their association with risk for non-small cell lung cancer. *Int J Mol Epidemiol Genet* **2**,

145-155 (2011).
539. Lener MR, *et al.* Prevalence of the NOD2 3020insC mutation in aggregations of breast and lung cancer. *Breast Cancer Res Treat* **95**, 141-145 (2006).
540. Lei Z, *et al.* TGFBR1 haplotypes and risk of non-small-cell lung cancer. *Cancer Res* **69**, 7046-7052 (2009).
541. Legrand-Andreoletti M, *et al.* Cytochrome P450 CYP2D6 gene polymorphism and lung cancer susceptibility in Caucasians. *Pharmacogenetics* **8**, 7-14 (1998).
542. Legrand M, Stucker I, Marez D, Sabbagh N, Lo-Guidice JM, Broly F. Influence of a mutation reducing the catalytic activity of the cytochrome P450 CYP2D6 on lung cancer susceptibility. *Carcinogenesis* **17**, 2267-2269 (1996).
543. Lee YL, *et al.* Association of genetic polymorphisms of CXCL12/SDF1 gene and its receptor, CXCR4, to the susceptibility and prognosis of non-small cell lung cancer. *Lung Cancer* **73**, 147-152 (2011).
544. Lee YC, *et al.* A case-control study of the association of the polymorphisms and haplotypes of DNA ligase I with lung and upper-aerodigestive-tract cancers. *Int J Cancer* **122**, 1630-1638 (2008).
545. Lee WK, *et al.* Polymorphisms in the Caspase7 gene and the risk of lung cancer. *Lung Cancer* **65**, 19-24 (2009).
546. Lee SY, *et al.* Polymorphisms in the caspase genes and the risk of lung cancer. *J Thorac Oncol* **5**, 1152-1158 (2010).
547. Lee SJ, *et al.* Vascular endothelial growth factor gene polymorphisms and risk of primary lung cancer. *Cancer Epidemiol Biomarkers Prev* **14**, 571-575 (2005).
548. Lee SJ, *et al.* DNMT3B polymorphisms and risk of primary lung cancer. *Carcinogenesis* **26**, 403-409 (2005).
549. Lee KM, *et al.* Polymorphisms in immunoregulatory genes, smoky coal exposure and lung cancer risk in Xuan Wei, China. *Carcinogenesis* **28**, 1437-1441 (2007).
550. Lee KM, *et al.* Interactive effect of genetic polymorphism of glutathione S-transferase M1 and smoking on squamous cell lung cancer risk in Korea. *Oncol Rep* **16**, 1035-1039 (2006).
551. Lee JA, *et al.* Association of low activity of UGT1A7 with Lung Cancer in Taiwan: A preliminary case control study. *Journal of Food and Drug Analysis* **19**, 403-409+538 (2011).
552. Lee J, Dahl M, Nordestgaard BG. Genetically lowered microsomal epoxide hydrolase activity and tobacco-related cancer in 47,000 individuals. *Cancer Epidemiol Biomarkers Prev* **20**, 1673-1682 (2011).
553. Lee GY, *et al.* XPC polymorphisms and lung cancer risk. *Int J Cancer* **115**, 807-813 (2005).
554. Lee CN, *et al.* NAT2 fast acetylator genotypes are associated with an increased risk for lung cancer with wildtype epidermal growth factor receptors in Taiwan. *Lung Cancer* **64**, 9-12 (2009).
555. Le Marchand L, *et al.* Associations of CYP1A1, GSTM1, and CYP2E1 polymorphisms with lung cancer suggest cell type specificities to tobacco carcinogens. *Cancer Res* **58**, 4858-4863 (1998).
556. Le Marchand L, Seifried A, Lum A, Wilkens LR. Association of the myeloperoxidase -463G-->a polymorphism with lung cancer risk. *Cancer Epidemiol Biomarkers Prev* **9**, 181-184 (2000).
557. Le Marchand L, Donlon T, Lum-Jones A, Seifried A, Wilkens LR. Association of the hOGG1 Ser326Cys polymorphism with lung cancer risk. *Cancer Epidemiol Biomarkers Prev* **11**, 409-412 (2002).
558. Lawson KA, Woodson K, Virtamo J, Albanes D. Association of the

NAD(P)H:quinone oxidoreductase (NQO1) 609C->T polymorphism with lung cancer risk among male smokers. *Cancer Epidemiol Biomarkers Prev* **14**, 2275-2276 (2005).
559. Larsen JE, Colosimo ML, Yang IA, Bowman R, Zimmerman PV, Fong KM. CYP1A1 Ile462Val and MPO G-463A interact to increase risk of adenocarcinoma but not squamous cell carcinoma of the lung. *Carcinogenesis* **27**, 525-532 (2006).
560. Larsen JE, Colosimo ML, Yang IA, Bowman R, Zimmerman PV, Fong KM. Risk of non-small cell lung cancer and the cytochrome P4501A1 Ile462Val polymorphism. *Cancer Causes Control* **16**, 579-585 (2005).
561. Landvik NE, Hart K, Skaug V, Stangeland LB, Haugen A, Zienolddiny S. A specific interleukin-1B haplotype correlates with high levels of IL1B mRNA in the lung and increased risk of non-small cell lung cancer. *Carcinogenesis* **30**, 1186-1192 (2009).
562. Landi S, *et al.* DNA repair and cell cycle control genes and the risk of young-onset lung cancer. *Cancer Res* **66**, 11062-11069 (2006).
563. Lan Q, *et al.* Smoky coal exposure, NBS1 polymorphisms, p53 protein accumulation, and lung cancer risk in Xuan Wei, China. *Lung Cancer* **49**, 317-323 (2005).
564. Lan Q, *et al.* Oxidative damage-related genes AKR1C3 and 0GG1 modulate risks for lung cancer due to exposure to PAH-rich coal combustion emissions. *Carcinogenesis* **25**, 2177-2181 (2004).
565. Lan Q, He X. Molecular epidemiological studies on the relationship between indoor coal burning and lung cancer in Xuan Wei, China. *Toxicology* **198**, 301-305 (2004).
566. Lan Q, *et al.* Longer telomere length in peripheral white blood cells is associated with risk of lung cancer and the rs2736100 (CLPTM1L-TERT) polymorphism in a prospective cohort study among women in China. *PLoS One* **8**, e59230 (2013).
567. Lam TK, *et al.* Copy number variants of GSTM1 and GSTT1 in relation to lung cancer risk in a prospective cohort study. *Ann Epidemiol* **19**, 546-552 (2009).
568. Laforest L, *et al.* CYP2D6 gene polymorphism in caucasian smokers: lung cancer susceptibility and phenotype-genotype relationships. *Eur J Cancer* **36**, 1825-1832 (2000).
569. Kuo WH, *et al.* Association of hypoxia inducible factor-1alpha polymorphisms with susceptibility to non-small-cell lung cancer. *Transl Res* **159**, 42-50 (2012).
570. Kumimoto H, *et al.* L-myc genotype is associated with different susceptibility to lung cancer in smokers. *Jpn J Cancer Res* **93**, 1-5 (2002).
571. Kumar M, Agarwal SK, Goel SK. Lung cancer risk in north Indian population: role of genetic polymorphisms and smoking. *Mol Cell Biochem* **322**, 73-79 (2009).
572. Kua LF, *et al.* UGT1A6 polymorphisms modulated lung cancer risk in a Chinese population. *PLoS One* **7**, e42873 (2012).
573. Krzesniak M, Butkiewicz D, Samojedny A, Chorazy M, Rusin M. Polymorphisms in TDG and MGMT genes - epidemiological and functional study in lung cancer patients from Poland. *Ann Hum Genet* **68**, 300-312 (2004).
574. Krontiris TG, Devlin B, Karp DD, Robert NJ, Risch N. An association between the risk of cancer and mutations in the HRAS1 minisatellite locus. *N Engl J Med* **329**, 517-523 (1993).
575. Korosec B, Glavac D, Volavsek M, Ravnik-Glavac M. ATP2A3 gene is involved in cancer susceptibility. *Cancer Genet Cytogenet* **188**, 88-94 (2009).

576. Konac E, *et al.* Genetic variations in the hypoxia-inducible factor-1alpha gene and lung cancer. *Exp Biol Med (Maywood)* **234**, 1109-1116 (2009).
577. Kohno T, *et al.* Association of polymorphisms in the MTH1 gene with small cell lung carcinoma risk. *Carcinogenesis* **27**, 2448-2454 (2006).
578. Kohno T, *et al.* Association of the OGG1-Ser326Cys polymorphism with lung adenocarcinoma risk. *Cancer Sci* **97**, 724-728 (2006).
579. Kohno T, *et al.* Association of KRAS polymorphisms with risk for lung adenocarcinoma accompanied by atypical adenomatous hyperplasias. *Carcinogenesis* **29**, 957-963 (2008).
580. Kohno T, *et al.* Contribution of the TP53, OGG1, CHRNA3, and HLA-DQA1 genes to the risk for lung squamous cell carcinoma. *J Thorac Oncol* **6**, 813-817 (2011).
581. Kohno T, *et al.* Association of CYP19A1 polymorphisms with risks for atypical adenomatous hyperplasia and bronchioloalveolar carcinoma in the lungs. *Carcinogenesis* **31**, 1794-1799 (2010).
582. Klinchid J, Chewaskulyoung B, Saeteng S, Lertprasertsuke N, Kasinrerk W, Cressey R. Effect of combined genetic polymorphisms on lung cancer risk in northern Thai women. *Cancer Genet Cytogenet* **195**, 143-149 (2009).
583. Kiyohara C, Wakai K, Mikami H, Sido K, Ando M, Ohno Y. Risk modification by CYP1A1 and GSTM1 polymorphisms in the association of environmental tobacco smoke and lung cancer: a case-control study in Japanese nonsmoking women. *Int J Cancer* **107**, 139-144 (2003).
584. Kiyohara C, Takayama K, Nakanishi Y. CYP2A13, CYP2A6, and the risk of lung adenocarcinoma in a Japanese population. *Journal of Health Science* **51**, 658-666 (2005).
585. Kiyohara C, Horiuchi T, Takayama K, Nakanishi Y. Genetic polymorphisms involved in the inflammatory response and lung cancer risk: a case-control study in Japan. *Cytokine* **65**, 88-94 (2014).
586. Kiyohara C, Horiuchi T, Takayama K, Nakanishi Y. Genetic polymorphisms involved in carcinogen metabolism and DNA repair and lung cancer risk in a Japanese population. *J Thorac Oncol* **7**, 954-962 (2012).
587. Kiyohara C, Horiuchi T, Takayama K, Nakanishi Y. Methylenetetrahydrofolate reductase polymorphisms and interaction with smoking and alcohol consumption in lung cancer risk: a case-control study in a Japanese population. *BMC Cancer* **11**, 459 (2011).
588. Kiyohara C, Horiuchi T, Takayama K, Nakanishi Y. IL1B rs1143634 polymorphism, cigarette smoking, alcohol use, and lung cancer risk in a Japanese population. *J Thorac Oncol* **5**, 299-304 (2010).
589. Kiyohara C, Horiuchi T, Miyake Y, Takayama K, Nakanishi Y. Cigarette smoking, TP53 Arg72Pro, TP53BP1 Asp353Glu and the risk of lung cancer in a Japanese population. *Oncol Rep* **23**, 1361-1368 (2010).
590. Kim MJ, Yoo SS, Choi YY, Park JY. A functional polymorphism in the pre-microRNA-196a2 and the risk of lung cancer in a Korean population. *Lung Cancer* **69**, 127-129 (2010).
591. Kim JS, *et al.* Association of a common AGO1 variant with lung cancer risk: a two-stage case-control study. *Mol Carcinog* **49**, 913-921 (2010).
592. Kim JH, *et al.* Aryl hydrocarbon receptor gene polymorphisms affect lung cancer risk. *Lung Cancer* **56**, 9-15 (2007).
593. Kim JH, *et al.* Genetic polymorphisms of ataxia telangiectasia mutated affect lung cancer risk. *Hum Mol Genet* **15**, 1181-1186 (2006).

594. Kim IS, *et al.* Polymorphisms and haplotypes in the XRCC1 gene and the risk of advanced non-small cell lung cancer. *J Thorac Oncol* **5**, 1912-1921 (2010).
595. Kim HK, *et al.* A study of RET proto-oncogene polymorphisms in association with lung cancer risk in the Korean population. *Anticancer Res* **30**, 3621-3627 (2010).
596. Kim DH, *et al.* A functional single nucleotide polymorphism at the promoter region of cyclin A2 is associated with increased risk of colon, liver, and lung cancers. *Cancer* **117**, 4080-4091 (2011).
597. Kihara M, Noda K, Kihara M. Distribution of GSTM1 null genotype in relation to gender, age and smoking status in Japanese lung cancer patients. *Pharmacogenetics* **5 Spec No**, S74-79 (1995).
598. Kihara M, Kihara M, Noda K. Lung cancer risk of the GSTM1 null genotype is enhanced in the presence of the GSTP1 mutated genotype in male Japanese smokers. *Cancer Lett* **137**, 53-60 (1999).
599. Kihara M, Kihara M, Noda K. Risk of smoking for squamous and small cell carcinomas of the lung modulated by combinations of CYP1A1 and GSTM1 gene polymorphisms in a Japanese population. *Carcinogenesis* **16**, 2331-2336 (1995).
600. Khaghanzadeh N, Erfani N, Ghayumi MA, Ghaderi A. CTLA4 gene variations and haplotypes in patients with lung cancer. *Cancer Genet Cytogenet* **196**, 171-174 (2010).
601. Kelsey KT, Wiencke JK, Spitz MR. A race-specific genetic polymorphism in the CYP1A1 gene is not associated with lung cancer in African Americans. *Carcinogenesis* **15**, 1121-1124 (1994).
602. Ke J, *et al.* Replication study in Chinese population and meta-analysis supports association of the 5p15.33 locus with lung cancer. *PLoS One* **8**, e62485 (2013).
603. Kawajiri K, Watanabe J, Eguchi H, Nakachi K, Kiyohara C, Hayashi S. Polymorphisms of human Ah receptor gene are not involved in lung cancer. *Pharmacogenetics* **5**, 151-158 (1995).
604. Kawajiri K, Nakachi K, Imai K, Watanabe J, Hayashi S. Germ line polymorphisms of p53 and CYP1A1 genes involved in human lung cancer. *Carcinogenesis* **14**, 1085-1089 (1993).
605. Kaur-Knudsen D, Bojesen SE, Tybjaerg-Hansen A, Nordestgaard BG. Nicotinic acetylcholine receptor polymorphism, smoking behavior, and tobacco-related cancer and lung and cardiovascular diseases: a cohort study. *J Clin Oncol* **29**, 2875-2882 (2011).
606. Kaur TB, Travaline JM, Gaughan JP, Richie JP, Jr., Stellman SD, Lazarus P. Role of polymorphisms in codons 143 and 160 of the O6-alkylguanine DNA alkyltransferase gene in lung cancer risk. *Cancer Epidemiol Biomarkers Prev* **9**, 339-342 (2000).
607. Katoh T, *et al.* Human glutathione S-transferase P1 polymorphism and susceptibility to smoking related epithelial cancer; oral, lung, gastric, colorectal and urothelial cancer. *Pharmacogenetics* **9**, 165-169 (1999).
608. Kato S, *et al.* Analysis of cytochrome P450 2E1 genetic polymorphisms in relation to human lung cancer. *Cancer Epidemiol Biomarkers Prev* **3**, 515-518 (1994).
609. Kato S, *et al.* Cytochrome P450IIE1 genetic polymorphisms, racial variation, and lung cancer risk. *Cancer Res* **52**, 6712-6715 (1992).
610. Katafigiotis S, *et al.* A case-control study on the rs3130932 single nucleotide polymorphism in the OCT4B translation initiation codon in association with

cancer state. *Eur J Cancer Prev* **20**, 248-251 (2011).
611. Karkucak M, *et al.* XRCC1 gene polymorphisms and risk of lung cancer in Turkish patients. *Int J Hum Genet* **12**, 113-117 (2012).
612. Karahalil B, Emerce E, Kocer B, Han S, Alkis N, Karakaya AE. The association of OGG1 Ser326Cys polymorphism and urinary 8-OHdG levels with lung cancer susceptibility: a hospital-based case-control study in Turkey. *Arh Hig Rada Toksikol* **59**, 241-250 (2008).
613. Karabon L, *et al.* CTLA-4, CD28, and ICOS gene polymorphism associations with non-small-cell lung cancer. *Hum Immunol* **72**, 947-954 (2011).
614. Kanzaki H, *et al.* Single nucleotide polymorphism of the AXIN2 gene is preferentially associated with human lung cancer risk in a Japanese population. *Int J Mol Med* **18**, 279-284 (2006).
615. Kanzaki H, *et al.* The association between RAD18 Arg302Gln polymorphism and the risk of human non-small-cell lung cancer. *J Cancer Res Clin Oncol* **134**, 211-217 (2008).
616. Kanzaki H, *et al.* Single nucleotide polymorphism at codon 133 of the RASSF1 gene is preferentially associated with human lung adenocarcinoma risk. *Cancer Lett* **238**, 128-134 (2006).
617. Kantarci OH, *et al.* Myeloperoxidase -463 (G-->A) polymorphism associated with lower risk of lung cancer. *Mayo Clin Proc* **77**, 17-22 (2002).
618. Kant Shukla R, Kant S, Mittal B, Bhattacharya S. Comparative study of GST polymorphism in relation to age in COPD and lung cancer. *Tuberkuloz ve Toraks* **61**, 275-282 (2013).
619. Kang HG, *et al.* Identification of polymorphisms in the XIAP gene and analysis of association with lung cancer risk in a Korean population. *Cancer Genet Cytogenet* **180**, 6-13 (2008).
620. Kang HG, *et al.* +61A>G polymorphism in the EGF gene does not increase the risk of lung cancer. *Respirology* **12**, 902-905 (2007).
621. Kang HG, *et al.* Polymorphisms in TGF-beta1 gene and the risk of lung cancer. *Lung Cancer* **52**, 1-7 (2006).
622. Kaabachi W, Amor AB, Kaabachi S, Rafrafi A, Tizaoui K, Hamzaoui K. Interleukin-17A and -17F genes polymorphisms in lung cancer. *Cytokine* **66**, 23-29 (2014).
623. Kaabachi S, Kaabachi W, Rafrafi A, Belkis H, Hamzaoui K, Haj Sassi F. Tumor necrosis factor gene polymorphisms in Tunisian patients with non-small cell lung cancer. *Clinical Laboratory* **59**, 1389-1395 (2013).
624. Jung HY, *et al.* Lack of association of fragile histidine triad (FHIT) polymorphisms with lung cancer in the Korean population. *J Hum Genet* **52**, 668-674 (2007).
625. Jun HJ, *et al.* Combined effects of p73 and MDM2 polymorphisms on the risk of lung cancer. *Mol Carcinog* **46**, 100-105 (2007).
626. Jourenkova-Mironova N, *et al.* Role of glutathione S-transferase GSTM1, GSTM3, GSTP1 and GSTT1 genotypes in modulating susceptibility to smoking-related lung cancer. *Pharmacogenetics* **8**, 495-502 (1998).
627. Jou YS, *et al.* Association of an EGFR intron 1 SNP with never-smoking female lung adenocarcinoma patients. *Lung Cancer* **64**, 251-256 (2009).
628. Jones NR, Spratt TE, Berg AS, Muscat JE, Lazarus P, Gallagher CJ. Association studies of excision repair cross-complementation group 1 (ERCC1) haplotypes with lung and head and neck cancer risk in a Caucasian population. *Cancer Epidemiol* **35**, 175-181 (2011).

629. Jo U, *et al.* The genetic polymorphisms of HER-2 and the risk of lung cancer in a Korean population. *BMC Cancer* **8**, (2008).
630. Jin Y, *et al.* Combined effects of serum trace metals and polymorphisms of CYP1A1 or GSTM1 on non-small cell lung cancer: a hospital based case-control study in China. *Cancer Epidemiol* **35**, 182-187 (2011).
631. Jin Y, *et al.* Combined effects of cigarette smoking, gene polymorphisms and methylations of tumor suppressor genes on non small cell lung cancer: a hospital-based case-control study in China. *BMC Cancer* **10**, 422 (2010).
632. Jin X, *et al.* Higher lung cancer risk for younger African-Americans with the Pro/Pro p53 genotype. *Carcinogenesis* **16**, 2205-2208 (1995).
633. Jin G, *et al.* Common genetic variants on 5p15.33 contribute to risk of lung adenocarcinoma in a Chinese population. *Carcinogenesis* **30**, 987-990 (2009).
634. Jin G, *et al.* Potentially functional polymorphisms of EXO1 and risk of lung cancer in a Chinese population: A case-control analysis. *Lung Cancer* **60**, 340-346 (2008).
635. Jiang Y, *et al.* Functional regulatory variants of MCL1 contribute to enhanced promoter activity and reduced risk of lung cancer in nonsmokers: implications for context-dependent phenotype of an antiapoptotic and antiproliferative gene in solid tumor. *Cancer* **118**, 2085-2095 (2012).
636. Jiang M, Wu H, Qin C. Genetic variant rs401681 at 5p15.33 modifies susceptibility to lung cancer but not esophageal squamous cell carcinoma. *PLoS One* **8**, e84277 (2013).
637. Jiang B, *et al.* STAT3 gene polymorphisms and susceptibility to non-small cell lung cancer. *Genet Mol Res* **10**, 1856-1865 (2011).
638. Jia Y, *et al.* MicroRNA-146a rs2910164 polymorphism is associated with susceptibility to non-small cell lung cancer in the Chinese population. *Med Oncol* **31**, (2014).
639. Ahn MJ, *et al.* A single nucleotide polymorphism in the phospholipase D1 gene is associated with risk of non-small cell lung cancer. *Int J Biomed Sci* **8**, 121-128 (2012).
640. Ahn MJ, *et al.* The 18p11.22 locus is associated with never smoker non-small cell lung cancer susceptibility in Korean populations. *Hum Genet* **131**, 365-372 (2012).
641. Aksoy-Sagirli P, *et al.* Paraoxonase-1 192/55 polymorphisms and the risk of lung cancer in a Turkish population. *Anticancer Res* **31**, 2225-2229 (2011).
642. Aldrich MC, *et al.* CYP1A1/2 haplotypes and lung cancer and assessment of confounding by population stratification. *Cancer Res* **69**, 2340-2348 (2009).
643. Alexandrie AK, Nyberg F, Warholm M, Rannug A. Influence of CYP1A1, GSTM1, GSTT1, and NQO1 genotypes and cumulative smoking dose on lung cancer risk in a Swedish population. *Cancer Epidemiol Biomarkers Prev* **13**, 908-914 (2004).
644. Alexandrie AK, Sundberg MI, Seidegard J, Tornling G, Rannug A. Genetic susceptibility to lung cancer with special emphasis on CYP1A1 and GSTM1: a study on host factors in relation to age at onset, gender and histological cancer types. *Carcinogenesis* **15**, 1785-1790 (1994).
645. Al-Tassan N, *et al.* Inherited variants in MYH are unlikely to contribute to the risk of lung carcinoma. *Hum Genet* **114**, 207-210 (2004).
646. Altinisik J, Balta ZB, Aydin G, Ulutin T, Buyru N. Investigation of glutathione S-transferase M1 and T1 deletions in lung cancer. *Mol Biol Rep* **37**, 263-267 (2010).

647. Amos CI, *et al.* Nicotinic acetylcholine receptor region on chromosome 15q25 and lung cancer risk among African Americans: a case-control study. *J Natl Cancer Inst* **102**, 1199-1205 (2010).
648. An S, *et al.* Mutation and polymorphism in the tyrosine kinase domain of KDR in Chinese human lung cancer patients. *Chinese-German Journal of Clinical Oncology* **8**, 309-313 (2009).
649. An SJ, *et al.* The -271 G>A polymorphism of kinase insert domain-containing receptor gene regulates its transcription level in patients with non-small cell lung cancer. *BMC Cancer* **9**, 144 (2009).
650. An Y, *et al.* Polymorphisms in hMLH1 and risk of early-onset lung cancer in a southeast Chinese population. *Lung Cancer* **59**, 164-170 (2008).
651. Antczak A, *et al.* Ctla-4 expression and polymorphisms in lung tissue of patients with diagnosed non-small-cell lung cancer. *Biomed Res Int* **2013**, 576486 (2013).
652. Araki J, *et al.* Polymorphism of UDP-glucuronosyltransferase 1A7 gene: a possible new risk factor for lung cancer. *Eur J Cancer* **41**, 2360-2365 (2005).
653. Arslan S, Karadayi S, Yildirim ME, Ozdemir O, Akkurt I. The association between methylene-tetrahydrofolate reductase gene polymorphism and lung cancer risk. *Mol Biol Rep* **38**, 991-996 (2011).
654. Arslan S, Pinarbasi H, Silig Y. Myeloperoxidase G-463A polymorphism and risk of lung and prostate cancer in a Turkish population. *Mol Med Rep* **4**, 87-92 (2011).
655. Arslan S, Silig Y, Pinarbasi H. An investigation of the relationship between SULT1A1 Arg(213)His polymorphism and lung cancer susceptibility in a Turkish population. *Cell Biochem Funct* **27**, 211-215 (2009).
656. Asada M, *et al.* Interleukin-1beta gene polymorphisms associated with risk of lung cancer in Japanese. *Lung Cancer* **54**, 261-263 (2006).
657. Atinkaya C, *et al.* The effect of CYP1A1, GSTT1 and GSTM1 polymorphisms on the risk of lung cancer: a case-control study. *Hum Exp Toxicol* **31**, 1074-1080 (2012).
658. Aynaci E, *et al.* Association between survivin gene promoter -31G/C and -644C/T polymorphisms and non-small cell lung cancer. *Genet Mol Res* **12**, 3975-3982 (2013).
659. Aysegul B, Veysi GH, Muzaffer M, Irfan D, Azra A, Hulyam K. Is a single nucleotide polymorphism a risk factor for lung cancer in the matrix metalloproteinase-2 promoter? *Mol Biol Rep* **38**, 1469-1474 (2011).
660. Bae EY, *et al.* Replication of results of genome-wide association studies on lung cancer susceptibility loci in a Korean population. *Respirology* **17**, 699-706 (2012).
661. Bai J, Li X, Tong D, Shi W, Song H, Li Q. T-cell immunoglobulin- and mucin-domain-containing molecule 3 gene polymorphisms and prognosis of non-small-cell lung cancer. *Tumour Biol* **34**, 805-809 (2013).
662. Bai L, Yu H, Wang H, Su H, Zhao J, Zhao Y. Genetic single-nucleotide polymorphisms of inflammation-related factors associated with risk of lung cancer. *Med Oncol* **30**, 414 (2013).
663. Bai Y, *et al.* Sequence variations in DNA repair gene XPC is associated with lung cancer risk in a Chinese population: a case-control study. *BMC Cancer* **7**, 81 (2007).
664. Bao WL, Shi H, Zhang AQ, Kong XM, Deng DH, Zhang YJ. Lack of associations of polymorphisms of IL-7R, IL-13 and IL-15 with NSCLCs in non-smoking Chinese. *Asian Pac J Cancer Prev* **12**, 3239-3244 (2011).

665. Bayramoglu A, *et al.* Plasminogen activator inhibitor-1 and susceptibility to lung cancer: A population genetics perspective. *Genet Test Mol Biomarkers* **18**, 587-590 (2014).
666. Bayramoglu A, Gunes HV, Metintas M, Degirmenci I, Mutlu F, Alatas F. The association of MMP-9 enzyme activity, MMP-9 C1562T polymorphism, and MMP-2 and -9 and TIMP-1, -2, -3, and -4 gene expression in lung cancer. *Genet Test Mol Biomarkers* **13**, 671-678 (2009).
667. B'Chir F, Pavanello S, Knani J, Boughattas S, Arnaud MJ, Saguem S. CYP1A2 genetic polymorphisms and adenocarcinoma lung cancer risk in the Tunisian population. *Life Sci* **84**, 779-784 (2009).
668. Belogubova EV, *et al.* 'Comparison of extremes' approach provides evidence against the modifying role of NAT2 polymorphism in lung cancer susceptibility. *Cancer Lett* **221**, 177-183 (2005).
669. Belogubova EV, *et al.* A novel approach for assessment of cancer predisposing roles of GSTM1 and GSTT1 genes: use of putatively cancer resistant elderly tumor-free smokers as the referents. *Lung Cancer* **43**, 259-266 (2004).
670. Belogubova EV, *et al.* Combined CYP1A1/GSTM1 at-risk genotypes are overrepresented in squamous cell lung carcinoma patients but underrepresented in elderly tumor-free subjects. *J Cancer Res Clin Oncol* **132**, 327-331 (2006).
671. Benhamou S, Reinikainen M, Bouchardy C, Dayer P, Hirvonen A. Association between lung cancer and microsomal epoxide hydrolase genotypes. *Cancer Res* **58**, 5291-5293 (1998).
672. Benhamou S, Voho A, Bouchardy C, Mitrunen K, Dayer P, Hirvonen A. Role of NAD(P)H:quinone oxidoreductase polymorphism at codon 187 in susceptibility to lung, laryngeal and oral/pharyngeal cancers. *Biomarkers* **6**, 440-447 (2001).
673. Bhat IA, *et al.* Association of interleukin 1 beta (IL-1(beta)) polymorphism with mRNA expression and risk of non small cell lung cancer. *Meta Gene* **2**, 123-133 (2014).
674. Bhat IA, *et al.* Lack of association of a common polymorphism in the 3' -UTR of interleukin 8 with non small cell lung cancer in Kashmir. *Asian Pac J Cancer Prev* **14**, 4403-4408 (2013).
675. Bhat IA, *et al.* COX-2 overexpression and -8473 T/C polymorphism in 3' UTR in non-small cell lung cancer. *Tumour Biol*, (2014).
676. Bianchino G, *et al.* Polymorphisms of the CYP1A1, CYP2E1 and XRCC1 genes and cancer risk in a Southern Italian population: A case-control study. *Anticancer Res* **31**, 1359-1365 (2011).
677. Biondi ML, *et al.* MMP1 and MMP3 polymorphisms in promoter regions and cancer. *Clin Chem* **46**, 2023-2024 (2000).
678. Birgander R, *et al.* P53 polymorphisms and haplotypes in lung cancer. *Carcinogenesis* **16**, 2233-2236 (1995).
679. Biros E, *et al.* Polymorphism of the p53 gene within the codon 72 in lung cancer patients. *Neoplasma* **48**, 407-411 (2001).
680. Biros E, Kalina I, Kohut A, Stubna J, Salagovic J. Germ line polymorphisms of the tumor suppressor gene p53 and lung cancer. *Lung Cancer* **31**, 157-162 (2001).
681. Bo W, *et al.* A newfound association between MDC1 functional polymorphism and lung cancer risk in Chinese. *PLoS One* **9**, (2014).
682. Bouchardy C, *et al.* N-acetyltransferase NAT1 and NAT2 genotypes and lung cancer risk. *Pharmacogenetics* **8**, 291-298 (1998).
683. Bowman ED. Chromosome 13 poly(ADP-ribose) polymerase polymorphisms

and lung cancer risk. *Oncol Rep* **4**, 269-272 (1997).
684. Brockmoller J, Kerb R, Drakoulis N, Nitz M, Roots I. Genotype and phenotype of glutathione S-transferase class mu isoenzymes mu and psi in lung cancer patients and controls. *Cancer Res* **53**, 1004-1011 (1993).
685. Buch SC, *et al.* Genetic variability in DNA repair and cell cycle control pathway genes and risk of smoking-related lung cancer. *Mol Carcinog* **51 Suppl 1**, E11-20 (2012).
686. Butkiewicz D, *et al.* Association between the risk for lung adenocarcinoma and a (-4) G-to-A polymorphism in the XPA gene. *Cancer Epidemiol Biomarkers Prev* **13**, 2242-2246 (2004).
687. Butkiewicz D, Rusin M, Enewold L, Shields PG, Chorazy M, Harris CC. Genetic polymorphisms in DNA repair genes and risk of lung cancer. *Carcinogenesis* **22**, 593-597 (2001).
688. Buyru N, Altinisik J, Isin M, Dalay N. p53 codon 72 polymorphism and HPV status in lung cancer. *Med Sci Monit* **14**, Cr493-497 (2008).
689. Cabral RE, Caldeira-de-Araujo A, Cabral-Neto JB, Costa Carvalho Mda G. Analysis of GSTM1 and GSTT1 polymorphisms in circulating plasma DNA of lung cancer patients. *Mol Cell Biochem* **338**, 263-269 (2010).
690. Caceres DD, Quinones LA, Schroeder JC, Gil LD, Irarrazabal CE. Association between p53 codon 72 genetic polymorphism and tobacco use and lung cancer risk. *Lung* **187**, 110-115 (2009).
691. Cajas-Salazar N, *et al.* Effect of epoxide hydrolase polymorphisms on chromosome aberrations and risk for lung cancer. *Cancer Genet Cytogenet* **145**, 97-102 (2003).
692. Cajas-Salazar N, Sierra-Torres CH, Salama SA, Zwischenberger JB, Au WW. Combined effect of MP0, GSTM1 and GSTT1 polymorphisms on chromosome aberrations and lung cancer risk. *Int J Hyg Environ Health* **206**, 473-483 (2003).
693. Cakina S, Gulyasar T, Ozen A, Sipahi T, Kocak Z, Sener S. Relationship between cyclin D1 (A870G) gene polymorphism and lung cancer. *Indian J Biochem Biophys* **50**, 233-236 (2013).
694. Campa D, *et al.* Lack of association between polymorphisms in inflammatory genes and lung cancer risk. *Cancer Epidemiol Biomarkers Prev* **14**, 538-539 (2005).
695. Campa D, *et al.* Lack of association between -251 T>A polymorphism of IL8 and lung cancer risk. *Cancer Epidemiol Biomarkers Prev* **14**, 2457-2458 (2005).
696. Campa D, *et al.* Polymorphisms of dopamine receptor/transporter genes and risk of non-small cell lung cancer. *Lung Cancer* **56**, 17-23 (2007).
697. Campa D, Zienolddiny S, Maggini V, Skaug V, Haugen A, Canzian F. Association of a common polymorphism in the cyclooxygenase 2 gene with risk of non-small cell lung cancer. *Carcinogenesis* **25**, 229-235 (2004).
698. Cantlay AM, *et al.* Association between the CYP1A1 gene polymorphism and susceptibility to emphysema and lung cancer. *Clin Mol Pathol* **48**, M210-M214 (1995).
699. Cao B, *et al.* Lack of association between the unique LMP2 gene polymorphism and the outcome of lung cancer in a population of Chinese Han nationality. *Hum Immunol* **73**, 580-584 (2012).
700. Cao G, Lu H, Feng J, Shu J, Zheng D, Hou Y. Lung cancer risk associated with thr495pro polymorphism of GHR in chines population. *Jpn J Clin Oncol* **38**, 308-316 (2008).
701. Cao L, *et al.* A regulatory variant in CYP2E1 affects the risk of lung squamous

cell carcinoma. *Tumour Biol* **35**, 455-462 (2014).
702. Carpenter CL, Yu MC, London SJ. Dietary isothiocyanates, glutathione S-transferase M1 (GSTM1), and lung cancer risk in African Americans and Caucasians from Los Angeles County, California. *Nutr Cancer* **61**, 492-499 (2009).
703. Cascorbi I, Brockmoller J, Mrozikiewicz PM, Bauer S, Loddenkemper R, Roots I. Homozygous rapid arylamine N-acetyltransferase (NAT2) genotype as a susceptibility factor for lung cancer. *Cancer Res* **56**, 3961-3966 (1996).
704. Cascorbi I, Brockmoller J, Roots I. A C4887A polymorphism in exon 7 of human CYP1A1: population frequency, mutation linkages, and impact on lung cancer susceptibility. *Cancer Res* **56**, 4965-4969 (1996).
705. Catarino R, *et al.* Cyclin D1 polymorphism in non-small cell lung cancer in a Portuguese population. *Cancer Biomark* **12**, 65-72 (2012).
706. Cauchi S, Stucker I, Cenee S, Kremers P, Beaune P, Massaad-Massade L. Structure and polymorphisms of human aryl hydrocarbon receptor repressor (AhRR) gene in a French population: relationship with CYP1A1 inducibility and lung cancer. *Pharmacogenetics* **13**, 339-347 (2003).
707. Cauchi S, *et al.* Polymorphisms of human aryl hydrocarbon receptor (AhR) gene in a French population: relationship with CYP1A1 inducibility and lung cancer. *Carcinogenesis* **22**, 1819-1824 (2001).
708. Cauffiez C, *et al.* Genetic polymorphism of the human cytochrome CYP2A13 in a French population: implication in lung cancer susceptibility. *Biochem Biophys Res Commun* **317**, 662-669 (2004).
709. Chae MH, *et al.* O6-alkylguanine-DNA alkyltransferase gene polymorphisms and the risk of primary lung cancer. *Mol Carcinog* **45**, 239-249 (2006).
710. Chan EC, Lam SY, Fu KH, Kwong YL. Polymorphisms of the GSTM1, GSTP1, MPO, XRCC1, and NQO1 genes in Chinese patients with non-small cell lung cancers: relationship with aberrant promoter methylation of the CDKN2A and RARB genes. *Cancer Genet Cytogenet* **162**, 10-20 (2005).
711. Chang CH, *et al.* Interactive effect of cigarette smoking with human 8-oxoguanine DNA N-glycosylase 1 (hOGG1) polymorphisms on the risk of lung cancer: a case-control study in Taiwan. *Am J Epidemiol* **170**, 695-702 (2009).
712. Chang FH, *et al.* Genetic polymorphism of T6235C mutation in 3' non-coding region of CYP1A1 and GSTM1 genes and lung cancer susceptibility in the Mongolian population. *Acad J Xian Jiaotong Univ* **21**, 225-229 (2009).
713. Chang HL, *et al.* Association of estrogen receptor (alpha) gene Pvuii and Xbai polymorphisms with non-small cell lung cancer. *Oncol Lett* **3**, 462-468 (2012).
714. Chang JS, *et al.* Nucleotide excision repair genes and risk of lung cancer among San Francisco Bay Area Latinos and African Americans. *Int J Cancer* **123**, 2095-2104 (2008).
715. Chang JS, *et al.* Base excision repair genes and risk of lung cancer among San Francisco Bay Area Latinos and African-Americans. *Carcinogenesis* **30**, 78-87 (2009).
716. Chang YL, *et al.* Association of insulin receptor substrate-1 G972R variant with non-small cell lung cancer risk. *Tuberc Respir Dis (Seoul)* **67**, 8-13 (2009).
717. Chan-Yeung M, *et al.* Lung cancer susceptibility and polymorphisms of glutathione-S-transferase genes in Hong Kong. *Lung Cancer* **45**, 155-160 (2004).
718. Che J, *et al.* Polymorphism in PHD1 gene and risk of non-small cell lung cancer in a Chinese population. *Tumour Biol* **35**, 8921-8925 (2014).

719. Chen D, *et al.* Genetic variants in peroxisome proliferator-activated receptor-gamma gene are associated with risk of lung cancer in a Chinese population. *Carcinogenesis* **29**, 342-350 (2008).
720. Chen D, *et al.* Association of human aryl hydrocarbon receptor gene polymorphisms with risk of lung cancer among cigarette smokers in a Chinese population. *Pharmacogenet Genomics* **19**, 25-34 (2009).
721. Chen H, Lum A, Seifried A, Wilkens LR, Marchand LL. Association of the NAD(P)H:quinone oxidoreductase 609C->T polymorphism with a decreased lung cancer risk. *Cancer Res* **59**, 3045-3048 (1999).
722. Chen J, *et al.* A two-SNP IL-6 promoter haplotype is associated with increased lung cancer risk. *J Cancer Res Clin Oncol* **139**, 231-242 (2013).
723. Chen KY, *et al.* EGFR polymorphisms, hormone replacement therapy and lung adenocarcinoma risk: analysis from a genome-wide association study in never-smoking women. *Carcinogenesis* **34**, 612-619 (2013).
724. Chen S, *et al.* DNA repair gene XRCC1 and XPD polymorphisms and risk of lung cancer in a Chinese population. *Carcinogenesis* **23**, 1321-1325 (2002).
725. Chen S, Xue K, Xu L, Ma G, Wu J. Polymorphisms of the CYP1A1 and GSTM1 genes in relation to individual susceptibility to lung carcinoma in Chinese population. *Mutat Res* **458**, 41-47 (2001).
726. Chen WC, *et al.* The contribution of DNA apurinic/apyrimidinic endonuclease genotype and smoking habit to Taiwan lung cancer risk. *Anticancer Res* **33**, 2775-2778 (2013).
727. Chen XF, *et al.* Multiple variants of TERT and CLPTM1L constitute risk factors for lung adenocarcinoma. *Genet Mol Res* **11**, 370-378 (2012).
728. Chen Y, Liu H, Wu W, Li Y, Li J. Osteopontin genetic variants are associated with overall survival in advanced non-small-cell lung cancer patients and bone metastasis. *J Exp Clin Cancer Res* **32**, 45 (2013).
729. Chen YZ, *et al.* Single-nucleotide polymorphisms of LIG1 associated with risk of lung cancer. *Tumour Biol* **35**, 9229-9232 (2014).
730. Cheng M, *et al.* A microRNA-135a/b binding polymorphism in CD133 confers decreased risk and favorable prognosis of lung cancer in Chinese by reducing CD133 expression. *Carcinogenesis* **34**, 2292-2299 (2013).
731. Cheng TJ, Christiani DC, Wiencke JK, Wain JC, Xu X, Kelsey KT. Comparison of sister chromatid exchange frequency in peripheral lymphocytes in lung cancer cases and controls. *Mutat Res* **348**, 75-82 (1995).
732. Cheng Z, Wang W, Dai LL, Kang Y. MTHFR C667T polymorphism association with lung cancer risk in Henan province: a case-control study. *Asian Pac J Cancer Prev* **13**, 2491-2494 (2012).
733. Cheon KT, Choi KH, Lee HB, Park SK, Rhee YK, Lee YC. Gene polymorphisms of endothelial nitric oxide synthase and angiotensin-converting enzyme in patients with lung cancer. *Lung* **178**, 351-360 (2000).
734. Chernitsa OI, Togo AV, Shutkin VA, Hanson KP, Imyanitov EN. S allele of L-MYC oncogene is associated with metastatic lung cancer in patients from Moldova. *Eur J Cancer* **34**, 1816-1817 (1998).
735. Chevrier I, *et al.* Myeloperoxidase: new polymorphisms and relation with lung cancer risk. *Pharmacogenetics* **13**, 729-739 (2003).
736. Chi YJ, *et al.* Polymorphisms in the hMSH2 gene and the risk of primary lung cancer. *Cancer Epidemiol Biomarkers Prev* **15**, 762-768 (2006).
737. Chin LJ, *et al.* A SNP in a let-7 microRNA complementary site in the KRAS 3' untranslated region increases non-small cell lung cancer risk. *Cancer Res* **68**,

8535-8540 (2008).
738. Chiou HL, *et al.* NAT2 fast acetylator genotype is associated with an increased risk of lung cancer among never-smoking women in Taiwan. *Cancer Lett* **223**, 93-101 (2005).
739. Choi HS, *et al.* Single-nucleotide polymorphisms in the promoter of the CDK5 gene and lung cancer risk in a Korean population. *J Hum Genet* **54**, 298-303 (2009).
740. Choi JE, *et al.* No association between p73 G4C14-to-A4T14 polymorphism and the risk of lung cancer in a Korean population. *Biochem Genet* **44**, 543-550 (2006).
741. Choi JE, *et al.* Polymorphisms in telomere maintenance genes and risk of lung cancer. *Cancer Epidemiol Biomarkers Prev* **18**, 2773-2781 (2009).
742. Choi JE, *et al.* Polymorphisms in the epidermal growth factor receptor gene and the risk of primary lung cancer: a case-control study. *BMC Cancer* **7**, 199 (2007).
743. Choi YY, *et al.* Comprehensive assessment of P21 polymorphisms and lung cancer risk. *J Hum Genet* **53**, 87-95 (2008).
744. Chua HW, *et al.* Effect of MDM2 SNP309 and p53 codon 72 polymorphisms on lung cancer risk and survival among non-smoking Chinese women in Singapore. *BMC Cancer* **10**, 88 (2010).
745. Chujo M, Noguchi T, Miura T, Arinaga M, Uchida Y, Tagawa Y. Comparative genomic hybridization analysis detected frequent overrepresentation of chromosome 3q in squamous cell carcinoma of the lung. *Lung Cancer* **38**, 23-29 (2002).
746. Church TR, *et al.* Interaction of CYP1B1, cigarette-smoke carcinogen metabolism, and lung cancer risk. *Int J Mol Epidemiol Genet* **1**, 295-309 (2010).
747. Colakogullari M, *et al.* The involvement of IL-10, IL-6, IFN-gamma, TNF-alpha and TGF-beta gene polymorphisms among Turkish lung cancer patients. *Cell Biochem Funct* **26**, 283-290 (2008).
748. Coskunpinar E, *et al.* The significance of HSP90AA1, HSP90AB1 and HSP90B1 gene polymorphisms in a Turkish population with non-small cell lung cancer. *Anticancer Res* **34**, 753-757 (2014).
749. Coskunpinar E, Eraltan IY, Turna A, Agachan B. Cyclooxygenase-2 gene and lung carcinoma risk. *Med Oncol* **28**, 1436-1440 (2011).
750. Cote ML, *et al.* Combinations of cytochrome P-450 genotypes and risk of early-onset lung cancer in Caucasians and African Americans: a population-based study. *Lung Cancer* **55**, 255-262 (2007).
751. Cote ML, *et al.* Tobacco and estrogen metabolic polymorphisms and risk of non-small cell lung cancer in women. *Carcinogenesis* **30**, 626-635 (2009).
752. Crosbie PA, *et al.* GSTM1 copy number and lung cancer risk. *Mutat Res* **664**, 1-5 (2009).
753. Crosbie PA, *et al.* Association between lung cancer risk and single nucleotide polymorphisms in the first intron and codon 178 of the DNA repair gene, O6-alkylguanine-DNA alkyltransferase. *Int J Cancer* **122**, 791-795 (2008).
754. Cui E, *et al.* The role of adiponectin (ADIPOQ) gene polymorphisms in the susceptibility and prognosis of non-small cell lung cancer. *Biochem Cell Biol* **89**, 308-313 (2011).
755. Cui LH, *et al.* Methylenetetrahydrofolate reductase C677T polymorphism in patients with lung cancer in a Korean population. *BMC Med Genet* **12**, 28 (2011).
756. Cui LH, Yu Z, Zhang TT, Shin MH, Kim HN, Choi JS. Influence of

polymorphisms in MTHFR 677 C-->T, TYMS 3R-->2R and MTR 2756 A-->G on NSCLC risk and response to platinum-based chemotherapy in advanced NSCLC. *Pharmacogenomics* **12**, 797-808 (2011).
757. Cybulski C, *et al.* Constitutional CHEK2 mutations are associated with a decreased risk of lung and laryngeal cancers. *Carcinogenesis* **29**, 762-765 (2008).
758. Dai X, Liu Z, Ren J, Wu G. Association between insulin-like growth factor binding protein-3 promoter polymorphism of -1590 C>A and lung cancer susceptibility in a Chinese Han population. *Genet Test Mol Biomarkers* **15**, 301-306 (2011).
759. Dally H, *et al.* Genotype relationships in the CYP3A locus in Caucasians. *Cancer Lett* **207**, 95-99 (2004).
760. Dally H, *et al.* The CYP3A4*1B allele increases risk for small cell lung cancer: effect of gender and smoking dose. *Pharmacogenetics* **13**, 607-618 (2003).
761. Dally H, *et al.* Myeloperoxidase (MPO) genotype and lung cancer histologic types: the MPO -463 A allele is associated with reduced risk for small cell lung cancer in smokers. *Int J Cancer* **102**, 530-535 (2002).
762. David-Beabes GL, London SJ. Genetic polymorphism of XRCC1 and lung cancer risk among African-Americans and Caucasians. *Lung Cancer* **34**, 333-339 (2001).
763. David-Beabes GL, Lunn RM, London SJ. No association between the XPD (Lys751G1n) polymorphism or the XRCC3 (Thr241Met) polymorphism and lung cancer risk. *Cancer Epidemiol Biomarkers Prev* **10**, 911-912 (2001).
764. de Mello RA, *et al.* Association between EGF +61 genetic polymorphisms and non-small cell lung cancer increased risk in a Portuguese population: a case-control study. *Tumour Biol* **33**, 1341-1348 (2012).
765. de Mello RA, *et al.* The impact of polymorphic variations in the 5p15, 6p12, 6p21 and 15q25 Loci on the risk and prognosis of portuguese patients with non-small cell lung cancer. *PLoS One* **8**, e72373 (2013).
766. De Ruyck K, *et al.* Polymorphisms in base-excision repair and nucleotide-excision repair genes in relation to lung cancer risk. *Mutat Res* **631**, 101-110 (2007).
767. Debniak T, *et al.* CDKN2A common variant and multi-organ cancer risk - A population-based study. *Int J Cancer* **118**, 3180-3182 (2006).
768. Demir A, Altin S, Demir I, Koksal V, Cetincelik U, Dincer I. The role of CYP1A1 Msp1 gene polymorphisms on lung cancer development in Turkey. *Tuberk Toraks* **53**, 5-9 (2005).
769. Demir A, Altin S, Demir I, Koksal V, Cetincelik U, Dincer SI. The frequency of GSTT1 null genotype in Turkish population and lung cancer risk. *Indian J Hum Genet* **11**, 89-93 (2005).
770. Demir A, *et al.* The role of GSTM1 gene polymorphisms in lung cancer development in Turkish population. *J Carcinog* **6**, (2007).
771. Demirkan A, *et al.* CYP1A1, GSTM1 and NQO1 gene polymorphisms: Genetic risk factors for small cell lung cancer. *Turkish Journal of Cancer* **35**, 171-176 (2005).
772. Deng D, Zhang Y, Bao W, Kong X. Low-density lipoprotein receptor-related protein 6 (LRP6) rs10845498 polymorphism is associated with a decreased risk of non-small cell lung cancer. *Int J Med Sci* **11**, 685-690 (2014).
773. Deng Q, *et al.* Genetic polymorphisms in ATM, ERCC1, APE1 and iASPP genes and lung cancer risk in a population of southeast China. *Med Oncol* **28**, 667-672

(2011).
774. Devic Pavlic S, Ristic S, Flego V, Kapovic M, Radojcic Badovinac A. Angiotensin-converting enzyme insertion/deletion gene polymorphism in lung cancer patients. *Genet Test Mol Biomarkers* **16**, 722-725 (2012).
775. Dialyna IA, Miyakis S, Georgatou N, Spandidos DA. Genetic polymorphisms of CYP1A1, GSTM1 and GSTT1 genes and lung cancer risk. *Oncol Rep* **10**, 1829-1835 (2003).
776. Dogan I, *et al.* Polymorphisms in the aurora-A gene is not associated with lung cancer in the Turkish population. *DNA Cell Biol* **27**, 443-448 (2008).
777. Dogan I, *et al.* Polymorphisms in the vitamin D receptor gene and risk of lung cancer. *Med Sci Monit* **15**, Br232-242 (2009).
778. Dolzan V, Rudolf Z, Breskvar K. Human CYP2D6 gene polymorphism in Slovene cancer patients and healthy controls. *Carcinogenesis* **16**, 2675-2678 (1995).
779. Dolzan V, Rudolf Z, Breskvar K. Genetic polymorphism of xenobiotic metabolising enzymes in Slovenian lung cancer patients. *Pflugers Arch* **439**, R29-30 (2000).
780. Domanska D, *et al.* STAT3 rs3816769 polymorphism correlates with gene expression level and may predispose to nonsmall cell lung cancer: A preliminary study. *Pol Arch Med Wewn* **123**, 672-679 (2013).
781. Dragani TA, *et al.* Population-based mapping of pulmonary adenoma susceptibility 1 locus. *Cancer Res* **60**, 5017-5020 (2000).
782. Drakoulis N, Cascorbi I, Brockmoller J, Gross CR, Roots I. Polymorphisms in the human CYP1A1 gene as susceptibility factors for lung cancer: exon-7 mutation (4889 A to G), and a T to C mutation in the 3'-flanking region. *Clin Investig* **72**, 240-248 (1994).
783. Dresler CM, Fratelli C, Babb J, Everley L, Evans AA, Clapper ML. Gender differences in genetic susceptibility for lung cancer. *Lung Cancer* **30**, 153-160 (2000).
784. Dzian A, *et al.* Lung adenocarcinoma and squamous cell carcinoma in association with genetic polymorphisms of GSTs in Slovak population. *Neoplasma* **59**, 160-167 (2012).
785. el-Zein R, Zwischenberger JB, Wood TG, Abdel-Rahman SZ, Brekelbaum C, Au WW. Combined genetic polymorphism and risk for development of lung cancer. *Mutat Res* **381**, 189-200 (1997).
786. Enewold L, Mechanic LE, Bowman ED, Platz EA, Alberg AJ. Association of matrix metalloproteinase-1 polymorphisms with risk of COPD and lung cancer and survival in lung cancer. *Anticancer Res* **32**, 3917-3922 (2012).
787. Enewold L, Mechanic LE, Bowman ED, Platz EA, Alberg AJ. SERPINA1 and ELA2 polymorphisms are not associated with COPD or lung cancer. *Anticancer Res* **32**, 3923-3928 (2012).
788. Engels EA, Wu X, Gu J, Dong Q, Liu J, Spitz MR. Systematic evaluation of genetic variants in the inflammation pathway and risk of lung cancer. *Cancer Res* **67**, 6520-6527 (2007).
789. Enokida Y, *et al.* Single-nucleotide polymorphism (c.309T>G) in the MDM2 gene and lung cancer risk. *Biomedical Reports* **2**, 719-724 (2014).
790. Eom SY, *et al.* Influence of NQO1, ALDH2, and CYP2E1 genetic polymorphisms, smoking, and alcohol drinking on the risk of lung cancer in Koreans. *Cancer Causes Control* **20**, 137-145 (2009).
791. Eren A, *et al.* Investigation of possible relationship between survivin gene

promoter polymorphisms and lung cancer. *Clin Genet* **78**, 72 (2010).
792. Erfani N, Nedaei Ahmadi AS, Ghayumi MA, Mojtahedi Z. Genetic polymorphisms of CCL22 and CCR4 in patients with lung cancer. *Iran J Med Sci* **39**, 367-373 (2014).
793. Erkisi Z, Yaylim-Eraltan I, Turna A, Gormus U, Camlica H, Isbir T. Polymorphisms in the microsomal epoxide hydrolase gene: role in lung cancer susceptibility and prognosis. *Tumori* **96**, 756-763 (2010).
794. Ewis AA, *et al.* Surfactant protein B gene variations and susceptibility to lung cancer in chromate workers. *Am J Ind Med* **49**, 367-373 (2006).
795. Fakhoury H, Noureddine S, Chmaisse HN, Tamim H, Makki RF. MMP1-1607(1G>2G) polymorphism and the risk of lung cancer in Lebanon. *Ann Thorac Med* **7**, 130-132 (2012).
796. Fakhoury HM, Noureddine S, Tamim H, Chmaisse H, Makki R. Association of MMP3-1171(5A>6A) polymorphism with lung cancer in Lebanon. *Genet Test Mol Biomarkers* **16**, 988-990 (2012).
797. Falvella FS, *et al.* Transcription deregulation at the 15q25 locus in association with lung adenocarcinoma risk. *Clin Cancer Res* **15**, 1837-1842 (2009).
798. Falvella FS, *et al.* Common polymorphisms in D12S1034 flanking genes RASSF8 and BHLHB3 are not associated with lung adenocarcinoma risk. *Lung Cancer* **56**, 1-7 (2007).
799. Fan R, *et al.* The p53 codon 72 polymorphism and lung cancer risk. *Cancer Epidemiol Biomarkers Prev* **9**, 1037-1042 (2000).
800. Fang HM, Tian G, Zhou LJ, Zhou HY, Fang YZ. FGFR4 genetic polymorphisms determine the chemotherapy response of Chinese patients with non-small cell lung cancer. *Acta Pharmacol Sin* **34**, 549-554 (2013).
801. Fang S, *et al.* Polymorphisms in the MMP1 and MMP3 promoter and non-small cell lung carcinoma in North China. *Carcinogenesis* **26**, 481-486 (2005).
802. Fang W, *et al.* The functional polymorphism of NBS1 p.Glu185Gln is associated with an increased risk of lung cancer in Chinese populations: Case-control and a meta-analysis. *Mutat Res* **770**, 61-68 (2014).
803. Fang X, *et al.* Genetic variation in an miRNA-1827 binding site in MYCL1 alters susceptibility to small-cell lung cancer. *Cancer Res* **71**, 5175-5181 (2011).
804. Farjadfar A, Mojtahedi Z, Ghayumi MA, Erfani N, Haghshenas MR, Ghaderi A. Interleukin-18 promoter polymorphism is associated with lung cancer: a case-control study. *Acta Oncol* **48**, 971-976 (2009).
805. Fathy M, Hamed M, Youssif O, Fawzy N, Ashour W. Association between environmental tobacco smoke exposure and lung cancer susceptibility: modification by antioxidant enzyme genetic polymorphisms. *Mol Diagn Ther* **18**, 55-62 (2014).
806. Fatma B, Aida T, Maurice JA, Saguem S. Glutathione S-transferase M1 and T1, CYP1A2-2467T/delT polymorphisms and non small-cell lung cancer risk in Tunisian sample. *Egyptian Journal of Medical Human Genetics* **13**, 307-312 (2012).
807. Fernandez-Rubio A, *et al.* The TP53 Arg72Pro polymorphism and lung cancer risk in a population of Northern Spain. *Lung Cancer* **61**, 309-316 (2008).
808. Feyler A, *et al.* Point: myeloperoxidase -463G --> a polymorphism and lung cancer risk. *Cancer Epidemiol Biomarkers Prev* **11**, 1550-1554 (2002).
809. Flego V, *et al.* Tumor necrosis factor-alpha gene promoter -308 and -238 polymorphisms in patients with lung cancer as a second primary tumor. *Med Sci Monit* **19**, 846-851 (2013).

810. Ford JG, *et al.* Glutathione S-transferase M1 polymorphism and lung cancer risk in African-Americans. *Carcinogenesis* **21**, 1971-1975 (2000).
811. Fujieda M, *et al.* Evaluation of CYP2A6 genetic polymorphisms as determinants of smoking behavior and tobacco-related lung cancer risk in male Japanese smokers. *Carcinogenesis* **25**, 2451-2458 (2004).
812. Gabrielsen ME, Romundstad P, Langhammer A, Krokan HE, Skorpen F. Association between a 15q25 gene variant, nicotine-related habits, lung cancer and COPD among 56,307 individuals from the HUNT study in Norway. *Eur J Hum Genet* **21**, 1293-1299 (2013).
813. Gallagher CJ, *et al.* The UDP-glucuronosyltransferase 2B17 gene deletion polymorphism: sex-specific association with urinary 4-(methylnitrosamino)-1-(3-pyridyl)-1-butanol glucuronidation phenotype and risk for lung cancer. *Cancer Epidemiol Biomarkers Prev* **16**, 823-828 (2007).
814. Gallegos-Arreola MP, *et al.* CYP1A1 *2B and *4 polymorphisms are associated with lung cancer susceptibility in Mexican patients. *Int J Biol Markers* **23**, 24-30 (2008).
815. Gallegos-Arreola MP, *et al.* GSTT1 gene deletion is associated with lung cancer in Mexican patients. *Dis Markers* **19**, 259-261 (2003).
816. Galvan A, *et al.* Genome-wide association study in discordant sibships identifies multiple inherited susceptibility alleles linked to lung cancer. *Carcinogenesis* **31**, 462-465 (2010).
817. Gao M, *et al.* The relationship between three well-characterized polymorphisms of the angiotensin converting enzyme gene and lung cancer risk: a case-control study and a meta-analysis. *J Renin Angiotensin Aldosterone Syst* **13**, 455-460 (2012).
818. Gao Y, Zhang Q. Polymorphisms of the GSTM1 and CYP2D6 genes associated with susceptibility to lung cancer in Chinese. *Mutat Res* **444**, 441-449 (1999).
819. Garcia-Closas M, Kelsey KT, Wiencke JK, Xu X, Wain JC, Christiani DC. A case-control study of cytochrome P450 1A1, glutathione S-transferase M1, cigarette smoking and lung cancer susceptibility (Massachusetts, United States). *Cancer Causes Control* **8**, 544-553 (1997).
820. Garcia-Martin E, Martinez C, Ladero JM, Gamito FJ, Rodriguez-Lescure A, Agundez JA. Influence of cytochrome P450 CYP2C9 genotypes in lung cancer risk. *Cancer Lett* **180**, 41-46 (2002).
821. Gaspar P, Moreira J, Kvitko K, Torres M, Moreira A, de Azevedo Weimer T. CYP1A1, CYP2E1, GSTM1, GSTT1, GSTP1, and TP53 polymorphisms: Do they indicate susceptibility to chronic obstructive pulmonary disease and non-small-cell lung cancer? *Genet Mol Biol* **27**, 133-138 (2004).
822. Gautschi O, *et al.* Cyclin D1 (CCND1) A870G gene polymorphism modulates smoking-induced lung cancer risk and response to platinum-based chemotherapy in non-small cell lung cancer (NSCLC) patients. *Lung Cancer* **51**, 303-311 (2006).
823. Ge H, *et al.* Detection and evaluation of p53 intron 2 polymorphism in lung carcinomas in Hong Kong. *Int J Cancer* **69**, 120-124 (1996).
824. Gervasini G, Carrillo JA, Garcia M, San Jose C, Cabanillas A, Benitez J. Adenosine triphosphate-binding cassette B1 (ABCB1) (multidrug resistance 1) G2677T/A gene polymorphism is associated with high risk of lung cancer. *Cancer* **107**, 2850-2857 (2006).
825. Gervasini G, *et al.* Haplotypes in the 5'-untranslated region of the CYP1A2 gene are inversely associated with lung cancer risk but do not correlate with caffeine

metabolism. *Environ Mol Mutagen* **54**, 124-132 (2013).
826. Gervasini G, Jose CS, Carrillo JA, Benitez J, Cabanillas A. GST polymorphisms interact with dietary factors to modulate lung cancer risk: Study in a high-incidence area. *Nutr Cancer* **62**, 750-758 (2010).
827. Golmoghaddam H, Pezeshki AM, Ghaderi A, Doroudchi M. CD1a and CD1d genes polymorphisms in breast, colorectal and lung cancers. *Pathol Oncol Res* **17**, 669-675 (2011).
828. Gomes M, Coelho A, Araujo A, Teixeira AL, Catarino R, Medeiros R. Influence of functional genetic polymorphism (-590C/T) in non-small cell lung cancer (NSCLC) development: the paradoxal role of IL-4. *Gene* **504**, 111-115 (2012).
829. Gonzalez-Arriaga P, *et al.* Polymorphism +17 C/G in matrix metalloprotease MMP8 decreases lung cancer risk. *BMC Cancer* **8**, 378 (2008).
830. Gonzalez-Arriaga P, Pascual T, Garcia-Alvarez A, Fernandez-Somoano A, Lopez-Cima MF, Tardon A. Genetic polymorphisms in MMP 2, 9 and 3 genes modify lung cancer risk and survival. *BMC Cancer* **12**, 121 (2012).
831. Gorlov IP, *et al.* Seizure 6-like (SEZ6L) gene and risk for lung cancer. *Cancer Res* **67**, 8406-8411 (2007).
832. Gormus U, *et al.* Fas-1377 A/G polymorphism in lung cancer. *In Vivo* **21**, 663-666 (2007).
833. Gromowski T, *et al.* Prevalence of the E318K and V320I MITF germline mutations in Polish cancer patients and multiorgan cancer risk-a population-based study. *Cancer Genet* **207**, 128-132 (2014).
834. Gruber M, *et al.* UDP-glucuronosyltransferase 2B17 genotype and the risk of lung cancer among Austrian Caucasians. *Cancer Epidemiol* **37**, 625-628 (2013).
835. Gsur A, *et al.* Genetic polymorphisms of CYP1A1 and GSTM1 and lung cancer risk. *Anticancer Res* **21**, 2237-2242 (2001).
836. Gsur A, *et al.* Association of microsomal epoxide hydrolase polymorphisms and lung cancer risk. *Br J Cancer* **89**, 702-706 (2003).
837. Gu J, Gong Y, Huang M, Lu C, Spitz MR, Wu X. Polymorphisms of STK15 (Aurora-A) gene and lung cancer risk in Caucasians. *Carcinogenesis* **28**, 350-355 (2007).
838. Gu J, Shen Y, Zhang Y. Association between interleukin-4 polymorphisms and environment and nonsmall cell lung cancer in Chinese population. *J Cancer Res Ther* **10**, C135-C139 (2014).
839. Gu J, Spitz MR, Yang F, Wu X. Ethnic differences in poly(ADP-ribose) polymerase pseudogene genotype distribution and association with lung cancer risk. *Carcinogenesis* **20**, 1465-1469 (1999).
840. Gu J, *et al.* A nonsynonymous single-nucleotide polymorphism in the PDZ-Rho guanine nucleotide exchange factor (Ser1416Gly) modulates the risk of lung cancer in Mexican Americans. *Cancer* **106**, 2716-2724 (2006).
841. Guan X, Wang LE, Liu Z, Sturgis EM, Wei Q. Association between a rare novel TP53 variant (rs78378222) and melanoma, squamous cell carcinoma of head and neck and lung cancer susceptibility in non-Hispanic Whites. *J Cell Mol Med* **17**, 873-878 (2013).
842. Gunes EG, Pinarbasi E, Pinarbasi H, Silig Y. Strong association between lung cancer and the AXIN2 polymorphism. *Mol Med Rep* **2**, 1029-1035 (2009).
843. Guo H, *et al.* Functional promoter -1271G>C variant of HSPB1 predicts lung cancer risk and survival. *J Clin Oncol* **28**, 1928-1935 (2010).
844. Guo H, *et al.* Variations in HSPA1B at 6p21.3 are associated with lung cancer risk and prognosis in Chinese populations. *Cancer Res* **71**, 7576-7586 (2011).

845. Guo S, *et al.* Synergistic association of PTGS2 and CYP2E1 genetic polymorphisms with lung cancer risk in northeastern Chinese. *PLoS One* **7**, e39814 (2012).
846. Guo S, Li X, Gao M, Li Y, Song B, Niu W. The relationship between XRCC1 and XRCC3 gene polymorphisms and lung cancer risk in northeastern Chinese. *PLoS One* **8**, e56213 (2013).
847. Guo Y, *et al.* Functional evaluation of missense variations in the human MAD1L1 and MAD2L1 genes and their impact on susceptibility to lung cancer. *J Med Genet* **47**, 616-622 (2010).
848. Habalova V, Salagovic J, Kalina I, Stubna J. A pilot study testing the genetic polymorphism of N-acetyltransferase 2 as a risk factor in lung cancer. *Neoplasma* **52**, 364-368 (2005).
849. Hagihara M, Shimura T, Sato K, Genga K, Suzuki M, Tsuji K. HLA and tumor necrosis factor beta gene polymorphisms in Okinawa lung cancer patients: comparative study with mainland Japan lung cancer patients. *Hum Immunol* **43**, 95-100 (1995).
850. Hamada GS, *et al.* The heme-binding region polymorphism of cytochrome P450IA1 (CypIA1), rather than the RsaI polymorphism of IIE1 (CypIIE1), is associated with lung cancer in Rio de Janeiro. *Cancer Epidemiol Biomarkers Prev* **4**, 63-67 (1995).
851. Hamajima N, *et al.* NAD(P)H: quinone oxidoreductase 1 (NQO1) C609T polymorphism and the risk of eight cancers for Japanese. *Int J Clin Oncol* **7**, 103-108 (2002).
852. Han SG, *et al.* Single nucleotide polymorphisms of IGFBP-3 gene and lung cancer risk in a Korean population. *Lung Cancer* **62**, 152-161 (2008).
853. Hao B, *et al.* A novel T-77C polymorphism in DNA repair gene XRCC1 contributes to diminished promoter activity and increased risk of non-small cell lung cancer. *Oncogene* **25**, 3613-3620 (2006).
854. Harms C, Salama SA, Sierra-Torres CH, Cajas-Salazar N, Au WW. Polymorphisms in DNA repair genes, chromosome aberrations, and lung cancer. *Environ Mol Mutagen* **44**, 74-82 (2004).
855. Hart K, Landvik NE, Lind H, Skaug V, Haugen A, Zienolddiny S. A combination of functional polymorphisms in the CASP8, MMP1, IL10 and SEPS1 genes affects risk of non-small cell lung cancer. *Lung Cancer* **71**, 123-129 (2011).
856. Hayashi SI, Watanabe J, Kawajiri K. High susceptibility to lung cancer analyzed in terms of combined genotypes of P450IA1 and mu-class glutathione S-transferase genes. *Jpn J Cancer Res* **83**, 866-870 (1992).
857. He F, Chang SC, Wallar GM, Zhang ZF, Cai L. Association of XRCC3 and XRCC4 gene polymorphisms, family history of cancer and tobacco smoking with non-small-cell lung cancer in a Chinese population: a case-control study. *J Hum Genet* **58**, 679-685 (2013).
858. He P, *et al.* CHRNA3 polymorphism modifies lung adenocarcinoma risk in the Chinese Han population. *Int J Mol Sci* **15**, 5446-5457 (2014).
859. He YQ, Bo Q, Yong W, Qiu ZX, Li YL, Li WM. FoxP3 genetic variants and risk of non-small cell lung cancer in the Chinese Han population. *Gene* **531**, 422-425 (2013).
860. He YQ, Zhou B, Shi SQ, Zhang L, Li WM. Genetic variation in PDCD6 and susceptibility to lung cancer. *Asian Pac J Cancer Prev* **13**, 4689-4693 (2012).
861. Heijmans BT, *et al.* A common variant of the methylenetetrahydrofolate reductase gene (1p36) is associated with an increased risk of cancer. *Cancer Res*

63, 1249-1253 (2003).
862. Heikkila K, *et al.* C-reactive protein-associated genetic variants and cancer risk: findings from FINRISK 1992, FINRISK 1997 and Health 2000 studies. *Eur J Cancer* **47**, 404-412 (2011).
863. Helmig S, Aliahmadi N, Schneider J. Tumour necrosis factor-alpha gene polymorphisms in asbestos-induced diseases. *Biomarkers* **15**, 400-409 (2010).
864. Helmig S, Belwe A, Schneider J. Association of transforming growth factor beta1 gene polymorphisms and asbestos-induced fibrosis and tumors. *J Investig Med* **57**, 655-661 (2009).
865. Helmig S, Grossmann M, Wubbeling J, Schneider J. Interleukin gene polymorphisms in pneumoconiosis. *Int J Mol Med* **30**, 401-408 (2012).
866. Hiraki A, *et al.* Different risk relations with smoking for non-small-cell lung cancer: comparison of TP53 and TP73 genotypes. *Asian Pac J Cancer Prev* **4**, 107-112 (2003).
867. Hirvonen A, Husgafvel-Pursiainen K, Anttila S, Karjalainen A, Sorsa M, Vainio H. Metabolic cytochrome P450 genotypes and assessment of individual susceptibility to lung cancer. *Pharmacogenetics* **2**, 259-263 (1992).
868. Hirvonen A, Husgafvel-Pursiainen K, Anttila S, Karjalainen A, Vainio H. The human CYP2E1 gene and lung cancer: DraI and RsaI restriction fragment length polymorphisms in a Finnish study population. *Carcinogenesis* **14**, 85-88 (1993).
869. Hirvonen A, Husgafvel-Pursiainen K, Anttila S, Karjalainen A, Vainio H. Polymorphism in CYP1A1 and CYP2D6 genes: possible association with susceptibility to lung cancer. *Environ Health Perspect* **101 Suppl 3**, 109-112 (1993).
870. Hirvonen A, Husgafvel-Pursiainen K, Anttila S, Vainio H. The GSTM1 null genotype as a potential risk modifier for squamous cell carcinoma of the lung. *Carcinogenesis* **14**, 1479-1481 (1993).
871. Hirvonen A, Husgafvel-Pursiainen K, Karjalainen A, Anttila S, Vainio H. Point-mutational MspI and Ile-Val polymorphisms closely linked in the CYP1A1 gene: lack of association with susceptibility to lung cancer in a Finnish study population. *Cancer Epidemiol Biomarkers Prev* **1**, 485-489 (1992).
872. Ho JC, *et al.* Manganese superoxide dismutase and catalase genetic polymorphisms, activity levels, and lung cancer risk in Chinese in Hong Kong. *J Thorac Oncol* **1**, 648-653 (2006).
873. Hong YS, Chang JH, Kwon OJ, Ham YA, Choi JH. Polymorphism of the CYP1A1 and glutathione-S-transferase gene in Korean lung cancer patients. *Exp Mol Med* **30**, 192-198 (1998).
874. Hong YS, *et al.* Association between microRNA196a2 rs11614913 genotypes and the risk of non-small cell lung cancer in Korean population. *J Prev Med Public Health* **44**, 125-130 (2011).
875. Honma HN, De Capitani EM, Barbeiro Ade S, Costa DB, Morcillo A, Zambon L. Polymorphism of the CYP1A1*2A gene and susceptibility to lung cancer in a Brazilian population. *J Bras Pneumol* **35**, 767-772 (2009).
876. Honma HN, *et al.* Influence of p53 codon 72 exon 4, GSTM1, GSTT1 and GSTP1*B polymorphisms in lung cancer risk in a Brazilian population. *Lung Cancer* **61**, 152-162 (2008).
877. Hou SM, *et al.* The XPD variant alleles are associated with increased aromatic DNA adduct level and lung cancer risk. *Carcinogenesis* **23**, 599-603 (2002).
878. Hou SM, Falt S, Nyberg F. Glutathione S-transferase T1-null genotype interacts synergistically with heavy smoking on lung cancer risk. *Environ Mol Mutagen*

38, 83-86 (2001).
879. Hou SM, *et al.* GSTM1 and NAT2 polymorphisms in operable and non-operable lung cancer patients. *Carcinogenesis* **21**, 49-54 (2000).
880. Hsia TC, *et al.* Genotype of DNA double-strand break repair gene XRCC7 is associated with lung cancer risk in Taiwan males and smokers. *Anticancer Res* **34**, 7001-7005 (2014).
881. Hsia TC, *et al.* Interleukin-10 (IL-10) promoter genotypes are associated with lung cancer risk in Taiwan males and smokers. *Anticancer Res* **34**, 7039-7044 (2014).
882. Hsia TC, *et al.* Association of DNA double-strand break gene XRCC6 genotypes and lung cancer in Taiwan. *Anticancer Res* **32**, 1015-1020 (2012).
883. Hsia TC, *et al.* Interaction of CCND1 genotype and smoking habit in Taiwan lung cancer patients. *Anticancer Res* **31**, 3601-3605 (2011).
884. Hsia TC, *et al.* Effects of ataxia telangiectasia mutated (ATM) genotypes and smoking habits on lung cancer risk in Taiwan. *Anticancer Res* **33**, 4067-4071 (2013).
885. Hsieh YS, *et al.* Association of EcoRI polymorphism of the metastasis-suppressor gene NME1 with susceptibility to and severity of non-small cell lung cancer. *Lung Cancer* **58**, 191-195 (2007).
886. Hsu NY, *et al.* Lung cancer susceptibility and genetic polymorphism of DNA repair gene XRCC4 in Taiwan. *Cancer Biomark* **5**, 159-165 (2009).
887. Hsu NY, *et al.* Lung cancer susceptibility and genetic polymorphisms of Exo1 gene in Taiwan. *Anticancer Res* **29**, 725-730 (2009).
888. Hu QY, *et al.* Genetic variation in the TP63 gene is associated with lung cancer risk in the Han population. *Tumour Biol* **35**, 1863-1866 (2014).
889. Hu Z, *et al.* Functional polymorphisms of matrix metalloproteinase-9 are associated with risk of occurrence and metastasis of lung cancer. *Clin Cancer Res* **11**, 5433-5439 (2005).
890. Hu Z, *et al.* Tagging single nucleotide polymorphisms in phosphoinositide-3-kinase- related protein kinase genes involved in DNA damage "checkpoints" and lung cancer susceptibility. *Clin Cancer Res* **14**, 2887-2891 (2008).
891. Hu Z, *et al.* Genetic variants in the MDM2 promoter and lung cancer risk in a Chinese population. *Int J Cancer* **118**, 1275-1278 (2006).
892. Hu Z, *et al.* A promoter polymorphism (-77T>C) of DNA repair gene XRCC1 is associated with risk of lung cancer in relation to tobacco smoking. *Pharmacogenet Genomics* **15**, 457-463 (2005).
893. Hu Z, *et al.* A common polymorphism in the 3'UTR of cyclooxygenase 2/prostaglandin synthase 2 gene and risk of lung cancer in a Chinese population. *Lung Cancer* **48**, 11-17 (2005).
894. Hu Z, *et al.* Polymorphisms in DNA damage binding protein 2 (DDB2) and susceptibility of primary lung cancer in the Chinese: a case-control study. *Carcinogenesis* **27**, 1475-1480 (2006).
895. Hu Z, *et al.* DNA repair gene XPC genotypes/haplotypes and risk of lung cancer in a Chinese population. *Int J Cancer* **115**, 478-483 (2005).
896. Hu Z, *et al.* Polymorphisms in the two helicases ERCC2/XPD and ERCC3/XPB of the transcription factor IIH complex and risk of lung cancer: a case-control analysis in a Chinese population. *Cancer Epidemiol Biomarkers Prev* **15**, 1336-1340 (2006).
897. Huang B, *et al.* Functional genetic variants of c-Jun and their interaction with

smoking and drinking increase the susceptibility to lung cancer in southern and eastern Chinese. *Int J Cancer* **131**, E744-758 (2012).
898. Yang L, *et al.* The polymorphisms and haplotypes of WWOX gene are associated with the risk of lung cancer in southern and eastern Chinese populations. *Mol Carcinog* **52 Suppl 1**, E19-27 (2013).
899. Huijts PE, *et al.* CHEK2*1100delC homozygosity in the Netherlands-prevalence and risk of breast and lung cancer. *Eur J Hum Genet* **22**, 46-51 (2014).
900. Hung RJ, *et al.* Sequence variants in cell cycle control pathway, X-ray exposure, and lung cancer risk: a multicenter case-control study in Central Europe. *Cancer Res* **66**, 8280-8286 (2006).
901. Hung RJ, *et al.* Large-scale investigation of base excision repair genetic polymorphisms and lung cancer risk in a multicenter study. *J Natl Cancer Inst* **97**, 567-576 (2005).
902. Hussein AG, Pasha HF, El-Shahat HM, Gad DM, Toam MM. CYP1A1 gene polymorphisms and smoking status as modifier factors for lung cancer risk. *Gene* **541**, 26-30 (2014).
903. Ihsan R, *et al.* Copy number polymorphism of glutathione-S-transferase genes (GSTM1 & GSTT1) in susceptibility to lung cancer in a high-risk population from north-east India. *Indian J Med Res* **139**, 720-729 (2014).
904. Ihsan R, *et al.* Investigation on the role of p53 codon 72 polymorphism and interactions with tobacco, betel quid, and alcohol in susceptibility to cancers in a high-risk population from North East India. *DNA Cell Biol* **30**, 163-171 (2011).
905. Irarrazabal CE, Rojas C, Aracena R, Marquez C, Gil L. Chilean pilot study on the risk of lung cancer associated with codon 72 polymorphism in the gene of protein p53. *Toxicol Lett* **144**, 69-76 (2003).
906. Ishibe N, Wiencke JK, Zuo ZF, McMillan A, Spitz M, Kelsey KT. Susceptibility to lung cancer in light smokers associated with CYP1A1 polymorphisms in Mexican- and African-Americans. *Cancer Epidemiol Biomarkers Prev* **6**, 1075-1080 (1997).
907. Islam MS, *et al.* Lung cancer risk in relation to nicotinic acetylcholine receptor, CYP2A6 and CYP1A1 genotypes in the Bangladeshi population. *Clin Chim Acta* **416**, 11-19 (2013).
908. Islam MS, Mostofa AG, Ahmed MU, Bin Sayeed MS, Hassan MR, Hasnat A. Association of CYP3A4, CYP3A5 polymorphisms with lung cancer risk in Bangladeshi population. *Tumour Biol* **35**, 1671-1678 (2014).
909. Ito H, *et al.* A limited association of OGG1 Ser326Cys polymorphism for adenocarcinoma of the lung. *J Epidemiol* **12**, 258-265 (2002).
910. Ito H, *et al.* Gene-environment interactions between the smoking habit and polymorphisms in the DNA repair genes, APE1 Asp148Glu and XRCC1 Arg399Gln, in Japanese lung cancer risk. *Carcinogenesis* **25**, 1395-1401 (2004).
911. Ito H, *et al.* Association between a genome-wide association study-identified locus and the risk of lung cancer in Japanese population. *J Thorac Oncol* **7**, 790-798 (2012).
912. Iwao N, *et al.* No association of the mitochondrial genotype (Mt5178A/C) with six cancers in a Japanese population. *Asian Pac J Cancer Prev* **4**, 331-336 (2003).
913. Jablonska E, Gromadzinska J, Sobala W, Reszka E, Wasowicz W. Lung cancer risk associated with selenium status is modified in smoking individuals by Sep15 polymorphism. *Eur J Nutr* **47**, 47-54 (2008).
914. Jacobsen NR, *et al.* XRCC3 polymorphisms and risk of lung cancer. *Cancer Lett*

213, 67-72 (2004).
915. Jacquet M, Lambert V, Baudoux E, Muller M, Kremers P, Gielen J. Correlation between P450 CYP1A1 inducibility, MspI genotype and lung cancer incidence. *Eur J Cancer* **32a**, 1701-1706 (1996).
916. Jang JS, *et al.* Identification of polymorphisms in the Caspase-3 gene and their association with lung cancer risk. *Mol Carcinog* **47**, 383-390 (2008).
917. Jang JS, *et al.* Polymorphisms in the survivin gene and the risk of lung cancer. *Lung Cancer* **60**, 31-39 (2008).
918. Jang JS, *et al.* Methyl-CpG binding domain 1 gene polymorphisms and risk of primary lung cancer. *Cancer Epidemiol Biomarkers Prev* **14**, 2474-2480 (2005).
919. Javid J, *et al.* Clinical and prognostic significance of promoter polymorphism (-31GC) of anti apoptotic gene Survivin (BIRC5) in North India patients with non small cell lung cancer. *J Cancer Sci Ther* **4**, 276-280 (2012).
920. Javid J, *et al.* Impact of MDM2 SNP309T>G polymorphism: Increased risk of developing non small cell lung cancer and poor prognosis in indian patients. *J Cancer Sci Ther* **4**, 341-346 (2012).
921. Javid J, *et al.* CC genotype of anti-apoptotic gene BCL-2 (null938 C/A) is an independent prognostic marker of unfavorable clinical outcome in patients with non-small-cell lung cancer. *Clin Transl Oncol* **17**, 289-295 (2015).
922. Jaworowska E, *et al.* Smoking related cancers and loci at chromosomes 15q25, 5p15, 6p22.1 and 6p21.33 in the Polish population. *PLoS One* **6**, e25057 (2011).
923. Jeon HS, *et al.* Relationship between XPG codon 1104 polymorphism and risk of primary lung cancer. *Carcinogenesis* **24**, 1677-1681 (2003).
924. Jeon HS, *et al.* A common polymorphism in pre-microRNA-146a is associated with lung cancer risk in a Korean population. *Gene* **534**, 66-71 (2014).
925. Ada AO, *et al.* Association between GSTM1, GSTT1, and GSTP1 polymorphisms and lung cancer risk in a Turkish population. *Mol Biol Rep* **39**, 5985-5993 (2012).
926. Ada TG, Ada AO, Kunak SC, Alpar S, Gulhan M, Iscan M. Association between glutathione S-transferase omega 1 A140D polymorphism in the Turkish population and susceptibility to non-small cell lung cancer. *Arh Hig Rada Toksikol* **64**, 247-253 (2013).
927. Adonis M, Martinez V, Marin P, Berrios D, Gil L. Smoking habit and genetic factors associated with lung cancer in a population highly exposed to arsenic. *Toxicol Lett* **159**, 32-37 (2005).
928. Agachan B, *et al.* Apurinic/apyrimidinic endonuclease (APE1) gene polymorphisms and lung cancer risk in relation to tobacco smoking. *Anticancer Res* **29**, 2417-2420 (2009).
929. Yang X, *et al.* Association of the functional BCL-2 rs2279115 genetic variant and small cell lung cancer. *Tumour Biol*, (2015).
930. Kurt H, *et al.* Determination of the Relationship Between rs4986790 and rs4986791 Variants of TLR4 Gene and Lung Cancer. *Inflammation*, (2015).
931. Qiu ZX, Peng Y, Li WM. CD226 gene polymorphisms are associated with non-small-cell lung cancer in the Chinese han population. *Ther Clin Risk Manag* **11**, 1259-1264 (2015).
932. Subhani S, Jamil K, Nirni SS. Association of MDR1 Gene (C3435T) Polymorphism and Gene Expression Profiling in Lung Cancer Patients Treated with Platinum-Based Chemotherapy. *Molecular Diagnosis and Therapy* **19**, 289-297 (2015).
933. Gao F, *et al.* A regulatory MDM4 genetic variant locating in the binding

sequence of multiple MicroRNAs contributes to susceptibility of small cell lung cancer. *PLoS One* **10**, (2015).
934. Hosono H, *et al.* CYP2A6 genetic polymorphism is associated with decreased susceptibility to squamous cell lung cancer in Japanese smokers. *Drug Metab Pharmacokinet* **30**, 263-268 (2015).
935. Ozbayer C, *et al.* The role of NOD1/CARD4 and NOD2/CARD15 genetic variations in lung cancer risk. *Inflamm Res* **64**, 775-779 (2015).
936. Pei N, *et al.* XAB2 tagSNPs contribute to non-small cell lung cancer susceptibility in Chinese population. *BMC Cancer* **15**, (2015).
937. Liu HN, Su JL, Zhou SH, Liu LJ, Qie P. Cytotoxic T lymphocyte-associated antigen-4 +49A>G polymorphism and the risk of non-small cell lung cancer in a Chinese population. *Int J Clin Exp Med* **8**, 11519-11523 (2015).
938. Zhou W, *et al.* CHRNA3 genetic polymorphism and the risk of lung cancer in the Chinese Han smoking population. *Tumour Biol* **36**, 4987-4992 (2015).
939. Javid J, Mir R, Julka PK, Ray PC, Saxena A. Association of p53 and mdm2 in the development and progression of non-small cell lung cancer. *Tumour Biol* **36**, 5425-5432 (2015).
940. Javid J, Mir R, Julka PK, Ray PC, Saxena A. Role of survivin re-expression in the development and progression of non-small cell lung cancer. *Tumour Biol* **36**, 5543-5550 (2015).
941. Wang T, *et al.* Association of PSMA4 polymorphisms with lung cancer susceptibility and response to cisplatin-based chemotherapy in a Chinese Han population. *Clin Transl Oncol* **17**, 564-569 (2015).
942. Dong Y, *et al.* Evaluating the association of eight polymorphisms with cancer susceptibility in a han Chinese population. *PLoS One* **10**, (2015).
943. Chen HY, *et al.* R331W missense mutation of oncogene YAP1 is a germline risk allele for lung adenocarcinoma with medical actionability. *J Clin Oncol* **33**, 2303-2310 (2015).
944. Chen HJ, *et al.* Contribution of Genotype of DNA Double-strand Break Repair Gene XRCC3, Gender, and Smoking Behavior to Lung Cancer Risk in Taiwan. *Anticancer Res* **35**, 3893-3899 (2015).
945. Tian H, He X, Yin L, Guo WJ, Xia YY, Jiang ZX. Relationship between genetic polymorphisms of DNA ligase 1 and non-small cell lung cancer susceptibility and radiosensitivity. *Genet Mol Res* **14**, 7047-7052 (2015).
946. Yin Z, *et al.* Interaction between polymorphisms in pre-miRNA genes and cooking oil fume exposure on the risk of lung cancer in Chinese non-smoking female population. *PLoS One* **10**, (2015).
947. Wang Q, *et al.* The SNP rs931794 in 15q25.1 is associated with lung cancer risk: A hospital-based case-control study and meta-analysis. *PLoS One* **10**, (2015).
948. Xiang C, *et al.* Pulmonary expression of CYP2A13 and ABCB1 is regulated by FOXA2, and Their genetic interaction is associated with lung cancer. *FASEB J* **29**, 1986-1998 (2015).
949. Liu SG, Ma L, Cen QH, Huang JS, Zhang JX, Zhang JJ. Association of genetic polymorphisms in TERT-CLPTM1L with lung cancer in a chinese population. *Genet Mol Res* **14**, 4469-4476 (2015).
950. Yang L, Wang J, Li FG, Han M, Chang XJ, Wang ZT. Relationship between genetic polymorphisms in MCP-1, CCR-2, and non-small-cell lung cancer in the Han nationality of Northern China. *Genet Mol Res* **14**, 3745-3752 (2015).
951. Ren Y, *et al.* TGFβ-1 and TGFBR2 polymorphisms, cooking oil fume exposure and risk of lung adenocarcinoma in Chinese nonsmoking females: A case control

study. *BMC Med Genet* **16**, (2015).
952. Eom SY, *et al.* Interactions between paraoxonase 1 genetic polymorphisms and smoking and their effects on oxidative stress and lung cancer risk in a Korean population. *PLoS One* **10**, (2015).
953. Ryan BM, *et al.* Identification of a functional SNP in the 3′UTR of CXCR2 that is associated with reduced risk of lung cancer. *Cancer Res* **75**, 566-575 (2015).
954. Zhang YM, Mao YM, Sun YX. Genetic polymorphisms of IL-6 and IL-10 genes correlate with lung cancer in never-smoking han population in China. *Int J Clin Exp Med* **8**, 1051-1058 (2015).
955. Zhao H, *et al.* FasL −844T/C and Fas −1377G/A: mutations of pulmonary adenocarcinoma in South China and their clinical significances. *Tumour Biol* **36**, 4319-4326 (2015).
956. Zhu DQ, Zou Q, Hu CH, Su JL, Zhou GH, Liu P. XRCC1 genetic polymorphism acts a potential biomarker for lung cancer. *Tumour Biol* **36**, 3745-3750 (2015).
957. Ebi H, *et al.* Lack of association between the BIM deletion polymorphism and the risk of lung cancer with and without EGFR mutations. *J Thorac Oncol* **10**, 59-66 (2015).
958. Chen KY, *et al.* Estrogen receptor gene polymorphisms and lung adenocarcinoma risk in never-smoking women. *J Thorac Oncol* **10**, 1413-1420 (2015).
959. Zheng M, *et al.* Potentially functional polymorphisms in PAK1 are associated with risk of lung cancer in a Chinese population. *Cancer Med*, (2015).
960. Kong J, *et al.* Genetic polymorphisms in the vitamin D pathway in relation to lung cancer risk and survival. *Oncotarget* **6**, 2573-2582 (2015).
961. Su WM, *et al.* Single nucleotide polymorphisms in VTI1A gene contribute to the susceptibility of Chinese population to non-small cell lung cancer. *Int J Biol Markers* **30**, e286-e293 (2015).
962. Wang Y, Ren B, Zhang L, Guo Z. Correlation between metabolic enzyme GSTP1 polymorphisms and susceptibility to lung cancer. *Exp Ther Med* **10**, 1521-1527 (2015).
963. Yoo SS, *et al.* Putative functional variants of XRCC1 identified by RegulomeDB were not associated with lung cancer risk in a Korean population. *Cancer Genet* **208**, 19-24 (2015).
964. Modesto JL, *et al.* NNK reduction pathway gene polymorphisms and risk of lung cancer. *Mol Carcinog* **54**, E94-E102 (2015).
965. Ma QY, Chen J, Wang SH, Wu N, Hao ZH, Chen XF. Interleukin 17A genetic variations and susceptibility to non-small cell lung cancer. *APMIS* **123**, 194-198 (2015).
966. Shen W, *et al.* Polymorphisms in alternative splicing associated genes are associated with lung cancer risk in a Chinese population. *Lung Cancer* **89**, 238-242 (2015).
967. Yin J, *et al.* NFKB1 common variants and PPP1R13L and CD3EAP in relation to lung cancer risk in a Chinese population. *Gene* **567**, 31-35 (2015).
968. Wang H, Li Y, Yu W, Ma L, Ji X, Xiao W. Expression of the receptor for advanced glycation end-products and frequency of polymorphism in lung cancer. *Oncol Lett* **10**, 51-60 (2015).
969. Gately K, *et al.* Mutational analysis of the insulin‑like growth factor 1 receptor tyrosine kinase domain in non-small cell lung cancer patients. *Mol Clin Oncol* **3**, 1073-1079 (2015).
970. Wang Y, Jiang W, Liu X, Zhang Y. Tankyrase 2 (TNKS2) polymorphism

associated with risk in developing non-small cell lung cancer in a Chinese population. *Pathol Res Pract* **211**, 766-771 (2015).
971. Li X, *et al.* The interaction of APEX1 variant with polycyclic aromatic hydrocarbons on increasing chromosome damage and lung cancer risk among male Chinese. *Mol Carcinog* **54**, E103-E111 (2015).
972. Gansmo LB, Knappskog S, Hveem K, Vatten L, Lønning PE. Influence of MDM2 SNP309 and SNP285 status on the risk of cancer in the breast, prostate, lung and colon. *Int J Cancer* **137**, 96-103 (2015).
973. Sodhi KK, Bahl C, Singh N, Behera D, Sharma S. Functional genetic variants in pre-MIR-146a and 196a2 genes are associated with risk of lung cancer in North Indians. *Future Oncol* **11**, 2159-2173 (2015).
974. Javid J, *et al.* CC genotype of anti-apoptotic gene BCL-2 (−938 C/A) is an independent prognostic marker of unfavorable clinical outcome in patients with non-small-cell lung cancer. *Clin Transl Oncol* **17**, 289-295 (2015).
975. Wang Y, Ni J, Sun Z, Chen S, Jiao Y, Bai C. The influence of XRCC1 genetic variants on lung cancer susceptibility in Chinese Han population. *Gene* **556**, 127-131 (2015).
976. Liu Y, Qing H, Su X, Wang C, Li Z, Liu S. Association of CD44 gene polymorphism with survival of NSCLC and risk of bone metastasis. *Med Sci Monit* **21**, 2694-2700 (2015).
977. Zhang S, *et al.* Polymorphisms in C-reactive protein and glypican-5 are associated with lung cancer risk and gartrokine-1 influences cisplatin-based chemotherapy response in a Chinese Han population. *Dis Markers* **2015**, (2015).
978. Rafrafi A, *et al.* CCR2-64I polymorphism is associated with Non-Small Cell Lung Cancer in Tunisian patients. *Hum Immunol* **76**, 348-354 (2015).
979. Kowal A, Wiśniewski A, Kuśnierczyk P, Jankowska R. Human leukocyte antigen (HLA)-G gene polymorphism in patients with non-small cell lung cancer. *Thoracic Cancer* **6**, 613-619 (2015).
980. Cheng S, *et al.* Interleukin 17A polymorphism elevates gene expression and is associated with increased risk of nonsmall cell lung cancer. *DNA Cell Biol* **34**, 63-68 (2015).
981. Dimitrakopoulos FID, *et al.* Variant of BCL3 gene is strongly associated with five-year survival of non-small-cell lung cancer patients. *Lung Cancer* **89**, 311-319 (2015).
982. He Y, *et al.* IL-17A and IL-17F single nucleotide polymorphisms associated with lung cancer in Chinese population. *Clin Respir J*, (2015).
983. Liu C, Zhou X, Gao F, Qi Z, Zhang Z, Guo Y. Correlation of genetic polymorphism of vascular endothelial growth factor gene with susceptibility to lung cancer. *Cancer Gene Ther* **22**, 312-316 (2015).
984. Subhani S, Jamil K, Atilli S. Association of CYP3A4*1B and CYP3A5*3 Genetic Polymorphisms with Lung Cancer and its Impact on Taxane Metabolism in Indian Population. *Acta Medica International* **2**, 19-27 (2015).
985. Sevilya Z, *et al.* Development of APE1 enzymatic DNA repair assays: Low APE1 activity is associated with increase lung cancer risk. *Carcinogenesis* **36**, 982-991 (2015).
986. Wang SS, Zhu XQ, Yang SD, Dong LL, Li W, Tang J. Association of p73 G4C14-to-A4T14 polymorphism with non-small cell lung cancer risk. *Oncol Lett* **10**, 995-999 (2015).
987. Ture M, *et al.* Investigation of FGFR4 (Gly388Arg) gene polymorphism in

primary lung cancer patients. *Int J Hum Genet* **15**, 7-12 (2015).
988. Bhat IA, *et al.* Significant impact of IL-6-174G/C but inverse relation with-634 C/G polymorphism in patients with non-small cell lung cancer in kashmiri population. *Immunol Invest* **44**, 349-360 (2015).
989. Wang Y, Chen L, Pan L, Xue J, Yu H. The association between NFKB1-94ins/del ATTG polymorphism and non-small cell lung cancer risk in a Chinese Han population. *Int J Clin Exp Med* **8**, 8153-8157 (2015).
990. Wiśniewski A, *et al.* Genetic polymorphisms and expression of HLA-G and its receptors, KIR2DL4 and LILRB1, in non-small cell lung cancer. *Tissue Antigens* **85**, 466-475 (2015).
991. Cho EN, *et al.* BCL2-like 11 intron 2 deletion polymorphism is not associated with non-small cell lung cancer risk and prognosis. *Lung Cancer* **90**, 106-110 (2015).
992. Li Y, *et al.* IL1B gene polymorphisms, age and the risk of non-small cell lung cancer in a Chinese population. *Lung Cancer* **89**, 232-237 (2015).
993. Fazelzadeh Haghighi M, Ghayumi MA, Behzadnia F, Erfani N. Investigation of FOXP3 genetic variations at positions -2383 C/T and IVS9+459 T/C in southern iranian patients with lung carcinoma. *Iran J Basic Med Sci* **18**, 465-471 (2015).
994. Yin L, Guo H, Zhao L, Wang J. The programmed death-1 gene polymorphism (PD-1.5 C/T) is associated with non-small cell lung cancer risk in a chinese han population. *Int J Clin Exp Med* **7**, 5832-5836 (2014).
995. Liu J, Wang X, Ren Y, Li X, Zhang X, Zhou B. Effect of single nucleotide polymorphism Rs189037 in ATM gene on risk of lung cancer in Chinese: A case-control study. *PLoS One* **9**, (2014).
996. Liang Y, *et al.* Correlation of CLPTM1L polymorphisms with lung cancer susceptibility and response to cisplatin-based chemotherapy in a Chinese Han population. *Tumour Biol* **35**, 12075-12082 (2014).
997. Uppal V, Mehndiratta M, Mohapatra D, Grover RK, Puri D. XRCC-1 gene polymorphism (Arg399Gln) and susceptibility to development of lung cancer in cohort of north indian population: A pilot study. *J Clin Diagn Res* **8**, CC17-CC20 (2014).
998. Zhang L, *et al.* A functional polymorphism in the 3'-UTR of PXR Interacts with smoking to increase lung cancer risk in Southern and Eastern Chinese smoker. *Int J Mol Sci* **15**, 17457-17468 (2014).
999. Chen X, *et al.* RECK gene polymorphisms influence NSCLC susceptibility, but not the chemotherapy response status in Chinese cohort. *Cell Biochem Biophys* **69**, 567-571 (2014).
1000. Tian G, Wang M, Xu X. The role of NQO1 polymorphisms in the susceptibility and chemotherapy response of Chinese NSCLC patients. *Cell Biochem Biophys* **69**, 475-479 (2014).
1001. Gu JD, Hua F, Mei CR, Zheng DJ, Wang GF, Zhou QH. HapMap-based study on the association between MPO and GSTP1 gene polymorphisms and lung cancer susceptibility in Chinese Han population. *Acta Pharmacol Sin* **35**, 636-644 (2014).
1002. Wang SS, *et al.* Association between a p73 gene polymorphism and genetic susceptibility to non-small cell lung cancer in the South of China. *Asian Pac J Cancer Prev* **15**, 10387-10391 (2014).
1003. Luo X, *et al.* Genetic variant in CLPTM1L confers reduced risk of lung cancer: a replication study in Chinese and a meta-analysis. *Asian Pac J Cancer Prev* **15**, 9241-9247 (2014).

1004. Robles AI, *et al.* A DRD1 polymorphism predisposes to lung cancer among those exposed to secondhand smoke during childhood. *Chest* **146**, 1210-1218 (2014).
1005. Saikia BJ, *et al.* Association of a p53 codon 72 gene polymorphism with environmental factors and risk of lung cancer: a case control study in Mizoram and Manipur, a high incidence region in North East India. *Asian Pac J Cancer Prev* **15**, 10653-10658 (2014).
1006. Jiang XY, Chang FH, Bai TY, Lv XL, Wang MJ. Susceptibility of lung cancer with polymorphisms of CYP1A1, GSTM1, GSTM3, GSTT1 and GSTP1 genotypes in the population of Inner Mongolia region. *Asian Pac J Cancer Prev* **15**, 5207-5214 (2014).
1007. Lu DY, *et al.* RTN4 3'-UTR insertion/deletion polymorphism and susceptibility to non-small cell lung cancer in Chinese Han population. *Asian Pac J Cancer Prev* **15**, 5249-5252 (2014).
1008. Kang HG, *et al.* A functional polymorphism in CSF1R gene is a novel susceptibility marker for lung cancer among never-smoking females. *J Thorac Oncol* **9**, 1647-1655 (2014).
1009. Iwamoto S, *et al.* Association of EPAS1 gene rs4953354 polymorphism with susceptibility to lung adenocarcinoma in female Japanese non-smokers. *J Thorac Oncol* **9**, 1709-1713 (2014).
1010. Kaabachi W, *et al.* Association of vitamin D receptor FokI and ApaI polymorphisms with lung cancer risk in Tunisian population. *Mol Biol Rep* **41**, 6545-6553 (2014).
1011. Nasir Uddin MM, *et al.* Genetic polymorphisms of GSTM1, GSTP1 and GSTT1 genes and lung cancer susceptibility in the Bangladeshi population. *Asian Pac J Trop Biomed* **4**, 982-989 (2014).
1012. Phukan RK, Borah PK, Saikia BJ, Das M, Sekhon GS, Mahanta J. Interaction of tobacco smoking and chewing with Angiotensin converting enzyme (insertion/deletion) gene polymorphisms and risk of lung cancer in a high risk area from northeast India. *Asian Pac J Cancer Prev* **15**, 10691-10695 (2014).
1013. Zhang JW, *et al.* Association of CYP2E1 and NAT2 polymorphisms with lung cancer susceptibility among Mongolian and Han populations in the Inner Mongolian region. *Asian Pac J Cancer Prev* **15**, 9203-9210 (2014).
1014. Phukan RK, Saikia BJ, Borah PK, Zomawia E, Sekhon GS, Mahanta J. Role of household exposure, dietary habits and glutathione S-Transferases M1, T1 polymorphisms in susceptibility to lung cancer among women in Mizoram India. *Asian Pac J Cancer Prev* **15**, 3253-3260 (2014).
1015. Xun X, *et al.* CLPTM1L genetic polymorphisms and interaction with smoking and alcohol drinking in lung cancer risk: A case-control study in the Han population from Northwest China. *Medicine* **93**, e289 (2014).
1016. Cavic M, *et al.* The influence of methylenetetrahydrofolate reductase and thymidylate synthetase gene polymorphisms on lung adenocarcinoma occurrence. *J BUON* **19**, 1024-1028 (2014).
1017. Lou G, Zhang Y, Bao W, Deng D. Association between polymorphisms in CHRNA3 and PHACTR2 gene and environment and NSCLC risk in Chinese population. *Acta Biochim Pol* **61**, 765-768 (2014).
1018. Shukla RK, *et al.* Associations of CYP1A1, GSTM1 and GSTT1 polymorphisms with lung cancer susceptibility in a Northern Indian population. *Asian Pac J Cancer Prev* **14**, 3345-3349 (2013).
